# Global Conservation Priorities for Marine Turtles

**DOI:** 10.1371/journal.pone.0024510

**Published:** 2011-09-28

**Authors:** Bryan P. Wallace, Andrew D. DiMatteo, Alan B. Bolten, Milani Y. Chaloupka, Brian J. Hutchinson, F. Alberto Abreu-Grobois, Jeanne A. Mortimer, Jeffrey A. Seminoff, Diego Amorocho, Karen A. Bjorndal, Jérôme Bourjea, Brian W. Bowen, Raquel Briseño Dueñas, Paolo Casale, B. C. Choudhury, Alice Costa, Peter H. Dutton, Alejandro Fallabrino, Elena M. Finkbeiner, Alexandre Girard, Marc Girondot, Mark Hamann, Brendan J. Hurley, Milagros López-Mendilaharsu, Maria Angela Marcovaldi, John A. Musick, Ronel Nel, Nicolas J. Pilcher, Sebastian Troëng, Blair Witherington, Roderic B. Mast

**Affiliations:** 1 IUCN/SSC Marine Turtle Specialist Group – Burning Issues Working Group, Arlington, Virginia, United States of America; 2 Global Marine Division, Conservation International, Arlington, Virginia, United States of America; 3 Division of Marine Science and Conservation, Duke University, Beaufort, North Carolina, United States of America; 4 Marine Geospatial Ecology Laboratory, Duke University, Durham, North Carolina, United States of America; 5 Archie Carr Center for Sea Turtle Research and Department of Biology, University of Florida, Gainesville, Florida, United States of America; 6 Ecological Modelling Services, Pty Ltd, University of Queensland, Brisbane, Australia; 7 Unidad Académica Mazatlán, Instituto de Ciencias del Mar y Limnología, Universidad Nacional Autónoma de México, Mazatlán, Sinaloa, México; 8 Department of Biology, University of Florida, Gainesville, Florida, United States of America; 9 Island Conservation Society, Victoria, Mahé, Republic of Seychelles; 10 Marine Turtle Ecology and Assessment Program, Southwest Fisheries Science Center, NOAA-National Marine Fisheries Service, La Jolla, California, United States of America; 11 Centro de Investigación para el Medio Ambiente y Desarrollo, Cali, Colombia; 12 Laboratoire Ressources Halieutiques, IFREMER, Île de la Réunion, France; 13 Hawaii Institute of Marine Biology, Kaneohe, Hawaii, United States of America; 14 Banco de Información sobre Tortugas Marinas (BITMAR), Unidad Mazatlán, Instituto de Ciencias del Mar y Limnología, Universidad Nacional Autónoma de México, Mazatlán, Sinaloa, México; 15 Department of Biology and Biotechnology “Charles Darwin,” University of Rome “La Sapienza,” Rome, Italy; 16 WWF Mediterranean Turtle Programme, World Wildlife Fund-Italy, Rome, Italy; 17 Department of Endangered Species Management, Wildlife Institute of India, Dehradun, Uttarakhand, India; 18 World Wildlife Fund-Mozambique, Maputo, Mozambique; 19 Southwest Fisheries Science Center, National Marine Fisheries Service, National Oceanic and Atmospheric Administration, La Jolla, California, United States of America; 20 Karumbé, Montevideo, Uruguay; 21 Association RENATURA, Albens, France, and Pointe-Noire, Congo; 22 Laboratoire d'Ecologie, Systématique et Evolution, Université Paris-Sud, Orsay, France; 23 School of Earth and Environmental Sciences, James Cook University, Townsville, Australia; 24 Projeto Tamar-ICMBio/Fundação Pro Tamar, Salvador, Bahía, Brazil; 25 Department of Ecology, Institute of Biology, Universidade do Estado do Rio de Janeiro, Rio de Janeiro, Brazil; 26 Virginia Institute of Marine Sciences, College of William and Mary, Gloucester Point, Virginia, United States of America; 27 School of Environmental Sciences, Nelson Mandela Metropolitan University, Summerstrand Campus, South Africa; 28 Marine Research Foundation, Sabah, Malaysia; 29 Department of Animal Ecology, Lund University, Lund, Sweden; 30 Scientific Advisory Committee, Sea Turtle Conservancy, Gainesville, Florida, United States of America; 31 Florida Fish and Wildlife Conservation Commission, Melbourne Beach, Florida, United States of America; National Oceanic and Atmospheric Administration/National Marine Fisheries Service/Southwest Fisheries Science Center, United States of America

## Abstract

Where conservation resources are limited and conservation targets are diverse, robust yet flexible priority-setting frameworks are vital. Priority-setting is especially important for geographically widespread species with distinct populations subject to multiple threats that operate on different spatial and temporal scales. Marine turtles are widely distributed and exhibit intra-specific variations in population sizes and trends, as well as reproduction and morphology. However, current global extinction risk assessment frameworks do not assess conservation status of spatially and biologically distinct marine turtle Regional Management Units (RMUs), and thus do not capture variations in population trends, impacts of threats, or necessary conservation actions across individual populations. To address this issue, we developed a new assessment framework that allowed us to evaluate, compare and organize marine turtle RMUs according to status and threats criteria. Because conservation priorities can vary widely (i.e. from avoiding imminent extinction to maintaining long-term monitoring efforts) we developed a “conservation priorities portfolio” system using categories of paired risk and threats scores for all RMUs (n = 58). We performed these assessments and rankings globally, by species, by ocean basin, and by recognized geopolitical bodies to identify patterns in risk, threats, and data gaps at different scales. This process resulted in characterization of risk and threats to all marine turtle RMUs, including identification of the world's 11 most endangered marine turtle RMUs based on highest risk and threats scores. This system also highlighted important gaps in available information that is crucial for accurate conservation assessments. Overall, this priority-setting framework can provide guidance for research and conservation priorities at multiple relevant scales, and should serve as a model for conservation status assessments and priority-setting for widespread, long-lived taxa.

## Introduction

Major challenges for conservation of widely distributed, long-lived taxa are assessing conservation status at biologically appropriate scales and establishing conservation priorities based on those assessments [1]–[3]. However, current global extinction risk frameworks, most notably the IUCN *Red List of Threatened Species*™ (www.iucnredlist.org), are not designed to capture and assess variation in status and trends of individual populations of wide-ranging species (e.g. sharks [4], marine turtles [5], [6], marine mammals [7]). Thus, assessing the status of and threats to distinct population segments or management units of these species are critical steps toward building sound frameworks for setting conservation priorities [3].

Despite consisting of only seven species, marine turtles are circumglobally distributed, inhabit nearly all oceans, occupy unique ecological niches, and exhibit intra-specific variations in population sizes, and trends, as well as reproduction and morphology [3]. On a global scale, marine turtle species are currently listed as Vulnerable (olive ridley, *Lepidochelys olivacea*), Endangered (loggerhead, *Caretta caretta*; green turtle, *Chelonia mydas*), Critically Endangered (Kemp's ridley, *Lepidochelys kempii*; hawksbill, *Eretmochelys imbricata*; leatherback, *Dermochelys coriacea*), and Data Deficient (flatback, *Natator depressus*) on the *Red List* [8]. Threats to marine turtles vary across regions, but general categories include fisheries bycatch (i.e. incidental capture by marine fisheries operations targeting other species), take (e.g. utilization of eggs, meat or other turtle products), coastal development, pollution and pathogens, and climate change [9].

The IUCN Marine Turtle Specialist Group, one of the IUCN/Species Survival Commission's specialist groups, is responsible for conducting regular *Red List* assessments of each marine turtle species on a global scale. However, because marine turtle population traits—as well as environmental conditions—vary geographically [10], the global extinction risk assessment framework represented by the *Red List* does not adequately assess conservation status of spatially and biologically distinct marine turtle populations (see [5], [6] for review). The MTSG has debated the utility and validity of this global classification system for decades, and has advocated for regional assessments using criteria that are more appropriate for assessing extinction risk of marine turtle populations [5]. In fact, recent MTSG species assessments have attempted to address this problem by evaluating species status in each ocean basin based on data compiled at the sub-ocean basin level [11]–[14]. Thus, the MTSG has faced a two-fold challenge: 1) to define population units for assessments, and 2) to develop a system for assessing the conservation status of those population units.

To address these challenges, the MTSG leadership convened the Burning Issues Working Group (MTSG-BI) of marine turtle experts from around the world who represented government agencies, nongovernmental organizations, and academic institutions (for a brief history of MTSG-BI, see [15]). The MTSG-BI addressed the first challenge by developing Regional Management Units (RMUs) (i.e., spatially explicit population segments defined by biogeographical data of marine turtle species) as the framework for defining population segments for assessments [3]. Toward addressing the second challenge, the MTSG-BI developed criteria and a process for evaluating and prioritizing the conservation status of marine turtle RMUs. This paper describes the assessment criteria and process, as well as the results and their implications for conservation priority-setting for marine turtles worldwide.

## Methods

The framework and process for conservation status assessments of marine turtles was developed during two MTSG-BI Working Group meetings held during August 2008 and September 2009, and further refined after both meetings. Briefly, the framework consists of semi-quantitative scoring of criteria related to status of and threats to individual RMUs. Scoring relied upon publicly available data from nearly 1,300 papers, reports, abstracts, and other sources (published through early 2010; full citations available in Dataset S1), exhaustive compilation of data provided by recent MTSG *Red List* assessments, and expertise of MTSG-BI workshop participants, and was later refined during review by the entire MTSG membership. The overall status and threats scores were then used to plot all RMUs on continua from low-to-high risk (i.e., population viability, based on population characteristics and status; defined below) and low-to-high threats (i.e., direct and indirect anthropogenic impacts; defined below), which allowed for comparisons of conservation status among all RMUs, and both within and among species.

### Matrices, assessment criteria, and scoring

Characteristics of populations (e.g. abundance, trends, vulnerability) and relative impacts of threats to populations are vital components to assessments of extinction risk. With this in mind, we first established two different matrices that would frame the evaluation process: one to evaluate population characteristics and status for each RMU (i.e. risk of decline based on a suite of traits; i.e. “the risk matrix”) and another to evaluate threats to each RMU (i.e., “the threats matrix”). The risk matrix evaluated population characteristics according to relative risk of population decline or loss of genetic diversity, while the threats matrix evaluated the relative impacts of different threats to RMUs. Although ‘hazards’ is the preferred term in risk-assessment literature [16], we used ‘threats’ as this is the more prevalent term in the conservation biology community.

To semi-quantitatively assess risk and threats for all RMUs, we established relevant criteria within each matrix. We scored all criteria on a 1 to 3 scale and calculated overall risk and threats scores for each RMU to compare overall scores among RMUs. If insufficient information was available for a score to be made for a criterion (e.g., no citations were available), it was scored as ‘data deficient’ or ‘DD.’ Risk and threat criteria scores for all RMUs are provided in Dataset S2. Significance of numerical values and scales is explained below.

#### Risk matrix criteria

In the risk matrix, we wanted not only to evaluate some direct measures of population viability (e.g. abundance and trends), but also other factors that are important considerations for conservation strategies, such as genetic diversity. Thus, the five criteria established within the risk matrix were: 1) population size, 2) recent trend, 3) long-term trend, 4) rookery vulnerability, and 5) genetic diversity. We scored risk criteria (defined below) according to relative risk to each RMU conveyed by each criterion, with risk increasing from 1 (low) to 3 (high). Thus, average ‘low-risk’ criteria scores (closer to 1) would correspond to large, increasing, genetically diverse RMUs, while ‘high-risk’ criteria scores (closer to 3) would correspond to small, decreasing, low diversity RMUs.

Because the common currency for monitoring and evaluating population status of marine turtles is annual abundance counts of nesting females [17], the risk criteria we used were based on available information from rookeries for all species. We used georeferenced nesting sites for all species available via the State of the World's Sea Turtles – SWOT database (http://seamap.env.duke.edu/swot), which relies on a global network of researchers who voluntarily contribute annual nesting data (nearly 3,000 distinct sites; a complete list of SWOT data providers is included in Dataset S3). We augmented the SWOT database with published information. Further details about the use of the SWOT nesting database for these analyses are available in reference 3.

We assessed population sizes based on average annual number of nesting females in each RMU, with scores of ‘1’ corresponding to the largest abundance bins for each species on a global scale, and scores of ‘3’ corresponding to the smallest abundance bins for each species on a global scale. Abundance bins were generally established by orders of magnitude, but we allowed for multiple bins where necessary to allow more refined assessments of population sizes for most species (Table S1). Differences in abundance bins reflect variation among species in relative abundance; e.g. the enormous mass nesting rookeries of *Lepidochelys* spp. [13]. Where multiple nesting populations were included within RMUs, we summed available abundance values and assigned the RMU with a score based on this cumulative abundance.

We scored recent trends—defined as the nesting population trend based on the past 10 years of available nesting data reported in the literature for each RMU through early 2010—as significantly increasing (score of 1), stable (score of 2), or significantly declining (score of 3). We included recent trends because these can be indicative of acute drivers of changes in population trends. In addition, short-term trends are more readily available for most rookeries and RMUs.

We scored long-term trends—defined as the nesting population trend based on a minimum timespan of one generation (“generation” as defined by IUCN *Red List* criteria) of available nesting data reported in the literature for each RMU through early 2010—as significantly increasing (score of 1), stable (score of 2), or significantly declining (score of 3). Although less frequently available for rookeries and RMUs, long-term trends better represent marine turtle population dynamics than recent trends [17], [18].

We scored rookery vulnerability—defined as the likelihood of extirpation of functional rookeries that would prevent recovery based on the number and distribution of rookeries within an RMU—as low (score of 1), medium (score of 2), or high (score of 3). This criterion was intended to assess the relative density of rookeries within the spatial extent of an RMU as an indicator of persistence of viable nesting in an RMU given various threats and potential for range shifts over time.

We scored genetic diversity—defined as the number of known or inferred genetic stocks (from species-specific patterns of genetic distinctiveness among rookeries based on analyses of mitochondrial DNA) within an RMU—as high (>2 stocks, score of 1), medium (2 stocks, score of 2), or low (1 stock, score of 3). This criterion was intended to assess the genetic uniqueness maintained within RMUs, and to reflect higher risk of loss of isolated genetic stocks.

#### Threats matrix criteria

For the threats matrix, we used the ‘Five Hazards to Marine Turtles’ established during BI-3 [9]: 1) fisheries bycatch, 2) take, 3) coastal development, 4) pollution and pathogens, and 5) climate change. We scored threats criteria according to relative impact to each RMU from that criterion, with all threat scores increasing from 1 (low) to 3 (high). Threats were scored separately for each RMU, rather than among RMUs. If insufficient information was available for a score to be made for a criterion, it was scored as data deficient (see below).

We scored fisheries bycatch, or incidental capture of marine turtles in fishing gear targeting other species, in terms of population-level impacts, taking into account the magnitude and mortality rates of reported bycatch, as well as life-stages affected. Bycatch was scored low = 1, medium = 2, high = 3, and when bycatch was scored as ‘high,’ we specified the gear type(s) that contributed most to this assessment.

We scored take—defined to include direct utilization of turtles or eggs for human use (i.e. consumption, commercial products) relative to population size—as low = 1, medium = 2, or high = 3. When take was scored as ‘high,’ we specified the type(s) of take contributing most to this assessment: a) egg and hatchling loss (feral animals); b) egg utilization (legal and illegal); c) nesting female take; d) adult/immature take).

We scored coastal development—defined to include human-induced alteration of coastal environments due to construction, dredging, beach modification, etc. —as low = 1, medium = 2, or high = 3. When coastal development was scored as ‘high,’ we specified the type(s) of development contributing most to this assessment.

We scored pollution and pathogens—defined as marine pollution and debris that affect marine turtles (i.e. through ingestion or entanglement, disorientation caused by artificial lights, making them more susceptible to infections), as well as impacts of pervasive pathogens (e.g. fibropapilloma virus) on turtle health—as low = 1, medium = 2, or high = 3. When pollution and pathogens was scored as ‘high,’ we specified the type(s) contributing most to this assessment.

We scored climate change impacts—defined as current and future impacts from climate change on marine turtles and their habitats (e.g. increasing sand temperatures on nesting beaches affecting hatchling sex ratios, sea level rise, storm frequency and intensity affecting nesting habitats, etc.)—as low = 1, medium = 2, or high = 3. When climate change was scored as ‘high,’ we specified the impact(s) contributing most to this assessment.

After initial rounds of scoring threats criteria, we noted an excessive number of data deficient scores for pollution and pathogens (33 of 58 RMUs; 57%) and climate change (38 of 58 RMUs; 66%) (Table 1). With these findings in mind, we determined that in cases where these threats had been given a score, they were disproportionately influential in overall threats scores compared to RMUs for which those threats had not been scored (i.e. scored ‘DD’). Thus, we decided to omit these threats from the calculation of overall threats scores for all RMUs; threats scores and threats data uncertainty indices (defined in next section) were then the average of scores for fisheries bycatch, take, and coastal development. However, we emphasize that enhanced monitoring of impacts to marine turtles from threats of pollution and pathogens as well as climate change are critical data gaps to improve future conservation status assessments [19].

**Table 1:** Average scores and number of RMUs scored for all criteria in risk and threats matrices.

*RISK SCORES*					
	population size	recent trend	long-term trend	rookery vulnerability	genetic diversity
**mean**	1.95	1.81	2.47	1.72	1.90
**No. RMUs scored**	58	43	38	57	58

*THREATS SCORES*					
	fisheries bycatch	take	coastal development	pollution and pathogens	climate change
**mean**	2.21	2.08	1.93	1.70	2.20
**No. RMUs scored**	56	57	53	25	20

Pollution and pathogens and climate change were omitted from calculations and categorizations (see Methods for descriptions of criteria and calculations).

#### Data uncertainty index

To account for data deficiencies and quality issues, we included information on data sources of all criteria scores. For a RMU to be ranked in a conservation priority category (see below), it must have received numeric scores for ≥3 criteria in the risk matrix and for ≥2 threats in the threats matrix. If an RMU failed to meet this threshold, it was omitted from scoring but included as a ‘critical data need’ (see below).

For each numeric score, we assessed the relative quality of available data used to assess each criterion, and provided all related literature citations (complete bibliography in Dataset S1, which includes full citations for all criteria scores and associated citations displayed in Dataset S2). Specifically, we combined data quality and data deficient (DD) scores for each RMU, to tabulate a ‘data uncertainty index’ that accompanied each overall risk or threats score. We assessed the data uncertainty index as the sum of a) the DD score, which was the proportion of DD scores for an RMU relative to the total number of criteria (i.e. five for risk, three for threats) for each matrix (range from 0 to 1), and b) the data quality score, which was the average of the data quality scores for all numerically scored criteria in each matrix, where low data quality = 1, medium = 0.5, and high = 0. Data quality was assessed as low (i.e. background information “*in litt*,” grey literature, expert opinion; no peer-reviewed publications specifically dealing with criterion in question), medium (i.e. available information included <50% of RMU population abundance; incomplete spatio-temporal coverage of RMU; combination of grey literature and some peer-reviewed publications), or high (i.e. available information included >50% of RMU population abundance; extensive peer-reviewed publications on both long-term monitoring of nesting, migrations, at-sea behavior and threats assessments). Thus, data uncertainty indices could range from 0 to 2 units, increasing with uncertainty in available data to facilitate visualization of the data uncertainty index in plots of risk versus threats scores. A paired score for an RMU had ‘lower uncertainty (i.e. higher reliability)’ if the data uncertainty index’ <1, and as ‘higher uncertainty (i.e. lower reliability)’ if the data uncertainty index ≥1. In this way, data needs for RMUs could be assessed within conservation priority portfolio categories.

#### Conservation Priorities Portfolio

**Figure 1:**
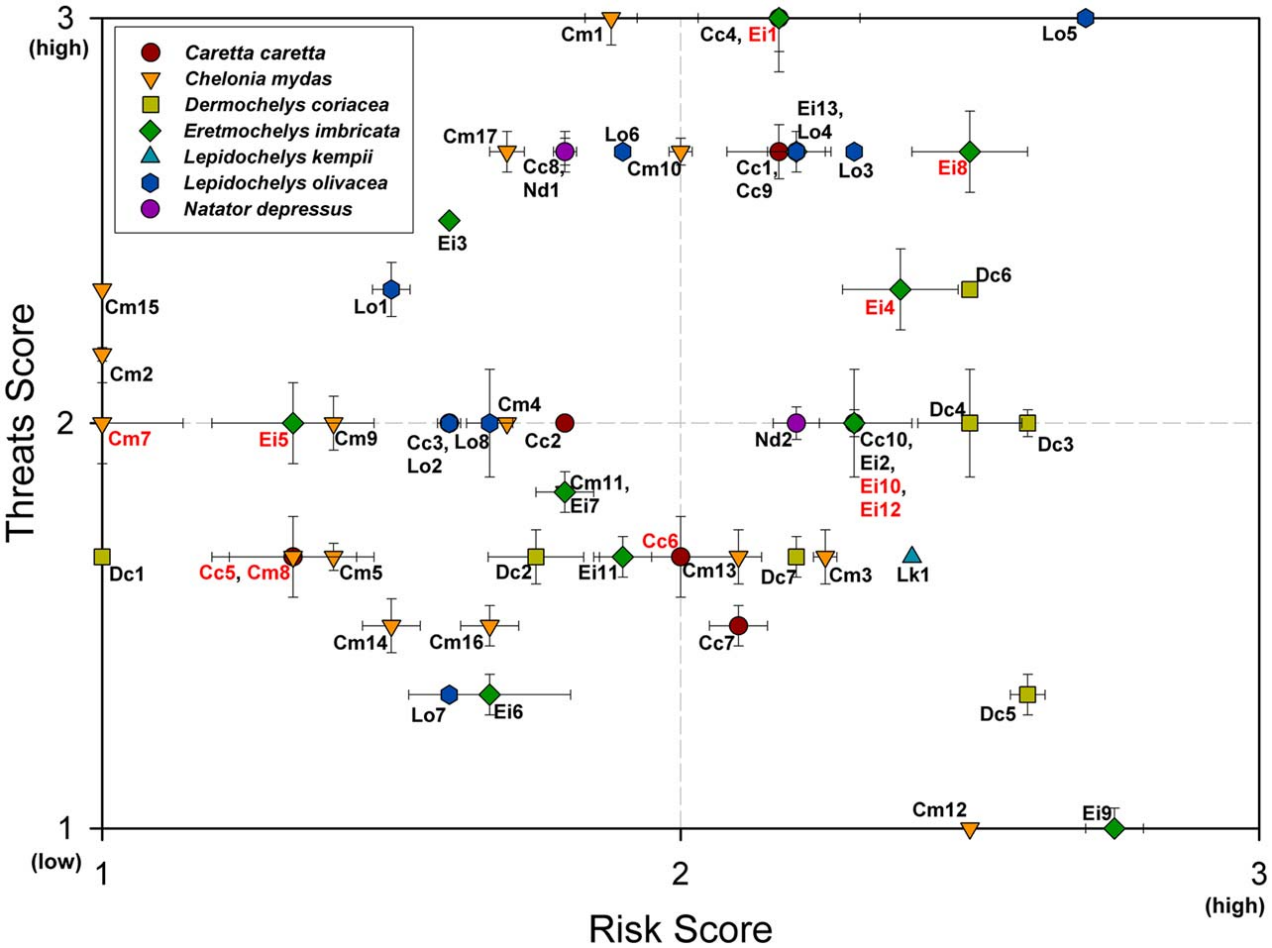
Conservation priority portfolio approach to displaying and interpreting paired risk (i.e. population viability characteristics) and threats scores (i.e., direct and indirect anthropogenic impacts), for marine turtle RMUs (see Table S3 for RMU codes). The four categories are: High risk-High threats, High risk-Low threats, Low risk-Low threats, Low risk-High threats; see Methods for more details on portfolio categories. RMUs were also classified as critical data needs if data uncertainty indices for both risk and threats ≥1 (denoting high uncertainty). Vertical and horizontal bars associated with each paired score represent the data uncertainty index; RMU IDs in red denote critical data needs (see Methods for details on how this was calculated). Where multiple RMUs have identical scores, RMU IDs are listed together, separated by commas. NOTE: *C. mydas*, Northeast Indian Ocean RMU was not plotted due to excessive data deficient scores.

Because conservation priorities can vary from avoiding imminent extinction, to conserving genetic diversity, to maintaining long-term monitoring efforts, to identifying assessment needs, we developed a ‘conservation priorities portfolio’ using combinations of scores from the risk and threats matrices for all RMUs. Specifically, for each RMU we plotted the average of scores for threats criteria against the average of scores for risk criteria, where each axis was on a scale of low to high (1 to 3). Scores fell within one of four quadrants that corresponded to four portfolio categories: 1) High Risk-High Threats; 2) High Risk-Low Threats; 3) Low Risk-Low Threats; 4) Low Risk-High Threats (Figure 1). If an RMU fell on the border between two categories, we applied a precautionary approach and assigned it to the higher-risk or higher-threat category. RMUs with data uncertainty scores ≥1 for both risk and threats were also identified as ‘critical data needs RMUs,’ in addition to being assigned to one of the other categories.

## Results and Discussion

Below we present results of the risk and threats assessments globally, by species, and by ocean basin to identify patterns in risk, threats, and data needs at different scales. In addition, we present results according to recognized MTSG regions (http://iucn-mtsg.org/regions/), as well as by UN Food and Agriculture Organization (FAO)-recognized Regional Fisheries Bodies (RFBs) with management mandates (http://www.fao.org/fishery/rfb/search/en), to determine patterns in risk, threats, and data needs according to relevant geographies and geopolitical bodies with potential to implement conservation strategies to address identified needs.

### Global-scale summary

We assessed the risk and threats scores for 58 RMUs (Table 1; Fig. 1, see Table S2 for RMU codes; Dataset S2). Including all RMUs, average scores of risk criteria were moderate, except for that of long-term trend, which reflected an overall pattern of population declines across species globally over the past generation. In contrast, average recent trend was near stable, and even slightly increasing (stable = 2, overall average recent trend = 1.81), perhaps reflecting an encouraging trend of recent conservation successes for some RMUs (e.g. [18]). As for threats criteria, average scores for fisheries bycatch and climate change ranked highest, although climate change was scored in only one-third of RMUs. Pollution and pathogens was ranked lowest among threats criteria, although it was scored in less than half of RMUs (Table 1). Our results agreed somewhat with a recent expert-based survey ranking anthropogenic threats to marine turtles in which respondents consistently ranked bycatch and coastal development as the most important threats, whereas pathogens (considered separately from pollution) was almost never ranked as a high threat [20].

Overall, 19 of the 58 total RMUs were categorized as High Risk-High Threats, nine as High Risk-Low Threats, 12 as Low Risk-Low Threats, and 17 as Low Risk-High Threats (Fig. 1). One RMU (*C. mydas*, Northeast Indian Ocean) was not scored because of excessive data deficient scores (three risk criteria scored DD). Thus, nearly two-thirds of scored RMUs (36 of 57) were categorized as High Threats. Twelve RMUs (including *C. mydas*, Northeast Indian Ocean) were assessed as critical data needs (Fig. 1; Table S3).

Of those categorized High Risk-High Threats, 11 RMUs fell completely within the quadrant boundaries (Fig. 1), and thus can be considered the most endangered marine turtle RMUs in the world (Table 2). The other categories of conservation priorities reflect different risk and threats scores and thus merit different conservation interventions, but these 11 RMUs are, overall, those with population characteristics of highest risk that are simultaneously under the highest degree of threats, and therefore are in the most danger of extinction. Of these 11 RMUs, five occur in the Indian Ocean, and four are *E. imbricata*.

**Table 2:** The world's 11 most endangered RMUs (grouped by ocean basin).

Regional Management Unit
*Lepidochelys olivacea*, West Indian Ocean
*Caretta caretta*, Northeast Indian Ocean
*Lepidochelys olivacea*, Northeast Indian Ocean
*Lepidochelys olivacea*, Northeast Indian Ocean (arribadas)
*Eretmochelys imbricata*, Northeast Indian Ocean
*Eretmochelys imbricata*, East Atlantic Ocean
*Caretta caretta*, Northeast Atlantic Ocean (Cape Verde)
*Eretmochelys imbricata*, East Pacific Ocean
*Dermochelys coriacea*, East Pacific Ocean
*Caretta caretta*, North Pacific Ocean
*Eretmochelys imbricata*, West Pacific Ocean

### Assessments by species

**Figure 2:**
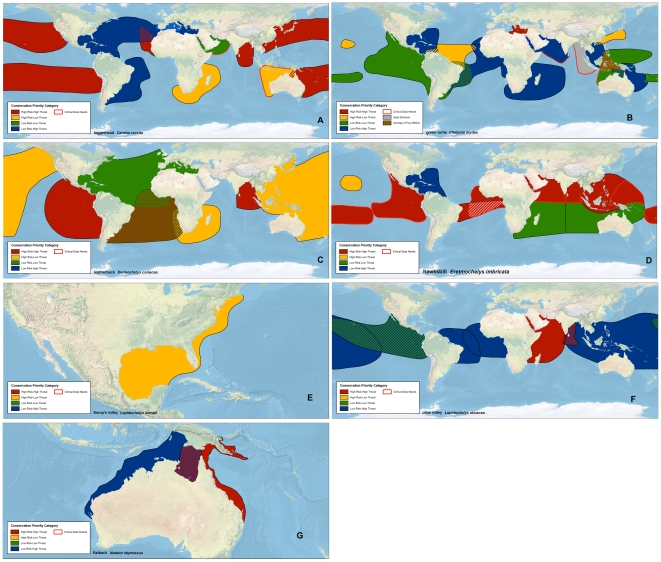
Conservation priority portfolio categories for RMUs of each marine turtle species. (A) loggerheads (*Caretta caretta*), (B) green turtles (*Chelonia mydas*), (C) leatherbacks (*Dermochelys coriacea*, (D) hawskbills (*Eretmochelys imbricata*), (E) Kemp's ridleys (*Lepidochelys kempii*), (F) olive ridleys (*Lepidochelys olivacea*), (G) flatbacks (*Natator depressus*). RMUs were classified as critical data needs if the data uncertainty indices for both risk and threats ≥1 (denoting high uncertainty), and are outlined in red. Hatched areas represent spatial overlaps between RMUs. The brown area in Fig. 2B highlights an overlap of four RMUs, while the grey area in Fig. 2B represents the *C. mydas* Northeast Indian Ocean RMU, which had excessive data deficient scores and was not included in overall calculations and categorization.

Risk scores ranged from 1.00 (*C. mydas*, northwest Indian Ocean; *D. coriacea*, Northwest Atlantic Ocean) to 2.70 (*L. olivacea*, West Indian Ocean), while threats scores ranged from 1.00 (*C. mydas*, Central North Pacific Ocean [Hawaii]; *E. imbricata*, Central North Pacific Ocean [Hawaii]) to 3.00 (*C. mydas* and *E. imbricata*, East Atlantic Ocean; *C. caretta*, Northeast Indian Ocean; *L. olivacea*, West Indian Ocean (Fig. 2; Table S3).

Conservation portfolio categories of RMUs for each species are displayed in map (Fig. 2) and graphical formats (Fig. S1). The High Risk-High Threats category was defined to identify the RMUs with low, declining abundance and low diversity simultaneously under high threats. These RMUs can be considered as warranting the most urgent conservation intervention because of this combination of high risk and high threats. More than half of *E. imbricata* RMUs (e.g. East Atlantic Ocean, East Pacific Ocean) and roughly 40% of *C. caretta* RMUs (e.g. Northeast Atlantic Ocean, Northeast Indian Ocean, North Pacific Ocean) and *D. coriacea* RMUs (e.g. East Pacific Ocean) were categorized as High Risk-High Threats (Figs. 1 and 2; Table S3). Only *L. kempii*, with just one RMU, did not have at least one RMU in this most urgent conservation category (Fig. 2E; Fig. S1F).

High Risk-Low Threats RMUs were characterized generally by low, declining abundance and low diversity, i.e. characteristics that make them more susceptible to population decline or loss, particularly if impacts from threats increase in severity. This category included *L. kempii*, Northwest Atlantic Ocean, *C. mydas*, Northwest Pacific, *D. coriacea*, Southwest Indian Ocean, and both RMUs (*C. mydas* and *E. imbricata*) from the Central North Pacific Ocean (Hawaii) (Figs. 1 and 2; Table S3).

RMUs categorized as Low Risk-Low Threats were characterized as having high and stable or increasing abundance, high diversity, while being under low to moderate threats. This category is intended to highlight large populations that, in many cases, are well-monitored and thus represent continued opportunities to generate valuable information about population abundances and trends, as well as other biological data, for all species that can be applied to situations where such information is unavailable. Low Risk-Low Threats included five *C. mydas* RMUs (e.g. South Central and West Central Pacific Ocean), three *E. imbricata* RMUs (e.g. Southwest Pacific Ocean), two *D. coriacea* RMUs (Northwest Atlantic and Southeast Atlantic), and one each for *C. caretta* (Northwest Indian Ocean) and *L. olivacea* (East Pacific Ocean arribada RMU) (Figs. 1 and 2; Table S3).

Low Risk-High Threats RMUs generally exhibited large, stable or increasing abundance with high diversity while under a relatively high degree of threats. As such, this category highlighted RMUs that are robust at present, but if threats are not abated, could decline in the future, thus warranting intervention before significant population-level impacts can manifest. The Low Risk-High Threats category included seven *C. mydas* RMUs (e.g. East Atlantic Ocean, West and Southwest Pacific Ocean), four *L. olivacea* RMUs (e.g. East Atlantic Ocean, East Pacific Ocean solitary nesters), three *C. caretta* RMUs (e.g. Mediterranean Sea), two *E. imbricata* (e.g. West Atlantic Ocean), and one *N. depressus* (Southeast Indian Ocean) (Figs. 1 and 2; Table S3).

Six of 13 *E. imbricata* RMUs, two of 10 *C. caretta* RMUs, and three of 17 *C. mydas* RMUs were classified as critical data needs due to excessively high uncertainty in available data (Figs. 1 and 2; Dataset S2).

### Assessments by ocean basin

When considering ocean basin scales (i.e. Atlantic Ocean and Mediterranean Sea, Indian Ocean, Pacific Ocean), RMUs in the Pacific Ocean had the highest average risk score (2.03), while RMUs in the Atlantic Ocean (including the Mediterranean) had the highest average threats score (2.16). RMUs in the Indian Ocean had the highest average data uncertainty scores for both risk and threats (Table 3).

**Table 3:** Average risk and threats scores (and accompanying data uncertainty indices) of RMUs that occur in each ocean basin.

ocean basin	average risk score	averagerisk scoredata uncertainty	averagethreats score	averagethreats scoredata uncertainty
**Atlantic/Med (n = 19)**	1.81	0.26	2.16	0.35
**Indian (n = 18)** *	1.92	0.78	2.08	0.68
**Pacific (n = 21)**	2.03	0.32	1.96	0.48

*One RMU (*C. mydas* northeast Indian Ocean) not scored.

**Figure 3:**
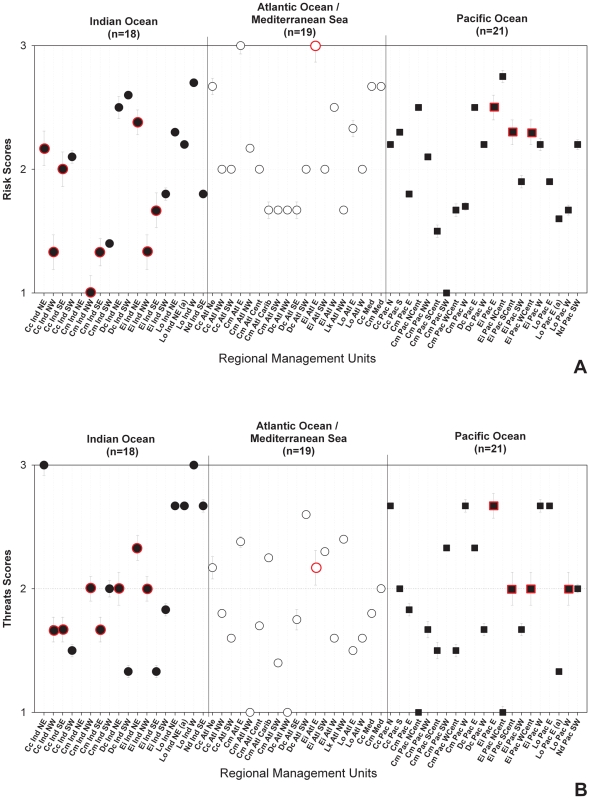
Risk (i.e. population viability) scores (A) and threats (i.e. direct and indirect anthropogenic impacts) scores (B) with data uncertainty indices by ocean basin. Symbols bordered in red are scores with accompanying data uncertainty indices that exceed 1 (see Methods for details). Refer to Table S3 for list of RMU IDs. NOTE: *C. mydas* Northeast Indian Ocean RMU was not plotted due to excessive data deficient scores.

All basins were represented in relatively similar proportions among categories, except for critical data needs, which occurred most frequently in the Indian Ocean (Table 4). Specifically, among Indian Ocean RMUs, data uncertainty was frequently scored as high for both risk (eight of 17 RMUs scored; Fig. 3A) and threats (seven of 18 RMUs scored; Fig. 3B), while no more than three RMUs in the other ocean basins had high data uncertainty scores.

**Table 4:** Categories in which RMUs occurred in each basin (including critical data needs RMUs).

		Categories				
ocean basin	critical data needs	HR-HT	HR-LT	LR-LT	LR-HT	Total
**Atlantic/Med (n = 19)**	1	5	2	3	9	19
**Indian (n = 18)** *	8	6	3	4	4	17*
**Pacific (n = 21)**	3	8	4	5	4	21
**Total**	**12**	**21**	**9**	**12**	**15**	**57** *

Categories: HR-HT = High Risk-High Threats; HR-LT = High Risk-Low Threats; LR-LT = Low Risk-Low Threats; LR-HT = Low Risk-High Threats.

*One RMU (*C. mydas*, northeast Indian Ocean) was scored critical data needs only.

Although extremely coarse geographically, our analyses by ocean basin suggest some relevant patterns, especially in regard to data uncertainty and data gaps. Specifically, risk and threats scores for RMUs in the Indian Ocean were associated with the lowest availability and quality of data among ocean basins (risk data uncertainty = 0.78; threats data uncertainty = 0.68). If RMUs from the Southwest Indian Ocean were removed from the calculations, data uncertainty increased further (risk data uncertainty = 0.91; threats data uncertainty = 0.73). This discrepancy between RMUs in the Southwest Indian Ocean compared to RMUs from the rest of the basin reflects the difference between the relative presence [21]–[23] and absence [24], respectively, of long-term monitoring initiatives in these sub-regions.

### Assessments by MTSG regions

To put analyses in a context of recommending future strategies to address conservation and data needs within the construct of the MTSG, we assessed risk and threats for RMUs occurring within existing MTSG regions (http://iucn-mtsg.org/regions/). RMUs were counted in each region in which they occurred.

**Figure 4:**
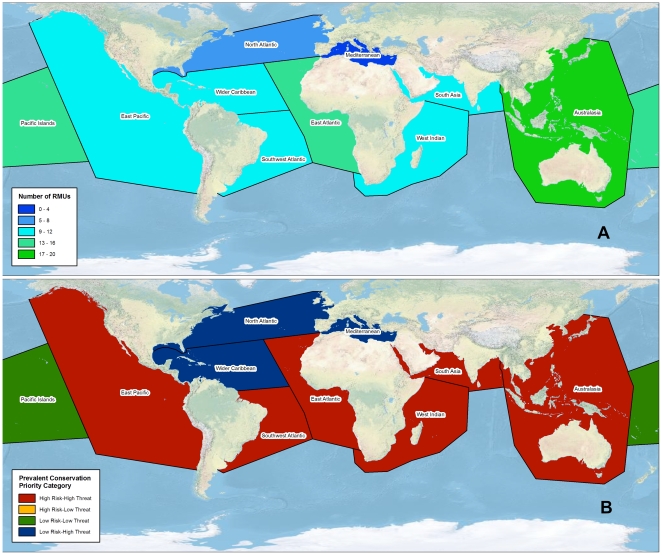
Conservation status assessments of marine turtle RMUs in regions recognized by the IUCN Marine Turtle Specialist Group (MTSG). (A) number of RMUs that occur within MTSG regions; (B) most prevalent conservation priority portfolio category (see Methods and Fig. 1 for descriptions) for RMUs that occur within each region.

Australasia was the most RMU-diverse region, with 20 RMUs occurring within its boundaries, while the Mediterranean was the least diverse region, with four RMUs (Table 5; Fig. 4A). The diversity of RMUs occurring in Australasia (n = 20) and the Pacific Islands (n = 15) might be attributed to the prevailing geographies of archipelagoes and the extensive coastlines present in these regions. The East Atlantic region (n = 16 RMUs) also showed high diversity, due not only to the extensive coastline of continental Africa, but also to its variation of foraging areas; several RMUs whose nesting sites are in the West Atlantic demonstrate trans-Atlantic connectivity with foraging and developmental areas in the East Atlantic [25]–[27]. That the two regions at highest latitudes—North Atlantic and Mediterranean—showed the lowest RMU diversity is not surprising, given that marine turtle distributions are most concentrated in the tropics and decrease with increasing latitudes [3].

**Table 5:** Conservation Priorities Portfolio results by MTSG regions.

MTSG Region	No. RMUs	critical data needs RMUs	average risk score	average risk score data uncertainty	average threats score	average threats score data uncertainty	*most prevalent category
North Atlantic	7	0	1.68	0.17	2.19	0.17	LR-HT
East Atlantic	16	1	1.94	0.33	2.09	0.44	HR-HT
Mediterranean	4	0	1.65	0.10	2.25	0.17	LR-HT
Wider Caribbean	12	1	1.81	0.26	2.06	0.28	LR-HT
Southwest Atlantic	12	1	1.81	0.26	2.00	0.35	LR-HT
South Asia**	12	5	1.94	0.74	2.39	0.74	HR-HT
Australasia	20	5	1.96	0.57	2.11	0.66	HR-HT
West Indian	12	3	1.93	0.53	2.03	0.51	HR-HT
East Pacific	11	2	2.14	0.27	2.01	0.47	HR-HT
Pacific Islands	15	2	1.96	0.27	1.81	0.47	LR-LT

*Categories: HR-HT = High Risk-High Threats; HR-LT = High Risk-Low Threats; LR-LT = Low Risk-Low Threats; LR-HT = Low Risk-High Threats.

**One RMU (*C. mydas*, northeast Indian Ocean) was scored critical data needs only.

As with global averages for species and ocean basins, average risk and threats scores for regions clustered around medium values (i.e. ∼2) (Table 5). Average risk scores ranged from 1.68 (North Atlantic) to 2.14 (East Pacific), and Average threat scores ranged from 1.81 (Pacific Islands) to 2.39 (South Asia) (Table 5). The most prevalent category among RMUs within regions was High Risk-High Threats (five regions), followed by Low Risk-High Threats (four regions); the most prevalent category among Pacific Islands RMUs was Low Risk-Low Threats (Table 5; Fig. 4B).

South Asia had the highest proportion of RMUs categorized as critical data needs (∼40%), followed by the West Indian Ocean (25%) and Australasia (20%); however, some RMUs occurred in more than one of these regions, probably contributing to the similarities (Table 5). The only regions with no critical data needs RMUs were the North Atlantic and the Mediterranean. South Asia RMUs also had the highest average data uncertainty for both risk and threats—corroborating results at the ocean basin scale (Table 3)—whereas the lowest uncertainty scores were associated with RMUs from the Mediterranean and North Atlantic (Table 5). The low data uncertainty in these latter regions is probably due to the fact that the regions are comprised predominantly of developed countries, and are characterized by several long-term monitoring projects [22], [28]–[30].

### Assessments by international management frameworks

Due to their highly migratory, geographically widespread nature, marine turtles warrant trans-boundary conservation strategies that often include multiple institutions and governing bodies, spanning several geopolitical borders, agreements, and instruments at local, national, and international scales [31], [32]. Navigating this complex management framework requires knowledge about the distributions, status, and trends of marine turtle populations that occur within various relevant borders [3].

The RMU-conservation portfolio framework can be applied by various geopolitical entities at different scales to inform management strategies toward marine turtle conservation. To demonstrate this potential, we assessed risk and threats (specifically fisheries bycatch) to RMUs occurring in Regional Fisheries Bodies (RFBs) that have mandates for management of marine resources within their Areas of Competence (Fig. S2; for complete lists Areas of Competence of RFBs by ocean basin, see FAO fishery governance fact sheets: http://www.fao.org/fishery/rfb/search/en). RMUs were counted in each RFB in which they occurred.

All 18 RFBs with management mandates had at least one RMU within their Areas of Competence, and several had more than 20 (ICCAT, CCSBT, IOTC, and WCPFC) (Table 6). Conversely, nearly all RMUs (with the exception of four) occurred in at least two RFBs, and one (*D. coriacea*, West Pacific Ocean) occurred in nine RFBs. Overall, the RFBs dedicated to management of tuna stocks (IATTC, ICCAT, CCSBT, IOTC) showed high RMU diversity (Table 6). This pattern was likely due to the broad geographic extents of these RFBs across multiple habitats utilized by marine turtles and other highly migratory, pelagic animals, such as tuna, thus demonstrating the importance of effective ecosystem-based management strategies at broad scales for these taxa [2], [33].

**Table 6:** Conservation Priorities Portfolio results by Regional Fisheries Bodies with a management mandate.

RFB	No. RMUs	No. critical data needs RMUs**	average risk scores	average bycatch scores	*most prevalent category
CCAMLR	1	0	2.60	2.00	HR-HT
CCBSP	1	0	2.20	2.00	HR-HT
CCSBT	22	3	1.89	2.07	HR-LT
GFCM	4	0	1.65	3.00	LR-HT
IATTC	13	2	2.13	2.08	HR-HT
ICCAT	22	1	1.88	2.52	LR-HT
IOTC	25	7	1.91	2.19	HR-HT
IPHC	2	0	2.20	2.50	HR-HT
NAFO	5	0	1.56	2.20	HR-HT
NASCO	7	0	1.68	2.43	HR-HT and LR-HT
NEAFC	4	0	1.69	3.00	HR-HT
NPFAC	2	0	2.20	2.50	LR-LT
PSC	2	0	2.20	2.50	LR-HT
RECOFI	4	3	1.59	2.50	HR-HT
SEAFO	14	1	1.86	2.54	LR-HT
SIOFA	12	6	1.88	2.09	LR-HT
SPRFMO	11	2	1.93	2.20	HR-HT
WCPFC	27	6	2.00	1.84	HR-HT and HR-LT

*Categories: HR-HT = High risk-High threats; HR-LT = High risk-Low threats; LR-LT = Low risk-Low threats; LR-HT = Low risk-High threats. RFB acronyms: CCAMLR: Commission on the Conservation of Antarctic Marine Living Resources; CCBSP: Convention on the Conservation and Management of Pollock Resources in the Central Bering Sea; CCSBT: Commission for the Conservation of Southern Bluefin Tuna; GFCM: General Fisheries Commission for the Mediterranean; IATTC: Inter-American Tropical Tuna Commission; ICCAT: International Commission for the Conservation of Atlantic Tunas; IOTC: Indian Ocean Tuna Commission; IPHC: International Pacific Halibut Commission; NAFO: Northwest Atlantic Fisheries Organization; NASCO: North Atlantic Salmon Conservation Organization; NEAFC: Northeast Atlantic Fisheries Commission; NPFAC: North Pacific Anadromous Fish Commission; PSC: Pacific Salmon Commission; RECOFI: Regional Commission for Fisheries; SEAFO: Southeast Atlantic Fisheries Organization; SIOFA: South Indian Ocean Fisheries Agreement; SPRFMO: South Pacific Regional Fisheries Management Organization; WCPFC: Western and Central Pacific Fisheries Commission.

**One RMU (*C. mydas*, northeast Indian Ocean) was scored critical data needs only.

Among RFBs with more than one RMU, average risk scores ranged from 1.59 (RECOFI) to 2.12 (IATTC), while average fisheries bycatch scores ranged from 1.84 (WCPFC) to 3.00 (GFCM, NEAFC) (Table 6). High bycatch threat scores of RFBs generally agreed with global patterns of marine turtle bycatch, which highlighted the Mediterranean Sea, Northwest and Southwest Atlantic, and East Pacific Oceans as regions with particularly high bycatch threats to marine turtles [34].

As with global averages at other scales of assessment, average risk and threats (i.e. bycatch) scores for RFBs clustered around medium values (Tables 3, 5, and 6). This result across all scales of comparison (i.e. species, ocean basin, MTSG regions, RFBs) suggests that patterns in assessments of RMU risk and threats are not associated with particular species or regional scales. Thus, risk and threats scores for RMUs vary according to combinations of characteristics of the RMUs themselves (i.e. population status and trends, species-specific biology) as well as the environmental conditions and threats present within regions. These observations indicate that understanding biogeographical factors that influence the biology and ecology of marine turtle RMUs, as well as the anthropogenic pressures on those RMUs, will improve status assessments and inform conservation strategies to protect or recover RMUs [3].

Despite moderate average risk and threats scores within RFBs, the most prevalent category for RMUs was High Risk-High Threats within 11 of 18 RFBs (Table 6). Low Risk-High Threats was the most prevalent category in six RFBs (tied with High Risk-High Threats in NASCO), while High Risk-Low Threats was the most prevalent category in two RFBs (CCSBT, tied with High Risk-High Threats in WCPFC). One RFB's most prevalent category was Low Risk-Low Threats (NPFAC).

Three RFBs with the most and highest proportion of RMUs classified as critical data needs occurred in the Indian Ocean (IOTC, SIOFA, and RECOFI; Table 6). This corroborates results at other scales (Tables 3 and 5) because the same data-poor RMUs were being assessed in all analyses, despite different frames of reference. The third RFB with several critical data needs RMUs, WCPFC, also had the highest number of RMUs (n = 27; Table 6), and has the broadest Area of Competence of any RFB that is focused mainly in low and tropical latitudes (see Fig. S2). The sheer geographic extent and diversity of RMUs present, including those in Southeast Asia, which is generally data-poor (Table 5), probably contributed to this result.

As RFBs represent international entities with distinct mandates to effectively manage marine resources, the overlaps of RMUs with multiple RFBs demonstrate the extremely complex system of management responsibility for protected species like marine turtles in high-seas areas (e.g. [31], [32]). Nonetheless, this straightforward exercise provided information that can refine management approaches to reducing marine turtle bycatch in fisheries activities and help to prioritize broad-scale funding and conservation efforts, especially in situations where RMUs are high risk and under high bycatch threats (Table 6). Additional international management frameworks that would be good candidates for conservation status assessments of marine turtle RMUs would be the Inter-American Convention for the Protection and Conservation of Sea Turtles (IAC: http://www.iacseaturtle.org/), signatories to the Convention on the Conservation of Migratory Species of Wild Animals (CMS or the Bonn Convention: http://www.cms.int/), or FAO fishing areas (http://www.fao.org/fishery/area/search/en).

### Caveats and future improvements

An inherent challenge to assessments that use expert-opinion is dealing with incongruities among evaluators in terms of how criteria are scored. For example, species-specific or regional scoring patterns might reflect the non-uniform influence of certain experts or expertise [20]. Also, universal agreement about qualitative scoring scales is very difficult to achieve, especially for impacts of threats that are poorly known or studied. We tried to overcome these potential biases by including a diverse representation of MTSG Regional Vice-Chairs for broad geographic expertise, assessors of recent *Red List* assessments for species-specific expertise, as well as other MTSG members with ample overall expertise in marine turtle biology and conservation. Moreover, during the scoring process, we relied on information available in published literature—not only expert-opinion—to substantiate criteria scores and achieve consensus. Also, an extended comment period for the entire MTSG membership (*ca.* 230 people) allowed members to evaluate the system and results and to suggest improvements and changes.

Although the paired risk and threats scores provided overall assessments of conservation status for RMUs, an important discrepancy existed in terms of which life stages were assessed by the two different sets of criteria. Whereas the risk criteria were based on information from nesting colonies (i.e. nesting females only), threats criteria were evaluations of degree of impact posed by each threat to the entire population (i.e. multiple life-stages, including adult males and immature individuals). Although risk criteria like population size and trends are estimates based on nesting females only, these metrics are proxies for underlying population processes that include mortality patterns and other vital rates related to other life stages [17], which were considered in the threats criteria scores. Because this discrepancy is a common impediment to effective monitoring and conservation of marine turtle populations, abundance estimates and trends based on nesting females need to be accompanied by long-term mark-recapture studies to enable interpretation of observed trends and identification of drivers of population dynamics [17].

The preponderance of data deficient scores for pollution and pathogens and climate change presented a challenge in terms of calculating threats scores. While scores and data citations for both of these threats appear in the threats matrix (Dataset S2), these values were not included in overall threats scores for RMUs due to a lack of reliable information. This was a disadvantage to those RMUs where impacts of either or both of these threats are reasonably well-known (e.g. pollution and pathogens for *C. mydas*, North Central Pacific Ocean [Hawaii]: [35], [36]; climate change for *C. caretta*, Northwest Atlantic Ocean: [37]; *C. mydas*, Southwest Pacific Ocean; [38]). However, these findings provide clear support for enhancing efforts to quantify impacts to marine turtles of pollution and pathogens as well as climate change to improve our overall evaluation of threats (see [19] for review of global research priorities for marine turtles).

To partially counteract the above issues, this system, under the auspices of the MTSG, will rely on and allow for periodic updates to adjust scores and improve data reliability as new information becomes available. By listing all citations that were considered in scoring risk and threats criteria (see Datasets S1 and S2), we made the assessments themselves transparent, which will allow users to evaluate not only the scores but also the justifications for the scores, and to suggest changes or improvements. This user-driven evaluation system will facilitate collaboration within the MTSG and broader marine turtle conservation community, will make marine turtle status evaluations straightforward, and could provide a model for conservation assessments of other taxa that are also widely distributed and require regional conservation strategies (e.g. sharks: [4]; marine mammals: [7]).

Although this system evaluates risk and threats to marine turtles, conservation priority-setting frameworks should also include ecological, legal, and social information to balance technical, governance, and societal factors in decision-making [39]. In this light, future iterations of the marine turtle priority-setting framework and process presented here could incorporate ‘conservation capacity,’ or the suite of factors that exists in each RMU that influence the feasibility and efficacy of efforts to protect and recover marine turtle populations. A conservation capacity matrix might include the factors (e.g. degree of research conducted, socioeconomic issues), institutions (e.g. NGOs, government agencies), and legal frameworks (e.g. laws to protect marine turtles, protected areas, enforcement and implementation capacity) in place that can be evaluated in relation to the risk and threats criteria for each RMU to provide further information for setting conservation priorities.

### Conclusions

The conservation priorities portfolio framework allowed evaluation of risk and threats to marine turtles at various scales, and can provide guidance for research and conservation priorities of biogeographically defined RMUs. Because the complete matrices, including scores for risk, threats, and data uncertainty are available to users and fully cited, the specific criteria that drive risk or threat scores for RMUs can be identified and targeted for future research or conservation efforts at multiple relevant scales.

Another important feature of the portfolio system is that it reflects the reality that conservation priorities vary widely with objectives and values of different management entities, NGOs, researchers, funding bodies, and other stakeholders. By recognizing that conservation priorities can range from prevention of imminent extinctions to maintaining long-term monitoring projects, from preserving genetic diversity to managing fisheries more sustainably, this approach provides sufficient information to allow for numerous applications.

Nonetheless, assessing relative extinction risk is of particular importance to species-focused conservation at many geographic scales, and is a primary objective of many NGOs, government agencies, and international agreements and conventions. Our assessment priority-setting exercise produced a global list of the 11 marine turtle RMUs most threatened with extinction, which includes RMUs from four different species, all three major ocean basins, and from four different MTSG regions (Fig. 1; Table 2). The ‘Top 11 most endangered RMUs’ include well-documented cases of populations that have collapsed and are under high threat (e.g. *D. coriacea*, East Pacific Ocean; [40]–[42]), as well as other RMUs about which little is known (e.g. *C. caretta* and *E. imbricata*, Northeast Indian Ocean; [24]; *E. imbricata*, East Pacific Ocean; [43]). The RMUs on this list merit immediate attention, whether through reduction of threats, increased monitoring to more confidently assess risk and threats, or both.

The portfolio approach also permitted detection of RMUs that are priorities for continued or enhanced monitoring (i.e. Low Risk-Low Threats, critical data needs; Fig. 1). Specifically, large, stable or increasing, highly diverse populations under low to moderate threats were categorized as Low Risk-Low Threats, which places value on the ongoing census and conservation efforts directed toward these RMUs because these initiatives tend to generate valuable information about marine turtle biology, ecology, and population demography [17], [18], [44]. In addition to recognizing the importance of Low Risk-Low Threats, we also classified RMUs with high data uncertainty as critical data needs. Because we have relatively less confidence in the paired risk and threats scores for these RMUs, they are clear priorities for enhancing population monitoring and quantification of threats impacts to improve confidence in risk and threats assessments.

In terms of regional patterns, five of the 11 most endangered RMUs occurred within the Indian Ocean (Table 2), indicating generally high basin-wide risk and threats scores, and a high number of critical data needs RMUs (Tables 3, 5 and 6), making it the region of most conservation concern. Both bycatch and take are pervasive threats to marine turtles in the Indian Ocean, particularly in the northern areas [24], and long-term monitoring projects with effective conservation efforts are largely limited to the Southwest Indian Ocean [21], [23]. Ongoing collaborative efforts through the Indian Ocean-Southeast Asia Marine Turtle Memorandum of Understanding (IOSEA)—an inter-governmental agreement made under the auspices of the CMS—hold promise to address these issues in the region by integrating monitoring and recovery initiatives at national and regional scales, but much work remains to address the conservation issues facing marine turtles in this region.

Finally, the current criteria and evaluation framework we present here might offer an effective resource for the MTSG's need to balance its mandate to conduct timely assessment of marine turtle species using IUCN *Red List* criteria with widespread recognition of the inability of *Red List* criteria and process to adequately assess marine turtle extinction risk [5], [6]. While much work remains to align the portfolio framework criteria and process with the *Red List* criteria and assessment process, this approach holds great potential to address a fundamental challenge for the MTSG and to establish a system for future conservation status assessments of marine turtle RMUs and species.

Conservation status assessments and subsequent priority-setting require the best available information for the species or populations being evaluated. The system we have developed is robust and flexible, and can be improved and refined with continuous user input. Taken together, the RMU framework [3] and conservation portfolio system described here provide a significant advance for status evaluations and conservation priority-setting for widely distributed, long-lived taxa.

## Supporting Information

Figure S1 **Paired risk and threats scores for RMUs of each marine turtle species.** (A) loggerheads (*Caretta caretta*), (B) green turtles (*Chelonia mydas*), (C) leatherbacks (*Dermochelys coriacea*, (D) hawskbills (*Eretmochelys imbricata*), (E) olive ridleys (*Lepidochelys olivacea*), (F) Kemp's ridleys (*Lepidochelys kempii*) and flatbacks (*Natator depressus*). Vertical and horizontal bars associated with each paired score represent the data uncertainty index; see text for details. RMUs in red denote critical data needs, i.e. data uncertainty indices for both risk and threats ≥1.
(TIF)

Figure S2 **Areas of Competence for Regional Fishery Bodies (RFB) with a management mandate.** RFB acronyms: CCAMLR: Commission on the Conservation of Antarctic Marine Living Resources; CCBSP: Convention on the Conservation and Management of Pollock Resources in the Central Bering Sea; CCSBT: Commission for the Conservation of Southern Bluefin Tuna; GFCM: General Fisheries Commission for the Mediterranean; IATTC: Inter-American Tropical Tuna Commission; ICCAT: International Commission for the Conservation of Atlantic Tunas; IOTC: Indian Ocean Tuna Commission; IPHC: International Pacific Halibut Commission; NAFO: Northwest Atlantic Fisheries Organization; NASCO: North Atlantic Salmon Conservation Organization; NEAFC: Northeast Atlantic Fisheries Commission; NPFAC: North Pacific Anadromous Fish Commission; PSC: Pacific Salmon Commission; RECOFI: Regional Commission for Fisheries; SEAFO: Southeast Atlantic Fisheries Organization; SIOFA: South Indian Ocean Fisheries Agreement; SPRFMO: South Pacific Regional Fisheries Management Organization; WCPFC: Western and Central Pacific Fisheries Commission. See FAO fact sheets for RFBs at http://www.fao.org/fishery/rfb/search/en.
(TIF)

Table S1 Scoring system for population size criterion in risk matrix. Numbers are average annual nesting females for the most recent survey data available.
(DOCX)

Table S2 List of Regional Management Unit (RMU) codes used in Fig. 1. Species: *Caretta caretta*, loggerhead; *Chelonia mydas*, green turtle; *Dermochelys coriacea*, leatherback; *Eretmochelys imbricata*, hawksbill; *Lepidochelys kempii*, Kemp's ridley; *Lepidochelys olivacea*, olive ridley; *Natator depressus*, flatback.
(DOCX)

Table S3 Categories in which RMUs for each species occurred (including critical data needs RMUs). Categories: HR-HT = High risk-High threats; HR-LT = High risk-Low threats; LR-LT = Low risk-Low threats; LR-HT = Low risk-High threats.
(DOCX)

Dataset S1 **Bibliography of literature used to score risk and threats criteria.**
(PDF)

Dataset S2 **Complete dataset used to score all criteria in Risk and Threats matrices, as well as data uncertainty scores. <Wallace_etal_MTSG_priority-setting_S3.xlsx>**
(XLS)

Dataset S3 **Complete list of SWOT – The State of the World's Sea Turtles data providers. <Wallace_etal_PLoSONE_DatasetS3.xls>**
(XLS)

## References

[1] Fowler SL, Cavanagh RD, Camhi M, Burgess GH, Cailliet GM (2005). Sharks, Rays and Chimaeras: The Status of the Chondrichthyan Fishes. Status Survey. IUCN/SSC Shark Specialist Group.

[2] Boyd C, Brooks TM, Butchart SHM, Edgar GJ, da Fonseca GAB (2008). Spatial scale and the conservation of threatened species.. Conservation Letters.

[3] Wallace BP, DiMatteo AD, Hurley BJ, Finkbeiner EM, Bolten AB (2010a). Regional Management Units for marine turtles: A novel framework for prioritizing conservation and research across multiple scales.. PLoS ONE.

[4] Fowler SL, Cavanagh RD, Fowler SL, Cavanagh RD, Camhi M, Burgess GH, Cailliet GM, Fordham SV, Simpfendorfer CA, Musick JA (2005). Species status reports.. Sharks, Rays and Chimaeras: The Status of the Chondrichthyan Fishes. Status Survey. IUCN/SSC Shark Specialist Group.

[5] Seminoff J, Shanker K (2008). Marine turtles and IUCN Red Listing: A review of the process, the pitfalls, and novel assessment approaches.. Journal of Experimental Marine Biology and Ecology.

[6] Godfrey MH, Godley BJ (2008). Seeing past the red: flawed IUCN global listings for sea turtles.. Endangered Species Research.

[7] Freeman MR (2008). Challenges of assessing cetacean population recovery and conservation status.. Endangered Species Research.

[8] IUCN (2010). IUCN Red List of threatened Species. Version 2010.1.. http://www.iucnredlist.org.

[9] Mast RB, Hutchinson BJ, Howgate E, Pilcher NJ (2005). MTSG update: IUCN/SSC Marine Turtle Specialist Group hosts the second Burning Issues Assessment Workshop.. Marine Turtle Newsletter.

[10] Wallace BP, Saba VS (2009). Environmental and anthropogenic impacts on intra-specific variation in leatherback turtles: opportunities for targeted research and conservation.. Endangered Species Research.

[11] Meylan AB, Donnelly M (1999). Status justification for listing the hawksbill turtle (*Eretmochelys imbricata*) as Critically Endangered on the 1996 IUCN Red List of Threatened Animals.. Chelonian Conservation and Biology.

[12] Seminoff JA (2004). Global Status Assessment: Green turtle (*Chelonia mydas*)..

[13] Abreu-Grobois FA, Plotkin PT (2007). IUCN Red List Status Assessment of the olive ridley sea turtle (*Lepidochelys olivacea*).

[14] Mortimer JA, Donnelly M (2008). Turtle Specialist Group 2007 IUCN Red List Status Assessment Hawksbill Turtle (*Eretmochelys imbricata*), 121 pages.. http://www.iucnredlist.org/documents/attach/8005.pdf.

[15] Mast RB, Wallace B, Hutchinson BJ, Chaloupka M, Bolten AB (2009). IUCN-SSC Marine Turtle Specialist Group Quarterly Report: Progress from the Fifth Burning Issues Workshop (BI-5).. Marine Turtle Newsletter.

[16] Merkhofer M (1987). Decision science and social risk management: comparative evaluation of cost-benefit analysis, decision analysis and other formal decision-aiding approaches.

[17] National Research Council (NRC) (2010). Assessment of Sea-Turtle Status and Trends: Integrating Demography and Abundance.

[18] Chaloupka M, Bjorndal KA, Balazs GH, Bolten AB, Ehrhart LM (2008). Encouraging outlook for recovery of a once severely exploited marine megaherbivore.. Global Ecology and Biogeography.

[19] Hamann M, Godfrey MH, Seminoff JA, Arthur K, Barata PCR (2010). Global research priorities for sea turtles: informing management and conservation in the 21st century.. Endangered Species Research.

[20] Donlon CJ, Wingfield DK, Crowder LB, Wilcox C (2010). Using expert opinion surveys to rank threats to endangered species: A case study with sea turtles.. Conservation Biology.

[21] Laurent-Stepler M, Bourjea J, Roos D, Pelletier D, Ryan P (2007). Reproductive seasonality and trend of Chelonia mydas in the SW Indian Ocean: a 20 year study based on track counts.. Endangered Species Research.

[22] Turtle Expert Working Group (2007). An assessment of the Leatherback Turtle Population in the Atlantic Ocean..

[23] Nel R (2008).

[24] Shanker K, Choudhury BC (2006). Marine Turtles of The Indian Subcontinent.

[25] Bolten AB, Bolten AB, Witherington B (2003). Active Swimmers - Passive Drifters: The Oceanic Juvenile Stage of Loggerheads in the Atlantic System.. Loggerhead Sea Turtles.

[26] Bolker BM, Okuyama T, Bjorndal KA, Bolten AB (2007). Incorporating multiple mixed stocks in mixed stock analysis: ‘many-to-many’ analyses.. Mol Ecol.

[27] Monzón-Argüello C, López-Jurado L, Rico C, Marco A, López P (2010). Evidence from gnetic and Lagrangian drifter data for transatlantic transport of small juvenile green turtles.. J Biogeogr.

[28] Margaritoulis D, Argano R, Baran I, Bentivegna F, Bradai MN, Bolten AB, Witherington BE (2003). Loggerhead turtles in the Mediterranean Sea: Present knowledge and conservation perspectives.. Loggerhead Sea Turtles.

[29] Ehrhart LE, Bagley DA, Redfoot WE, Bolten A, Witherington B (2003). Loggerhead turtles in the Atlantic Ocean: Geographic distribution, abundance, and population status.. Loggerhead Sea Turtles.

[30] Casale P, Margaritoulis D (2010). Sea turtles in the Mediterranean: Distribution, threats and conservation priorities.

[31] Shillinger GL, Palacios DM, Bailey H, Bograd SJ, Swithenbank AM (2008). Persistent Leatherback Turtle Migrations Present Opportunities for Conservation.. PLoS Biology.

[32] Witt MJ, Bonguno EA, Broderick AC, Coyne MS, Formia A (2011). Tracking leatherback turtles from the world's largest rookery: assessing threats across the South Atlantic.. Proceedings of the Royal Society B.

[33] Crowder LB, Norse E (2008). Essential ecological insights for marine ecosystem-based management and marine spatial planning.. Marine Policy.

[34] Wallace BP, Lewison R, McDonald S, McDonald R, Kot C (2010b). Global patterns of marine turtle bycatch.. Conservation Letters.

[35] Chaloupka M, Work TM, Balazs GH, Murakawa SKK, Morris R (2008b). Cause-specific temporal and spatial trends in green sea turtle strandings in the Hawaiian Archipelago (1982–2003).. Marine Biology.

[36] Van Houtan KS, Hargrove SK, Balazs GH (2010). Land use, macroalgae, and a tumor-forming disease in marine turtles.. PLoS ONE.

[37] Hawkes LA, Broderick AC, Godfrey MH, Godley BJ (2007). Investigating the potential impacts of climate change on a marine turtle population.. Global Change Biology.

[38] Fuentes MMPB, Limpus CJ, Hamann M (2010). Vulnerability of sea turtle nesting grounds to climate change.. Global Change Biology.

[39] Marsh H, Dennis A, Hines H, Kutt A, McDonald K (2006). Optimizing allocation of management resources for wildlife.. Conservation Biology.

[40] Spotila JR, Reina RD, Steyermark AC, Plotkin PT, Paladino FV (2000). Pacific leatherback turtles face extinction.. Nature.

[41] Santidrián Tomillo P, Veléz E, Reina RD, Piedra R, Paladino FV (2007). Reassessment of the leatherback turtle (*Dermochelys coriacea*) nesting population at Parque Nacional Marino Las Baulas, Costa Rica: effects of conservation efforts.. Chelonian Conservation and Biology.

[42] Sarti Martínez L, Barragán AR, Muñoz DG, García N, Huerta P (2007). Conservation and biology of the leatherback turtle in the Mexican Pacific.. Chelonian Conservation and Biology.

[43] Gaos AR, Abreu-Grobois FA, Alfaro-Shigueto J, Amorocho D, Arauz R (2010). Signs of hope in the eastern Pacific: International collaboration reveals encouraging status for the severely depleted population of hawksbill turtles.. Oryx.

[44] Limpus CJ, Miller JD, Parmenter CJ, Limpus DJ (2003). The green turtle, *Chelonia mydas*, population of Raine Island and the Northern Great Barrier Reef: 1843–2001.. Memoirs Queensland Museum.

