# Supplementary figures and images for: Global Conservation Priorities for Marine Turtles
Source: PLoS One. 2011 Sep 28;6(9):e24510. doi: 10.1371/journal.pone.0024510 (PMC3182175; doi:10.1371/journal.pone.0024510)

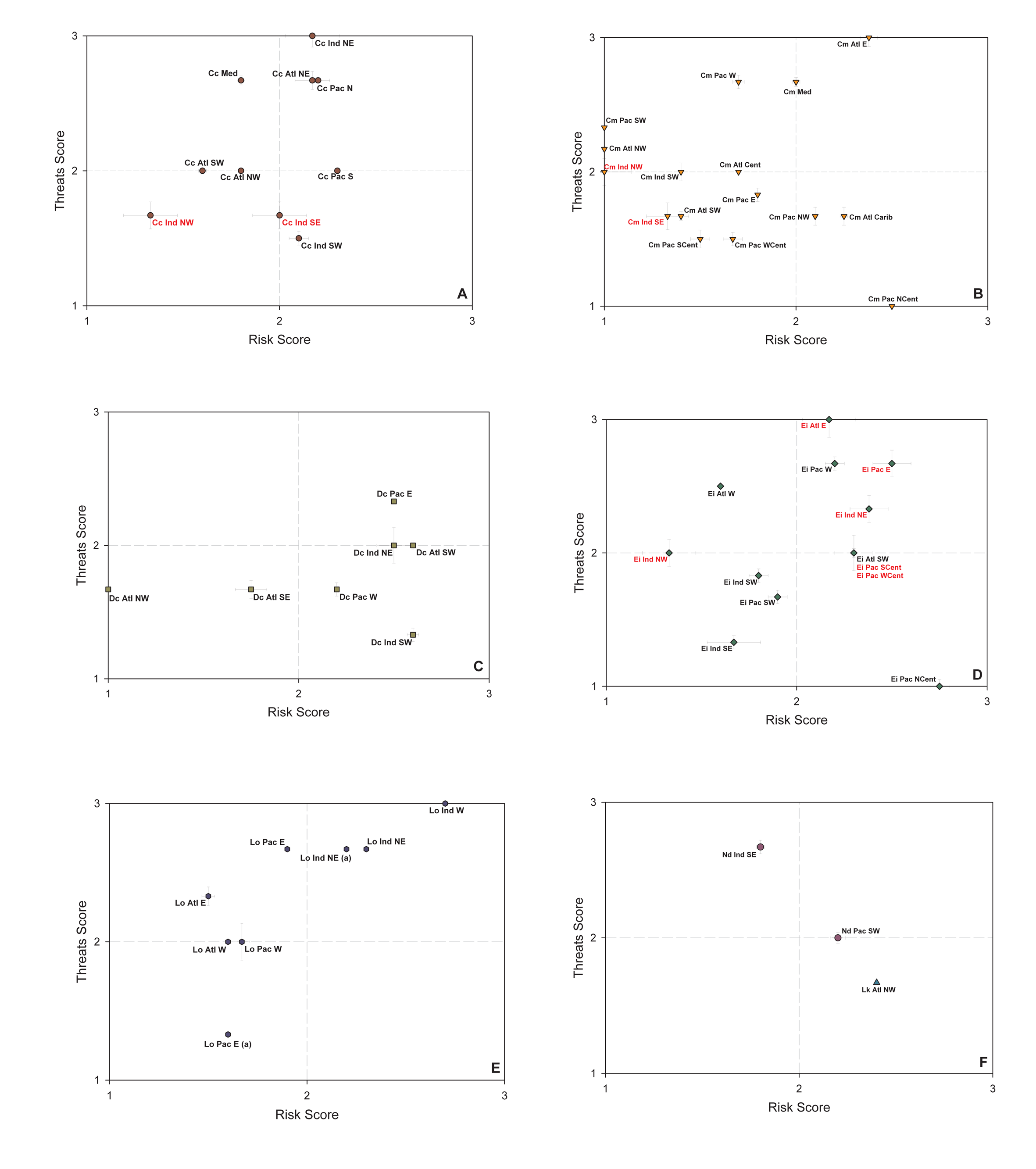

Supplement: Figure S1 — Paired risk and threats scores for RMUs of each marine turtle species. (A) loggerheads (Caretta caretta), (B) green turtles (Chelonia mydas), (C) leatherbacks (Dermochelys coriacea, (D) hawskbills (Eretmochelys imbricata), (E) olive ridleys (Lepidochelys olivacea), (F) Kemp's ridleys (Lepidochelys kempii) and flatbacks (Natator depressus). Vertical and horizontal bars associated with each paired score represent the data uncertainty index; see text for details. RMUs in red denote critical data needs, i.e. data uncertainty indices for both risk and threats ≥1. (TIF) [file pone.0024510.s001.tif]

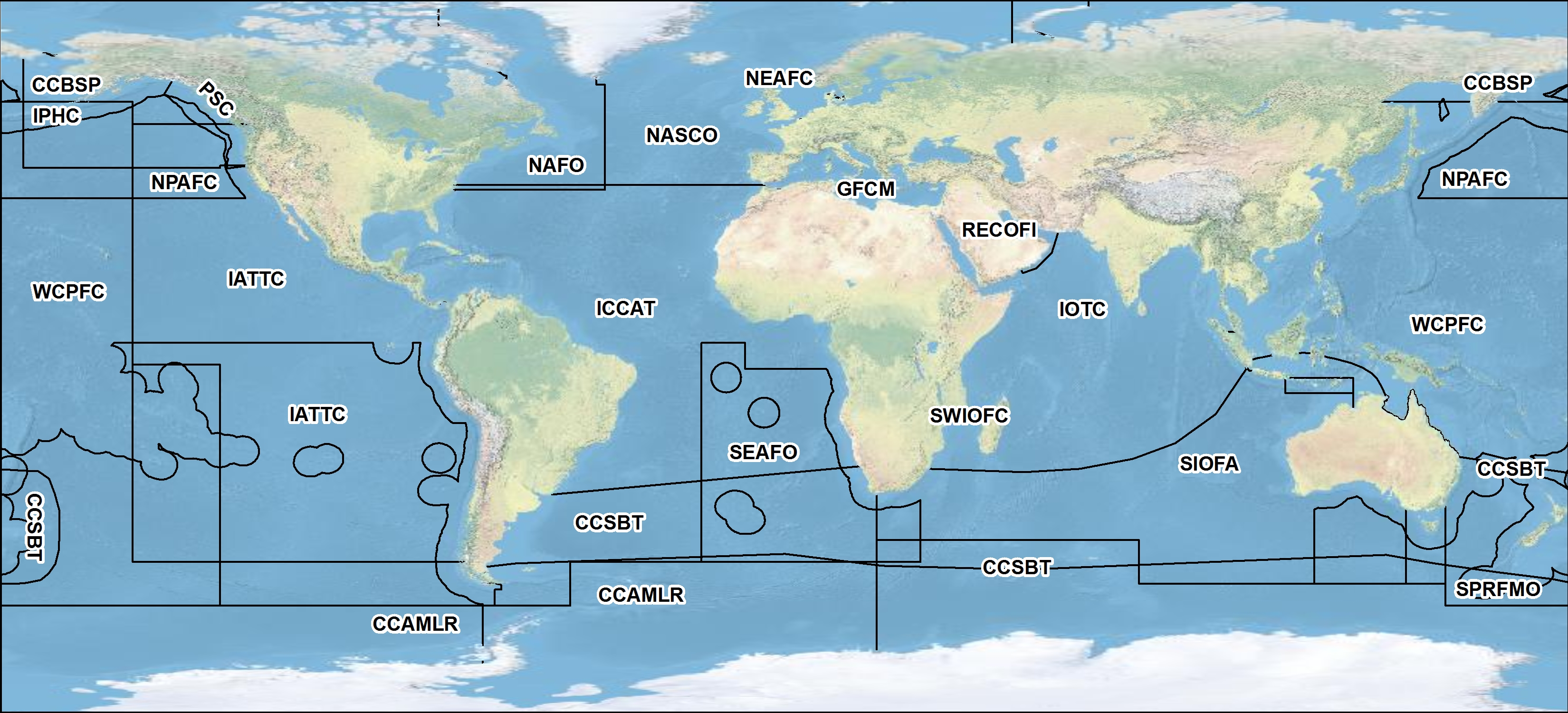

Supplement: Figure S2 — Areas of Competence for Regional Fishery Bodies (RFB) with a management mandate. RFB acronyms: CCAMLR: Commission on the Conservation of Antarctic Marine Living Resources; CCBSP: Convention on the Conservation and Management of Pollock Resources in the Central Bering Sea; CCSBT: Commission for the Conservation of Southern Bluefin Tuna; GFCM: General Fisheries Commission for the Mediterranean; IATTC: Inter-American Tropical Tuna Commission; ICCAT: International Commission for the Conservation of Atlantic Tunas; IOTC: Indian Ocean Tuna Commission; IPHC: International Pacific Halibut Commission; NAFO: Northwest Atlantic Fisheries Organization; NASCO: North Atlantic Salmon Conservation Organization; NEAFC: Northeast Atlantic Fisheries Commission; NPFAC: North Pacific Anadromous Fish Commission; PSC: Pacific Salmon Commission; RECOFI: Regional Commission for Fisheries; SEAFO: Southeast Atlantic Fisheries Organization; SIOFA: South Indian Ocean Fisheries Agreement; SPRFMO: South Pacific Regional Fisheries Management Organization; WCPFC: Western and Central Pacific Fisheries Commission. See FAO fact sheets for RFBs at http://www.fao.org/fishery/rfb/search/en. (TIF) [file pone.0024510.s002.tif]
