# Supplementary material for: Global Conservation Priorities for Marine Turtles
Source: PLoS One. 2011 Sep 28;6(9):e24510. doi: 10.1371/journal.pone.0024510 (PMC3182175; doi:10.1371/journal.pone.0024510)
Supplement: Table S1 — Scoring system for population size criterion in risk matrix. Numbers are average annual nesting females for the most recent survey data available. (DOCX) [file pone.0024510.s003.docx]

**Global Conservation Priorities for Marine Turtles**

Bryan P. Wallace et al.

[b.wallace@conservation.org](mailto:b.wallace@conservation.org)

**Supplemental Information**

**Table S1.** Scoring system for population size criterion in risk matrix. Numbers are average annual nesting females for the most recent survey data available.

| **Species** | **3** | **2.5** | **2** | **1.5** | **1** |
| --- | --- | --- | --- | --- | --- |
| *C. caretta* | <100 | 101-1,000 | 1,001-5,000 | 5,001-10,000 | >10,000 |
| *C. mydas* | <100 | 101-1,000 | 1,001-5,000 | 5,001-10,000 | >10,000 |
| *E. imbricata* | <100 | 101-1,000 | 1,001-5,000 | 5,001-10,000 | >10,000 |
| *D. coriacea* | <100 | 101-1,000 | 1,001-5,000 | 5,001-10,000 | >10,000 |
| *L. olivacea* | <100 | 101-1,000 | 1,001-10,000 | 10,001-100,000 | >100,000 |
| *L. kempii* | <100 | 101-1,000 | 1,001-10,000 | 10,001-100,000 | >100,000 |
| *N. depressus* | <100 |  | 101-1,000 |  | >1,000 |
