# Supplementary material for: Global Conservation Priorities for Marine Turtles
Source: PLoS One. 2011 Sep 28;6(9):e24510. doi: 10.1371/journal.pone.0024510 (PMC3182175; doi:10.1371/journal.pone.0024510)
Supplement: Table S2 — List of Regional Management Unit (RMU) codes used in Fig. 1. Species: Caretta caretta, loggerhead; Chelonia mydas, green turtle; Dermochelys coriacea, leatherback; Eretmochelys imbricata, hawksbill; Lepidochelys kempii, Kemp's ridley; Lepidochelys olivacea, olive ridley; Natator depressus, flatback. (DOCX) [file pone.0024510.s004.docx]

**Global Conservation Priorities for Marine Turtles**

Bryan P. Wallace et al.

[b.wallace@conservation.org](mailto:b.wallace@conservation.org)

**Supplemental Information**

**Table S2. List of Regional Management Unit (RMU) codes used in Fig. 1.** Species: *Caretta caretta*, loggerhead; *Chelonia mydas*, green turtle; *Dermochelys coriacea*, leatherback; *Eretmochelys imbricata*, hawksbill; *Lepidochelys kempii*, Kemp’s ridley; *Lepidochelys olivacea*, olive ridley; *Natator depressus*, flatback.

| **RMU code** | **RMU name** | **Species** | **RMU ocean basin** | **RMU primary region** | **RMU secondary region** |
| --- | --- | --- | --- | --- | --- |
| Cc1 | Cc Atl NE | *Caretta caretta* | Atlantic | Northeast | Cape Verde |
| Cc2 | Cc Atl NW | *Caretta caretta* | Atlantic | Northwest |  |
| Cc3 | Cc Atl SW | *Caretta caretta* | Atlantic | Southwest |  |
| Cc4 | Cc Ind NE | *Caretta caretta* | Indian | Northeast |  |
| Cc5 | Cc Ind NW | *Caretta caretta* | Indian | Northwest |  |
| Cc6 | Cc Ind SE | *Caretta caretta* | Indian | Southeast |  |
| Cc7 | Cc Ind SW | *Caretta caretta* | Indian | Southwest |  |
| Cc8 | Cc Med | *Caretta caretta* | Mediterranean | Mediterranean |  |
| Cc9 | Cc Pac N | *Caretta caretta* | Pacific | North |  |
| Cc10 | Cc Pac S | *Caretta caretta* | Pacific | South |  |
| Cm1 | Cm Atl E | *Chelonia mydas* | Atlantic | East |  |
| Cm2 | Cm Atl NW | *Chelonia mydas* | Atlantic | Northwest |  |
| Cm3 | Cm Atl Carib | *Chelonia mydas* | Atlantic | South Caribbean |  |
| Cm4 | Cm Atl Cent | *Chelonia mydas* | Atlantic | Central | Ascension |
| Cm5 | Cm Atl SW | *Chelonia mydas* | Atlantic | Southwest |  |
| Cm6 | Cm Ind NE | *Chelonia mydas* | Indian | Northeast |  |
| Cm7 | Cm Ind NW | *Chelonia mydas* | Indian | Northwest |  |
| Cm8 | Cm Ind SE | *Chelonia mydas* | Indian | Southeast |  |
| Cm9 | Cm Ind SW | *Chelonia mydas* | Indian | Southwest |  |
| Cm10 | Cm Med | *Chelonia mydas* | Mediterranean | Mediterranean |  |
| Cm11 | Cm Pac E | *Chelonia mydas* | Pacific | East |  |
| Cm12 | Cm Pac NCent | *Chelonia mydas* | Pacific | North Central | Hawaii |
| Cm13 | Cm Pac NW | *Chelonia mydas* | Pacific | Northwest |  |
| Cm14 | Cm Pac SCent | *Chelonia mydas* | Pacific | South Central |  |
| Cm15 | Cm Pac SW | *Chelonia mydas* | Pacific | Southwest |  |
| Cm16 | Cm Pac WCent | *Chelonia mydas* | Pacific | West Central | Palau, Micronesia, Marianas |
| Cm17 | Cm Pac W | *Chelonia mydas* | Pacific | West | West Pacific / SE Asia |
| Dc1 | Dc Atl NW | *Dermochelys coriacea* | Atlantic | Northwest |  |
| Dc2 | Dc Atl SE | *Dermochelys coriacea* | Atlantic | Southeast |  |
| Dc3 | Dc Atl SW | *Dermochelys coriacea* | Atlantic | Southwest |  |
| Dc4 | Dc Ind NE | *Dermochelys coriacea* | Indian | Northeast |  |
| Dc5 | Dc Ind SW | *Dermochelys coriacea* | Indian | Southwest |  |
| Dc6 | Dc Pac E | *Dermochelys coriacea* | Pacific | East |  |
| Dc7 | Dc Pac E | *Dermochelys coriacea* | Pacific | West |  |
| Ei1 | Ei Atl E | *Eretmochelys imbricata* | Atlantic | East |  |
| Ei2 | Ei Atl SW | *Eretmochelys imbricata* | Atlantic | Southwest |  |
| Ei3 | Ei Atl W | *Eretmochelys imbricata* | Atlantic | West | Western Caribbean / USA |
| Ei4 | Ei Ind NE | *Eretmochelys imbricata* | Indian | Northeast |  |
| Ei5 | Ei Ind NW | *Eretmochelys imbricata* | Indian | Northwest |  |
| Ei6 | Ei Ind SE | *Eretmochelys imbricata* | Indian | Southeast |  |
| Ei7 | Ei Ind SW | *Eretmochelys imbricata* | Indian | Southwest |  |
| Ei8 | Ei Pac E | *Eretmochelys imbricata* | Pacific | East |  |
| Ei9 | Ei Pac NCent | *Eretmochelys imbricata* | Pacific | North Central | Hawaii |
| Ei10 | Ei Pac SCent | *Eretmochelys imbricata* | Pacific | South Central |  |
| Ei11 | Ei Pac SW | *Eretmochelys imbricata* | Pacific | Southwest |  |
| Ei12 | Ei Pac WCent | *Eretmochelys imbricata* | Pacific | West Central | Palau, Micronesia, Marianas |
| Ei13 | Ei Pac W | *Eretmochelys imbricata* | Pacific | West | Indonesia (both coasts), Malaysia, Coral Triangle |
| Lk1 | Lk Atl NW | *Lepidochelys kempii* | Atlantic | Northwest |  |
| Lo1 | Lo Atl E | *Lepidochelys olivacea* | Atlantic | East |  |
| Lo2 | Lo Atl W | *Lepidochelys olivacea* | Atlantic | West |  |
| Lo3 | Lo Ind NE | *Lepidochelys olivacea* | Indian | Northeast |  |
| Lo4 | Lo Ind NE (a) | *Lepidochelys olivacea* | Indian | Northeast (arribadas) |  |
| Lo5 | Lo Ind W | *Lepidochelys olivacea* | Indian | West |  |
| Lo6 | Lo Pac E | *Lepidochelys olivacea* | Pacific | East |  |
| Lo7 | Lo Pac E (a) | *Lepidochelys olivacea* | Pacific | East (arribadas) |  |
| Lo8 | Lo Pac W | *Lepidochelys olivacea* | Pacific | West |  |
| Nd1 | Nd Ind SE | *Natator depressus* | Indian | Southeast | Northern Australia |
| Nd2 | Nd Pac SW | *Natator depressus* | Pacific | Southwest | Eastern Australia |
