# Supplementary material for: Global Conservation Priorities for Marine Turtles
Source: PLoS One. 2011 Sep 28;6(9):e24510. doi: 10.1371/journal.pone.0024510 (PMC3182175; doi:10.1371/journal.pone.0024510)
Supplement: Table S3 — Categories in which RMUs for each species occurred (including critical data needs RMUs). Categories: HR-HT = High risk-High threats; HR-LT = High risk-Low threats; LR-LT = Low risk-Low threats; LR-HT = Low risk-High threats. (DOCX) [file pone.0024510.s005.docx]

**Global Conservation Priorities for Marine Turtles**

Bryan P. Wallace et al.

[b.wallace@conservation.org](mailto:b.wallace@conservation.org)

**Supplemental Information**

**Table S3.** Categories in which RMUs for each species occurred (including critical data needs RMUs; see methods for details). Categories: HR-HT=High risk-High threats; HR-LT=High risk-Low threats; LR-LT=Low risk-Low threats; LR-HT=Low risk-High threats.

|  |  | **Categories** | | | |  |
| --- | --- | --- | --- | --- | --- | --- |
| **Species** | **critical data needs** | **HR-HT** | **HR-LT** | **LR-LT** | **LR-HT** | **Total*** |
| ***C. caretta* (n=10)** | 2 | 4 | 2 | 1 | 3 | 10 |
| ***C. mydas* (n=17) *** | 4 | 1 | 3 | 5 | 7 | 16 |
| ***D. coriacea* (n=7)** | 0 | 3 | 2 | 2 | 0 | 7 |
| ***E. imbricata* (n=13)** | 6 | 7 | 1 | 3 | 2 | 13 |
| ***L. kempii* (n=1)** | 0 | 0 | 1 | 0 | 0 | 1 |
| ***L. olivacea* (n=8)** | 0 | 3 | 0 | 1 | 4 | 8 |
| ***N. depressus* (n=2)** | 0 | 1 | 0 | 0 | 1 | 2 |
| **Total** | **12** | **21** | **9** | **12** | **15** | **57*** |
|  |  |  |  |  |  |  |
| * one RMU (*C. mydas*, northeast Indian Ocean) scored as critical data needs only | | | | | | |
