# Supplementary material for: Global Conservation Priorities for Marine Turtles
Source: PLoS One. 2011 Sep 28;6(9):e24510. doi: 10.1371/journal.pone.0024510 (PMC3182175; doi:10.1371/journal.pone.0024510)
Supplement: Dataset S1 — Bibliography of literature used to score risk and threats criteria. (PDF) [file pone.0024510.s006.pdf]

Global conservation priorities for marine turtles

Bryan P. Wallace et al.

Supporting Information Dataset S1. Bibliography of literature used to score risk and threats criteria.

(1992) First Kemp's ridley nesting in South Carolina. Marine Turtle Newsletter 59:23
(1993) Satellites used to Study the Oceanic Migrations of Hawaii's Green Sea Turtles. Marine Turtle Newsletter 61:7-9
(1994) Long-Distance Migration of Green Sea Turtles from Pulau Redang Tracked by Satellites. Marine Turtle Newsletter 66:5-7
(1994) Kemp's Ridley Nests in Florida. Marine Turtle Newsletter 67:16
(1995) Sea Turtles at Dungsha Tao, South China Sea. Marine Turtle Newsletter 70:13-14
(1995) Sea turtle recovery action plans. Marine Turtle Newsletter 69:22
(1997) Sea Turtle Conservation in Guatemala. Marine Turtle Newsletter 77:15-17
(1998) Preliminary observations on green turtles, *Chelonia mydas*, in foraging pastures of the United Arab Emirates. Marine Turtle Newsletter 79:8-9
(1998) Turtle voyages from Pakistan to Africa. Marine Turtle Newsletter 79:19
(1999a) Tartaruga gigante na Ilha surpreende biólogos. Diário Catarinense, Florianópolis, 3 January 1999. In Barata, P.C.R. & F.F.C. Fabiano. 2002. Evidence for Leatherback Sea Turtle (Dermochelys coriacea) Nesting in Arraial do Cabo, State of Rio de Janeiro, and a Review of Occasional Leatherback Nests in Brazil. Marine Turtle Newsletter 96: 13-16.
(1999b) Tartaruga desova na praia do Campeche. O Estado, Florianópolis, 18 January 1999. In Barata, P.C.R. & F.F.C. Fabiano. 2002. Evidence for Leatherback Sea Turtle (Dermochelys coriacea) Nesting in Arraial do Cabo, State of Rio de Janeiro, and a Review of Occasional Leatherback Nests in Brazil. Marine Turtle Newsletter 96: 13-16.
(2004) Laporan Tahunan TN Alas Purwo. Annual Report of Alas Purwo National Park Banyuwangi, East Java, Indonesia: Alas Purwo National Park Office, Department of Forestry
(2006) Hawaii: A Center for Pacific Sea Turtle Research & Conservation. Western Pacific Regional Fishery Management Council. Special publication
(2006) AMP Isole Pelagie. Available online *at*: http://www.isole-pelagie.it/file/archivio/news/2006.html.
(2006,2007) Turtle track Sri Lanka. Marine Conservation Society. *Online at:* http://www.mcsuk.org/marineworld/trackturtle/how+turtle+tracking+works
(2007 (January)) RP giant turtle migrates to Vietnam. Philippine Star, Manilla
'de Luque Fernandez AC, Lopez Barrera EA, Rosada Leon AC, Vera Jimenez NI (2003) Determinación de las condiciones de anidamiento en las playas focales del sector de arrecifes (PNNT) e implementación de sistemas de incubación para huevos de tortugas marinas. Seminario de Investigación (Proyecto II), Universidad Jorge Tadeo Lozano, Facultad de Biología Marina Santa Marta, Colombia
Abella-Guitérrez I, López-Conlon M (2006) Informe de la Anidación de Tortugas Marinas en la Reserva Pacuare durante la temporada 2005. Reserva Pacuare, Limón, Costa Rica. In: Mast RB, Bailey LM, Hutchinson BH (eds) SWoT Report—The State of the World's Sea Turtles, Volume II, Washington, DC. 49p. Available online *at*: http://seaturtlestatus.org/report/view
Abreu-Grobois FA, Pers. Comm. (2009) Locations of nesting Olives ridley, *Lepidochelys olivacea*, nests along the coast of Mexico and Central America
Abreu-Grobois FA, LeRoux RA (2007-2008) Hawksbill genetics explained. In: Mast RB, Bailey LM, Hutchinson BH (eds) SWoT Report—The State of the World's Sea Turtles, Volume III, Vol Available online *at*: http://seaturtlestatus.org/report/view, Washington, DC. 43p. Available online *at*: http://seaturtlestatus.org/report/view, p 16
Abreu-Grobois FA, Plotkin PT (2007) IUCN Red List Status Assessment of the olive ridley sea turtle (*Lepidochelys olivacea*). IUCN/SSC-Marine Turtle Specialist Group, 39 pp

Addison D (2007) Personal communication. Loggerhead nesting in Cay Sal Bank, Bahamas. In: Mast RB, Bailey LM, Hutchinson BH (eds) SWoT Report—The State of the World's Sea Turtles, Volume II, Washington, DC. 49p. Available online *at*: http://seaturtlestatus.org/report/view

Adjei R, Boakye G, Adu S (2001) Organisational profile: Ghana Wildlife Society. Marine Turtle Newsletter 93

Adnyana W, Soede LP, Gearheart G, Halim M (2008) Status of green turtle (*Chelonia mydas*) nesting and foraging populations of Berau, East Kalimantan, Indonesia, including results from tagging and telemetry. Indian Ocean Turtle Newsletter 7:2-11

Aggarwal RK, Lalremruata A, Velavan TP, Sowjanya AP, Singh L (2008) Development and characterization of ten novel microsatellite markers from olive ridley sea turtle (*Lepidochelys olivacea*). Conservation Genetics:981-984

Aguirre AA, Balazs GH, Murakawa SKK, Spraker TR (1998) Oropharyngeal fibropapillomas in Hawaiian green turtles (Chelonia mydas): pathological and epidemiologic perspectives. In: Epperly S, Braun J (eds) Proceedings of the seventeenth annual sea turtle symposium. U.S. Dep. Commer. NOAA Tech. Memo. NMFS-SEFSC-415, Orlando, Florida. 342 p.

Ahamada S (2004) Personal Communication. Cited in Seminoff, J.A., (assessor). 2004. Global Status Assessment: Green turtle (Chelonia mydas). Marine Turtle Specialist Group. Species Survival Commission, Red List Programme: 71.

Akesson S, Broderick AC, Glen F, Godley AC, Luschi P, Papi F, Hays G (2003) Navigation by green turtles: which strategy do displaced adults use to find Ascension Island? Oikos 103:363-372

Al Ansi M, Pilcher NJ (2007) Development and Implamentation of the Conservation and Monitoring Project for Marine Turtles, Ras Laffan Industrial City, Qatar. 78 p

Al-Merghani M, Miller JD, Pilcher NJ, Al-Mansi A (2000) The green and hawksbill turtles in the Kingdom of Saudi Arabia: Synopsis of nesting studies 1986-1997. Fauna of Arabia 18:369-384

Al-Merhgani (2000)

Al-Saady (1997) Pers. comm.

Alava JJ, Pritchard P, Wyneken J, Valverde H (2007) First Documented Record of Nesting by the Olive Ridley Turtle (*Lepidochelys olivacea*) in Ecuador. Chelonian Conservation and Biology 6:282-285

Alexander J, Garret K, Conrad J, Coles W (2004) Tagging and Nesting Research on Leatherback Sea Turtles (*Dermochelys coriacea*) on Sandy Point, St. Croix, U.S. Virgin Islands. Annual Report to Fish and Wildlife Service

Alfaro-Shigueto J, Dutton DH, Mangel J, Vega D (2004) First confirmed occurrence of loggerhead turtles *Caretta caretta* in Peru. Marine Turtle Newsletter 103:7-11

Alfaro-Shigueto J, Dutton PH, Bressem M-foV, Mangel J (2007) Interactions Between Leatherback Turtles and Peruvian Artisanal Fisheries. Chelonian Conservation and Biology 6:129-134

AlKindi A, Mahmoud I, Al-Gheilani H, Al-Bahry S, Bakheit CS (2002) Disorientation of the green turtle, Chelonia mydas, during nesting exercise relative to some physical and human factors at Ras Al-Hadd Reserve, Oman. In: Seminoff JA (ed) Proceedings of the Twenty-Second Annual Symposium on SeaTurtle Biology and Conservation. U.S. Dep. Commer. NOAA Tech. Memo. NMFS-SEFSC-503, Miami, Florida. 308 p, p 218-220

AlKindi A, Mahmoud I, Al-Gheilani H, Al-Bahry S, Bakheit CS (2002) Nest selection in green turtles, Chelonia mydas, relative to physical and biotic factors at Ras Al-Hadd Reserve, Oman. In: Seminoff JA (ed) Proceedings of the Twenty-Second Annual Symposium on SeaTurtle Biology and Conservation. U.S. Dep. Commer. NOAA Tech. Memo. NMFS-SEFSC-503, Miami, Florida. 308 p, p 221-222

Allard MW, Miyamoto MM, Bjorndal KA, Bolten AB, Bowen BW (1994) Support for Natal Homing in Green Turtles from Mitochondrial DNA Sequences. Copeia 1

Allen M, Turtlewatch Episkopi (2007) Western British Sovereign Base Area, Turtlewatch Episkopi Report. In: Mast RB, Bailey LM, Hutchinson BH (eds) SWoT Report—The State of the World's Sea Turtles, Volume II, Washington, DC. 49p. Available online *at*: http://seaturtlestatus.org/report/view

Alvabera E (2006) Revisión de la anidación de tortugas marinas en los campamentos del Centro Mexicano De La Tortuga durante el periodo 2001-2005. Paper presented at colloquium "Presencia del Centro Mexicano de la Tortuga en la Universidad Autónoma Benito Juárez de Oaxaca" Oaxaca de Juárez, Oax., 22 -23 May, 2006. 28pp

Alvarado-Diaz J, Delgado-Trejo C, Suazo-Ortuno I (2001) Evaluation of the Black Turtle Project in Michoacan, Mexico. Marine Turtle Newsletter 92:4-7

Amarasooriya KD, Jayathilaka MRA (2002) A classification of the sea turtles' nesting beaches of southern Sri Lanka. Paper presented at Second ASEAN Symposium on Sea Turtle Biology and Conservation

Amigos para la Conservacion de Cabo Pulmo and Grupo Tortuguero de las Californias (2007) Leatherback nesting in Baja California Sur, Mexico. In: Mast RB, Bailey LM, Hutchinson BH (eds) SWoT Report—The State of the World's Sea Turtles, Volume II, Washington, DC. 49p. Available online *at*: http://seaturtlestatus.org/report/view

Amorocho D (2003) Monitoring nesting loggerheads (*Caretta caretta*) in the central Caribbean Coast of Colombia. Marine Turtle Newsletter 101:8-13

Amorocho D (2007) Personal communication. Loggerhead nesting in Colombia. In: Mast RB, Bailey LM, Hutchinson BH (eds) SWoT Report—The State of the World's Sea Turtles, Volume II, Washington, DC. 49p. Available online *at*: http://seaturtlestatus.org/report/view

Amorocho DF, Reina RD (2007) Feeding ecology of the East Pacific green sea turtle Chelonia mydas agassizii at Gorgona National Park, Colombia. Endangered Species Research 3:43-51

Andrews H (2000) Current marine turtle situation in the Andaman and Nicobar Islands – An urgent need for conservation action. Kachhapa 3:21-25

Andrews H, Krishnan S, Biswas P (2001) The status and distribution of marine turtles around the Andaman and Nicobar archipelago.

Andrews H, Krishnan S, Biswas P (2006) Distribution and status of marine turtles in the Andaman and Nicobar Islands. In: Shanker K, Choudhury BC (eds) Marine Turtles of the Indian Subcontinent. Universities Press, India, Hyderguda, p 33-57

Andrews H, Krishnan S, Biswas. P (2006) The Status and Distribution of Marine Turtles Around the Andaman and Nicobar Archipelago. India: Andaman and Nicobar Islands Environmental Team, Center for Herpetology/Madras Crocodile Bank Trust. .

Andrews HV, Chandi M, Vaughan A, Aungthong J, Aghue S, Johnny S, John S, Naveen S (2006) Marine turtle status and distribution in the Andaman and Nicobar Islands after the 2004 M 9 quake and tsunami. Indian Ocean Turtle Newsletter 4:3

Andrews HV, Krishnan S, Biswas P (2002) Leatherback nesting in the Andaman & Nicobar Islands. Kachhapa 6:15-18

Andrews HV, Tripathy A (2004) Tracing the migrations of Indian marine turtles towards an integrated and collaborative conservation programme: Andaman and Nicobar Archipelago, India. An Interim Report to the Convention on the Conservation of Migratory Species of Wild Animals & United Nations Environment Programme Tamil Nadu, India: Madras Crocodile Bank Trust

Angoni H (2004) Suivi et Conservation des Tortues Marines Dans l'U.T.O. Campo - Ma'an. Rapport Technique

Arauz R, López E, Gaos A, Yañez I, Reyes W, Bejarano. S (2004) Sea turtle conservation and research using coastal community organizations as the cornerstone of support. PRETOMA, Costa Rica

Arauz R, Pyle A, Serna. JA (2004) Conservation of leatherback sea turtles, *Dermochelys coriacea*, and monitoring of sea turtle nesting activity in Playa Caletas and Playa Pencal, Costa Rica from July 15, 2003 to April 15, 2004. PRETOMA, Costa Rica

Argano R, Basso R, Cocco M, Gerosa G (1992) Nouvi dati sugli spostamenti di tartaruga marine commune (*Caretta caretta*) in Mediterraneo. Bollettino del Museo dell' Instituto di Biologia dell' Universita de Genova 56-57:137-163

Arias RGM (2006) Informe de Resultados de la temporada 2006. I Reunión de

Responsables de Centros y/o playas de Protección y Conservación de las tortugas Marinas. Centros Indígenas para la protección de la tortuga marina en Veracruz. Comisión Nacional para el Desarrollo de los Pueblos Indígenas. Centro de Desarrollo Indigenista. Acayucan, Ver, Organizado por la CONANP-PNSAV. Sala Multimedia del Ayuntamiento de Boca del río, Ver., 25 de noviembre de 2006.

Aronne M (2000) Anidación Semiartificial para la Conservación de Tortuga Marina Carey (Eretmochelys imbricata) en el Área Protegida de Cayos Cochinos, del 18 Junio al 30 Octubre 2000. Fundación Hondureña para los Arrecifes Coralinos (HCRF)

Arrinal (1997) Nesting green turtles at Meru Betiri National Park, Suka Made, East Java. (c/o C. Limpus) *in* FIVE YEAR REVIEW.

Aruna E (2006) Personal communication. Leatherback nesting in Sierra Leone. In: Mast RB, Bailey LM, Hutchinson BH (eds) SWoT Report—The State of the World's Sea Turtles, Volume I, Washington, DC. 36 p. Available online *at*: http://seaturtlestatus.org/report/view

Aruna E (2007) Personal communication. Loggerhead nesting in Sierra Leone. In: Mast RB, Bailey LM, Hutchinson BH (eds) SWoT Report—The State of the World's Sea Turtles, Volume I, Washington, DC. 36 p. Available online *at*: http://seaturtlestatus.org/report/view

Aruna E (2008) Personal Communication. Sea turtle nesting in Sierra Leone.

Arvy C, Dia AT, Colas F, Fretey J (2000) Records of *Caretta caretta* in Mauritania. Marine Turtle Newsletter 88:8

Asrar FF (1999) Decline of Marine Turtle Nesting Populations in Pakistan. Marine Turtle Newsletter 83:13-14

Association KAWAN (2006) Leatherback nesting in Martinique. In: Mast RB, Bailey LM, Hutchinson BH (eds) SWoT Report—The State of the World's Sea Turtles, Volume II, Washington, DC. 49p. Available online *at*: http://seaturtlestatus.org/report/view

Aureggi M (2006) Personal communication. Leatherback nesting in Thailand. In: Mast RB, Bailey LM, Hutchinson BH (eds) SWoT Report—The State of the World's Sea Turtles, Volume II, Washington, DC. 49p. Available online *at*: http://seaturtlestatus.org/report/view

Aureggi M, Gerosa G, Chantrapornsyl S (1999) Marine Turtle Survey at Phra Thong Island, South Thailand. Marine Turtle Newsletter 85:4-5

Avise J, Nelson W, Bowen B, Walker D (2000) Phylogeography of colonially nesting seabirds, with special reference to global matrilineal patterns in the sooty tern (<i>Sterna fuscata</i>). Molecular Ecology 9:1783-1792

Bacon PR (1971) Sea turtles in Trinidad and Toboago. Proceedings of the Second working meeting of the IUCN Marine Turtle Specialist Group. IUCN Publishings. New Series Supplemental Paper 31, p. 79-83

Bacon PR (1973) The status and management of sea turtles of Trinidad and Tobago. Unpublished report to the Permanent Secretary, Ministry of Agriculture 40 p

Bacon PR (1981) The status of sea turtle stocks management in the Western Central Atlantic, Western Central Atlantic Fishery Commission

Bal G, Breheret N, Girard A (2007) Rapport d'activite du programme d'etude et de sauvegarde des tortues marines au Congo. Saison 2006-2007, Renatura. 46p. Available online *at* www.renatura.asso.eu.org

Bal G, Breheret N, Girard A (2008) Rapport d'activite du programme d'etude et de sauvegarde des tortues marines au Congo. Saison 2007-2008. Renatura Available online *at* wwwrenaturaassoeuorg

Bal G, Breheret N, Vanleeuwe H (2007) An Update on Sea Turtle Conservation Activities in the Republic of Congo. Marine Turtle Newsletter 116:9-10

Bal G, Breheret NR (2006) Rapport d'activité du programme d'étude et de sauvegarde des tortues marines au Congo. Saison 2004-2005. In: Mast RB, Bailey LM, Hutchinson BH (eds) SWoT Report—The State of the World's Sea Turtles, Volume II, Washington, DC. 49p. Available online *at*: http://seaturtlestatus.org/report/view

Balazs GH, Chaloupka M (2003) Thirty-year recovery trend in the once depleted Hawaiian green sea turtle stock. Biological Conservation 117:491-498

Balazs GH, Chaloupka M (2004) Thirty-year recovery trend in the once depleted Hawaiian green sea turtle stock. Biological Conservation 117:491-498
Balazs GH, Keuper-Bennett U, Bennett P, Rice MR, Russell DJ (2002) Evidence for near shore nocturnal foraging by green turtles at Honokowai, Maui, Hawaii Islands. In: Seminoff JA (ed) Proceedings of the Twenty-Second Annual Symposium on Sea Turtle Biology and Conservation. U.S. Dep. Commer. NOAA Tech. Memo. NMFS-SEFSC-503 Miami, Florida. 308 p., p 32
Balazs GH, Murakawa KK, Wyneken J, Schroeder BA (1998) Differences in flipper size and esophagus morphology of green turtles from Hawaii and Florida. In: Epperly S, Braun J (eds) Proceedings of the seventeenth annual sea turtle symposium. U.S. Dep. Commer. NOAA Tech. Memo. NMFS-SEFSC-415, Orlando, Florida. 342 p.
Balazs GH, Puleloa W, Medeiros E, Murakawa SKK, Ellis DM (1998) Growth rates and incidence of fibropapillomatosis in Hawaiian green turtles utilizing coastal foraging pastures at Palaau, Molokai. In: Epperly S, Braun J (eds) Proceedings of the seventeenth annual sea turtle symposium. U.S. Dep. Commer. NOAA Tech. Memo. NMFS-SEFSC-415, Orlando, Florida. 342 p.
Balazs GH, Siu P, Landret J-P (1995) Ecological aspects of green turtles nesting at Scilly Atoll in French Polynesia. In: Richardson J, Richardson T (eds) Proceedings 12th Annual Workshop on Sea Turtle Biology and Conservation. U.S. Dep. Commer. NOAA Tech. Memo. NMFS-SEFSC-361, p. 7-10
Baldwin R, Hughes GR, Prince RIT (2003) Loggerhead turtles in the Indian Ocean. In: Bolten AB, Witherington B (eds) Loggerhead Sea Turtles. Smithsonian Institution Press, Washington, DC. 319 p., p 218-232
Bali J (2008) Hawksbill nesting in Sarawak, Malaysia. In: Mast RB, Bailey LM, Hutchinson BH (eds) SWoT Report—The State of the World's Sea Turtles, Volume III, Washington, DC. 43p. Available online *at*: http://seaturtlestatus.org/report/view
Bali J, Liew H-C, Chan E-H, Braken O (2000) Long Distance Migration of Green Turtles from the Sarawak Turtle Islands, Malaysia. In: Mosier A, Foley A, Brost B (eds) Proceedings of the Twentieth Annual Symposium on Sea Turtle Biology and Conservation. NOAA Tech. Memo. NMFS-SEFSC-477, Orlando, Florida. 369 p.
Baptistotte C (2007) Caracterização especial e temporal da fibropapilomatose em tartarugas marinhas da costa brasileira. PhD. Thesis. Universidade de São Paulo
Baptistotte C, Thome JCA, Bjorndal KA (2003) Reproductive biology and conservation status of the loggerhead sea turtle (Caretta caretta) in Espirito Santo State, Brazil. Chelonian Conservation and Biology 4:523-529
Barantes MVA (2006) Costa Rica Second Annual Report to the Inter-American Convention for the Protection and Conservation of Sea Turtles, National System of Conservation Areas, Ministry of Energy and the Environment, Peralta, Costa Rica. 23 p
Barata PCR, Fabiano FFC (2002) Evidence for Leatherback Sea Turtle (*Dermochelys coriacea*) Nesting in Arraial do Cabo, State of Rio de Janeiro, and a Review of Occasional Leatherback Nests in Brazil. Marine Turtle Newsletter 96:13-16
Barbosa C, Broderick AC, Catry P (1998) Marine Turtles in the Orango National Park (Bijagós Archipelago, Guinea-Bissau). Marine Turtle Newsletter:6-7
Barmes T, Eckert KL, Sybesma J (1993) Sea Turtle Recovery Action Plan for Aruba, Kingston, Jamaica. 58 p
Barnett LK, Emms C, Jallow A, Cham AM, Mortimer JA (2004) The distribution and conservation status of marine turtles in The Gambia, West Africa: a first assessment. Oryx 38:203-208
Barragan A, Dutton DM (2000) Genetic population structure of the leatherback turtle in the eastern Pacific: conservation implications. In: Abreu-Grobois FA, Briseno-Duenas D, Marquez-Millan. R, Sarti-Martinez AL (eds) Proceedings of the eighteenth international sea turtle symposium. NOAA Technical Memorandum NMFS-SEFSC-436, Mazatlán, Sinaloa, México. 293 p., p 154
Barragan A, Dutton PH, Abreu-Grobois FA (1998) Population genetics of the leatherback turtle in the Mexican Pacific. In: Epperly S, Braun J (eds)

Proceedings of the seventeenth annual sea turtle symposium. NOAA Technical Memorandum NMFS-SEFSC-415, Orlando, Florida. 342 p., p 6-7
Barragán A, Tavera A, Ocampo E, Escudero A (2004) Informe final de investigación de las actividades de conservación desarrolladas en la playa de Cahuitán durante la temporada 2003–2004. In: Sarti ML, Barragán RAR, Juárez JA (eds) Conservación y evaluación de la población de tortuga laúd Dermochelys coriacea en el Pacífico Mexicano, temporada de anidación 2003-2004 DGVS-SEMARNAT-Kutzari, Asociación para el Estudio y Conservación de las Tortugas Marinas AC
Barragán MJ, In The State of the World's Sea Turtles Report v (2006) Personal communication. Leatherback nesting in Ecuador. In: Mast RB, Bailey LM, Hutchinson BH (eds) SWoT Report—The State of the World's Sea Turtles, Volume I, Washington, DC. 36 p. Available online *at*: http://seaturtlestatus.org/report/view
Barrios-Garrido H, Montiel-Villalobos MG (2002) Present status of the green turtle (Chelonia mydas) in the Gulf of Venezuela. In: Seminoff JA (ed) Proceedings of the Twenty-Second Annual Symposium on SeaTurtle Biology and Conservation. U.S. Dep. Commer. NOAA Tech. Memo. NMFS-SEFSC-503, Miami, Florida. 308 p, p 257
Bass AL (1994) Conservation genetics of hawksbill turtles, *Eretmochelys imbricata*, in the Caribbean and Western Atlantic. M.S. Thesis. Louisiana State University
Bass AL (1996) Testing models of female reproductive migratory behavior and population structure in the Caribbean hawksbill turtle, *Eretmochelys imbricata*, with mtDNA sequences. Molecular Ecology 5:321-328
Bass AL (1999) Genetic Analysis to Elucidate the Natural History and Behavior of Hawksbill Turtles (*Eretmochelys imbricata*) in the Wider Caribbean: a Review and Re-Analysis. Chelonian Conservation and Biology 3:195-199
Bass AL, Epperly S, Braun-McNeill J (2004) Multi-year analysis of stock composition of a loggerhead turtle (*Caretta caretta*) foraging habitat using maximum liklihood and Bayesian methods. Conservation Genetics 5:783-796
Bass AL, Epperly SP, Braun-McNeill J (2006) Green Turtle (Chelonia mydas) Foraging and Nesting Aggregations in the Caribbean and Atlantic: Impact of Currents and Behavior on Dispersal. Journal of Heredity 97:346-354
Bass AL, Lagueux CJ, Bowen BW (1998) Mixed stock composition of the Miskitu Cay green turtle fishery based on mtDNA markers. In: Epperly S, Braun J (eds) Proceedings of the seventeenth annual sea turtle symposium. U.S. Dep. Commer. NOAA Tech. Memo. NMFS-SEFSC-415, Orlando, Florida. 342 p.
Bass AL, Lagueux CJ, Bowen BW (1998) Origin of Green Turtles, Chelonia mydas, at "Sleeping Rocks" off the Northeast Coast of Nicaragua. Copeia 4:1064-1069
Bass AL, Witzell WN (2000) Demographic Composition of Immature Green Turtles (Chelonia mydas) from the East Central Florida Coast: Evidence from mtDNA Markers. Herpetologica 56:357-367
Batibasaga A, Nand N (2008) Hawksbill nesting in Fiji. In: Mast RB, Bailey LM, Hutchinson BH (eds) SWoT Report—The State of the World's Sea Turtles, Volume III, Washington, DC. 43p. Available online *at*: http://seaturtlestatus.org/report/view
Beggs JA, Horrocks JA, Krueger BH (2007) Increase in hawksbill Eretmochelys imbricata turtle nesting in Barbados, West Indies. Endangered Species Research 3
Bell CD, Solomon JL, Blumenthal JM, Austin TJ, Ebanks-Petrie G, Broderick AC, Godley BJ (2007) Monitoring and conservation of critically reduced marine turtle nesting populations: lessons from the Cayman Islands. Animal Conservation 10:39-47
Bell CD, Solomon JL, Blumenthal JM, Austin TJ, Ebanks-Petrie G, Broderick AC, Godley BJ (In press) Monitoring and conservation of critically reduced marine turtle nesting populations: Lessons from the Cayman Islands. . Animal Conservation
Bell IP (2006) Milman Island Nesting Studies 8th to 29th January 2006. Queensland Parks and Wildlife Service
Bell IP, Miller JD, Dobbs KA, Limpus CJ (1999) Hawksbill turtle movements in the coral

sea. In: Abreu-Grobois FA, Briseno R, Marquez-M. R, Sarti L (eds) Proceedings of the 18th International Symposium on Sea Turtle Biology and Conservation, NOAA Tech. Memo. NMFS-SEFSC-436., p 95

Bellini C (1999) Nest reported in 1990 from Prado, Southern State of Bahia, Brazil. Barata, P.C.R. (recipient). Pers. comm.

Bellini C, Grosmann A, Lima EHSM (2008) *Chelonia mydas* (Linnaeus, 1758). In: Livro vermelho da fauna brasileira ameaçada de extinção Brasília: MMA, v2, (Biodiversitas, 19), p 359-361 *in*

Bellini C, Marcovaldi MA, Sanches TM, Grossman A, Sales G (1996) Atol das Rocas biological reserve: second largest Chelonia rookery in Brazil. Marine Turtle Newsletter 72:1-2

Ben Hassine S, Jribi I, Bradai MN, Bouain A, Echwkhi K, Karaa S (2008) Nesting Activity of Loggerhead Turtkes, *caretta caretta*, in Kuriat island, Tunisia (2006-2007) Third Mediterranean Conference on Marine Turtles, Tunisia, p 31

Ben-Tuvia A (undated) Sea turtles from the Mediterranean and Red Sea coasts of Israel and Sinai. Jerusalem, Hebrew University (Manuscript)

Ben-Tuvia A (undated) Sea turtles from the Mediterranean and Red Sea coasts of Israel and Sinai. Jerusalem, Hebrew University (Manuscript) *In* Frazier, J. and S. Salas (1984). "The status of marine turtles in the Egyptian Red Sea." Biological Conservation 30: 41-67.

Benson SR, Dutton PH, Hitipeuw C, Samber B, Bakarbessy J, Parker D (2007b) Post-Nesting Migrations of Leatherback Turtles (Dermochelys coriacea) from Jamursba-Medi, Bird's Head Peninsula, Indonesia. Chelonian Conservation and Biology 6:150-154

Benson SR, Forney KA, Harvey JT, Carretta JV, Dutton PH (2007c) Abundance, distribution, and habitat of leatherback turtles (Dermochelys coriacea) off California, 1990-2003. Fishery Bulletin 105:337-347

Benson SR, Kisokau KM, Ambio L, Rei V, Dutton PH, Parker D (2007a) Beach Use, Internesting Movement, and Migration of Leatherback Turtles, Dermochelys coriacea, Nesting on the North Coast of Papua New Guinea. Chelonian Conservation and Biology 6:7-14

Bentivegna F (2006) Report from Seaturtle.org Forum. Available online *at*: http://wwwseaturtleorg/gforum/gforumcgi?post=4320

Bentivegna F (2008) unpublished data.

Bentivegna F, Treglia G, Hochscheid S (2005) The first report of a loggerhead turtle *Caretta caretta* nesting on the central Tyrrhenian coast (western Mediterranean). JMBA2 - Biodiversity Records Published online: http://wwwmbaacuk/jmba/pdf/5143pdf

Bernerd F, Jun O, Elizabeth M, Simmons N, John M, Charles M, Hossain Yeamin M (2007) A Preliminary Assessment of the Green Sea Turtle *Chelonia mydas* Population and its Foraging Grounds in Kilifi Creek, Kenya. South Pacific Studies 28:31-41

Beyer K, Ekau W, Blay J (2002) Sea turtle nesting and the effect of predation on the hatching success of the olive Ridley (*Lepidochelys olivacea*) on Old Ningo Beach, Ghana, West Africa. In: Mosier A, Foley A, Brost B (eds) Proceedings of the Twentieth Annual Symposium on Sea Turtle Biology and Conservation. NOAA Technical Memorandum NMFS-SEFSC-477, Orlando, Florida. 370 pp., p 108-110

Beyer KM (2004) Reproductive output and nest location of olive ridley turtles (*Lepidochelys olivacea*) at Old Ningo Beach, Ghana, West Africa. In: Mast RB, Hutchinson BJ, Hutchinson AH (eds) Proceedings of the Twenty-Fourth Annual Symposium on Sea Turtle Biology and Conservation. U.S. Dep. Commer. NOAA Tech. Memo. NMFS-SEFSC-567, p. 114

Bhaskar S (1984) Sea turtles in North Andaman and other Andaman Islands. Report., WWF-India. 46 pp. *In* Andrews, H., S. Krishnan & P. Biswas. 2006. Distribution and status of marine turtles in the Andaman and Nicobar Islands. pp. 33-57. In: K. Shanker & B.C. Choudhury (ed.) Marine Turtles of the Indian Subcontinent, Universities Press, India, Hyderguda.

Bhaskar S (1993) The status and ecology of sea turtles in the Andaman and Nicobar Islands. ST 1/93. Centre for Herpetology/ Madras Crocodile Bank Trust, Tamil

Nadu, India. *In* Andrews, H., S. Krishnan & P. Biswas. 2006. Distribution and status of marine turtles in the Andaman and Nicobar Islands. pp. 33-57. In: K. Shanker & B.C. Choudhury (ed.) Marine Turtles of the Indian Subcontinent, Universities Press, India, Hyderguda.

Bhaskar S (1993b) Andaman and Nicobar sea turtle project. Phase- II: South Reef Island. Unpublished Report. Centre for Herpetology/ Madras Crocodile Bank Trust, Tamil Nadu, India. *In* Andrews, H., S. Krishnan & P. Biswas. 2006. Distribution and status of marine turtles in the Andaman and Nicobar Islands. pp. 33-57. In: K. Shanker & B.C. Choudhury (ed.) Marine Turtles of the Indian Subcontinent, Universities Press, India, Hyderguda.

Bhaskar S (1993c) Andaman and Nicobar sea turtle project. Phase- III. Unpublished report. Centre for Herpetology/ Madras Crocodile Bank Trust, Tamil Nadu, India. *In* Andrews, H., S. Krishnan & P. Biswas. 2006. Distribution and status of marine turtles in the Andaman and Nicobar Islands. pp. 33-57. In: K. Shanker & B.C. Choudhury (ed.) Marine Turtles of the Indian Subcontinent, Universities Press, India, Hyderguda.

Bhaskar S (1994a) Andaman and Nicobar sea turtle project. Phase- IV. Unpublished report. Centre for Herpetology/ Madras Crocodile Bank Trust, Tamil Nadu, India. *In* Andrews, H., S. Krishnan & P. Biswas. 2006. Distribution and status of marine turtles in the Andaman and Nicobar Islands. pp. 33-57. In: K. Shanker & B.C. Choudhury (ed.) Marine Turtles of the Indian Subcontinent, Universities Press, India, Hyderguda.

Bhaskar S (1994b) Andaman and Nicobar sea turtle project. Phase- V. Unpublished report. Centre for Herpetology/ Madras Crocodile Bank Trust, Tamil Nadu, India. *In* Andrews, H., S. Krishnan & P. Biswas. 2006. Distribution and status of marine turtles in the Andaman and Nicobar Islands. pp. 33-57. In: K. Shanker & B.C. Choudhury (ed.) Marine Turtles of the Indian Subcontinent, Universities Press, India, Hyderguda.

Bhaskar S (1994c) Andaman and Nicobar sea turtle project. Phase- VI. Unpublished report. Centre for Herpetology/ Madras Crocodile Bank Trust, Tamil Nadu, India. *In* Andrews, H., S. Krishnan & P. Biswas. 2006. Distribution and status of marine turtles in the Andaman and Nicobar Islands. pp. 33-57. In: K. Shanker & B.C. Choudhury (ed.) Marine Turtles of the Indian Subcontinent, Universities Press, India, Hyderguda.

Bhaskar S (1995a) Andaman and Nicobar sea turtle project. Phase- VIa. Unpublished report. Centre for Herpetology/ Madras Crocodile Bank Trust, Tamil Nadu, India. *In* Andrews, H., S. Krishnan & P. Biswas. 2006. Distribution and status of marine turtles in the Andaman and Nicobar Islands. pp. 33-57. In: K. Shanker & B.C. Choudhury (ed.) Marine Turtles of the Indian Subcontinent, Universities Press, India, Hyderguda.

Bhaskar S (1995b) Andaman and Nicobar sea turtle project. Phase- VII. Unpublished report. Centre for Herpetology/ Madras Crocodile Bank Trust, Tamil Nadu, India. *In* Andrews, H., S. Krishnan & P. Biswas. 2006. Distribution and status of marine turtles in the Andaman and Nicobar Islands. pp. 33-57. In: K. Shanker & B.C. Choudhury (ed.) Marine Turtles of the Indian Subcontinent, Universities Press, India, Hyderguda.

Bhaskar S (1995c) Andaman and Nicobar sea turtle project. Phase- VIII. Unpublished report. Centre for Herpetology/ Madras Crocodile Bank Trust, Tamil Nadu, India *In* Andrews, H, S Krishnan & P Biswas 2006 Distribution and status of marine turtles in the Andaman and Nicobar Islands pp 33-57 In: K Shanker & BC Choudhury (ed) Marine Turtles of the Indian Subcontinent, Universities Press, India, Hyderguda

Bhupathy S, Saravanan S (2006) Marine turtles of Tamil Nadu. In: Shanker K, Choudhury BC (eds) Marine Turtles of the Indian Subcontinent. Universities Press, India, Hyderguda, p 58-67

Billes A, Fretey J, Moundemba J-B (2003) Monitoring of leatherback turtles in Gabon. In: Seminoff JA (ed) Proceedings of the Twenty-Second Annual Symposium on SeaTurtle Biology and Conservation. NOAA Technical Memorandum NMFS-SEFSC-503, Miami, Florida. 308 p, p 131-132

Billes A, Fretey J, Verhage B, Huijbregts B, Giffoni B, Prosdocimi L, Albareda DA, Georges J-Y, Tiwari M (2006a) First evidence of leatherback movement from Africa to South America. Marine Turtle Newsletter 111:13-14

Billes A, Huijbregts B, Marmet J, Mounguengui A, Mamfoumbi J-C, Odzeano C (2006b) Nesting of sea turtles in the Gamba complex of protected areas: first monitoring of a nesting beach. Bulletin of the Biological Society of Washington 12:319-325

Binninger DM, Chin-Lenn MD, Perry GW, Lutz AP (1998) Differential gene expression in green turtle fibropapillomatosis. In: Epperly S, Braun J (eds) Proceedings of the seventeenth annual sea turtle symposium. U.S. Dep. Commer. NOAA Tech. Memo. NMFS-SEFSC-415, Orlando, Florida. 342 p.

Bioinsight/DIREN Guyane (2003) Plan de Restauration des Tortues Marines en Guyane, Direction Régionale de l'Environnement Guyane, Cayenne, Guyane. 90 p

Bitsindou A (2006) Rapport d'activité du programme d'étude et de sauvegarde des tortues marines au Congo. Saison 2005. 2006. 34 pp. *In* Bal, G., N. Breheret & H. Vanleeuwe. 2007. An Update on Sea Turtle Conservation Activities in the Republic of Congo. Marine Turtle Newsletter 116: 9-10.

Bjorndal KA, Bolten AB, Chaloupka M (2002) Estimates of survival probabilities for immature green turtles in the southern Bahamas. In: Seminoff JA (ed) Proceedings of the Twenty-Second Annual Symposium on Sea Turtle Biology and Conservation. U.S. Dep. Commer. NOAA Tech. Memo. NMFS-SEFSC-503, Miami, Florida. 308 p, p 3

Bjorndal KA, Bolten AB, Moreira L, Bellini C, Marcovaldi MA (2006) Population Structure and Diversity of Brazilian Green Turtle Rookeries Based on Mitochondrial DNA Sequences. Chelonian Conservation and Biology 5:262-268

Bjorndal KA, Bolten AB, Troeng S (2005) Population structure and genetic diversity in green turtles nesting at Tortuguero, Costa Rica, based on mitochondrial DNA control region sequences. Marine Biology 147:1449-1457

Bjorndal KA, Wetherall JA, Bolten AB, Mortimer JA (1999) Twenty-six years of nesting data from Tortuguero, Costa Rica: an encouraging trend. Conservation Biology 13:126-134

Blumenthal JM, Abreu-Grobois FA, Austin TJ, Broderick AC, Bruford MW, Coyne MS, Ebanks-Petrie G, Formia A, Meylan PA, Meylan AB, Godley BJ (2009) Turtle groups or turtle soup: dispersal patterns of hawksbill turtles in the Caribbean. Molecular Ecology 18:4841-4853

Blumenthal JM, Solomon JL, Bell CD, Austin TJ, Ebanks-Petrie G, Coyne MS, Broderick AC, Godley AC (2006) Satellite tracking highlights the need for international cooperation in marine turtle management. Endangered Species Research 2:51-61

Bolker B, Okuyama T, Bjorndal KA, Bolten AB (2003) Sea Turtle Stock Estimation Using Genetic Markers: Accounting for Sampling Error of Rare Genotypes. Ecological Applications 13:763-775

Bolker B, Okuyama T, Bjorndal KA, Bolten AB (2007) Incorporating multiple mixed stocks in mixed stock analysis: 'many-to-many' analyses. Molecular Ecology 16:685-695

Bolten A (2007) Personal communication. Loggerhead nesting in the Bahamas. In: Mast RB, Bailey LM, Hutchinson BH (eds) SWoT Report—The State of the World's Sea Turtles, Volume II, Washington, DC. 49p. Available online *at*: http://seaturtlestatus.org/report/view

Bolten A (2008) Personal communication. Hawksbill nesting in Bahamas. In: Mast RB, Bailey LM, Hutchinson BH (eds) SWoT Report—The State of the World's Sea Turtles, Volume III, Washington, DC. 43p. Available online *at*: http://seaturtlestatus.org/report/view

Bolten AB (2003) Active Swimmers - Passive Drifters: The Oceanic Juvenile Stage of Loggerheads in the Atlantic System. In: Bolten AB, Witherington B (eds) Loggerhead Sea Turtles. Smithsonian Books, Washington, DC. 319 p., p 63-78

Bolten AB, Bjorndal KA, Martins HR, Dellinger T, Biscoito MJ, Encalada SE, Bowen BW (1998) Transatlantic Development Migrations of Loggerhead Sea Turles

Demostrated by mtDNA Sequence Analysis. Ecological Applications 8:1-7
Bolten AB, Martins HR, Natali ML, Thome JC, Marcovaldi MA (1990) Loggerhead released in Brazil recaptured in Azores. Marine Turtle Newsletter 48:24-25
Boodram D, Reyes C, Osborne N, Zapata G, Rapetti J (2002) The influence of nesting zone on the hatching and emergence success of green turtles (Chelonia mydas) in Tortuguero, Costa Rica. In: Seminoff JA (ed) Proceedings of the Twenty-Second Annual Symposium on SeaTurtle Biology and Conservation. U.S. Dep. Commer. NOAA Tech. Memo. NMFS-SEFSC-503, Miami, Florida. 308 p, p 133-134
Bourjea J, Ciccione S (2008) Personal communication. Hawksbill nesting in French Overseas Territories. In: Mast RB, Bailey LM, Hutchinson BH (eds) SWoT Report—The State of the World's Sea Turtles, Volume III, Washington, DC. 43p. Available online *at*: http://seaturtlestatus.org/report/view
Bourjea J, Ciccione S, Rantsimbazafy R (2006) Marine turtle survey in Nosy Iranja Kely, northwestern Madagascar. Western Indian Ocean Journal of Marine Science 5:209-212
Bourjea J, Frappier J, Quillard M, Ciccione S, Roos D, Hughes GR, Grizel H (2007b) Mayotte Island: another important green turtle nesting site in the southwest Indian Ocean. Endangered Species Research 3
Bourjea J, Lapegue S, Gagnevin L, Broderick D, Mortimer JA, Ciccione S, Roos D, Taquet C, Grizel H (2007a) Phylogeography of the green turtle, Chelonia mydas, in the Southwest Indian Ocean. Molecular Ecology 16:175-186
Bourjea J, Nel R, Jiddawi NS, Koonjul MS, Bianchi G (2008) Sea Turtle Bycatch in the West Indian Ocean: Review, Recommendations and Research Priorities. Western Indian Ocean Journal of Marine Science 7:137-150
Bovenberg M, Vonk R (2007) The Hawksbill Turtle (*Eretmochelys imbricata*) on Fregate Island: The Hawksbill Turtle's Nesting Population and Nesting Habitat on Fregate During the North-West Monsoon in 2006–07. Unpublished report
Bowen B, Bass A, Chow S-M, Bostrom M, Bjorndal K, Bolten A, Okuyama T, Bolker B, Epperly S, Lacasella E, Shaver D, Dodd M, Hopkins-Murphy S, Musick J, Swingle M, Rankin-Baransky K, Teas W, Witzell W, Dutton P (2004) Natal homing in juvenile loggerhead turtles (<i>Caretta caretta</i>). Molecular Ecology 13:3797-3808
Bowen B, Grant W, Hillis-Starr Z, Shaver D, Bjorndal K, Bolten A, Bass A (2007) Mixed-stock analysis reveals the migrations of juvenile hawksbill turtles (<i>Eretmochelys imbricata</i>) in the Caribbean Sea. Molecular Ecology 16:49-60
Bowen BW, Abreu-Grobois FA, Balazs GH, Kamezaki N, Limpus CJ, Ferl RJ (1995) Trans-Pacific migrations of the loggerhead turtle (*Caretta caretta*) demonstrated with mitochondrial DNA markers. Proceedings of the National Academy of Sciences 92:3371-3734
Bowen BW, Bass AL, Soares LS, Toonen RJ (2005) Conservation implications of complex population structure: lessons from the loggerhead turtle (*Caretta caretta*). Molecular Ecology 14:2389-2402
Bowen BW, Clark AM, Abreu-Grobois FA, Chaves A, Reichart HA, Ferl RJ (1998) Global phylogeography of the ridley sea turtles (*Lepidochelys* spp.) as inferred from mitochondrial DNA sequences. Genetica 101:179-189
Bowen BW, Kamezaki N, Limpus CJ, Hughes GR, Meylan AB, Avise JC (1994) Global Phylogeography of the Loggerhead Turtle (*Caretta caretta*) as Indicated by Mitochondrial DNA Haplotypes. Evolution 48:1820-1828
Bowen BW, Karl SA (1997) Population genetics, phylogeography, and molecular evolution. In: Lutz PL, Musick JA (eds) The Biology of Sea Turtles. CRC Press, Inc., Boca Raton, Florida. 432 p., p 29-50
Bowen BW, Karl SA (2007) Population genetics and phylogeography of sea turtles. Molecular Ecology 16:4886-4907
Bowen BW, Meylan AB, Avise JC (1989) An odyssey of the green sea turtle: Ascension Island revisited. Evolution 86:573-576
Bowen BW, Meylan AB, Avise JC (1991) Evolutionary distinctiveness of the endangered Kemp's ridley sea turtle. Nature 352:709-711
Bowen BW, Meylan AB, Ross JP, Limpus CJ, Balazs GH, Avise JC (1992) Global

population structure and natural history of the green turtle (Chelonia mydas) in terms of matriarchal phylogeny. Evolution v46:p865(817)
Bowen BW, Nelson WS, Avise JC (1993) A molecular phylogeny for marine turtles: Trait mapping, rate assessment, and conservation relevance. Proceedings of the National Academy of Sciences 90:5574-5577
Boyle M (2007) Unpublished work cited in: Nichols, W.J. 2007. Loggerhead sea turtle (Caretta caretta) 5-year review: summary and evaluation. National Marine Fisheries Service and U.S. Fish and Wildlife Service. 65 p. James Cook University
Boyle MC, Fitzsimmons NN, Limpus CJ, Kelez S, Velez-Zuazo X, Waycott M (2009) Evidence for transoceanic migrations by loggerhead turtles in the southern Pacific Ocean. Proceedings of the Royal Society B 276:1993-1999
Bravo GPR, A. Barrios H (2001) Reporte de comisión a las playas de Tamiahua-Cabo Rojo, Ver, SEMARNAT-DGVS-Delegación de SEMARNAT en Veracruz, Inédito. 2 p.
Bravo GPR, R.C. Martínez P (2007) Breve reseña y resultados en la protección y conservación de las tortugas marinas en el Estado de Veracruz, 2003-2006, SEMARNAT-CONANP-PNSAV, En revisión
Brian C (2007) Personal communication. Loggerhead nesting in Angola. In: Mast RB, Bailey LM, Hutchinson BH (eds) SWoT Report—The State of the World's Sea Turtles, Volume II, Washington, DC. 49p. Available online *at*: http://seaturtlestatus.org/report/view
Briseno R, Abreu-Grobois FA, (ed.) (2006) Personal Communication. Olive ridley population estimates in Ixtapilla, Michoacan, Mexico. Cited in Marine Turtle Specialist Group. 2007. Red List Assessment-*Lepidochelys olivacea*. 39 p.
Briseño-Dueñas D (1998) Variación genética de la región control del ADN mitocondrial de poblaciones de la tortuga golfina (*Lepidochelys olivacea*) en el Pacífico oriental e implicaciones para su conservación. Master's Thesis. Universidad Autónoma
Broadstone M, Witherington B, Gorham J, Bresette M, Ehrhart LM, Bagley DA, Kubis S, Herren R (2002) Abundance and distribution of green turtles within shallow, hard-bottom foraging habitat adjacent to a Florida nesting beach. In: Seminoff JA (ed) Proceedings of the Twenty-Second Annual Symposium on SeaTurtle Biology and Conservation. U.S. Dep. Commer. NOAA Tech. Memo. NMFS-SEFSC-503, Miami, Florida. 308 p, p 242
Broderick A (2009) Unpublished data. In: Mast RB, Hutchinson BJ, Villegas PE, Wallace B, Yarnell L (eds) SWoT Report—The State of the World's Sea Turtles, Volume IV, Washington, DC. 49 p. Available online *at*: http://seaturtlestatus.org/report/view
Broderick AC, Coyne MS, Fuller WJ, Glen F, Godley BJ (2007) Fidelity and over-wintering of sea turtles. Proceedings of the Royal Society 274:1533-1538
Broderick AC, Frauenstein R, Glen F, Hays G, Jackson AL, Pelembe T, Ruxton GD, Godley AC (2006) Are green turtles globally endangered? Global Ecology and Biogeography 15:21-26
Broderick AC, Glen F, Godley BJ, Hays GC (2002) Estimating the number of green and loggerhead turtles nesting annually in the Mediterranean. Oryx 36:227-235
Broderick AC, Godley BJ, Hays GC (2001) Metabolic heating and the prediction of sex ratios for green turtles (*Chelonia mydas*). Physiological and biochemical zoology 74:161-170
Broderick AC, Godley BJ, Hays GC (2001a) Trophic status drives inter-annual variability in nesting numbers of marine turtles. Proceedings of the Royal Society 268:1481-1487
Broderick AC, Godley BJ, Hays GC (2001b) Monitoring and conservation of marine turtles of Ascension Island: a sustainable resource. Interim Report to Foreign and Commonwealth Office Environment Fund for the Overseas Territories 13 pp
Broderick AC, Godley BJ, Kelly A, McGowan A (1997) Glasgow University Turtle Conservation Expedition 1997: Expedition report. Marine Turtle Research Group, Graham Kerr Building, University of Glasgow, Glasgow G12 8QQ, Scotland 23pp
Broderick D, Moritz C (1996) Hawksbill breeding and foraging populations in the Indo-

Pacific region. In: Bowen BW, Witzell WN (eds) Proceedings of the International Symposium on Sea Turtle Conservation Genetics. NOAA Technical Memorandum NMFS-SEFSC-396. 173p., p 119-128
Broderick D, Moritz C, Miller JD, Guinea M, Prince RJ, Limpus CJ (1994) Genetic studies of the hawksbill turtle (*Eretmochelys imbricata*): evidence for multiple stocks in Australian waters. Pacific Conservation Biology 1:123-131
Brongersma L (1972) European Atlantic Turtles. Zoologsische Verhandelingen 121:1-318
Brooke MdL (1995) Seasonality and numbers of green turtles Chelonia mydas nesting on the Pitcairn Islands. Biological Journal of the Linean Society 56:325-327
Brooks LB, Nichols WJ, Harvey JT (2002) Estero Banderitas Marine Protected Area: a critical component to the recovery of the East Pacific green turtle (*Chelonia mydas*). In: Seminoff JA (ed) Proceedings of the Twenty-Second Annual Symposium on SeaTurtle Biology and Conservation. U.S. Dep. Commer. NOAA Tech. Memo. NMFS-SEFSC-503, Miami, Florida. 308 p, p 237-238
Brost B (2007) Personal communication. Loggerhead nesting in Florida. In: Mast RB, Bailey LM, Hutchinson BH (eds) SWoT Report—The State of the World's Sea Turtles, Volume II, Washington, DC. 49p. Available online *at*: http://seaturtlestatus.org/report/view
Brost B (2008) Personal communication. Hawksbill nesting in Florida, USA. In: Mast RB, Bailey LM, Hutchinson BH (eds) SWoT Report—The State of the World's Sea Turtles, Volume III, Washington, DC. 43p. Available online *at*: http://seaturtlestatus.org/report/view
Browne DC, Horrocks JA, Abreu-Grobois FA (2009) Population subdivision in hawksbill turtles nesting on Barbados, West Indies, determined from mitochondrial DNA control region sequences. Conservation Genetics Published online. URL: http://www.springerlink.com/content/x05w7j72744774ug
Buck Island Reef National Monument Sea Turtle Research Program (2006) Seasonal Report 2006. St Croix, US Virgin Islands: National Park Service, Buck Island Reef National Monument, Division of Resource Management unpublished report
Buck Island Sea Turtle Research Program, National Park Service (2009) Loggerhead nesting at Buck Island Reef National Monument, St. Croix, U.S. Virgin Islands. In: Mast RB, Hutchinson BJ, Villegas PE, Wallace B, Yarnell L (eds) SWoT Report—The State of the World's Sea Turtles, Volume IV, Washington, DC. 49 p. Available online *at*: http://seaturtlestatus.org/report/view
Bugoni L, Krause L, Petry MV (2001) Marine debris and human impacts on sea turtles in Southern Brazil. Marine Pollution Bulletin 42:1330-1334
Buitrago J, Guada HJ (2002) La Tortuga Carey (*Eretmochelys imbricata*) en Venezuela. Interciencia 27:392-399
Bulter JA (2001) Nesting Biology of the Sea Turtles of St. Kitts, West Indies. Chelonian Conservation and Biology 4:191-196
Burchfield PM, U.S. Fish and Wildlife Service, Gladys Porter Zoo, Secretaria de Medio Ambiente y Recursos Naturales, Secretaria de Desarrollo Urbano y Ecologia (2009) Report on the Mexico / United States of America population restoration project for the Kemp´s Ridley sea turtle, *Lepidochelys kempii*, on the coasts of Tamaulipas, Mexico. Report to the Gladys Porter Zoo, Brownsville Texas 11 pp
Burgess L, Booth D, Lanyon J (2002) The effect of incubation temperature on morphology and swimming performance of the green sea turtle hatchling. In: Seminoff JA (ed) Proceedings of the Twenty-Second Annual Symposium on SeaTurtle Biology and Conservation. U.S. Dep. Commer. NOAA Tech. Memo. NMFS-SEFSC-503, Miami, Florida. 308 p., p 184
Butynski TM (1996) Marine turtles on Bioko Island, Equatorial Guinea. Oryx 30:143-149
Byles R (1988) Satellite telemetry of Kemp's ridley sea turtle, *Lepidochelys kempii*, in the Gulf of Mexico. Report to the National Fish and Wildlife Foundation, Albuquerque, NM
Byles R (1989) Satellite Telemetry of Kemp's Ridley sea turtle, *Lepidochelys kempi*, in the Gulf of Mexico. In: Eckert SA, Eckert KL, Richardson TH (eds) Proceedings of the Ninth Annual Workshop on Sea Turtle Conservation and Biology. NOAA

Technical Memorandum NMFS-SEFSC-232, Jekyll Island, Georgia. 306 p., p 25-26
Byrne R (2006) Leatherback nesting in Dominica. In: Mast RB, Bailey LM, Hutchinson BH (eds) SWoT Report—The State of the World's Sea Turtles, Volume I, Washington, DC. 36 p. Available online *at*: http://seaturtlestatus.org/report/view
Byrne R (2006) Rosalie sea turtle initiative (RoSTI), unpublished report. 23 pp
Byrne R (2008) Personal communication. Hawksbill nesting in Dominica. In: Mast RB, Bailey LM, Hutchinson BH (eds) SWoT Report—The State of the World's Sea Turtles, Volume III, Washington, DC. 43p. Available online *at*: http://seaturtlestatus.org/report/view
Byrne R, Eckert K (2004) 2003 Annual Report: Rosalie Sea Turtle Initiative (RoSTI). Roseau, Dominica, West Indies. Prepared by WIDECAST for the Ministry of Agriculture and the Environment (Forestry, Wildlife and Parks Division)
Byrne R, Eckert KL (2006) Rosalie sea turtle initiative (RoSTI): Biennium project report 2004-2005, 51 p.
Caballero A (2006) Personal communication. Leatherback nesting in St. Maarten, Netherlands Antilles. In: Mast RB, Bailey LM, Hutchinson BH (eds) SWoT Report—The State of the World's Sea Turtles, Volume I, Washington, DC. 36 p. Available online *at*: http://seaturtlestatus.org/report/view
Calvo MV, Lezama C, Lopez-Mendilaharsu M, Fallabrino A, Coll J (2002) Stomach content analysis of stranded juvenile green turtles in Uruguay. In: Seminoff JA (ed) Proceedings of the Twenty-Second Annual Symposium on SeaTurtle Biology and Conservation. U.S. Dep. Commer. NOAA Tech. Memo. NMFS-SEFSC-503, Miami, Florida. 308 p, p 203-204
Camacho-Mosquera L, Amorocho DF, Mejia-Ladino LM, Palacio-Mejia JD, Rondon-Gonzalez F (2008) Caracterización genética de la colonia reproductiva de la tortuga marina golfina –*lepidochelys olivacea*– en el parque nacional natural gorgona (pacífico colombiano) a partir de secuencias de adn mitocondrial. Bol Invest Mar Cost 37:77-92
Campbell C (2002) Survival estimates of large juvenile and adult green turtles in the western Caribbean. In: Seminoff JA (ed) Proceedings of the Twenty-Second Annual Symposium on Sea Turtle Biology and Conservation. U.S. Dep. Commer. NOAA Tech. Memo. NMFS-SEFSC-503, Miami, Florida. 308 p, p 4
Campbell CL, Lagueux CJ, Huertas V (2007) 2006 Pearl Cays Hawksbill Conservation Project, Nicaragua. Wildlife Conservation Society Final Report
Campbell CL, Lagueux CJ, Mortimer JA (1996) Leatherback turtle, *Dermochelys coriacea*, Nesting at Tortuguero, Costa Rica in 1995. Chelonian Conservation and Biology 2:169-172
Canbolat AF (2004) A review of sea turtle nesting activity along the Mediterranean coast of Turkey. Biological Conservation 116:81-91
Canbolat AF (2007) BTC Crude Oil Pipeline Project. Turkey Environmental Department. Sea Turtle Expedition Project (STEP). 2005. . In: Mast RB, Bailey LM, Hutchinson BH (eds) SWoT Report—The State of the World's Sea Turtles, Volume II, Washington, DC. 49p. Available online *at*: http://seaturtlestatus.org/report/view
Caraccio M, Formia A, Hernandez M, Fallabrino A, Bruford MW (2006) Preliminary mixed stock analysis of juvenile green turtles in Uruguay using mitochondrial DNA sequences In: Proceedings of the 23rd Annual Symposium on Sea Turtle Biology and Conservation. US Dept of Commerce NOAA Technical Memorandum, Kuala Lumpur, Malaysia
Caraccio M, Naro-Maciel E, Hernandez M, Perez R (2007) Experiencia en la caraterizacion genetica de un area de alimentacion y desarrollo de tortuga verde...un caso uruguayo. In: Estrades A (ed) III Jornadas de Conservacion e Investigacion de Tortugas Marinas en el Atlantico Sur Occidental, Piriapolis, Uruguay, p 21-22
Caraccio MN, Domingo A, Marquez A, Naro-Maciel E, Miller P, Pereira A (2008) Las aguas del Atlantico Sudoccidental y su importancia en el ciclo de vida de la tortuga cabezona (*Caretta caretta*): evidencias a través del análisis del ADNmt. Collective Volume of Science papers, ICCAT 62:1831-1837
Caraccio MN, Naro-Maciel E, Hernández M., R. P (2005) Composición genética de la

tortuga verde (Chelonia mydas) en el rea de alimentación y desarrollo de Cerro Verde, Rocha, Uruguay. Jornada de Conservação e Pesquisa de Tartarugas Marinhas no Atlântico Sul Ocidental 14 e 15 de Novembro de 2005, Praia do Cassino, Brasil:39-41. Available online *at* http://www.seaturtle.org/PDF/NEMA_2005_ASO.pdf
Caribbean Conservation Corporation (2006) Personal communication. Loggerhead nesting in Chiriquí, Panama. In: Mast RB, Bailey LM, Hutchinson BH (eds) SWoT Report—The State of the World's Sea Turtles, Volume II, Washington, DC. 49p. Available online *at*: http://seaturtlestatus.org/report/view
Carr A, Meylan A, Mortimer J, Bjorndal K, Carr T (1982) Surveys of sea turtle populations and habitats in the Western Atlantic. U.S. Department of Commerce, National Marine Fisheries Service, NOAA Technical Memorandum NMFS-SEFC-91. 82 p
Carr A, Ross P, Carr S (1974) Internesting Behavior of the Green Turtle, Chelonia mydas, at a Mid-Ocean Island Breeding Ground. Copeia 1974:703-706
Carr T, Carr N (1991) Surveys of the Sea Turtles of Angola. Biological Conservation 58:19-29
Carreras C, Pascual M, Cardona L. M, Aguilar A, Margaritoulis D, Rees AF, Turkozan O, Levy Y, Gasith A, Aureggi M, Khalil M (2007) The genetic structure of the loggerhead sea turtle (*Caretta caretta*) in the Mediterranean as revealed by nuclear and mitochondrial DNA and its conservation implications. Conservation Genetics 8:761-775
Casale P (2008) Incidental catch of marine turtles in the Mediterranean Sea: captures, mortality, priorities. WWF Italy, Rome
Casale P, Abbate G, Freggi D, Conte N, Oliverio M, Argano R (2008) Foraging ecology of loggerhead sea turtles *Caretta caretta* in the central Mediterranean Sea: evidence for a relaxed life history model. Marine Ecology Progress Series 372:265-276
Casale P, Freggi D, Rocco M (2008) Mortality induced by drifting longline hooks and branchlines in loggerhead sea turtles, estimated through observation in captivity. Aquatic Conservation: Marine and Freshwater Ecosystems 18:945-954
Casale P, Margaritoulis D (2010) Sea turtles in the Mediterranean: Distribution, threats and conservation priorities., IUCN, Gland, Switzerland
Casale P, Margaritoulis D (In preparation) Overview. In: Casale P, Margaritoulis D (eds) Sea turtles in the Mediterranean: distribution, threats and conservation priorities
Casale P, Margaritoulis D (In preparation) Cyprus. In: Casale P, Margaritoulis D (eds) Sea turtles in the Mediterranean: distribution, threats and conservation priorities
Casale P, Margaritoulis D (In preparation) Turkey. In: Casale P, Margaritoulis D (eds) Sea turtles in the Mediterranean: distribution, threats and conservation priorities
Casey RN, Quackenbush SL, Work TM, Balazs GH, Bowser PR, Casey JW (1998) Evidence for retroviruses infections in green sea turtles from the Hawaiian Islands. In: Epperly S, Braun J (eds) Proceedings of the seventeenth annual sea turtle symposium. U.S. Dep. Commer. NOAA Tech. Memo. NMFS-SEFSC-415, Orlando, Florida. 342 p.
Castilhos JC, Tiwari M (2006) Preliminary Data and Observations from an Increasing Olive Ridley Population in Sergipe, Brazil. Marine Turtle Newsletter 113:6-7
Castro-Morales C, Campos-Rodriguez F (2006) Final Report: Research and Protection of the Leatherback, Green and Hawksbill Turtles of the Parismina River Mouth. Asociación Salvemos Las Tortugas de Parismina Unpublished report
Catry P, Barbosa C, Indjai B, Almeida A, Godley BJ, Vi J-C (2002) First census of the green turtle at Poilao, Bijagos Archipelago, Guinea-Bissau: the most important nesting colony on the Atlantic coast of Africa. Oryx 36:400-403
Ceballos-Fonseca C (2004) Distribucion de playas de anidacion y areas de almientacion de tortugas marinas y sus amenazas en el Caribe Colombiano. Bol Invest Mar Cost 33
Celini A, Soto JMR, Serafini TZ (2002) Fibropapillomatosis on green turtles, Chelonia mydas, on the southern Brazilian coast. In: Seminoff JA (ed) Proceedings of the

Twenty-Second Annual Symposium on SeaTurtle Biology and Conservation. U.S. Dep. Commer. NOAA Tech. Memo. NMFS-SEFSC-503, Miami, Florida. 308 p, p 300

CEROCOMA and PROTOMAC Rapport des Activités. Cameroon, Central Africa. Unpublished report

Chacón D, Carvajal JM (2004) Informe de la Anidación de Tortuga Baula (*Dermochelys coriacea*), en el Parque Nacional Cahuita, Limón, Costa Rica. Proyecto para la conservación de Tortugas Marinas del Caribe Sur, Talamanca, Costa Rica Temporada 2004

Chacón D, Hancock JM (2004) Anidación de la tortuga baula *Dermochelys coriacea* en Playa Gandoca, Talamanca, Costa Rica. Programa de Conservación de Tortugas Marinas del Caribe Sur, Talamanca, Costa Rica Temporada 2004

Chacón D, Machado J (2005) Informe de anidación en Playa Gandoca, Talamanca, Costa Rica. Refugio Nacional de Vida Silvestre Gandoca Manzanillo. Asociación ANAI/WIDECAST

Chacón D, McFarlane G (2005) Anidación de la tortuga baula (*Dermochelys coriacea*) en Playa Negra/Puerto Vargas, Parque Nacional Cahuita, Talamanca, Costa Rica. Asociacion ANAI/WIDECAST Informe de Actividades, temporada 2005

Chacon D, Quesada C, Drews C (2006) Hawksbill turtles of the Caribbean. Available online *at*: http://www.hawksbillwwf.org/php/English/index.php.

Chacón-Chaverrí D (2004) Synopsis of the leatherback turtle (*Dermochelys coriacea*). Inter-American Convention for the Protection and Conservation of Sea Turtles Document INF-16-04

Chacón-Chaverrí D (2008) Personal communication. Hawksbill nesting in Costa Rica. In: Mast RB, Bailey LM, Hutchinson BH (eds) SWoT Report—The State of the World's Sea Turtles, Volume III, Washington, DC. 43p. Available online *at*: http://seaturtlestatus.org/report/view

Chacon-Chaverri D, Eckert KL (2007) Leatherback Sea Turtle Nesting at Gandoca Beach in Caribbean Costa Rica: Management Recommendations from Fifteen Years of Conservation. Chelonian Conservation and Biology 6:101-110

Chaloupka M (2001) Historical trends, seasonality and spatial synchrony in green turtle egg production. Biological Conservation 101:263-279

Chaloupka M (2003) Stochastic simulation modelling of loggerhead sea turtle population dynamics given exposure to competing mortality risks in the western south Pacific. In: Bolten AB, Witherington B (eds) Loggerhead Sea Turtles. Smithsonian Institution Press, Washington, DC, 319 p., p 274-294

Chaloupka M, Balazs G (2007a) Using Bayesian state-space modelling to assess the recovery and harvest potential of the Hawaiian green sea turtle stock. Ecological Modelling 205:93-109

Chaloupka M, Balazs GH, Rice MR (2002) Spatial and temporal variation in Hawaiian green turtle somatic growth behavior. In: Seminoff JA (ed) Proceedings of the Twenty-Second Annual Symposium on Sea Turtle Biology and Conservation. U.S. Dep. Commer. NOAA Tech. Memo. NMFS-SEFSC-503, Miami, Florida. 308 p, p 35

Chaloupka M, Bjorndal KA, Balazs GH, Bolten AB, Ehrhart LM, Limpus CJ, Suganuma H, Troeng S, Yamaguchi M (2008a) Encouraging outlook for recovery of a once severely exploited marine megaherbivore. Global Ecology and Biogeography 17:297-304

Chaloupka M, Dutton P, Nakano H (2004) Status of sea turtle stocks in the Pacific Papers presented at the expert consultation on interactions between sea turtles and fisheries within an ecosystem context. FAO Fisheries Report no. 738 (supplement), Rome, Italy, p 135-164 *In*

Chaloupka M, Kamezaki N, Limpus CJ (2008b) Is climate change affecting the population dynamics of the endangered Pacific loggerhead sea turtle? Journal of Experimental Marine Biology and Ecology 356:136-143

Chaloupka M, Limpus CJ (1998) Modeling green turtle survivorship rates. In: Epperly S, Braun J (eds) Proceedings of the seventeenth annual sea turtle symposium. U.S. Dep. Commer. NOAA Tech. Memo. NMFS-SEFSC-415, Orlando, Florida. 342 p.

Chaloupka M, Limpus CJ (2001) Trends in the abundance of sea turtles resident in

southern Great Barrier Reef waters. Biological Conservation 102:235-249
Chaloupka M, TM W, GH B, SKK M, R M (2008c) Cause-specific temporal and spatial trends in green sea turtle strandings in the Hawaiian Archipelago (1982-2003). Marine Biology 154:887-898
Chan E (2004) Personal Communication. Cited in Seminoff, J.A., (assessor). 2004. Global Status Assessment: Green turtle (*Chelonia mydas*). Marine Turtle Specialist Group. Species Survival Commission, Red List Programme: 71.
Chan E-H (2006) Marine turtles in Malaysia: on the verge of extinction? Aquatic Ecosystems Health and Management 9:175-184
Chan E-H, Liew H-C, Mazlan AG (1988) The Incidental Capture of Sea Turtles in Fishing Gear in Terengganu, Malaysia. Biological Conservation 43:1-7
Chan EH, Liew HC (1996) Decline of the leatherback population in Terengganu, Malaysia, 1956-1995. Chelonian Conservation and Biology 2:196-203.
Chan SK-F, Cheng IJ, Zhou T, Wang H-J, Gu H-X, Song X-J (2007) A Comprehensive Overview of the Population and Conservation Status of Sea Turtles in China. Chelonian Conservation and Biology 6:185-198
Chan SKF, Chan JK, Lo LT, Balazs GH (2003) Satellite Tracking of the Post-nesting Migration of a Green Turtle (*Chelonia mydas*) from Hong Kong. Marine Turtle Newsletter 102:2-4
Chandler M (1991) New Records of Marine Turtles in Chile. Marine Turtle Newsletter 52:8-11
Chantrapornsyl S (1992) Biology and conservation olive ridley turtle (*Lepidochelys olivacea*, Eschscholtz) in the Andaman Sea, southern Thailand. Phuket Marine Biological Center Research Bulletin 57:51-66
Charuchinda M (2001) Pers. comm.
Charuchinda M, Monanunsap S (1998) Monitoring survey on sea turtle nesting in the Inner Gulf of Thailand, 1994–1996. Thailand Marine Fisheries Research Bulletin 6:17-25
Charunchinda M, Monanunsap S (2000) Reproductive biology of green turtle at Ko Khram Island, Chonburi Province, Thailand Proceedings of The First SEASTAR2000 Workshop, p.17-23
Chassin-Noria O, Abreu-Grobois A, Dutton PH, Oyama K (2004) Conservation Genetics of the East Pacific Green Turtle (Chelonia mydas) in Michoacan, Mexico. Genetica 121:195-206
Chatto R, Baker B (2007) The distribution and status of marine turtle nesting in the Northern Territory. Parks and Wildlife Service of the Northern Territory, Australia
Cháves G, Morera R, Aviles JR (2006) Leatherback nesting in the Ostional National Wildlife Refuge, Costa Rica. In: Mast RB, Bailey LM, Hutchinson BH (eds) SWoT Report—The State of the World's Sea Turtles, Volume I, Washington, DC. 36 p. Available online *at*: http://seaturtlestatus.org/report/view
Cháves G, Morera R, Aviles JR, Castro JC, Alvarado M (2005) Trends of the nesting activity of the "arribadas" of the olive ridley (*Lepidochelys olivacea*, Eschscholtz 1829), in the Ostional National Wildlife Refuge (1971-2003). Unpublished report
Chen T-H, Cheng IJ (1995) Breeding biology of the green turtle, *Chelonia mydas*, (Reptilia: Cheloniidae) on Wan-An Island, Peng-Hu Archipelago, Taiwan. I. Nesting ecology. Marine Biology 124:9-15
Cheng I-J (1997) Studies on Chinese sea turtles. Sichuan Journal of Zoology 15 (Supplement):27-50
Cheng I-J (2007) Personal communication. Loggerhead nesting in Xisha Archipelago, South China Sea. In: Mast RB, Bailey LM, Hutchinson BH (eds) SWoT Report—The State of the World's Sea Turtles, Volume II, Washington, DC. 49p. Available online *at*: http://seaturtlestatus.org/report/view
Cheng I-J, Chen T-H (1997) Short note The incidental capture of five species of sea turtles by coastal setnet fisheries in the eastern waters of Taiwan. Biological Conservation 82:235-239
Cheng IJ (1995) Sea turtles at Dungsha Tao, South China Sea. Marine Turtle Newsletter 70:13-14
Cheng IJ (1996) Sea Turtles at Taipin Tao, South China Sea. Marine Turtle Newsletter 75:6-8

Cheng IJ (2000) Post-nesting migrations of green turtles (*Chelonia mydas*) at Wan-An Island, Penghu Archipelago, Taiwan. Marine Biology 137:747-754
Cheng IJ (2000) Sea turtles at Dungsha Tao (Pratas Islands) and Taipin Tao (Spratly Islands), South China Sea, Vol. Academic Press
Cheng IJ (2002) Satellite telemetry of green turtles nesting at Taipin Tao, Nan-Sha Archipelago. In: Seminoff JA (ed) Proceedings of the Twenty-Second Annual Symposium on SeaTurtle Biology and Conservation. U.S. Dep. Commer. NOAA Tech. Memo. NMFS-SEFSC-503, Miami, Florida. 308 p, p 58
Cheng IJ (2007) Nesting Ecology and Postnesting Migration of Sea Turtles on Taipin Tao, Nansha Archipelago, South China Sea. Chelonian Conservation and Biology 6:277-282
Cheng IJ, Balazs GH (1998) The post-nesting long range migration of the green turtles that nest at Wan-An Island, PengHu Archipelago, Taiwan. In: Epperly S, Braun J (eds) Proceedings of the seventeenth annual sea turtle symposium. U.S. Dep. Commer. NOAA Tech. Memo. NMFS-SEFSC-415, Orlando, Florida. 342 p.
Cheng IJ, Huang C-T, Hung P-Y, Ke B-Z, Kuo C-W, Fong C-L (2009) Ten Years of Monitoring the Nesting Ecology of the Green Turtle, *Chelonia mydas*, on Lanyu (Orchid Island), Taiwan.
Chevalier J (2002) Status of green turtle (Chelonia mydas) populations nesting in the French West Indies. In: Seminoff JA (ed) Proceedings of the Twenty-Second Annual Symposium on SeaTurtle Biology and Conservation. U.S. Dep. Commer. NOAA Tech. Memo. NMFS-SEFSC-503, Miami, Florida. 308 p, p 134
Chevalier J (2005) Plan de restauration des tortues marines des Antilles Francaises, Office National de la Chasse et de al Faune Sauvage. Direction Regionale Outre Mer, 152 p.
Choudhury BC, Das SK, Ghose PS (2006) Marine turtles of West Bengal. In: Shanker K, Choudhury BC (eds) Marine Turtles of the Indian Subcontinent. Universities Press, India, Hyderguda, p 107-116
Clarke M, Campbell AC, Simms C, Hameid WS (2002) Observations on the Ecology of Marine Turtles Nesting on the Mediterranean Coast of Egypt. In: Mosier A, Foley A, Brost B (eds) Proceedings of the Twentieth Annual Symposium on Sea Turtle Biology and Conservation. NOAA Technical Memorandum NMFS-SEFSC-477, Orlando, Florida, 369 p, p 257-258
Cogger HG, Lindner DA (1969) Marine turtles in northern Australia. Australian Zoologist 15:150-159
CONANP, Comite Estatal para la Proteccion y Conservacion de las Tortugas Marinas del Estado de Campeche (2008) Hawksbill and Green nesting in Campeche, Mexico.
CONANP, Comité Estatal para la Protección y Conservación de las Tortugas Marinas del Estado de Campeche (2008) Hawksbill nesting in Campeche, Mexico. In: Mast RB, Bailey LM, Hutchinson BH (eds) SWoT Report—The State of the World's Sea Turtles, Volume III, Washington, DC. 43p. Available online *at*: http://seaturtlestatus.org/report/view
Conant TA, Dutton PH, Eguchi T, Epperly SP, Fahy CC, Godfrey MH, MacPherson SL, Possardt EE, Schroeder BA, Seminoff JA, Snover ML, Upite CM, Witherington BE (2009) LOGGERHEAD SEA TURTLE (CARETTA CARETTA) 2009 STATUS REVIEW UNDER THE U.S. ENDANGERED SPECIES ACT. Report of the Loggerhead Biological Review Team to the National Marine
Fisheries Service, August 2009:222 pp.
Cordero A, Arellano J, Gardner SC (2002) Trematode infection and resulting immunological mimetic in green sea turtles (Chelonia mydas agassizii) from Magdalena Bay, Baja California Sur, Mexico. In: Seminoff JA (ed) Proceedings of the Twenty-Second Annual Symposium on SeaTurtle Biology and Conservation. U.S. Dep. Commer. NOAA Tech. Memo. NMFS-SEFSC-503, Miami, Florida. 308 p, p 300-301
Cornelius S (1982) Status of sea turtles along the Pacific coast of middle America. In: Bjorndal KA (ed) Biology and Conservation of Sea Turtles. Smithsonian Institution Press, Washington, DC. 583 p, p 211-219
Costa A (2007) Personal communication. Loggerhead nesting in Mozambique. In: Mast RB, Bailey LM, Hutchinson BH (eds) SWoT Report—The State of the

World's Sea Turtles, Volume II, Washington, DC. 49p. Available online *at*: http://seaturtlestatus.org/report/view

Costa A (2007) Report of Marine Turtle Conservation in Quirimbas National Park, Cabo Delgado. Maputo, Mozambique: Marine Programme, WWF Mozambique

Costa A, Motta H, Pereira AM, Videira EJS, Louro MM, Joao J (2007) Marine Turtles in Mozambique: towards an effective conservation and management program Marine Turtle Newsletter 117:1-3

Craig P, Parker D, Brainard R, Rice M, Balazs G (2004) Migrations of green turtles in the central South Pacific. Biological Conservation 116:433-438

Cross H, Rizk C, Khalil M, Venizelos L (2005) Marine Turtle Conservation in the Mediterranean: Population Status and Conservation Activities on Sea Turtle Nesting Beaches in South Lebanon, 2005. Online *at*: http://wwwmedassetorg/pdf/Lebanon_Report_2005pdf

Cruce-Johnson J (2006) Yap State Sea Turtle Conservation Program, Ulithi Tagging Project, Gielop and Iar islands, Summer 2005. Yap State, Federated States of Micronesia: Marine Resources Management Division Report Unpublished report

Cruz MF (2004) Informe interno de la temporada 2003, Centro para la Protección y conservación de las tortugas marinas. Central Núcleo-eléctrica Laguna Verde. Inédito

Cruz R (2008) Personal communication. Hawksbill nesting in the Philippines. In: Mast RB, Bailey LM, Hutchinson BH (eds) SWoT Report—The State of the World's Sea Turtles, Volume III, Washington, DC. 43p. Available online *at*: http://seaturtlestatus.org/report/view

Cruz RD (2002) Marine Turtle Distribution in the Philippines. In: Kinan I (ed) Proceedings of the Western Pacific Sea Turtle Cooperative Research and Management Workshop. Western Pacific Regional Fishery Management Council, Honolulu, Hawaii. 290 p, p 57-65

Cuevas E, Canul-Rosado D, Tzeek-Tuz M, Muñoz-Terán K, Loyo-Buenfil F (2006) Reporte Final de Actividades de Conservación en las Playas de Anidación de Celestún y El Cuyo en Yucatán e Isla Holbox en Quintana Roo, México. Mérida, Yucatán, Mexico Pronatura Península de Yucatán. Unpublished report

Cuevas HRI (2005) Reporte Global de las cuatro temporadas (1996-1997-1998-1999) de protección de la tortuga verde (*Chelonia mydas*), Sociedad Cooperativa de Producción Pesquera "Santa Ana", En la localidad de Santander, Municipio de Alto Lucero de Gutiérrez Barrios, Veracruz, SEMARNAT. Delegación en Veracruz. Subdelegación de Gestión para la Protección del Medio Ambiente y Recursos Naturales. 13 p. Inédito

Cummings V (2002) Sea turtle conservation in Guam. In: Kinan I (ed) Proceedings of the Western Pacific Sea Turtle Cooperative Research and Management Workshop. Western Pacific Regional Fishery Management Council, Honolulu, Hawaii. 290 p., p 37-38

d'Auvergne C, Eckert KL (1993) WIDECAST Sea Turtle Recovery Action Plan for St. Lucia (Karen L. Eckert, Editor). CEP Technical Report No. 26. UNEP Caribbean Environment Programme, Kingston, Jamaica xiv + 70pp

d'Auvergne C, Eckert KL (1993) Sea Turtle Recovery Action Plan for St. Lucia,, UNEP Caribbean Environment Programme, Kingston, Jamaica. 70 p

Da Silva ACCD, Castilhos JC, Lopez GG, Barata PCR (2007) Nesting biology and conservation of the olive ridley sea turtle (*Lepidochelys olivacea*) in Brazil, 1991/1992 to 2002/2003. Journal of the Marine Biological Association of the United Kingdom 87:1047-1056

Da Silva ACCD, Castilhos JC, Rocha DAS, Oliveira FLC (2003) Nesting biology and conservation of the olive ridley sea turtle (*Lepidochelys olivacea*) in the state of Sergipe, Brazil. In: Seminoff JA (ed) Proceedings of the Twenty-Second Annual Symposium on Sea Turtle Biology and Conservation. NOAA Technical Memorandum NMFS-SEFSC-503, Miami, FL, 309 p, p 89

Davenport J (1998) The effects of current velocity and temperature upon swimming in juvenile green turtles

Chelonia mydas L. In: Epperly S, Braun J (eds) Proceedings of the seventeenth annual sea turtle symposium. U.S. Dep. Commer. NOAA Tech. Memo. NMFS-

SEFSC-415, Orlando, Florida. 342 p.
de Beauville-Scott S (1999) Beach and Mangal Systems of Saint Lucia., St. Lucia Ministry of Agriculture, Forestry and Fisheries. Unpublished report 24 p
De Dijn B (2003) Country report of Suriname: Marine turtle season 2002. In: I. Nolibos I, L. Kelle L, Thoisy BD, Lochon S (eds) Proceedings of the Sixth Sea Turtle Symposium for the Guianas, Remire–Montjoly, French Guiana, p 8-10
De Haro A, al. e (2007) Report on the 2006 Leatherback Program at Tortuguero, Costa Rica. Caribbean Conservation Corporation Unpublished report.
De Haro A, et al (2006) Report on the 2005 Green Turtle Program at Tortuguero, Costa Rica. Caribbean Conservation Corporation unpublished report.
de Haro A, Troeng S (2006a) Report on the 2005 Leatherback Program at Tortuguero, Costa Rica, Caribbean Conservation Corporation, Gainesville, Florida. Unpublished report. 49 p
de Haro A, Troeng S (2006b) Report on the 2005 Green Program at Tortuguero, Costa Rica, Caribbean Conservation Corporation, Gainesville, Florida. Unpublished report, 49 p
de los Llanos V (2002) Evaluación de la situación de las poblaciones de tortugas marinas en el Parque Nacional Archipiélago Los Roques, Universidad Central de Venezuela, Unpublished report. 77 p
De Ruyck C (2006) Leatherback nesting in the Bahamas. In: Mast RB, Bailey LM, Hutchinson BH (eds) SWoT Report—The State of the World's Sea Turtles, Volume I, Washington, DC. 36 p. Available online *at*: http://seaturtlestatus.org/report/view
De Silva AS (1986) Turtle tagging and international tag returns for Sabah, East Malaysia. Sarawak Museum Journal 34:263-273
De Silva AS (2006) An annotated bibliography of publications on marine turtles of Sri Lanka. Indian Ocean Turtle Newsletter 3:12-26
Debade X, et al. (2008) Report on the 2007 Green Turtle Program at Tortuguero, Costa Rica. Caribbean Conservation Corporation unpublished report
Debrot AO, Esteban N, Le Scao R, Caballero A, Hoetjes PC (2005) New Sea Turtle Nesting Records for the Netherlands Antilles Provide Impetus to Conservation Action. Caribbean Journal of Science 41:334-339
Debrot AO, Pors LJJ (1995) Sea Turtle nesting activity on northeast coast beaches in Curacao, 1993. Caribbean Journal of Science 31:333-338
Delaguerre M, Cesarini C (2004) Confirmed nesting of the loggerhead turtle in Corsica. Marine Turtle Newsletter 104:12
Delcroix E (2006) Rapport d'Activité Gestion du Réseau Tortues Marines de Guadeloupe 2006. Unpublished report
Delcroix E (2007) Rapport d'activité Gestion du Réseau Tortues Marines de Guadeloupe 2005. In: Mast RB, Bailey LM, Hutchinson BH (eds) SWoT Report —The State of the World's Sea Turtles, Volume II, Washington, DC. 49p. Available online *at*: http://seaturtlestatus.org/report/view
Delcroix E (2007) Rapport d'Activite Gestion du Reseau Tortues Marines de Guadeloupe 2007. Unpublished report
Delcroix E, DeProft P, Saint-Auret A, Dumont R, Guiougou F (2006) Leatherback nesting in Guadeloupe. In: Mast RB, Bailey LM, Hutchinson BH (eds) SWoT Report—The State of the World's Sea Turtles, Volume I, Washington, DC. 36 p. Available online *at*: http://seaturtlestatus.org/report/view
Delgado-Trejo C Pers. comm.
Department of the Environment W, Heritage and the Arts (2009) *Chelonia mydas* in Species Profile and Threats Database, Department of the Environment, Water, Heritage and the Arts, Canberra. Online *at*: http://wwwenvironmentgovau/sprat
Department of Wildlife Conservation Sri Lanka (2006) Marine Turtles of Sri Lanka. Online at: http://wwwdwlclk/cgi-bin/templatepl?turtle:%20%3E%20Turtle %20Conservation
Dermawan A (2002) Marine turtle management and conservation in Indonesia. In: Kinan I (ed) Proceedings of the Western Pacific Sea Turtle Cooperative Research and Management Workshop. Western Pacific Regional Fishery Management Council, Honolulu, Hawaii, 300 p, p 67-75
Dethmers K, Broderick D (2002) Green turtle fisheries in Australasia: assessing the

extent of their impact using mtDNA markers. In: Seminoff JA (ed) Proceedings of the Twenty-Second Annual Symposium on Sea Turtle Biology and Conservation. NOAA Technical Memorandum NMFS-SEFSC-503, Miami, Florida. 308 p, p 41-43

Dethmers KE, Broderick D, Moritz C, Fitzsimmons NN, Limpus CJ, Lavery S, Whiting S, Guinea M, Prince RIT, Kennet R (2006) The genetic structure of Australasian green turtles (*Chelonia mydas*): exploring the geographical scale of genetic exchange. Molecular Ecology 15:3931-3946

DFMR (2008) Ongoing nesting beach surveys.

Díaz SH (2004) Protección y Conservación de las tortugas marinas del campamento Boca de Lima, Municipio de Tecolutla, Veracruz, SEMARNAT-CONANP-PNSAV Inédito

Diaz-Fernandez R, Okayama T, Uchiyama T, Carillo E, Espinosa G, Diez CE, Koike H (1998) Genetic sourcing for the hawksbill turtle, *Eretmochelys imbricata*, in the northern Caribbean region. Chelonian Conservation and Biology 3:296-300

Diez CE (2005) Proyecto de Tortugas Marinas en Culebra. Puerto Rico Natural Resources Department (DRNA) Internal report

Diez CE, van Dam RP (2007) Mona and Monito Island, Puerto Rico, Hawksbill Turtle Research Project. Research Report for 2006 Unpublished report

Diez CE, Velez-Zuazo X, van Dam R (2002) In-water surveys of green sea turtles (Chelonia mydas) at Culebra Archipelago, Puerto Rico. In: Seminoff JA (ed) Proceedings of the Twenty-Second Annual Symposium on SeaTurtle Biology and Conservation. U.S. Dep. Commer. NOAA Tech. Memo. NMFS-SEFSC-503, Miami, Florida. 308 p, p 238

DNER (2004) Status of marine turtle nesting beach productivity in Puerto Rico. Internal report::3 p

Dobbs (2002) Personal Communication. Cited in Seminoff, J.A., (assessor). 2004. Global Status Assessment: Green turtle (*Chelonia mydas*). Marine Turtle Specialist Group. Species Survival Commission, Red List Programme: 71.

Dodd CKJ (1988) Synopsis of the biological data on the loggerhead sea turtle *Caretta caretta* (Linnaeus 1758). US Fish and Wildlife Service biological report 88(14). 110 pp.

Dodd M, Mackinnon A (2005) Loggerhead Turtle Nesting in Georgia, 2005. Georgia Department of Natural Resources, Brunswick, Georgia

Dominici G (1996) Monitoreo de anidamiento de tortuga tinglar (*Dermochelys coriacea*) en playas del Parque Nacional Jaragua. In Memorias del Segundo Congreso de la Biodiversidad Caribeña. Santo Domingo, Republica Dominicana, Jan 14–16, 1996

Donaldson A, Kerr R (2006) Personal communication. Leatherback nesting in Jamaica. In: Mast RB, Bailey LM, Hutchinson BH (eds) SWoT Report—The State of the World's Sea Turtles, Volume I, Washington, DC. 36 p. Available online *at*: http://seaturtlestatus.org/report/view

Donoso M, Dutton PH (2007) Distribucion, abundancia relativa y origen del stock de tortugas marinas capturadas incidentalmente en la flota palangrera chilena de pez espada XII Congreso Latino-Americano de Ciencias do Mar, Florianopolis, Uruguay

Donoso MP, Dutton DM, Serra R, Brito-Montero JL (2000) Sea turtles found in waters off Chile. In: Kalb H, Wibbels T (eds) Proceedings of the nineteenth annual symposium on sea turtle conservation and biology. NOAA Technical Memorandum NMFS-SEFSC-443, South Padre Island, Texas, U.S.A. 291 p., p 218-219

Dontaine J-F (2006) Unpublished data. Cited in Formia, A., B.J. Godley, J.-F. Dontaine & M.W. Bruford. 2006. Mitochondrial DNA diversity and phylogeography of endangered green turtle (*Chelonia mydas*) populations in Africa. Conservation Genetics 7: 353-369.

Dossou-Bodjrenou JS, Tehou A (2002) The status of efforts to protect Atlantic sea turtles in Benin (West Africa). In: Mosier A, Foley A, Brost B (eds) Proceedings of the Twentieth Annual Symposium on Sea Turtle Biology and Conservation. NOAA Technical Memorandum NMFS-SEFSC-477, Orlando, Florida, 369 p, p 108-110

Doussou Bodjrenou J, J.; M, Sagbo P (2005) Challenges and prospects for sea turtle conservation in Benin, West Africa. In: Coyne MS, Clark RD (eds) Proceedings of the Twenty-first Symposium on Sea Turtle Biology and Conservation. NOAA Technical Memorandum NMFS-SEFSC-528, Philadelphia, Pennsylvania, 368 pp.

Dow W, Eckert K, Palmer M, Kramer P (2007) An Atlas of Sea Turtle Nesting Habitat for the Wider Caribbean Region. The Wider Caribbean Sea Turtle Conservation Network and The Nature Conservancy. WIDECAST Technical Report No 6 Beaufort, North Carolina 267 pages, plus electronic Appendices

Dow WE, Eckert KL (2007) Sea Turtle Nesting Habitat - A Spatial Database for the Wider Caribbean Region. Wider Caribbean Sea Turtle Conservation Network (WIDECAST) and The Nature Conservancy. WIDECAST Technical Report No 6 Beaufort, North Carolina

Dupuy AR (1986) The Status of Marine Turtles in Senegal. Marine Turtle Newsletter 39:4-7

Durrell Wildlife Conservation Trust and St. Lucia Forestry Department (Ministry of Agriculture) (2008) Hawksbill nesting in St. Lucia. In: Mast RB, Bailey LM, Hutchinson BH (eds) SWoT Report—The State of the World's Sea Turtles, Volume III, Washington, DC. 43p. Available online *at*: http://seaturtlestatus.org/report/view

Dutton DM, Balazs GH, Dizon A, Barragan A (2000b) Genetic stock identification and distribution of leatherbacks in the Pacific: potential effects on declining populations. In: Abreu-Grobois FA, Briseno-Duenas D, Marquez-Millan. R, Sarti-Martinez AL (eds) Proceedings of the eighteenth international sea turtle symposium. NOAA Technical Memorandum NMFS-SEFSC-436, Mazatlán, Sinaloa, México. 293 p., p 38-39

Dutton DM, Dutton PH (1998) Accelerated growth in San Diego Bay green turtles? In: Epperly S, Braun J (eds) Proceedings of the seventeenth annual sea turtle symposium. U.S. Dep. Commer. NOAA Tech. Memo. NMFS-SEFSC-415, Orlando, Florida. 342 p.

Dutton PB, D. ; FitzSimmons, N. (2002a) Defining management units: molecular genetics. In: Kinan I (ed) Proceedings of the Western Pacific Sea Turtle Cooperative Research and Management Workshop. Western Pacific Regional Fishery Management Council, Honolulu, Hawaii. 290 p, p 93-101

Dutton PH (2005-2006) Building our knowledge of the leatherback stock structure. In: Mast RB, Bailey LM, Hutchinson BH (eds) SWoT Report—The State of the World's Sea Turtles, Volume I, Washington, DC. 36 p. Available online *at*: http://seaturtlestatus.org/report/view, p 10-11

Dutton PH (2008) Unpublished data. Cited in Dutton, P.H., G.H. Balazs, R.A. LeRoux, S.K.K. Murakawa, P. Zarate & L.S. Martinez. 2008. Composition of Hawaiian green turtle foraging aggregations: mtDNA evidence for a distinct regional population. Endangered Species Research 5: 37-44.

Dutton PH, Balazs GH, Dizon AE (1998) Genetic stock identification of sea turtles caught in the Hawaii-based pelagic longline fishery. In: Epperly S, Braun J (eds) Proceedings of the seventeenth annual sea turtle symposium. U.S. Dep. Commer. NOAA Tech. Memo. NMFS-SEFSC-415, Orlando, Florida. 342 p., p 43-44

Dutton PH, Balazs GH, LeRoux RA, Murakawa SKK, Zarate P, Martinez LS (2008) Composition of Hawaiian green turtle foraging aggregations: mtDNA evidence for a distinct regional population. Endangered Species Research 5:37-44

Dutton PH, Bowen BW, Owens DW, Barragan A, Davis SK (1999) Global phylogeography of the leatherback turtle (*Dermochelys coriacea*). Journal of Zoology (London) 248:397-409

Dutton PH, Dutton DL, Frey AN (2002b) Familial relationships among nesting female leatherbacks examined with genetic markers. In: Mosier A, Foley A, Brost B (eds) Proceedings of the Twentieth Annual Symposium on Sea Turtle Biology and Conservation. NOAA Technical Memorandum NMFS-SEFSC-477, Orlando, Florida. 370 pp., p 20

Dutton PH, Frey A, LeRoux R, Balazs GH (2000a) Molecular ecology of leatherback turtles in the Pacific. In: Pilcher NJ, Ismail G (eds) Sea turtles of the Indo-Pacific:

Research management and conservation. ASEAN Academic Press, London, p 248-253
Dutton PH, Hitipeuw C, Zein M, Benson SR, Petro G, Pita J, Rei V, Ambio L, Bakarbessy J (2007) Status and genetic structure of nesting populations of leatherback turtles (*Dermochelys coriacea*) in the western Pacific. Chelonian Conservation and Biology 6:47-53
Dutton PH, Roden S, Galver LM, Hughes GR (2003) Genetic population structure of leatherbacks in the Atlantic elucidated by microsatellite markers. In: Seminoff JA (ed) Proceedings of the Twenty-Second Annual Symposium on SeaTurtle Biology and Conservation. NOAA Technical Memorandum NMFS-SEFSC-503, Miami, Florida. 308 p, p 44-45
Dutton PHB, S. R. ; Eckert, S. A. (2006) Identifying origins of leatherback turtles from Pacific foraging grounds off Central California, USA. In: Pilcher NJ (ed) Proceedings of the Twenty-Third Annual Symposium on Sea Turtle Biology and Conservation, Kuala Lumpur, Malaysia. 261 p., p 228
Dye TS, Graham TR (2004) Review of Archaeological and Historical Data Concerning Reef Fishing in Hawaii and American Samoa. Western Pacific Regional Fishery Management Council:160 pp.
Eckert KL, Honebrink TD (1992) Sea Turtle Recovery and Action Plan for St. Kitts and Nevis,, UNEP Caribbean Environment Programme, Kingston, Jamaica. 116 p
Eckert KL, Overing JA, Lettsome BB (1992) Sea Turtle Recovery Action Plan for the British Virgin Islands, Kingston, Jamaica. 116 p
Eckert KL, Wallace B, Frazier J, Eckert SA, Pritchard P (2009) Synopsis of the biological data on the leatherback sea turtle *Dermochelys coriacea* (Vandelli, 1761). US Fish and Wildlife Service agency report 183 pp
Eckert S (2006a) High-use oceanic areas for Atlantic leatherback sea turtles (Dermochelys coriacea) as identified using satellite telemetered location and dive information. Marine Biology 149:1257-1267
Eckert SA, Bagley D, Kubis S, Ehrhart L, Johnson C, Stewart K, DeFreese D (2006b) Internesting and postnesting movements and foraging habitats of leatherback sea turtles (*Dermochelys coriacea*) nesting in Florida Chelonian Conservation and Biology 5:239-248
Ehrhart LE, Bagley DA, Redfoot WE (2003) Loggerhead turtles in the Atlantic Ocean: Geographic distribution, abundance, and population status. In: Bolten A, Witherington B (eds) Loggerhead Sea Turtles. Smithsonian Books, Washington, DC. 319 p., p 157-174
Ekanayake EML, Kapurusinghe T, Saman MM, Premakumara MGC (2002) Estimation of the number of leatherback (*Dermochelys coriacea*) nesting at the Godavaya turtle rookery in southern Sri Lanka during the nesting season in the year 2001. Kachhapa 6:13-14
Ekanayake EML, Ranawana KB, Kapurusinghe T, Premakumara MGC, Saman MM (2002) Marine turtle conservation in Rekawa turtle rookery in southern Sri Lanka. Ceylon Journal of Science (Biological Science) 30:79-88
Encalada SE, Bjorndal KA, Bolten AB, Zurita JC, Schroeder BA, Possardt F, Sears CJ, Bowen BW (1998) Population structure of loggerhead turtle (*Caretta caretta*) nesting colonies in the Atlantic and Mediterranean as inferred from mitochondrial DNA control region sequences. Marine Biology 130:567-575
Encalada SE, Lahanas PN, Bjorndal KA, Bolten AB, Miyamoto MM, Bowen BW (1996) Phylogeography and population structure of the Atlantic and Mediterranean green turtle Chelonia mydas: a mitochondrial DNA control region sequence assessment. Molecular Ecology 5:473-483
Engstrom TN, Bradley WG, Gray JA, Meylan aB, Meylan PA, Roess WB (1998) Genetic identity of green turtles in Bermuda waters. In: Epperly S, Braun J (eds) Proceedings of the seventeenth annual sea turtle symposium. U.S. Dep. Commer. NOAA Tech. Memo. NMFS-SEFSC-415, Orlando, Florida. 342 p.
Ergene S, Aymak C, Uçar AH (2006) Nesting activity of the marine turtle (Chelonia mydas and Caretta caretta) during 2005 in Alata, Mersin-Türkiye. In: Frick HCI, Panagopoulo A, Rees AF, Williams K (eds) Proceedings of the Twenty-sixth Annual Symposium on Sea Turtle Biology and Conservation, Island of Crete, Greece. 376 p, p 293

Erosa SA (2002) Informe de Resultados del Programa de Protección de Tortugas marina en la zona hotelera de Cancún, temporada 2002, Dirección General de Ecología, Benito Juárez Quintana Roo
Erosa SA (2003) Informe de Resultados del Programa de Protección de Tortugas marina en la zona hotelera de Cancún, temporada 2002, Dirección General de Ecología, Benito Juárez Quintana Roo
Erosa SA, Aguilar CC, Aguilar FS, T. Bernal V, Fanjul RR, R. Figueroa P, J. Juarez G, M. Rivero F (1994) Programa de Protección de la tortuga marina, Temporada 1994, CRIP Puerto Morelos, INP, SEPESCA-SEDESOL, México. Informe Final
Erosa SA, J Juarez G (1996) Primer registro de anidación de *Dermochelys coriacea* en la zona hotelera de Cancún, Jalapa, Veracruz, México
Erosa SA, J. Juarez G (1998) Turtle marine protection in the hotel zone of Cancun, Q. Roo: a retrospective *In* Mem 18vo International Symposium on sea turtle Biology and Conservation, Mazatlan, Mexico
Erzin T, Kıraç A, Kaska Y (2006) The spatial distribution of loggerhead sea turtles nests and their temperature and sex ratio variations on Dalaman Beach, Turkey. In: Frick HCI, Panagopoulo A, Rees AF, Williams K (eds) Proceedings of the Twenty-sixth Annual Symposium on Sea Turtle Biology and Conservation, Island of Crete, Greece. 376 p, p 293
Evans D, Ordonez C, Troeng S, Drews C (2007) Satellite tracking of leatherback turtles from Caribbean Central America reveals unexpected foraging grounds. In: Frick HCI, Panagopoulou A, Rees AF, Williams JA (eds) Proceedings of the Twenty-Seventh Annual Symposium on Sea Turtle Biology and Conservation. NOAA Technical Memorandum NMFS-SEFSC-569, Mrytle Beach, South Carolina. 261 p.
Evans KE, Vargas AR (1998) Sea turtle egg commercialization in Isla de Cañas, Panama. In: Byles R, Fernandez Y (eds) Proceedings of the Sixteenth Annual Symposium on Sea Turtle Biology and Conservation. NOAA Technical Memorandum NMFS-SEFSC-412, Hilton Head, South Carolina. 158 p., p 45
Everlasting Nature of Asia Project in Indonesia. Online *at* http://www.elna.or.jp/en/pj_id/index.html.
Fastigi M, YWF - Kido Foundation (2008) Personal Communication. Nesting on Carriacou Island, Grenada.
Fernandes (2004) Variabilidade no DNA mitocondrial de Lepidochelys olivacea (Tartaruga oliva) na costa brasileria. 50° Congresso Brasilerio de Genetica
Ferraroli S, Eckert SA, Le Maho Y (2004) Satellite tracking of leatherback turtles nesting in French Guiana In: Coyne MS, Clark RD (eds) Proceedings of the Twenty-First Annual Symposium on Sea Turtle Biology and Conservation. NOAA Technical Memorandum NMFS-SEFSC-528, Philadelphia, Pennsylvania, 368 pp., p 168
Ferreira B Personal Comunication. To Renatura concerning CM exchange between Angola and Congo
Ferreira MB, Garcia M, Al-Kiyumi A (2002) Human and natural threats to the green turtles, Chelonia mydas, at Ra's al Hadd turtle reserve, Arabian Sea, Sultanate of Oman. In: Seminoff JA (ed) Proceedings of the Twenty-Second Annual Symposium on SeaTurtle Biology and Conservation. U.S. Dep. Commer. NOAA Tech. Memo. NMFS-SEFSC-503, Miami, Florida. 308 p, p 142
Ferreira MB, Garcia M, Jupp B, Al-Kiyumi A (2002) Feeding ecology of the green turtle, Chelonia mydas, at Ra's Al Hadd, Arabian Sea, Sultanate of Oman. In: Seminoff JA (ed) Proceedings of the Twenty-Second Annual Symposium on SeaTurtle Biology and Conservation. U.S. Dep. Commer. NOAA Tech. Memo. NMFS-SEFSC-503, Miami, Florida. 308 p., p 205-206
Fiedler FN (2009) As pescarias industriais de rede de emalhe de superficie e as tartarugas marinhas: Caracterização das frotas de itajaí, navegantes, porto belo (Santa Catarina) e ubatuba (São Paulo), suas áreas de atuação, sazonalidade e a interação com as tartarugas marinhas. M.Sc. Thesis. Universidade Federal do Paraná
Firdous F (1991) A Turtle's Journey from Pakistan (Karachi) to India (Gujarat). Marine Turtle Newsletter 53:18-19
Fisheries Department of Malaysia (2006) Report on the marine turtle management

program in Terengganu for 2005. Economic Planning Unit, State Secretary Presented at the Meeting of the Turtle Sanctuary Advisory Council, Meeting No. 1/2006, 12 August 2006
Fitzsimmons NN (2009) Personal communication. Known genetic stocks of the flatback turtle. In: Mast RB, Hutchinson BH, Villegas PE, Wallace B, Yarnell L (eds) SWoT Report—The State of the World's Sea Turtles, vol IV, Washington, DC. 49 p. Available online *at*: http://seaturtlestatus.org/report/view, p 19-25
FitzSimmons NN (1996b) Use of microsatellite loci to investigate multiple paternity in marine turtles. In: Bowen BW, Witzell WN (eds) Proceedings of the International Symposium on Sea Turtle Conservation Genetics. NOAA Technical Memorandum NMFS-SEFSC-396. 173pp.
FitzSimmons NN, Limpus CJ, Norman JA, Goldizen AR, Miller JD, Moritz C (1997a) Philopatry of male marine turtles inferred from mitochondrial DNA markers. Proceedings of the National Academy of Sciences 94:8912-8917
FitzSimmons NN, Moritz C, Limpus CJ, Miller JD, Parmenter CJ, Prince RIT (1996a) Comparative genetic structure of green, loggerhead, and flatback populations in Australia based on variable mtDNA and nDNA regions. In: Bowen BW, Witzell W (eds) Proceedings of the International Symposium on Sea Turtle Conservation Genetics, p 173
FitzSimmons NN, Moritz C, Limpus CJ, Pope L, Prince R (1997b) Geographic Structure of Mitochondrial and Nuclear Gene Polymorphisms in Australian Green Turtle Populations and Male-Biased Gene Flow. Genetics 147:1843-1854
Fitzsimmons NN, Moritz C, Moore SS (1995) Conservation and Dynamics of Microsatellite Loci over 300 Million Years of Marine Turtle Evolution. Molecular Biology and Evolution 12:432-440
Flora Fauna y Cultura de México (2006) Reporte del Programa de Protección y Conservación de Tortugas Marinas en el Litoral Central del Estado de Quintana Roo Temporada 2005.63 p
Flora Fauna y Cultura de Mexico AC (2008) Programa de Proteccion y Conservacion de Tortugas Marinas en el Litoral Central del Estado de Quintana Roo Temporada 2008. Report 48 p
Flores ENJ (2005) Estado de la situación de conservación comunitaria de tortugas marinas en la Reserva de Biosfera de Río Plátano: Una experiencia de gestión colectiva en la comunidad de Plaplaya, Municipio de Juan Francisco Bulnes, "Walumugu", 1995-2005. . Unpublished report
Florida Fish and Wildlife Conservation Commission, Fish and Wildlife Research Institute (2006) Nesting Activity reports 2005 data. Available online *at*: http://research.myfwc.com/features/view_article.asp?id=2377.
Florida Fish and Wildlife Conservation Commission, Fish and Wildlife Research Institute: Marine Turtle Program Leatherback Nesting in Florida. Online *at*: http://researchmyfwccom/features/view_articleasp?id=2479
Florida Fish and Wildlife Conservation Commission, Fish and Wildlife Research Institute: Marine Turtle Program Loggerhead Nesting in Florida. Online *at*: http://researchmyfwccom/features/view_articleasp?id=11812
Florida Fish and Wildlife Conservation Commission: Fish and Wildlife Research Institute Statewide Nesting Beach Survey Program. Online *at*: http://researchmyfwccom
Florida Fish and Wildlife Conservation Commission: Fish and Wildlife Research Institute (2009) Reported nesting activity of the Kemp's ridley, *Lepidochelys kempii*, in Florida, 1979-2008.1 p.
Florida Fish and Wildlife Conservation Commission: Fish and Wildlife Research Institute, The State of Florida (2008) Turtle Nesting Beaches. Available online *at*: http://ocean.floridamarine.org/mrgis_ims/Description_Layers_Marine.htm#turtle
Foley A, Dutton DM, Singel KE, Redlow AE, Teas WG (2003) The First Records of Olive Ridleys in Florida, USA. Marine Turtle Newsletter 101:23-25
Foley MM, Halpern BS, Micheli F, Armsby MH, Caldwell MR, Crain CM, Prahler E, Rohr N, Sivas D, Beck MW, Carr MH, Crowder LB, Emmett Duffy J, Hacker SD, McLeod KL, Palumbi SR, Peterson CH, Regan HM, Ruckelshaus MH, Sandifer

PA, Steneck RS Guiding ecological principles for marine spatial planning. Marine Policy In Press, Corrected Proof
Formia A (1999) Les tortues marines de la baie de Corisco. Canopee 14:i-ii
Formia A, Broderick AC, Glen F, Godley BJ, Hays GC, Bruford MW (2007) Genetic composition of the Ascension Island green turtle rookery based on mitochondrial DNA: implications for sampling and diversity. Endangered Species Research 3:145-158
Formia A, Bruford MW (2002) Green turtle (Chelonia mydas) nesting and feeding populations along the Atlantic Coast of Africa described through mitochondrial DNA. In: Seminoff JA (ed) Proceedings of the Twenty-Second Annual Symposium on Sea Turtle Biology and Conservation. U.S. Dep. Commer. NOAA Tech. Memo. NMFS-SEFSC-503, Miami, Florida. 308 p, p 39
Formia A, Godley BJ, Dontaine J-F, Bruford MW (2006) Mitochondrial DNA diversity and phylogeography of endangered green turtle (*Chelonia mydas*) populations in Africa. Conservation Genetics 7:353-369
Formia A, Tiwari M, Fretey J, Billes A (2003) Sea Turtle Conservation along the Atlantic Coast of Africa. Marine Turtle Newsletter 100:33-37
Fossette S, Girard C, L√≥pez-Mendilaharsu M, Miller P, Domingo As, Evans D, Kelle L, Plot V, Prosdocimi L, Verhage S, Gaspar P, Georges J-Y Atlantic Leatherback Migratory Paths and Temporary Residence Areas. PLoS ONE 5:e13908
Fournillier K, Eckert KL (1998) Draft WIDECAST Sea Turtle Recovery Action Plan for Trinidad and Tobago, United Nations Caribbean Environment Programme, Kingston, Jamaica
Francia G (2006) Proyecto de Conservacion Baulas del Pacífico de Junquillal (WWF). In: Mast RB, Bailey LM, Hutchinson BH (eds) SWoT Report—The State of the World's Sea Turtles, Volume II, Washington, DC. 49p. Available online *at*: http://seaturtlestatus.org/report/view
Francia G (2008) Proyecto de Conservación Baulas del Pacífico de Junquillal. World Wildlife Fund
Frazier J (1980) Exploitation of Marine Turtles in the Indian Ocean. Human Ecology 8:329-370
Frazier J (2000) Kachhapa - Ashoka's most accomplished ambassador. Kachhapa 3:3-4
Frazier J (2003) Prehistoric and Ancient Historic Interactions between Humans and Marine Turtles. In: Lutz PL, Musick JA, Wyneken J (eds) The Biology of Sea Turtles, Volume II. CRC Press LLC, Boca Raton, Florida, U.S.A. 455 p., p 1-38
Frazier J (2006) India's marine turtles: sentinels from antediluvian to post-modern times. Journal of the Bombay Natural History Society 103:401-407
Frazier J, Salas S (1984) The status of marine turtles in the Egyptian Red Sea. Biological Conservation 30:41-67
Frazier J, Salas S, Hassan Didi NT (2000) Marine Turtles in the Maldive Archipelago. Maldives Marine Research Bulletin 4:80 pp
Fretey J (1989b) Reproduction de la Tortue olivâtre (*Lepidochelys olivacea*) en Guyane française pendant la saison
1987. Nature guyanaise 1:8-13
Fretey J (1999) Repartition des tortues du genre *Lepidochelys* Fitzinger, 1843. I. L'Atlantique ouest. Biogeographica 75:97-117
Fretey J (2001) Biogeography and conservation of marine turtles of the Atlantic coast of Africa, Vol. Secretariat, Convention on Migratory Species, CMS technical series publication, no. 6, Bonn, Germany
Fretey J, Billes A, Baxter B, Hughes C (2007a) Discovery of a Gabonese leatherback in South Africa. Marine Turtle Newsletter 116:25
Fretey J, Billes A, Tiwari M (2007) Leatherback, Dermochelys coriacea, Nesting Along the Atlantic Coast of Africa. Chelonian Conservation and Biology 6:126-129
Fretey J, Billes A, Tiwari M (2007b) Leatherback, *Dermochelys coriacea*, nesting along the Atlantic coast of Africa. Chelonian Conservation and Biology 6:126-129
Fretey J, Formia A, Tomas J, Dontaine J-F, Billes A, Angoni H (2005) Presence, nesting, and conservation of *Lepidochelys olivacea* in the Gulf of Guinea. In: Coyne MS, Clark AM (eds) Proceedings of the Twenty-first Symposium on Sea Turtle Biology and Conservation. NOAA Technical Memorandum NMFS-

SEFSC-528, Philadelphia, Pennsylvania, 368 pp., p 172
Fretey J, Girardin N (1989a) Preliminary data on the marine turtles on Gabon. Compte Rendu des Seances, Societe de Biogeographie 65:39-57
Fretey J, Malaussena J-P (1991) Sea Turtle Nesting in Sierra Leone, West Africa. Marine Turtle Newsletter 54:10-12
Frontier-Madagascar (2003) Artisanal and traditional turtle resource utilisation in South West Madagascar. Society for Environmental Exploration, UK and the Institute of Marine Sciences, University of Toliara, Madagascar Frontier-Madagascar Environmental Research Report
Fuentes MMPB, Dawson J, Smithers S, Limpus CJ, Hamann M (2010c) Sedimentological characteristics of key sea turtle rookeries: potential implications under projected climate change. Journal of Marine and Freshwater Research 61:464-473
Fuentes MMPB, Hamann M, Limpus CJ (2010a) Past, current and future thermal profiles of green turtle nesting grounds: Implications from climate change. Journal of Experimental Marine Biology and Ecology 383:56-64
Fuentes MMPB, Limpus CJ, Hamann M (2010d) Vulnerability of sea turtle nesting grounds to climate change. Global Change Biology
Fuentes MMPB, Limpus CJ, Hamann M, Dawson J (2010b) Potential impacts of projected sea level rise to sea turtle rookeries. Aquatic Conservation: Marine and Freshwater Systems 20:132-139
Fuentes MMPB, Maynard JA, Guinea M, Bell IP, Werdell PJ, Hamann M (2009) Proxy indicators of sand temperature help project impacts of global warming on sea turtles. Endangered Species Research 9:33-40
Fuller JE, Eckert KL, Richardson JI (1992) Sea Turtle Recovery Action Plan for Antigua and Barbuda, Kingston, Jamaica. 88 p
Fuller WJ, Broderick AC, Godley BJ, Walker J (2005) Marine Turtle Conservation Project Northern Cyprus 2005. Annual Report:15 p
Fundación Mario Dary Rivera: Consejo Nacional de Áreas Protegidas, The Nature Conservancy (2006) Plan de Conservación de Área 2007–2011 Refugio de Vida Silvestre Punta de Manabique. Guatemala: FUNDARY-PROARCA-TNC
Galante I (2007) Report from Seaturtle.org Forum. Available online *at*: http://wwwseaturtleorg/gforum/gforumcgi?post=5918
Galia F, Freggi D, d'Angelo S, Lo Valvo M (2006) An unusual nest activity along southern Sicilian Coasts: An hope for sea turtle survival? In: Frick HCI, Panagopoulo A, Rees AF, Williams K (eds) Proceedings of the Twenty-sixth Annual Symposium on Sea Turtle Biology and Conservation, Island of Crete, Greece. 376 p
Gallardo A (2007) Importancia de las playas del este del Estado Vargas para la anidación de las tortugas marinas, Universidad Central de Venezuela, unpublished report. 103 p
Gallo BMG, Macedo S, Giffoni B. de B., Becker JH, Barata PCR (2006) Sea turtle conservation in Ubatuba, Southeastern Brazil, a feeding area with incidental capture in coastal fisheries. Chelonian Conservation and Biology 5:93-101
Gaos AR, Abreu-Grobois FA, Alfaro-Shigueto J, Amorocho D, Arauz R, Baquero A, Briseño R, Chacón D, ; , Dueñas C, Hasbún C, Liles M, Mariona G, Muccio C, Muñoz JP, Nichols WJ, Peña M, Seminoff JA, Vásquez M, Urteaga J, Wallace B, Yañez IL, Zárate. P (2010) Signs of hope in the eastern Pacific: International collaboration reveals encouraging status for the severely depleted hawksbill turtles. Oryx
Gaos AR, Yañez IL, Arauz RM (2006) Sea Turtle Conservation and Research on the Pacific Coast of Costa Rica. Programa Restauración de Tortugas Marinas (PRETOMA) Technical Report
Garcia M, Ferreira MB, Calvario J, Al-Kiyumi A, Jupp B (2002) The first report on epizoic algae of nesting green turtles, Chelonia mydas, at Ra's Al Hadd Turtle Reserve, Arabian Sea, Oman. In: Seminoff JA (ed) Proceedings of the Twenty-Second Annual Symposium on SeaTurtle Biology and Conservation. U.S. Dep. Commer. NOAA Tech. Memo. NMFS-SEFSC-503, Miami, Florida. 308 p
García NE (2005) Protección y Conservación de las tortugas marinas del campamento "Tortuga de la Mar" Municipio de Cazones de Herrera, Veracruz, Ayuntamiento

de Cazones de Herrera. Inédito
Garner JA, Garner SA, Coles W (2005) Tagging and Nesting Research on Leatherback Sea Turtles (*Dermochelys coriacea*) on Sandy Point, St. Croix, U.S. Virgin Islands, 2005. Annual Report to Fish and Wildlife Service
Garner JA, Garner SA, Coles W (2005) Tagging and nesting research of leatherback sea turtles (*Dermochelys coriacea*) on Sandy Point St. Croix, U.S. Virgin Islands, 2005. WIMARCS, St Croix 58 pp
Garner JA, Garner SA, Coles W (2006) Tagging and nesting research of leatherback sea turtles (*Dermochelys coriacea*) on Sandy Point St. Croix, U.S. Virgin Islands, 2006. WIMARCS, St Croix 52 pp
Garnier J, Silva I (2008) Hawksbill nesting in Mozambique. In: Mast RB, Bailey LM, Hutchinson BH (eds) SWoT Report—The State of the World's Sea Turtles, Volume III, Washington, DC. 43p. Available online *at*: http://seaturtlestatus.org/report/view
Garnier S, Silva I (2007) Cabo Delgado Biodiversity and Tourism Project. Marine turtle programme: Report of activities 2006 / 2007:30 p.
Gerand GD (2008) Personal Communication. Nature Seychelles, unpublished data.
Gilman E, Kobayashi D, Swenarton T, Brothers N, Dalzell P, Kinan-Kelly I (2007) Reducing sea turtle interactions in the Hawaii-based longline swordfish fishery. Biological Conservation 139:19-28
Girard A (2008) Personal Communication.
Girard C, Sudre J, Benhamou S, Roos D, Luschi P (2006) Homing in green turtles *Chelonia mydas*: oceanic currents act as a constraint rather than as an information source. Marine Ecology Progress Series 322:281-289
Giri V (2000) A preliminary survey of sea turtles along the coast of Maharashtra and Goa. Kachhapa 3:7-8
Giri V (2006) Sea turtles of Maharashtra and Goa. In: Shanker K, Choudhury BC (eds) Marine Turtles of the Indian Subcontinent. Universities Press, India, Hyderguda, p 147-155
Girondot M, Godfrey MH, Ponge L, Rivalan P (2007) Modeling approaches to quantify leatherback nesting trends in French Guiana and Suriname. Chelonian Conservation and Biology 6:37-46
Gladstone N (2007) Personal communication. Loggerhead nesting in Madagascar. In: Mast RB, Bailey LM, Hutchinson BH (eds) SWoT Report—The State of the World's Sea Turtles, Volume II, Washington, DC. 49p. Available online *at*: http://seaturtlestatus.org/report/view
Gladstone N, Andriantahina F, Soafiavy B (2003) Azafady Project Fanomena Final Report. Unpublished manuscript. .
Godfrey M (2008) Personal Communication. North Carolina Wildlife Resources Commission.
Godfrey M, North Carolina Wildlife Resources Commission (2006) Personal communication. Leatherback nesting in North Carolina. In: Mast RB, Bailey LM, Hutchinson BH (eds) SWoT Report—The State of the World's Sea Turtles, Volume II, Washington, DC. 49p. Available online *at*: http://seaturtlestatus.org/report/view
Godfrey M, North Carolina Wildlife Resources Commission (2006) Personal communication. Leatherback nesting in North Carolina. In: Mast RB, Bailey LM, Hutchinson BH (eds) SWoT Report—The State of the World's Sea Turtles, Volume I, Washington, DC. 36 p. Available online *at*: http://seaturtlestatus.org/report/view
Godfrey M, North Carolina Wildlife Resources Commission (2007) Personal communication. Loggerhead nesting in North Carolina. In: Mast RB, Bailey LM, Hutchinson BH (eds) SWoT Report—The State of the World's Sea Turtles, Volume II, Washington, DC. 49p. Available online *at*: http://seaturtlestatus.org/report/view
Godgenger M-C, Breheret N, Bal G, N'Damite K, Girard A, Girondot M (2009) Nesting estimation and analysis of threats for Critically Endangered Leatherback *Dermochelys coriacea* and Endangered Olive Ridley *Lepidochelys olivacea* marine turtles nesting in Congo. Oryx 43:1-8
Godgenger M-C, Gibudi A, Girondot M (2008) Activités de ponte des tortues marines

sur l'Ouest africain. Rapport d'étude pour Protomac, Gabon. Orsay, France, Université Paris Sud, AgroParisTech, CNRS et Protomac. 700 p.
Godley BJ, Almeida, Barbosa C, Broderick AC, Catry PX, C. Hays GC, Indjai B (2003) Using Satellite Telemetry to Determine Post-Nesting Migratory Corridors and Foraging Grounds of Green Turtles Nesting at Poilão, Guinea Bissau. CMS, FIBA, People's trust for endangered species:26
Godley BJ, Broderick AC, Campbell LM, Ranger S, Richardson PB (2004) An Assessment of the Status and Exploitation of Marine Turtles in Montserrat
Godley BJ, Broderick AC, Campbell LM, Ranger S, Richardson PB (2004) An assessment of the status and exploitation of matine turtles in Anguilla, Department of Environment, Food and Rural Affairs and the Commonwealth Office, final project report
Godley BJ, Broderick AC, Frauenstein R, Glen F, Hays G (2002) Reproductive seasonality and sexual dimorphism in green turtles. Marine Ecology Progress Series 226:125-133
Godley BJ, Broderick AC, Hays G (2001) Nesting of green turtles (*Chelonia mydas*) at Ascension Island, South Atlantic. Biological Conservation 97:151-158
Godley BJ, Castro B, Michael B, Annette CB, Paulo C, Michael SC, Angela F, Graeme CH, Matthew JW (2010) Unravelling migratory connectivity in marine turtles using multiple methods. Journal of Applied Ecology 47:769-778
Godley BJ, Lima EHSM, Akesson S, Broderick AC, Glen F, Godfrey MH, Luschi P, Hays GC (2003) Movement patterns of green turtles in Brazilian coastal waters described by satellite tracking and flipper tagging. Marine Ecology Progress Series 253:279-288
Godley BJ, Richardson S, Broderick AC, Coyne MS, Glen F, Hays GC (2002) Long-term satellite telemetry of the movements and habitat utilisation by green turtles in the Mediterranean. Ecography 25:352-362
Goitom M (2008) Unpublished data. Ministry of Fisheries
Goitom M, Teclemariam Y, Mengstu T (2006) Field Trip Report on Sea Turtle Nesting Assessment on the Islands of Mojeidi and Aucan Massawa, Eritrea. Ministry of Fisheries Unpublished report
Gómez GL, Sarti L (2004) Protección y conservación de la tortuga laúd (*Dermochelys coriacea*) en Barra de la Cruz, Oaxaca, temporada 2003-2004: Informe final. In: Sarti ML, Barragán RAR, Juárez C JA (eds) Conservación y evaluación de la población de tortuga laúd *Dermochelys coriacea* en el Pacífico Mexicano, temporada de anidación 2003-2004. DGVS-SEMARNAT-Kutzari, Asociación para el Estudio y Conservación de las Tortugas Marinas A.C.
Gómez J (2006) Leatherback nesting in Cote d'Ivoire. In: Mast RB, Bailey LM, Hutchinson BH (eds) SWoT Report—The State of the World's Sea Turtles, Volume I, Washington, DC. 36 p. Available online *at*: http://seaturtlestatus.org/report/view
Gómez J (2007) Projet de conservation de tortues marines en Côte d'Ivoire. In: Mast RB, Bailey LM, Hutchinson BH (eds) SWoT Report—The State of the World's Sea Turtles, Volume II, Washington, DC. 49p. Available online *at*: http://seaturtlestatus.org/report/view
Gomez JB, Sory B, Mamadou K (2003) A preliminary survey of sea turtles in the Ivory Coast. . In: Seminoff JA (ed) Proceedings of the Twenty-second Annual Symposium on Sea Turtle Biology and Conservation. U.S. Dep. Commer. NOAA Tech. Memo. NMFS-SEFSC-503, Miami, Florida. 308 p, p 146
González E, Pinal R (2004) Informe final del programa de inestigación y protección de la tortuga marina, y educación ambiental en el estado de Baja California Sur. Temporada 2003-2004. ASUPMATOMA, AC
González PGA (2003) Proyecto de Protección y Conservación de las Tortugas Marinas. H Ayunta-miento Municipal de Tuxpan, Ver Dirección de Ecología Tuxpan, Veracruz Enero de 2003 Playas de Chile Frío, Benito Juárez y Emiliano Zapata 2001-2004 Asesor Inédito
González PGA (2005) Resultados parciales de la temporada de anidaciòn de las tortugas marinas del período de abril a julio de 2005 en el Centro de Protección y Conservación de las Tortugas marinas en la playa de ejido Barra de Galindo, Tuxpan, Veracruz, Facultad de Ciencias Biológicas y Agropecuarias.

Universidad Veracruzana, 7 p. Inédito
Gordillo GM (2006) Campaña de protección y conservación de las tortugas marinas en Veracruz. Coordinación de apoyos y resultados I Reunión de Responsables de Centros y/o playas de Protección y Conservación de las tortugas Marinas. Consejo Estatal de Protección al Ambiente de Gobierno del Estado de Veracruz. Ayuntamiento de Tamiahua. Organizado por la CONANP-PNSAV. 25 de noviembre de 2006, Sala Multimedia del Ayuntamiento de Boca del río, Veracruz
Gore S, Hastings M, Pickering A, Frett G (2006) Leatherback Nesting in the British Virgin Islands. In: Mast RB, Bailey LM, Hutchinson BH (eds) SWoT Report—The State of the World's Sea Turtles, Volume I, Washington, DC. 36 p. Available online *at*: http://seaturtlestatus.org/report/view
Gore S, Hastings M, Pickering A, Frett G (2007) Leatherback Nesting in the British Virgin Islands. In: Mast RB, Bailey LM, Hutchinson BH (eds) SWoT Report—The State of the World's Sea Turtles, Volume II, Washington, DC. 49p. Available online *at*: http://seaturtlestatus.org/report/view
Government of Western Australia: Department of Environment and Conservation Marine Turtles in Western Australia. Online *at*: http://wwwcalmwagovau/science/turtleshtml
Goverse E (2003) Aerial Survey of the Coastline of Eastern Suriname and Nesting Beach Characteristics. As Part of the Sea Turtles of Suriname 2002 Project. Guianas Forests and Environmental Conservation Project (CFECP) Technical report World Wildlife Fund Guianas/Biotopic Foundation Amsterdam, the Netherlands 21 p available online *at*: http://wwwseaturtleorg/PDF/Goverse_2003_Biotopicpdf
Gow GF (1981) Herpetofauna of Groote Eylandt, Northern Territory. Australian Journal of Herpetology 1:62-70
Gray J (1990a) Successful Hatchlings Solve the Mystery. Crittertalk: Newsletter of Bermuda Aquarium Museum and Zoo 13
Gray J (1990b) Turtles Hatch at Clearwater. Crittertalk: Newsletter of Bermuda Aquarium Museum and Zoo 13
Gray J (2007) Rare Nesting of a Loggerhead in Bermuda Brings Anticipation for the Future. In prep
Gray JA, Mitchell WH, Ward JA, Frick HCI, Meylan PA, Meylan AB (1998) The Bermuda Turtle Project: Studies of immature green turtles at an oceanic feeding ground, 1968-1997. In: Epperly S, Braun J (eds) Proceedings of the seventeenth annual sea turtle symposium. U.S. Dep. Commer. NOAA Tech. Memo. NMFS-SEFSC-415, Orlando, Florida. 342 p., p 59
Great Barrier Reef Marine Park Authority (2006) Loggerhead Turtles - *Caretta caretta*. Online *at*: http://wwwgbrmpagovau/corp_site/key_issues/conservation/natural_values/threatened_species/marine_turtles/loggerhead_turtles
Green D (1984) Long-Distance Movements of Galapagos Green Turtles. Journal of Herpetology 18:121-130
Green D (1998) Basking in Galapagos green turtles. In: Epperly S, Braun J (eds) Proceedings of the seventeenth annual sea turtle symposium. U.S. Dep. Commer. NOAA Tech. Memo. NMFS-SEFSC-415, Orlando, Florida. 342 p.
Green D (2002) Movements of green turtles within and without the Galapagos Archipelago, Ecuador. In: Seminoff JA (ed) Proceedings of the Twenty-Second Annual Symposium on SeaTurtle Biology and Conservation. U.S. Dep. Commer. NOAA Tech. Memo. NMFS-SEFSC-503, Miami, Florida. 308 p, p 74
Greiner EC, Gillette LW, Jacobson ER (1998) Helminths in green turtles (Chelonia mydas) from Florida. In: Epperly S, Braun J (eds) Proceedings of the seventeenth annual sea turtle symposium. U.S. Dep. Commer. NOAA Tech. Memo. NMFS-SEFSC-415, Orlando, Florida. 342 p.
Griffin D, South Carolina Department of Natural Resources (2007) Personal communication. Loggerhead nesting in South Carolina. In: Mast RB, Bailey LM, Hutchinson BH (eds) SWoT Report—The State of the World's Sea Turtles, Volume II, Washington, DC. 49p. Available online *at*: http://seaturtlestatus.org/report/view
Grossman A (2001) Biologia reprodutiva de Chelonia mydas (Reptilia), na Reserva

Biológica do Atol das Rocas. M.Sc. Thesis. Pontifícia Universidade Católica do Rio Grande do Sul

Grossman A, Bellini C, Marcovaldi MA (2002) Reproductive biology of the green turtle at the biological reserve of Atol Das Rocas off northeast Brazil. In: Seminoff JA (ed) Proceedings of the Twenty-Second Annual Symposium on SeaTurtle Biology and Conservation. U.S. Dep. Commer. NOAA Tech. Memo. NMFS-SEFSC-503, Miami, Florida. 308 p, p 136

Guada H (2007) Personal communication. Loggerhead nesting in Venezuela. In: Mast RB, Bailey LM, Hutchinson BH (eds) SWoT Report—The State of the World's Sea Turtles, Volume II, Washington, DC. 49p. Available online *at*: http://seaturtlestatus.org/report/view

Guada H (2008) Personal communication. Hawksbill nesting in Venezuela. In: Mast RB, Bailey LM, Hutchinson BH (eds) SWoT Report—The State of the World's Sea Turtles, Volume III, Washington, DC. 43p. Available online *at*: http://seaturtlestatus.org/report/view

Guada HJ (2000) Areas de anidación e impactos hacia las tortugas marinas en la Península de Paria y lineamientos de protección. M.Sc. thesis. Universidad Simón Bolívar

Guada HJ, (ed.) (2004) Status of the Leatherback Turtle in Venezuela. National Analysis CICTMAR-WIDECAST

Guada HJ, S. GS (2000) Plan de Accion para la Recuperacion de las Tortugas Marinas de Venezuela, 112 p

Guada HJ, Solé G (2000) WIDECAST Plan de Acción para la Recuperación de las Tortugas Marinas de Venezuela. Informe Técnico del PAC No 39 Kingston, Jamaica:United Nations Environment Programme Caribbean Environment Programme

Guinea GF (1990) Notes on sea turtle rookeries on the Arafura Sea Islands of Arnhem Land, Northern Territory. Northern Territory Naturalist 12:4-12

Guinea M (2009) Personal communication. Flatback nesting at Bare Sand Island, Northern Territory. In: Mast RB, Hutchinson BJ, Villegas PE, Wallace B, Yarnell L (eds) SWoT Report—The State of the World's Sea Turtles, Volume IV, Washington, DC. 49 p. Available online *at*: http://seaturtlestatus.org/report/view

Gumbs J (2006) Personal communication. Leatherback nesting in Anguilla. In: Mast RB, Bailey LM, Hutchinson BH (eds) SWoT Report—The State of the World's Sea Turtles, Volume II, Washington, DC. 49p. Available online *at*: http://seaturtlestatus.org/report/view

Gumbs J (2006) Personal communication. Leatherback nesting in Anguilla. In: Mast RB, Bailey LM, Hutchinson BH (eds) SWoT Report—The State of the World's Sea Turtles, Volume I, Washington, DC. 36 p. Available online *at*: http://seaturtlestatus.org/report/view

Gumbs J (2008) Personal communication. Hawksbill nesting in Anguilla. In: Mast RB, Bailey LM, Hutchinson BH (eds) SWoT Report—The State of the World's Sea Turtles, Volume III, Washington, DC. 43p. Available online *at*: http://seaturtlestatus.org/report/view

Gumbs J (2009) Personal communication. Hawksbill nesting in Anguilla. In: Mast RB, Hutchinson BJ, Villegas PE, Wallace B, Yarnell L (eds) SWoT Report—The State of the World's Sea Turtles, Volume IV, Washington, DC. 49 p. Available online *at*: http://seaturtlestatus.org/report/view

Gutiérrez IA, López M (2006) Leatherback nesting in Pacuare, Costa Rica. In: Mast RB, Bailey LM, Hutchinson BH (eds) SWoT Report—The State of the World's Sea Turtles, Volume I, Washington, DC. 36 p. Available online *at*: http://seaturtlestatus.org/report/view

Guzman V (2006) Direccion general de manejo para la conservacion

Guzman-Hernandez V (2006) Direccion general de manejo para la conservacion. Informe tecnico final del prgrama de conservacion de torturgas marinas de Campeche, Mexico en 2005. Unpublished data

Guzman-Hernandez V, Cuevas-Flores EA, Marquez-M. R (2007) Occurrence of Kemp's ridley (*Lepidochelys kempii*) along the coast of the Yucatan Peninsula, Mexico. Chelonian Conservation and Biology 6:274-277

Gyuris E, Limpus CJ (1988) The loggerhead turtle, *Caretta caretta*, in Queensland:

Population Breeding Structure. Australian Wildlife Research 15:197-209
Hadjichristophorou M, Demetropoulos A (2006) Cyprus Turtle Conservation Project Report. Department of Fisheries and Marine Research internal report
Hadjichristophorou M, Demetropoulos A (2007) Cyprus Turtle Conservation Project Report, Department of Fisheries and Marine Research. In: Mast RB, Bailey LM, Hutchinson BH (eds) SWoT Report—The State of the World's Sea Turtles, Volume II, Washington, DC. 49p. Available online *at*: http://seaturtlestatus.org/report/view
Hadjichristophorou M, Demetropoulos A (2007) Cyprus Turtle Conservation Project Report. Department of Fisheries and Marine Research internal report
Hahn AT, Castilhos JC, Soares LS, Bowen BW, Bonatto S-L (in review) Population structure of Olive Ridley sea turtle (*Lepidochelys olivacea*) in the Atlantic Ocean using microsatellites markers.
Hamann M (2009) Personal communication. Flatback nesting in the Gulf of Carpentaria and Torres Strait. In: Mast RB, Hutchinson BJ, Villegas PE, Wallace B, Yarnell L (eds) SWoT Report—The State of the World's Sea Turtles, Volume IV, Washington, DC. 49 p. Available online *at*: http://seaturtlestatus.org/report/view
Hamann M, Cuong CT, Hong ND, Thuoc P, Thuhien BT (2006) Distribution and abundance of marine turtles in the Socilaist Republic of Viet Nam. Biodiversity and Conservation 15:3703-3720
Hamann M, Limpus C, Hughes G, Mortimer J, Pilcher N (2006) Assessment of the conservation status of the leatherback turtle in the Indian Ocean and South-East Asia. IOSEA Marine Turtle MoU Secretariat, Bangkok.
Hamann M, The Cuong C, Duy Hong N, Thuoc P, Thi Thuhien B (2006) Distribution and abundance of marine turtles in the Socialist Republic of Viet Nam. Biodiversity and Conservation 15:3703-3720
Hamman M, Limpus CJ, Hughes GR, Mortimer JA, Pilcher NJ (2006) Assessment of the conservation status of the leatherback turtle in the Indian Ocean and South-East Asia. p.1-166.
Hamza A, Elghmati H (2005) Conservation of marine turtles nesting at three sites West of Sirte, Libya. The Environment General Authority (EGA), Marine Biology Research Center (MBRC) and the Regional Activity Centre for Specially Protected Areas (UNEP-MAP RAC/SPA) Technical Report
Hamza A, Mohamed K, Abdulkarim S, Abdallah I, Hamad M, Bourass E (2008) Loggerhead Nesting Activity at Ain Al Ghazala and Boulfraies beaches in 2007, NE Libya 3rd Mediterranean Conference on Marine Turtles, Tunisia. 30 p
Hamza A, Swayeb B, Elhalloub R, Beki S, Alimal A, Saied A (2008) Loggerhead Marine turtle *caretta caretta* Nesting Activity and Conservation in North West Gulf of Sirte 3rd Mediterranean Conference on Marine Turtles, Tunisia. 24 p
Harker T (2006) Status and Conservation of Sea Turtles in Jamaica. Unpublished report
Harrison E (2006) St Eustatius Sea Turtle Conservation Programme Annual Report 2005. St Eustatius National Parks Foundation. In: Mast RB, Bailey LM, Hutchinson BH (eds) SWoT Report—The State of the World's Sea Turtles, Volume II, Washington, DC. 49p. Available online *at*: http://seaturtlestatus.org/report/view
Harrison E (2008) Personal communication.
Hasbun CR, Vasquez M (1999) Sea Turtles of El Salvador. Marine Turtle Newsletter 85:7-9
Hatase H, Kinoshita M, Bando T, Kamezaki N, Sato K, Matsuzawa Y, Goto K, Omita K, Nakashima Y, Takeshita H, Sakamoto W (2002) Population structure of loggerhead turtles, *Caretta caretta*, nesting in Japan: bottlenecks on the Pacific population. Marine Biology 141:299-305
Hatase H, Sato K, Yamaguchi M, Takahashi K, Tsukamoto K (2006) Individual variation in feeding habitat use by adult female green sea turtles (*Chelonia mydas*): are they obligately neritic herbivores? Oecologia 149:52-64
Hawkes LA, Broderick AC, Coyne MS, Godfrey MH, Jurado-Lopez L-P, Lopez-Suarez P, Merino SE, Varo-Cruz N, Godley BJ (2006) Phenotypically Linked Dichotomy in Sea Turtle Foraging Requires Multiple Conservation Approaches. Current

Biology 16:990-995
Haynes-Sutton A, Kerr-Bjorkland R, Donaldson A, Hamilton M (2005) DRAFT Sea Turtle Recovery Action Plan for Jamaica, UNEP Caribbean Environment Programme, Kingston, Jamaica
Hays Brown C, Brown WM (1982) Status of sea turtles in the southeastern Pacific: Emphasis on Peru. In: Bjorndal KA (ed) Biology and conservation of sea turtles. Smithsonian Institution Press, Washington DC, p 235-240
Hays G, Akesson S, Broderick AC, Glen F, Godley AC, Papi F, Luschi P (2003) Island-Finding Ability of Marine Turtles. Proceedings of the Royal Society of London B (Supplement) 270:S5-S7
Hays G, Broderick AC, Glen F, GOdley BJ, Nicols WJ (2001) The movements and submergence behaviour of male green turtles at Ascension Island. Marine Biology 139:395-399
Hays GC, Akesson S, Broderick AC, Glen F, Godley AC, Luschi P, Martin C, Metcalfe J, Papi F (2001) The diving behaviour of green turtles undertaking oceanic migration to and from Ascension Island: dive durations, dive profiles and depth distribution. The Journal of Experimental Biology 204:4093-4098
Hays GC, Broderick AC, Godley BJ, Lovell P, Martin C, McConnell BJ, Richardson S (2002) Biphasal long-distance migration in green turtles. Animal Behaviour 64:895-898
Hays GC, Dray M, Quaife T, Smyth TJ, Mironnet NC, Luschi P, Papi F, Barnsley MJ (2001) Movements of migrating green turtles in relation to AVHRR derived sea surface temperature. International Journal of Remote Sensing 22:1403-1411
Hays GC, Houghton JDR, Myers AE (2004) Pan-Atlantic leatherback turtle movements Nature 429:522
Hays GC, Luschi P, Papi F, del Seppia C, Marsh R (1999) Changes in behaviour during the inter-nesting period and post-nesting migration for Ascension Island green turtles. Marine Ecology Progress Series 189:263-273
Hearn GW, Rader H, Bradsby JL (2006) Leatherback nesting in Bioko Island, Equatorial Guinea. In: Mast RB, Bailey LM, Hutchinson BH (eds) SWoT Report —The State of the World's Sea Turtles, Volume I, Washington, DC. 36 p. Available online *at*: http://seaturtlestatus.org/report/view
Hernández MR (2005) Reporte de anidaciòn de tortugas marinas en playas de Chachalacas, Municipio de Ursula Galván, Ver. Ayuntamiento de Ursulo Galván. Ver. Ayuntamiento de Ursulo Galván. Regiduría Primera. Inédito.
Hernández R, Buitrago J, Guada H (2006) Personal communication. Leatherback nesting in Venezuela. In: Mast RB, Bailey LM, Hutchinson BH (eds) SWoT Report—The State of the World's Sea Turtles, Volume I, Washington, DC. 36 p. Available online *at*: http://seaturtlestatus.org/report/view
Hernandez R, Buitrago J, Guada H, Hernandez-Hamon H, Llano M (2007) Nesting Distribution and Hatching Success of the Leatherback, Dermochelys coriacea, in Relation to Human Pressures at Playa Parguito, Margarita Island, Venezuela. Chelonian Conservation and Biology 6:79-86
Herrera A, Harrison E (2007) St. Eustatius Sea Turtle Conservation Programme: Annual Report 2006.
Hewavisenthi S (1990) Exploitation of Marine Turtles in Sri Lanka: Historic Background and the Present Status. Marine Turtle Newsletter 48:14-19
Hien TM (2002) Status of Sea Turtle Conservation in Vietnam. In: Kinan I (ed) Proceedings of the Western Pacific Sea Turtle Cooperative Research and Management Workshop. Western Pacific Regional Fishery Management Council, Honolulu, Hawaii, USA. 290 p., p 191-194
Hilterman ML, Goverse E (2005) Annual Report on the 2004 Leatherback Turtle Research and Monitoring Project in Suriname. World Wildlife Fund - Guianas Forests and Environmental Conservation Project (WWF-GFECP) Technical Report of the Netherlands Committee for IUCN (NC-IUCN), Amsterdam, the Netherlands:18 p
Hilterman ML, Goverse E (2005) Annual Report on the 2004 Leatherback Turtle Research and Monitoring Project in Suriname. World Wildlife Fund - Guianas Forests and Environmental Conservation Project (WWF-GFECP) Technical Report of the Netherlands.

Hilterman ML, Goverse E (2007) Nesting and nest success of the leatherback turtle (*Dermochelys coriacea*) in Suriname, 1999-2005. Chelonian Conservation and Biology 6:87-100
Hilterman ML, Goverse E, Tordoir MT, Reichart HA (2008) Beaches come and beaches go: Coastal dynamics in Suriname are affecting important sea turtle rookeries. In: Kalb H, Rohde AS, Gayheart K, Shanker K (eds) Proceedings of the Twenty-Fifth Annual Symposium on Sea Turtle Biology and Conservation. NOAA Technical Memorandum NMFS-SEFSC-582, Savannah, Georgia. 204 p, p 140-141
Hilterman ML, Goverse. E (2006) Annual Report on the 2005 Leatherback Turtle Research and Monitoring Project in Suriname. World Wildlife Fund - Guianas Forests and Environmental Conservation Project (WWF-GFECP) Technical Report of the Netherlands Committee for IUCN (NC-IUCN), Amsterdam, the Netherlands Technical Report of the IUCN Netherlands
Hirama S, Ehrhart LM (2002) Prevalence of green turtle Fibropapillomatosis in three developmental habitats on the east coast of Florida. In: Seminoff JA (ed) Proceedings of the Twenty-Second Annual Symposium on SeaTurtle Biology and Conservation. U.S. Dep. Commer. NOAA Tech. Memo. NMFS-SEFSC-503, Miami, Florida. 308 p, p 302
Hirth HF (1980) Some Aspects of the Nesting Behavior and Reproductive Biology of Sea Turtles American Zoologist. Oxford University Press, p 507-523
Hitipeuw C, Dutton PH, Benson S, Thebu J, Bakarbessy J (2007) Population status and internesting movement of leatherback turtles, *Dermochelys coriacea*, nesting on the northwest coast of Papua, Indonesia. Chelonian Conservation and Biology 6:28-36
Hitipeuw C, WWF Indonesia (2006) Personal communication. Leatherback nesting in Papua Indonesia. In: Mast RB, Bailey LM, Hutchinson BH (eds) SWoT Report—The State of the World's Sea Turtles, Volume I, Washington, DC. 36 p. Available online *at*: http://seaturtlestatus.org/report/view
Hitipeuw C, WWF Indonesia (2007) Personal communication. Leatherback nesting in Papua, Indonesia. In: Mast RB, Bailey LM, Hutchinson BH (eds) SWoT Report—The State of the World's Sea Turtles, Volume II, Washington, DC. 49p. Available online *at*: http://seaturtlestatus.org/report/view
Hoekert WEJ, Neufeglise H, Schouten AD, Menken SBJ (2002) Multiple paternity and female-biased mutation at a microsatellite locus in the olive ridley sea turtle (*Lepidochelys olivacea*). Heredity 89:107-113
Hoekert WEJ, Schouten AD, Van Tienen LHG, Weijerman M (1996) Is the Surinam Olive Ridley on the Eve of Extinction? First Census Data for Olive Ridleys, Green Turtles and Leatherbacks Since 1989. Marine Turtle Newsletter 75:1-4
Hoffman W, Fritts TH (1982) Sea Turtle Distribution along the Boundary of the Gulf Stream Current off Eastern Florida. Herpetologica 38:405-409
Hoinsoude GS, Bowessidjaou JE, Kokouvi GA, Iroko F, Fretey J (2002) Plan for sea turtle conservation in Togo. In: Seminoff JA (ed) Proceedings of the Twenty-second Symposium on Sea Turtle Biology and Conservation. NOAA Technical Memorandum NMFS-SEFSC-503, Miami, Florida, 307 pp., p 117
Honarvar S, van den Berghe EP (2008) Monitoring olive ridley turtles (*Lepidochelys olivaeacea*) on the Pacific coast of Nicaragua. In: Kalb H, Rohde AS, Gayheart K, Shanker K (eds) Proceedings of the Twenty-Fifth Annual Symposium on Sea Turtle Biology and Conservation. NOAA Technical Memorandum NMFS-SEFSC-582, Savannah, Georgia. 204 p, p 125
Hope R (2000) Egg harvesting of the olive ridley marine turtle (*Lepidochelys olivacea*) along the Pacific Coast of Nicaragua and Costa Rica an arribada sustainability analysis. MA Thesis. University of Manchester
Hope R, Smit N (1998) Marine turtle monitoring in Gurig National Park and Coburg Marine Park. In: Webb K, Duff A, Guinea M, Hill G (eds) Proceedings of the Marine Turtle Conservation and Management in Northern Australia Workshop. Centre for Indigenous Natural and Cultural Resource Management and Centre for Tropical Wetlands Management, Northern Territory University, Darwin, Northern Territory
Hopkins-Murphy SR, Murphy TM, Hope CP, Coker JW, Hoyle ME (1999) Population

trends and nesting distribution of the loggerhead turtle (*Caretta caretta*) in South Carolina, 1980-1997. Final completion report to the US Fish and Wildlife

Horrocks J, Krueger B, Beggs J (2008) Hawksbill nesting in Barbados. In: Mast RB, Bailey LM, Hutchinson BH (eds) SWoT Report—The State of the World's Sea Turtles, Volume III, Washington, DC. 43p. Available online *at*: http://seaturtlestatus.org/report/view

Horrocks J, Krueger B, Harewood A (2008) unpublished data.

Horrocks JA (1992) Sea Turtle Recovery Action Plan for Barbados, United National Environment Programme, Kingston, Jamaica. 61 p

Horta H (2004) Leatherback nesting surveys, 2004. Puerto Rico Natural Resources Department (DRNA) Internal report:3 p

Horta H, al. e (2005) Reporte de actividades de conservacion de tortugas marinas en el Noreste de Puerto Rico. Puerto Rico Natural Resources Department (DRNA) Internal report

Huerta P, Machuca C (2004) Informe final de investigación de las actividades de conservación desarrolladas en la playa de Mexiquillo durante la temporada 2003–2004. In: Sarti M. L, Barragan RAR, Juárez C. JA (eds) Conservación y evaluación de la población de tortuga laúd *Dermochelys coriacea* en el Pacífico Mexicano, temporada de anidación 2003-2004. DGVS-SEMARNAT-Kutzari, Asociación para el Estudio y Conservación de las Tortugas Marinas A. C

Hughes DA, Richard JD (1974) The Nesting of the Pacific Ridley Turtle *Lepidochelys olivacea* on Playa Nancite, Costa Rica. Marine Biology 24:97-107

Hughes GR (1972) The Olive Ridley Sea-turtle (*Lepidochelys olivacea*) in South-east Africa. Biological Conservation 4:128-134

Hughes GR (1974) The sea turtles of south east Africa. Ph.D. Thesis. University of Natal

Hughes GR (1996) Nesting of leatherback turtle (*Dermochelys coriacea*) in Tongaland, KwaZulu-Natal, South Africa, 1963-1995. Chelonian Conservation and Biology 2:153-158

Hughes GR, Bartholomew W (1998) The Tongaland sea turtle project 1 April 1997 - 31 March 1998. Natal Parks Board Annual Research Report May:1-12 *in* Limpus. 2007. Marine Turtle Interactive Mapping System *online at:* UNEP/CMS www.unep-wcmc.org

Hughes GR, Huntley B, Wearne D (1973) Conservation around the world: sea turtles in Angola. Biological Conservation 5:58-59

Hurtado M Unpublished data.

Hurtado M Pers. comm.

Hutchinson A (2008) Personal communication. Hawksbill nesting on Playa Camaronal, Costa Rica. In: Mast RB, Bailey LM, Hutchinson BH (eds) SWoT Report—The State of the World's Sea Turtles, Volume III, Washington, DC. 43p. Available online *at*: http://seaturtlestatus.org/report/view

Hutchinson BJ (2006-2007) Modern Genetics Reveals Ancient Diversity in the Loggerhead. In: Mast RB, Bailey LM, Hutchinson BH (eds) SWoT Report—The State of the World's Sea Turtles, Volume II, Washington, DC. 49p. Available online *at*: http://seaturtlestatus.org/report/view, p 18-19

Ibarra ME, Angulo J, Espinosa Lopez G, Nodarse Konnorov A, Colectivo de Estudiantes de la Facultad de Biologia (1998) Informe Final 1ra Temporada de Trabajo. Proyecto universitario para el estudio y conservacion de las tortugas marinas en Cuba, Centro de Investigaciones Marinas, Universidad de la Habana, Habana, Cuba

Ibarra ME, Azanza Ricardo J, Hernandez Zulueta J, Espinosa Lopez G, Colectivo de Estudiantes de la Facultad de Biologia (2008) Informe Final de la 11na Temporada de Trabajo. Proyecto universitario para el estudio y conservacion de las tortugas marinas en Cuba, Centro de Investigaciones Marinas, Universidad de la Habana, Habana, Cuba

Ibarra ME, Azanza Ricardo J, Hernandez Zulueta J, Espinosa Lopez G, Diaz-Fernandez R, Colectivo de Estudiantes de la Facultad de Biologia (2006) Informe Final 8va Temporada de Trabajo. Proyecto universitario para el estudio y conservacion de las tortugas marinas en Cuba, Centro de Investigaciones

Marinas, Universidad de la Habana, Habana, Cuba
Ibarra ME, Díaz Fernández R, Azanza J, Díaz R, Espinosa G (2005) Informe Final 8va Temporada de Trabajo Proyecto universitario para el estudio y conservación de las tortugas marinas en Cuba. Centro de Investigaciones Marinas Cuba: Universidad de la Habana
Ibarra ME, Diaz-Fernandez R, Azanza Ricardo J, Espinosa Lopez G, Colectivo de Estudiantes de la Facultad de Biologia (2002) Informe Final 8va Temporada de Trabajo. Proyecto universitario para el estudio y conservacion de las tortugas marinas en Cuba, Centro de Investigaciones Marinas, Universidad de la Habana, Habana, Cuba
Ibarra ME, Diaz-Fernandez R, Espinosa Lopez G, Azanza Ricardo J, Hernandez Orozco F, Colectivo de Estudiantes de la Facultad de Biologia (2001) Informe Final 8va Temporada de Trabajo. Proyecto universitario para el estudio y conservacion de las tortugas marinas en Cuba, Centro de Investigaciones Marinas, Universidad de la Habana, Habana, Cuba
Ibarra ME, Diaz-Fernandez R, Espinosa Lopez G, Azanza Ricardo J, Nodarse Konnorov A, Hernandez Orozco F, Colectivo de Estudiantes de la Facultad de Biologia (1999) Informe Final 8va Temporada de Trabajo. Proyecto universitario para el estudio y conservacion de las tortugas marinas en Cuba, Centro de Investigaciones Marinas, Universidad de la Habana, Habana, Cuba
Ilgaz C, O T, Ozdemir A, Kaska Y, M S (2007) Population decline of loggerhead turtles: two potential scenarios for Fethiye Beach, Turkey. Biodiversity and Conservation 16:1027-1037
Iniciativa Carey del Pacifico Oriental (ICAPO) (2008) Proceedings from the First Workshop of the Hawksbill Turtle in the Eastern Pacific, El Salvador
Instituto de Investigaciones Marinas y Costeras José Benito Vives de Andréis (INVEMAR), Ministerio del Medio Ambiente (MMA) (2003) Distribución de playas de anidación actual y zonas de avistamiento en el caribe colombiano de las tortugas caguama (*Caretta caretta*), verde (*Chelonia mydas*), carey (*Eretmochelys imbricata*) y canal (*Dermochelys coriacea*). Proyecto tortugas marinas del caribe colombiano
Inter-American Tropical Tuna Commission: Working Group on Bycatch (2004) Review of the status of sea turtle stocks in the Eastern Pacific. Document BYC-4-04. 9p.
Ishihara T (2007) Bycatch investigations in coastal Japan. In: Dalzell P (ed) North Pacific loggerhead sea turtle expert workshop. Western Pacific Regional Fisheries Management Council, Honolulu, HI USA, p 21-23
Islam MZ (2001) Notes on the Trade in Marine Turtle Products in Bangladesh. Marine Turtle Newsletter 94:10
Islam MZ (2002) Marine Turtle Nesting at St. Martin's Island, Bangladesh. Marine Turtle Newsletter 96:19-21
Islam MZ (2006) Personal communication. Loggerhead nesting in Bangladesh. In: Mast RB, Bailey LM, Hutchinson BH (eds) SWoT Report—The State of the World's Sea Turtles, Volume II, Washington, DC. 49p. Available online *at*: http://seaturtlestatus.org/report/view
Island Conservation Society of Seychelles (2008) Hawksbill nesting in Aride Island Nature Reserve, Seychelles. In: Mast RB, Bailey LM, Hutchinson BH (eds) SWoT Report—The State of the World's Sea Turtles, Volume III, Washington, DC. 43p. Available online *at*: http://seaturtlestatus.org/report/view
Iwamoto T, Ishii M, Nakashima Y, Takeshita H, Itoh A (1985) Nesting cycles and migration of the loggerhead sea turtle in Miyazaki, Japan. Japanese Journal of Ecology 35:505-511
Jackson JB (2001) What was natural in the coastal oceans? Proceedings of the National Academy of Sciences 98:5411-5418
Jackson JB, Kirby MX, Berger WH, Bjorndal KA, Botsford LW, Bourque BJ, Bradbury RH, Cooke R, Erlandson J, Estes JA, Hughes TP, Kidwell S, Lange CA, Lenihan HS, Pandolfi JM, Peterson CH, Stenbeck RS, Tegner MJ, Warner RR (2001) Historical Overfishing and the Recent Collapse of Coastal Ecosystems
. Science 293:629-638
James M, Eckert S, Myers R (2005b) Migratory and reproductive movements of male leatherback turtles (Dermochelys coriacea). Marine Biology 147:845-853

James M, Ottensmeyer C, Myers R (2005a) Identification of high-use habitat and threats to leatherback sea turtles in northern waters: new directions for conservation. Ecology Letters 8:195-201
James M, Sherrill-Mix S, Martin K, Myers R (2006) Canadian waters provide critical foraging habitat for leatherback sea turles. Biological Conservation 133:347-357
James M, Sherrill-Mix S, Myers R (2007) Population characteristics and seasonal migrations of leatherback sea turtles at high latitudes. Marine Ecology Progress Series 337:245-254
James MC, Myers RA, Ottensmeyer CA (2005c) Behaviour of leatherback sea turtles, Dermochelys coriacea, during the migratory cycle. Proceedings of the Royal Society B: Biological Sciences 272:1547-1555
Jaramillo AP (2005) Mexico First Annual Report for the Inter-American Convention for the Protection and Conservation of Sea Turtles., Tlalpan, Mexico. 22 p
Jaramillo AP (2006) Mexico Second Annual Report for the Inter-American Convention for the Protection and Conservation of Sea Turtles., Tlalpan, Mexico. 20 p
Jensen MP, Abreu-Grobois FA, Frydenberg J, Loeschcke V (2006) Microsatellites provide insight into contrasting mating patterns in arribada vs. non-arribada olive ridley sea turtle rookeries. Molecular Ecology 15:2567-2575
Joliffe K (2009) Personal communication. In: Mast RB, Hutchinson BJ, Villegas PE, Wallace B, Yarnell L (eds) SWoT Report—The State of the World's Sea Turtles, Volume IV, Washington, DC. 49 p. Available online *at*: http://seaturtlestatus.org/report/view
Jolliffe K, Jolliffe SM (2008) Personal communication. Hawksbill nesting on Cousine Island, Seychelles. In: Mast RB, Bailey LM, Hutchinson BH (eds) SWoT Report —The State of the World's Sea Turtles, Volume III, Washington, DC. 43p. Available online *at*: http://seaturtlestatus.org/report/view
Jonsen I, Myers R, James M (2006) Robust hierarchical state-space models reveal diel variation in travel rates of migrating leatherback turtles. Journal of Animal Ecology 75:1046-1057
Jonsen I, Myers R, James M (2007) Identifying leatherback turtle foraging behaviour from satellite telemetry using a switching state-space model. Marine Ecology Progress Series 337:255-264
Jorgensen SJ, Reeb CA, Chapple TK, Anderson S, Perle C, Van Sommeran SR, Fritz-Cope C, Brown AC, Klimley AP, Block BA Philopatry and migration of Pacific white sharks. Proceedings of the Royal Society B: Biological Sciences 277:679-688
Jribi I, Bradai MN, Bouain A (2006) Loggerhead Turtle Nesting Activity in Kuriat Islands, Tunisia: Assessment of Nine Years Monitoring. Marine Turtle Newsletter 112:12-13
Juarez R, Muccio C (1997) Sea turtle conservation in Guatemala. Marine Turtle Newsletter 77:15-17
Juarez-Ceron JA, Sart-Martinez AL, Dutton PH (2003) First study of the green/black turtles of the Revillagigedo Archipelago: a unique nesting stock in the Eastern Pacific. In: Seminoff JA (ed) Proceedings of the Twenty-Second Annual Symposium on Sea Turtle Biology and Conservation. NOAA Technical Memorandum NMFS-SEFSC-503, Miami, Florida. 308 p, p 70
Justiniano M (2004) Leatherback nesting surveys. Internal repor to DNER and tPuerto Rico Natural Resources Department (DRNA):12 p
Kalamandeen M, DeFreitas R, Pritchard P (2007) Aspects of Marine Turtle Nesting in Guyana, 2007. Guianas Forests and Environmental Conservation Project (GFECP) World Wildlife Fund: Technical Report
Kalamandeen M, DeFreitas R, Stewart K, Pritchard P (2006) Aspects of Marine Turtle Nesting in Guyana, 2006. Guianas Forests and Environmental Conservation Project (GFECP) World Wildlife Fund: Technical Report
Kamezaki N, Chaloupka M, Matsuzawa Y, Omuta K, Takeshita H, Goto K (In press) Long-term temporal and geographic trends in nesting abundance of the endangered loggerhead sea turtle in the Japanese Archipelago. Endangered Species Research
Kamezaki N, Matsuzawa Y, Abe O, Asakawa H, Fujii T, Goto K, Hagino S, Hayami M,

Ishii M, Iwamoto T, Kamata T, Kato H, Kodama J, Kondo Y, Miyawaki I, Mizobuchi K, Nakamura Y, Nakashima Y, Naruse H, Omuta K, Samejima M, Suganuma H, Takeshita H, Tanaka T, Toji T, Uematsu M, Yamamoto A, Yamato T, Wakabayashi I (2003) Loggerhead turtles nesting in Japan. In: Bolten AB, Witherington B (eds) Loggerhead Sea Turtles. Smithsonian Institution Press, Washington, DC. 319 p., p 210-217

Kamezaki N, Miyakawa I, Suganuma H, Omuta K, Nakajima Y, Goto K, Sato K, Matsuzawa Y, Samejima M, Ishii M, Iwamoto T (1997) Post-nesting migration of Japanese loggerhead turtle, *Caretta caretta*. Wildlife Conservation Japan 3:29-39

Kapurusinghe T (2006) Status and Conservation of Marine Turtles in Sri Lanka. In: Shanker K, Choudhury BC (eds) Marine Turtles of the Indian Subcontinent. Universities Press, India, Hyderguda, p 174-187

Kar CS, Bhaskar S (1982) Status of sea turtles in the eastern Indian Ocean. In: Bjorndal KA (ed) Biology and conservation of sea turtles. Smithsonian Institution Press, Washington DC, p 365-372

Karl SA (1998) From green to black and back: Taxonomic distinctiveness of the black turtle, Chelonia agassizii. In: Epperly S, Braun J (eds) Proceedings of the seventeenth annual sea turtle symposium. U.S. Dep. Commer. NOAA Tech. Memo. NMFS-SEFSC-415, Orlando, Florida. 342 p.

Karl SA, Bowen BW, Avise JC (1992) Global Population Genetic Structure and Male-Mediated Gene Flow in the Green Turtle (*Chelonia mydas*): RFLP Analyses of Anonymous Nuclear Loci. Genetics 131:163-173

Kaska YR, Downie R, Tippet R, Furness R (1998) Hatching success of green and loggerhead turtle nests at the west coast of northern Cyprus. In: Epperly S, Braun J (eds) Proceedings of the seventeenth annual sea turtle symposium. U.S. Dep. Commer. NOAA Tech. Memo. NMFS-SEFSC-415, Orlando, Florida. 342 p.

Kaska YR, Furness R, Baran I, Senol A (1998) Inter- and intra-clutch temperature variation of loggerhead and green turtle nests in the Mediterranean. In: Epperly S, Braun J (eds) Proceedings of the seventeenth annual sea turtle symposium. U.S. Dep. Commer. NOAA Tech. Memo. NMFS-SEFSC-415, Orlando, Florida. 342 p.

Kaska YR, Sheps JA, Cohen BL, Furness R (1998) Genetic sequence diversity in the mitochondrial DNA control region of the green turtle population of northern Cyprus. In: Epperly S, Braun J (eds) Proceedings of the seventeenth annual sea turtle symposium. U.S. Dep. Commer. NOAA Tech. Memo. NMFS-SEFSC-415, Orlando, Florida. 342 p.

Kasparek M, Godley BJ, Broderick AC (2001) Nesting of the Green Turtle, Chelonia mydas, in the Mediterranean: a review of status and conservation needs. Zoology in the Middle East 24

Kavanaght R (1984) The National Report: Haiti. In: Bacon, et al (eds) Proceedings of the Western Atlantic Sea Turtle Symposium, Volume 3. University of Miami Press, Miami, Florida, San Jose, Costa Rica

Kelez S, Velez-Zuazo X, Angulo F, Manrique C (2009) Olive Ridley Lepidochelys olivacea Nesting in Peru: The Southernmost Records in the Eastern Pacific. Marine Turtle Newsletter 126:5-9

Kelle L (2008) Personal communication. Hawksbill nesting in French Guiana. In: Mast RB, Bailey LM, Hutchinson BH (eds) SWoT Report—The State of the World's Sea Turtles, Volume III, Washington, DC. 43p. Available online *at*: http://seaturtlestatus.org/report/view

Kelle L, Gratiot N, De Thoisy B (2009) Olive ridley turtle Lepidochelys olivacea in French Guiana: back from the brink of regional extirpation? Oryx 43:243-246

Khalil M, Syed H, Aureggi M (2007) Marine turtle monitoring at El Mansouri and El Koliala, 07. Unpublished final report

Khan MS, Asrar FF, Ahmed N (2009) Green Sea Turtle. available online *at*: http://www.wildlifeofpakistan.com/ReptilesofPakistan/greenseaturtle.htm.

Kichler K, Holder MT, Davis SK, Marquez-M. R, Owens DW (1999) Detection of multiple paternity in Kemp's ridley sea turtle with limited sampling. Molecular Ecology 8:819-830

Kinan I, (ed.) (2005) Proceedings of the Second Western Pacific Sea Turtle Cooperative Research and Management Workshop. Volume I: West Pacific Leatherback and Southwest Pacific Hawksbill Sea Turtles. Western Pacific Regional Fishery Management Council, Honolulu, HI, USA. 290 p

King RS, Lloyd CB (2006) Personal communication. Leatherback nesting in Grenada. In: Mast RB, Bailey LM, Hutchinson BH (eds) SWoT Report—The State of the World's Sea Turtles, Volume I, Washington, DC. 36 p. Available online *at*: http://seaturtlestatus.org/report/view

Kinzel M (2002) Home range and habitat analysis of green sea turtles, Chelonia mydas, in the Gulf of Mexico. In: Seminoff JA (ed) Proceedings of the Twenty-Second Annual Symposium on SeaTurtle Biology and Conservation. U.S. Dep. Commer. NOAA Tech. Memo. NMFS-SEFSC-503, Miami, Florida. 308 p, p 289-290

Kisakao K (2005) Community based conservation and monitoring of leatherback turtles at Kamiali Wildlife Management Area performed by Kamiali Integrated Conservation Development Group. Western Pacific Regional Fishery Management Council Final Report

Kisokau KM, Ambio L (2005) The community based conservation and monitoring of leatherback turtles (Dermochelys coriacea) at Kamiali Wlidlife Management Area, Morobe Province, Papua New Guinea. In: Kinan I (ed) Proceedings of the Second Western Pacific Sea Turtle Cooperative Research and Management Workshop Volume I: West Pacific Leatherback and Southwest Pacific Hawksbill Sea Turtles. Western Pacific Regional Fishery Management Council, Honolulu, HI, USA, p 51-58

Klain S, Eberdong J (2007) Palau Marine Turtle Conservation and Monitoring Program, 2005–2006. Unpublished report to the National Oceanic and Atmospheric Administration (NOAA)

Klain S, Eberdong J, Kitalong A, Yalap Y, Mathews E, Eledui A, Morris M, Andrew W, Albis D, Kemesong P (2007) Linking Micronesia and Southeast Asia: Palau Sea Turtle Satellite Tracking and Flipper Tag Returns. Marine Turtle Newsletter 118:9-11

Klein PA, Jacobson ER, Brown D, Schumacher I, Brown T, Moretti R, Herbst LH (1998) Update on long term experimental transmission studies of green turtle fibropapillomatosis (GTFP). In: Epperly S, Braun J (eds) Proceedings of the seventeenth annual sea turtle symposium. U.S. Dep. Commer. NOAA Tech. Memo. NMFS-SEFSC-415, Orlando, Florida. 342 p.

Kobayashi D, Polovina JJ, Parker DM, Kamezaki N, Cheng IJ, Uchida I, Dutton DM, Balazs GH (2008) Pelagic habitat characterization of loggerhead sea turtles, Caretta caretta, in the North Pacific(1997–2006): Insights from satellite tag tracking and remotely sensed data. Journal of Experimental Marine Biology and Ecology 356:96-114

Koch V, Nichols WJ, Peckham H, de la Toba V (2006) Estimates of sea turtle mortality from poaching and bycatch in Bahı´a Magdalena, Baja California Sur, Mexico. Biological Conservation 128:327-334

Kochery T (2000) Indian Fisheries over the past 50 years (Part II) Coastal Industrial Aquaculture. Kachhapa 3:16-19

Koike B (1998) Sea turtle stranding and salvage network- Louisiana Cajun style. In: Byles R, Fernandez Y (eds) Proceedings of the Sixteenth Annual Symposium on Sea Turtle Biology and Conservation. NOAA Technical Memorandum NMFS-SEFSC-412, Hilton Head, South Carolina. 158 p., p 86

Kolinski SP (1995) Migrations of the green turtle, *Chelonia mydas*, breeding in Yap State, Federated States of Micronesia. Micronesica 28:1-8

Kolinski SP, Hoeke RK, Holzwarth SR, Ilo LI, Cox EF, O'Connor RC, Vroom PS (2006) Nearshore Distribution and an Abundance Estimate for Green Sea Turtles, Chelonia mydas, at Rota Island, Commonwealth of the Northern Mariana Islands. Pacific Science 60:509-522

Kotas JE, dos Santos S, Azevedo VG, Gallo BMG, Barata PCR (2004) Incidental capture of loggerhead (*Caretta caretta*) and leatherback (*Dermochelys coriacea*) sea turtles by the pelagic longline fishery off southern Brazil Brazil Fishery Bulletin 102:393-399

Krueger BISRTSotWsST, vol. 3 (2008). (2008) Personal communication. Hawksbill nesting in Papua New Guinea. In: Mast RB, Bailey LM, Hutchinson BH (eds) SWoT Report—The State of the World's Sea Turtles, Volume III, Washington, DC. 43p. Available online *at*: http://seaturtlestatus.org/report/view

Kutle B, Kuzuturk E, Altınkaya H, Sahin I, Demir M, Itaatli H, Ilgaz M, Ilgaz S, Yörükoglu R, Sahin M, Koyluoglu H (2006) Cıralı: An example from community-based conservation of marine turtles. In: Frick HCI, Panagopoulo A, Rees AF, Williams K (eds) Proceedings of the Twenty-sixth Annual Symposium on Sea Turtle Biology and Conservation, Island of Crete, Greece. 376 p

Kutty R (2000) Turtles and Tourists – A coastal village in Goa shows the way. Kachhapa 3:5-7

LaCasella EL, Dutton PH (2007) Longer mtDNA sequences resolve leatherback stock structure. In: Rees AF, Frick MA, Panagopoulou AF, Williams K (eds) Proceedings of the Twenty-Seventh Annual Symposium on Sea Turtle Biology and Conservation. NOAA Technical Memorandum NMFS-SEFSC-569, Mrytle Beach, South Carolina. 261 p., p 155

Lagueux C, Campbell C, 2006.. In The State of the WorldÆs Sea Turtles Report v (2006) Personal communication. Leatherback nesting in Nicaragua. In: Mast RB, Bailey LM, Hutchinson BH (eds) SWoT Report—The State of the World's Sea Turtles, Volume I, Washington, DC. 36 p. Available online *at*: http://seaturtlestatus.org/report/view

Lagueux CJ (1989) Olive Ridley (*Lepidochelys olivacea*) Nesting in the Gulf of Fonseca and the Commercialization of its Eggs in Honduras. M.Sc. Thesis. University of Florida

Lagueux CJ, Campbell CL (2005) Sea turtle nesting and conservation needs on the south-east coast of Nicaragua. Oryx 39:398-405

Lagueux CJ, Campbell CL, Lauck EW (2005) Management strategy for marine turtle conservation on the Caribbean Coast of Nicaragua, Vol, Gainesville, Florida

Lagueux CJ, Campbell CL, McCoy WA (2003) Nesting and conservation of the hawksbill turtle, *Eretmochelys imbricata*, in the Pearl Cays, Nicaragua. Chelonian Conservation and Biology 4:588-602

Lahanas PN, Bjorndal K, Bolten A, Encalada SE, Miyamoto MM, Valverde RA, Bowen BW (1998) Genetic composition of a green turtle (Chelonia mydas) feeding ground population: evidence for multiple origins. Marine Biology 130:345-352

Lahanas PN, Miyamoto MM, Bjorndal KA, Bolten AB (1994) Molecular evolution and population genetics of Greater Caribbean green turtles (*Chelonia mydas*) as inferred from mitochondrial DNA control region sequences. Genetica 94:57-67

Lambardi P, Lutjeharms JRE, Mencacci R, Hays GC, Luschi P (2008) Influence of ocean currents on long-distance movement of leatherback sea turtles in the Southwest Indian Ocean. Marine Ecology Progress Series 353:289-301

Lara-Ruiz P, Lopez GG, Santos FR, Soares LS (2006) Extensive hybridization in hawksbill turtles (Eretmochelys imbricata) nesting in Brazil revealed by mtDNA analyses. Conservation Genetics:773-781

Laurent L, Casale P, Bradai MN, Godley BJ, Gerosas G, Broderick AC, Schroth W, Schierwater B, Levy AM, Freggi D, Abd El-Mawla EM, Hadoud DA, Gomati HE, Domingo M, Hadjichristophorou M, Kornasky L, Demirayak F, Gautier CH (1998) Molecular resolution of marine turtle stock composition in fishery bycatch: a case study in the Mediterranean. Molecular Ecology 7:1529-1542

Laurent L, Lescure J (1994) L'hivernage des tortues caouannes *Caretta caretta* dans le sud Tunisien. Revue d' Ecologie (Terre et Vie) 49:63-85

Laurent-Stepler M, Bourjea J, Roos D, Pelletier D, Ryan P, Ciccione S, Grizel H (2007) Reproductive seasonality and trend of Chelonia mydas in the SW Indian Ocean: a 20 yr study based on track counts. Endangered Species Research 3:217-227

Lazar B, Casale P, Tvrtkovic N, Kozul V, Tutman P, Glavic N (2004) The presence of the green sea turtle, *Chelonia mydas*, in the Adriatic Sea. Herpetological Journal 14 147-147 *in* White, M., I. Haxhiu, et al. (2009). "Monitoring and Conservation of Important Sea Turtle Feeding Grounds in the Patok Area of Albania. 2008 Annual Report." Joint project of: MEDASSET; GEF/SGP; RAC/SPA (UNEP/MAP); Ministry of Environment, Albania; Natural History Museum, Albania; H.A.S., Albania; University of Tirana; ECAT, Albania: 2091 pp.

Lazar B, Margaritoulis D, Tvrtkovic N (2000) Migrations of the loggerhead sea turtle (*Caretta caretta*) into the Adriatic Sea. In: Abreu-Grobois FA, Briseno-Duenas D, Marquez-Millan. R, Sarti-Martinez AL (eds) Proceedings of the eighteenth international sea turtle symposium. NOAA Technical Memorandum NMFS-SEFSC-436, Mazatlán, Sinaloa, México. 293 p., p 101

Le Gall JY, Bosc P, Chateau D, Taquet M (1986) Estimation du nombre de Tortues Vertes femelles adultes *Chelonia mydas* par saison de ponte à Tromelin et Europa (océan indien) (1973–1985). Océanographie Tropicale 21:3-22

Le scao R, Esteban N (2003) 2003 Annual Report: St. Eustatius Sea Turtle Monitoring Programme. Prepared for St Eustatius National Parks Foundation (STENAPA)

Le Scao R, Esteban N (2005) St. Eustatius Sea Turtle Monitoring Programme: Annual Report 2004

León Y (2006) Personal communication. Leatherback nesting in the Dominican Republic. In: Mast RB, Bailey LM, Hutchinson BH (eds) SWoT Report—The State of the World's Sea Turtles, Volume I, Washington, DC. 36 p. Available online *at*: http://seaturtlestatus.org/report/view

León Y (2007) Personal communication. Leatherback nesting in the Dominican Republic. In: Mast RB, Bailey LM, Hutchinson BH (eds) SWoT Report—The State of the World's Sea Turtles, Volume II, Washington, DC. 49p. Available online *at*: http://seaturtlestatus.org/report/view

LeRoux RA, Balazs GH, Dutton PH (2002) Genetic stock composition of foraging green turtles off the southern coast of Molokai, Hawaii. In: Seminoff JA (ed) Proceedings of the Twenty-Second Annual Symposium on SeaTurtle Biology and Conservation. U.S. Dep. Commer. NOAA Tech. Memo. NMFS-SEFSC-503, Miami, Florida. 308 p, p 251-252

Levy Y (2007) Sea turtle nesting activity along the Mediterranean shores of Israel. In: Mast RB, Bailey LM, Hutchinson BH (eds) SWoT Report—The State of the World's Sea Turtles, Volume II, Washington, DC. 49p. Available online *at*: http://seaturtlestatus.org/report/view

Lezama C (2009) Sea Turtle Bycatch in Small-Scale Artisanal Fisheries of Uruguay. Sea Turtle Interactions in Coastal Net Fisheries in Brazil. In: Gilman E (ed) Proceedings of the Technical Workshop on Mitigating Sea Turtle Bycatch in Coastal Net Fisheries, p 27

Liang YL, Dai YR, Liu YQ, Liu SY, Wan XJ, Song ZH, Chen DT, al. e (1990) The investigation of sea turtle resources in the South China Sea and the development of artificial hatching techniques for sea turtles. Report of the South China Sea Turtle Resources Conservation Station, Major Research Project of the Aquaculture Department China: Bureau of Agriculture

Liew H-C, Bali J, Chan E-H, Braken O (2000) Satellite Tracking of Green Turtles from the Sarawak Turtle Islands, Malaysia. Marine Turtle Newsletter 87:20

Liew H-C, Chan E-H, Luschi P, Papi F (1995) Satellite tracking data on Malaysian Green Turtle migration. Rendiconti Lincei 6:239-246

Liew HC (2002) Status of Marine turtle conservation and research in Malaysia. In: Kinan I (ed) Proceedings of the Western Pacific Sea Turtle Cooperative Research and Management Workshop. Western Pacific Regional Fishery Management Council, Honolulu, Hawaii. 290 p., p 51-56

Lima EHSM, Lagueux CJ, Castro DW, Marcovaldi MA (1999) From One Feeding Ground to Another: Green Turtle Migration Between Brazil and Nicaragua. Marine Turtle Newsletter 85:10

Lima EHSM, Troeng S (2001) Link Between Green Turtles Foraging in Brazil and Nesting in Costa Rica? Marine Turtle Newsletter 94

Limpus C (2001) Report to 3rd IOSEA meeting. Manila, Phillipines.

Limpus C (2007a) A biological review for conservation of the flatback turtle, *Natator depressus* (Garman), in Australia *in*. In: A Biological Review of Australian Marine Turtles 5. Queensland Government Environmental Protection Agency. 53 p., Brisbane, Australia. Available online *at*: http://www.derm.qld.gov.au/wildlife-ecosystems/wildlife/caring_for_wildlife/

Limpus C (2007b) Personal communication. Loggerhead nesting in eastern Australia. In: Mast RB, Bailey LM, Hutchinson BH (eds) SWoT Report—The State of the World's Sea Turtles, Volume II, Washington, DC. 49p. Available online *at*: http://

seaturtlestatus.org/report/view
Limpus C, Couper P, Read M (1994) The green turtle, *Chelonia mydas*, in Queensland: population structure in a warm temperate feeding area. Memoirs Queensland Museum 35:139-154
Limpus CJ (1985) A study of the loggerhead turtle, *Caretta caretta*, in eastern Australia. Ph.D. Thesis. University of Queensland
Limpus CJ (1993) The green turtle, Chelonia mydas, in Queensland: breeding males. Wildlife Research **20**, 513–523. *in* Limpus, C. J. (2008). A biological review of Australian Marine turtles. F. M. S. Unit, Queensland Environmental Protection Agency.
Limpus CJ (2002) Personal Comminuction. Cited in Seminoff, J.A., (assessor). 2004. Global Status Assessment: Green turtle (*Chelonia mydas*). Marine Turtle Specialist Group. Species Survival Commission, Red List Programme: 71.
Limpus CJ (2006) Personal communication. Leatherback nesting in Australia. In: Mast RB, Bailey LM, Hutchinson BH (eds) SWoT Report—The State of the World's Sea Turtles, Volume I, Washington, DC. 36 p. Available online *at*: http://seaturtlestatus.org/report/view
Limpus CJ (2007a) A Biological Review of Australian Marine Turtles. 5. Flatback Turtle *Natator depressus* (Garman), The State of Queensland. Environmental Protection Agency
Limpus CJ (2007b) Marine Turtle Interactive Mapping System. *online at:* http://stort.unep-wcmc.org/imaps/indturtles/viewer.htm. United Nations Environment Programme
Limpus CJ (2008a) A biological review of Australian Marine turtles: Green turtle *Chelonia mydas* (Linnaeus), Queensland Environmental Protection Agency
Limpus CJ (2008b) A biological review of Australian Marine Turtles: Loggerhead turtle, *Caretta caretta* (Linnaeus). F.M.S. Unit, Queensland Environmental Protection Agency
Limpus CJ (2008c) A biological review of Australian Marine Turtles: Olive ridley turtle *Lepidochelys olivacea*(Eschscholtz). F.M.S. Unit, Queensland Environmental Protection Agency
Limpus CJ (2009a) A biological review of Australian marine turtles: Hawksbill turtle *Eretmochelys imbricata* (Linnaeus), Queensland Environmental Protection Agency.
Limpus CJ (2009b) A biological review of Australian marine turtles: Leatherback turtle *Dermochelys coriacea* (Vandelli), Queensland Environmental Protection Agency.
Limpus CJ, Boyle M, Sunderland T (2005) New Caledonian loggerhead turtle population assessment: 2005 pilot study. In: Kinan I (ed) Proceedings of the Second Western Pacific Sea Turtle Cooperative Research and Management Workshop Volume II: North Pacific Loggerhead Sea Turtles, Honolulu, HI, USA
Limpus CJ, Chatto R (2004) Marine Turtles. In Description of Key Species Groups in the Northern Planning Area. National Oceans Office Hobart, Australia
Limpus CJ, Environmental Protection Agency (2009) Personal communication. Flatback nesting in Queensland. In: Mast RB, Hutchinson BJ, Villegas PE, Wallace B, Yarnell L (eds) SWoT Report—The State of the World's Sea Turtles, Volume IV, Washington, DC. 49 p. Available online *at*: http://seaturtlestatus.org/report/view
Limpus CJ, Limpus DJ (2003) The biology of the loggerhead turtle in Western South Pacific Ocean foraging areas. In: Bolten AB, Witherington B (eds) Loggerhead Sea Turtles. Smithsonian Institution Press. 319 p., Washington, DC, p 93-113
Limpus CJ, Miller JD (2000) Australian Hawksbill Turtle Population Dynamics Project. Final Report Queensland, Australia: Queensland Parks and Wildlife Service, and the Japan Bekko Association
Limpus CJ, Miller JD (2008d) Australian Hawksbill Turtle Population Dynamics Project. Queensland Parks and WIldlife Service 130 pp
Limpus CJ, Miller JD, Parmenter CJ, Limpus DJ (2003) The green turtle, *Chelonia mydas*, population of Raine Island and the Northern Great Barrier Reef: 1843-2001. Memoirs Queensland Museum 49:349 - 440
Limpus CJ, Miller JD, Parmenter CJ, McLachlan N, Webb R (1992) Migration of green

(*Chelonia mydas*) and loggerhead (Caretta caretta) turtles to and from eastern Australian rookeries. Wildlife Research **19** (347-358) *in* Limpus, C. J. (2008). A biological review of Australian Marine turtles. F. M. S. Unit, Queensland Environmental Protection Agency.

Limpus CJ, Miller JD, Parmenter CJ, Raimer D, McLachlan N, Webb R (1992) Migration of green (*Chelonia mydas*) and loggerhead (*Caretta caretta*) turtles to and from eastern Australian rookeries. Wildlife Research 19:347-358

Limpus CJ, Mortimer JA, Pilcher NJ (2002) The Raine Island green turtle rookery: Y2K update. In: Mosier A, Foley A, Brost B (eds) Proceedings of the Twentieth Annual Symposium on Sea Turtle Biology and Conservation. NOAA Technical Memorandum NMFS-SEFSC-477, Orlando, Florida. 370 pp., p 132-134

Limpus CJ, Parmenter CJ, Baker V, Fleay A (1983) The Crab Island sea turtle rookery in the northeastern Gulf of Carpentaria. Australian Wildlife Research 10:173-184

Limpus CJ, Preece N (1992) One and All Expedition, 11–31 July 1992: Weipa to Darwin via Wellesley Group and the outer islands of Arnhem Land. Unpublished report, Queensland Department of Environment and Heritage, Brisbane:1-41

Livingstone S (2005) Report of Olive ridley (*Lepidochelys olivacea*) nesting on the north coast of Trinidad. Marine Turtle Newsletter 109:6

Livingstone SR (2005) Report of Olive Ridley Nesting on the North Coast of Trinidad. Marine Turtle Newsletter 109:6-7

Livingstone SR (2006) Sea Turtle Ecology and Conservation on the North Coast of Trinidad. Ph.D. Thesis. University of Glasgow

Livingstone SR (2007) Personal communication. Leatherback nesting in Trinidad. In: Mast RB, Bailey LM, Hutchinson BH (eds) SWoT Report—The State of the World's Sea Turtles, Volume II, Washington, DC. 49p. Available online *at*: http://seaturtlestatus.org/report/view

Livingstone SR, Downie LR Unpublished data.

Lloyd C, Ocean Spirits (2008) Personal communication. Hawksbill nesting in Grenada. In: Mast RB, Bailey LM, Hutchinson BH (eds) SWoT Report—The State of the World's Sea Turtles, Volume III, Washington, DC. 43p. Available online *at*: http://seaturtlestatus.org/report/view

Lohmann KJ, Lohmann CMF (1996) Orientation and open-sea navigation in sea turtles. The Journal of Experimental Biology
199:73-81

Lombard P (2006) Marine turtle monitoring and conservation in southern Mozambique. Update 2005 / 2006. p.1-8.

Lombard P (2008) Marine turtle monitoring and conservation in southern Mozambique. Update 2007 / 2008. p.1-7.

Lopez Carcache J, Vega R, Carballo A, Rodriguez M, Cortez B, Mota S, Camacho M, Urteaga J (2008) Monitoring of isolated and arribada nests of olive ridley, *Lepidochelys olivacea*, in Chacocente Beach, Rio Escalante-Chacocente Wildlife Refuge, Pacific coast of Nicaragua (2002-2004) In: Kalb H, Rohde AS, Gayheart K, Shanker K (eds) Proceedings of the Twenty-Fifth Annual Symposium on Sea Turtle Biology and Conservation. NOAA Technical Memorandum NMFS-SEFSC-582, Savannah, Georgia. 204 p, p 135

Lopez E, Arauz R (2003) Nesting records of East Pacific green turtle (*Chelonia mydas agassizii*) in south Pacific Costa Rica, including notes on incidental capture by shrimping and longline activities. In: Seminoff JA (ed) Proceedings of the Twenty-Second Annual Symposium on SeaTurtle Biology and Conservation. NOAA Technical Memorandum NMFS-SEFSC-503, Miami, Florida. 308 p, p 84-85

Lopez Jurado LF, Sanz P, Abella E (2007) Loggerhead nesting on Boa Vista, República de Cabo Verde. In: Mast RB, Bailey LM, Hutchinson BH (eds) SWoT Report—The State of the World's Sea Turtles, Volume II, Washington, DC. 49p. Available online *at*: http://seaturtlestatus.org/report/view

Lopez-Castro MC, Rocha-Olivares A (2005) The panmixia paradigm of eastern Pacific olive ridley turtles revised: consequences for their conservation and evolutionary biology. Molecular Ecology 14:3325-3334

Lopez-Jurado LF, Cabrera I, Cejudo D, Evora C, Alfama P (2000) Distribution of marine turtles in the Archipelago of Cape Verde, Western Africa. In: Kalb H, Wibbels T (eds) Proceedings of the nineteenth annual symposium on sea turtle conservation and biology. NOAA Technical Memorandum NMFS-SEFSC-443, South Padre Island, Texas, U.S.A. 291 p., p 245

Lopez-Mendilaharsu M, Gardner SC, Seminoff JA (2002) Feeding ecology of the East Pacific green turtle (Chelonia mydas agassizii), in Bahía Magdalena, B.C.S. México. In: Seminoff JA (ed) Proceedings of the Twenty-Second Annual Symposium on SeaTurtle Biology and Conservation. U.S. Dep. Commer. NOAA Tech. Memo. NMFS-SEFSC-503, Miami, Florida. 308 p

Lopez-Mendilaharsu M, Sales G, Giffoni B, Miller P, Niemeyer Fiedler F, Domingo A (2007) Distribucion y composicion de tallas de las tortugas marinas (*Caretta caretta* y *Dermochelys coriacea*) que interactuan con el palangre pelagico en el Atlantico Sur. Collective Volume of Scientific Papers, ICCAT 60:2094-2109

Louro CMM, Pereira MAM, Costa ACD (2006) Report on the conservation status of marine turtles in Mozambique. Report submitted to República de Moçambique Ministério para a Coordenação da Acção Ambiental, Maputo 40 p

Lum LL, Lima EHSM, Santos A (1998) Green Turtle Tagged in Brazil Recovered in Trinidad. Marine Turtle Newsletter 82:9

Luschi P, Akesson S, Broderick AC, Glen F, Godley BJ, Papi F, Hays G (2001) Testing the Navigational Abilities of Ocean Migrants: Displacement Experiments on Green Sea Turtles (*Chelonia mydas*). Behavioral Ecology and Sociobiology 50:528-534

Luschi P, Benhamou S, Girard C, Ciccione S, Roos D, Sudre J, Benvenuti S (2007) Marine Turtles Use Geomagnetic Cues during Open-Sea Homing. Current Biology 17:126-133

Luschi P, Hays G, del Seppia C, Marsh R, Papi F (1998) The Navigational Feats of Green Sea Turtles Migrating from Ascension Island Investigated by Satellite Telemetry. Proceedings of the Royal Society of London B 265:2279-2284

Luschi P, Lutjeharms JRE, Lambardi P, Mencacci R, Hughes GR, Hays G (2006) A review of migratory behaviour of sea turtles off southeastern Africa. South African Journal of Science 102:51-58

Luschi P, Papi F, Liew H-C, Chan E-H, Bonadonna F (1996) Long-distance migration and homing after displacement in the green turtle (*Chelonia mydas*): a satellite tracking study. Journal of Comparative Physiology A 178:447-452

Luschi P, Sale A, Mencacci R, Hughes GR, Lutjeharms JRE, Papi F (2003b) Current transport of leatherback sea turtles (*Dermochelys coriacea*) in the ocean. Proceedings of the Royal Society of London B 270:S129-S132

Lutcavage M, Andrews R, Rhodin A, Sadove S, Rehm Conroy C, Horta H (2003) Post-nesting movements of leatherback turtles tracked from Culebra and Fajardo, Puerto Rico with pop-up archival and TDR staellite tags. In: Seminoff JA (ed) Proceedings of the Twenty-Second Annual Symposium on Sea Turtle Biology and Conservation. NOAA Technical Memorandum NMFS-SEFSC-503, Miami, Florida. 308 p, p 58

Macia A (2006) Transboundary networks of marine protected areas in East Africa (Transmap). Periodic activity report Work package 4: Special taxa, Year 1: 70-72

Macias FS (2006) Honduras Second Annual Report *In*, Direction of Biodiversity/ SERNA, unpublished report. 15p

Mackay AL (2005) Sea Turtle Monitoring Program The East End Beaches of St. Croix, U.S. Virgin Islands, 2005. WIMARCS, St Croix 18pp

Mackay AL (2006) Sea Turtle Monitoring Program The East End Beaches of St. Croix, U.S. Virgin Islands, 2006. WIMARCS, St Croix 16 p

Mackay Turtle Watch (2009) Flatback nesting in northern Queensland. In: Mast RB, Hutchinson BJ, Villegas PE, Wallace B, Yarnell L (eds) SWoT Report—The State of the World's Sea Turtles, Volume IV, Washington, DC. 49 p. Available online *at*: http://seaturtlestatus.org/report/view

Maggiani F (2006) AMP Capo Carbonara. Available online at: http:// wwwampcapocarbonarait/eventihtm

Majil I (2005) Belize First Annual Report (unpublished), Marine Protected Areas

Coordinator, Fisheries Department, 9 p
Majil I (2006) Belize Second Annual Report (unpublished), Marine Protected Areas Coordinator, Fisheries Department, 9 p
Majil I, Bacalar Chico Marine Reserve and National Park (2007) Loggerhead nesting in Belize. In: Mast RB, Bailey LM, Hutchinson BH (eds) SWoT Report—The State of the World's Sea Turtles, Volume II, Washington, DC. 49p. Available online *at*: http://seaturtlestatus.org/report/view
Makowski C, Slattery R, Salmon M (2002) "Shark fishing": a technique for estimating the distribution of juvenile green turtles (*Chelonia mydas*) in shallow water developmental habitats, Palm Beach County, Florida USA. In: Seminoff JA (ed) Proceedings of the Twenty-Second Annual Symposium on SeaTurtle Biology and Conservation. U.S. Dep. Commer. NOAA Tech. Memo. NMFS-SEFSC-503, Miami, Florida. 308 p, p 241
Mann MA, Mellgren RL, Arenas A (1998) Comparative development of green (Chelonia mydas) and hawksbill (Eretmochelys imbricata) sea turtles. In: Epperly S, Braun J (eds) Proceedings of the seventeenth annual sea turtle symposium. U.S. Dep. Commer. NOAA Tech. Memo. NMFS-SEFSC-415, Orlando, Florida. 342 p.
Mansfield KL, Reardon R (1998) Dry Tortugas sea turtle monitoring program year two: a season of green turtle (Chelonia mydas) nesting activity. In: Epperly S, Braun J (eds) Proceedings of the seventeenth annual sea turtle symposium. U.S. Dep. Commer. NOAA Tech. Memo. NMFS-SEFSC-415, Orlando, Florida. 342 p.
Manzano CF (2005) Reporte final de la temporada 2005. campamento Vida Milenaria A. C. *In* I Reunión de Responsables de Centros y/o playas de Protección y Conservación de las tortugas Marinas. Organizado por la CONANP-PNSAV, Sala Multimedia del Ayuntamiento de Boca del río, Veracruz. Inédito
Marambio M, Lopez C, Brito J (2007) Nuevo registro de una poblacion de Chelonia mydas residente en un area de alimentacion en la costa de la Region de Atacama, Norte de Chile. In: VII Simposio sobre medio ambiente: Estado Actual y Perspectivas de la Investigacion y Conservacion de las Tortugas Marinas en las Costas del Pacifico Sur Oriental (ed CREA), Antofogasta, Chile:39
MARAPA ONG, PROTOMAC (São Tomé and Príncipe, Central Africa. Rapport des Activités. Unpublished report.)
Marco A, Lopez O, Abella E, Varo N, Marins S, Gaona P, Sanz P, Lopez-Jurado L (2008) Massive capture of nesting females is severely threatening the Cabo Verdian loggerhead population 28th Annual Symposium on Sea Turtle Biology and Conservation, Loreto, Baja California Sur, Mexico
Marcovaldi M (2006) Brazil Second Annual Report to the Inter-American Convention for the Protection and Conservation of Sea Turtles, Centro Tamar-Ibama, Bahia, Brazil. 23 p.
Marcovaldi M, Lopez G, Soares L, Lima E, Thome J, Almeida A (2010) Satellite-tracking of female loggerhead turtles highlights fidelity behavior in northeastern Brazil. Endangered Species Research 12:263-272
Marcovaldi M, Soares L, Bellini C (2008) Hawksbill nesting in Brazil. In: Mast RB, Bailey LM, Hutchinson BH (eds) SWoT Report—The State of the World's Sea Turtles, Volume III, Washington, DC. 43p. Available online *at*: http://seaturtlestatus.org/report/view

Marcovaldi MA (1996) Brazilian sea turtle program - TAMAR/IBAMA: 'Ecotourism and Educational Program -- Praia do Forte, Bahia-Brazil' In: Keinath JA, Barnard DE, Musick JA, Bell BA (eds) Proceedings of the Fifteenth Annual Symposium on Sea Turtle Biology and Conservation. NOAA Technical Memorandum NMFS-SEFSC-387, Hilton Head, South Carolina. 355 p, p 187-188
Marcovaldi MA (2000) Status of olive ridley sea turtle (Lepidochelys olivacea) in the western Atlantic ocean. *In*: Memories of Marine Turtle Conservation in the Wider Caribbean Region - A dialogue for Effective Regional Management. Dominican Republic. September 1999. *in*: Livingstone, S.R. 2005. Report of Olive Ridley Nesting on the North Coast of Trinidad. Marine Turtle Newsletter 109: 6-7.
Marcovaldi MA (2001) Status and Distribution of the Olive Ridley Turtle, Lepidochelys olivacea, in the Western Atlantic Ocean. In: Eckert KL, Grobois FAA (eds)

Marine Turtle Conservation in the Wider Caribbean Region: A Dialogue for Effective Regional Management, Santo Domingo, Domincan Republic, p 52-56
Marcovaldi MA, Chaloupka M (2007) Conservation status of the loggerhead sea turtle in Brazil: an encouraging outlook. Endangered Species Research 3:133-143
Marcovaldi MA, da Silva ACCD, Gallo BM, Baptistotte C, Lima EP, Bellini C, Lima EH, Castilhos JC, Thome J, Moreira L, Sanches TM (2000) Recapture of Tagged Turtles from Nesting and Feeding Grounds Protected by Projecto TAMAR-IBAMA, Brasil. In: Kalb H, Wibbels T (eds) Proceedings of the nineteenth annual symposium on sea turtle conservation and biology. NOAA Technical Memorandum NMFS-SEFSC-443, South Padre Island, Texas, U.S.A. 291 p., p 164-166
Marcovaldi MA, Giffoni B. de B., Becker H, Fiedler FN (2009) Sea Turtle Interactions in Coastal Net Fisheries in Brazil. In: Gilman E (ed) Proceedings of the Technical Workshop on Mitigating Sea Turtle Bycatch in Coastal Net Fisheries, Honolulu, Hawaii. 76 p., p 28
Marcovaldi MA, Lopez E, Marcovaldi MA, Lopez GG, Soares LS, Santos AJB, Bellini C, Barata PCR (2007) Fifteen years of hawksbill sea turtle (*Eretmochelys imbricata*) nesting in Brazil. Chelonian Conservation and Biology 6:223-228
Marcovaldi MA, Lopez GG, Bellini B (2008) *Eretmochelys imbricata* (Linnaeus, 1766). In: Livro vermelho da fauna brasileira ameaçada de extinção Brasília: MMA, v2, Biodiversitas, 19, p 362-363 *in*
Marcovaldi MA, Marcovaldi GG (1999a) Marine turtles of Brazil: the history and structure of Projeto TAMAR-IBAMA. Biological Conservation 91:35-41
Marcovaldi MÂ, Sales G, Thomé JC, da Silva AC, Gallo BM, Lima EH, Lima EP, Bellini C (2006) Sea turtles and fishery interactions in Brazil: identifying and mitigating potential conflicts. Marine Turtle Newsletter 112:4-8
Marcovaldi MA, Thome J, Frazier J (2003) Marine Turtles in Latin America and the Caribbean: A Regional Perspective of Successes, Failures and Priorities for the Future. Marine Turtle Newsletter 100:38-42
Marcovaldi MA, Thomé JCA (1999b) Reducing threats to turtles. In: Eckert KL, Bjorndal KA, Abreu-Grobois FA, Donnelly M (eds) Research and Management Techniques for the conservation of sea turtles. IUCN/SSC Marine Turtle Specialist Group Publication No. 4.
Marcovaldi MA, Thome JCA, Almeida AdP, Lopez GG, da Silva ACCD, Apolinario M (2008) Satellite telemetry studies in Brazilian nesting areas: preliminary results. In: Rees AF, Frick M, Panagopoulou A, Williams K (eds) Proceedings of the 27th Annual Symposium on Sea Turtle Biology and Conservation. NOAA Technical Memorandum NMFS-SEFSC-569, Mrytle Beach, South Carolina. 262 p, p 47
Margaritoulis D (1988) Post-nesting movements of loggerhead sea turtles tagged in Greece. Rapports et Proces-verbaux des reunions de la Commission Internationale pour l'Exploration Scientifique de la Mer Mediterranee 31:284
Margaritoulis D (2005) Nesting activity and reproductive output of loggerhead sea turtles, *Caretta caretta*, over 19 seasons (1984-2002) at Laganas Bay, Zakynthos, Greece: The largest rookery in the Mediterranean. Chelonian Conservation and Biology 4:916-929
Margaritoulis D, Argano R, Baran I, Bentivegna F, Bradai MN, Caminas JA, Casale P, De Metrio G, Demetropoulos A, Gerosa G, Godley BJ, Haddoud DA, Houghton J, Laurent L, Lazar B (2003) Loggerhead turtles in the Mediterranean Sea: Present knowledge and conservation perspectives. In: Bolten AB, Witherington BE (eds) Loggerhead Sea Turtles. Smithsonian Books, Washington, DC. 319 p., p 175-198
Margaritoulis D, Demetropoulis A (2003) Proceedings of the First Mediterranean Conference on Marine Turtles. Barcelona Convention – Bern Convention – Bonn Convention (CMS), Nicosia, Cyprus. 270 p.
Margaritoulis D, Rees A, ARCHELON (2007) Loggerhead nesting in Greece. In: Mast RB, Bailey LM, Hutchinson BH (eds) SWoT Report—The State of the World's Sea Turtles, Volume II, Washington, DC. 49p. Available online *at*: http://seaturtlestatus.org/report/view
Margaritoulis D, Rees A, Grimanis K (2005) Monitoring work and conservation efforts for the loggerhead sea turtle nesting population in Laganas Bay, Zakynthos

Island, Greece, during 2005. Unpublished report. Athens: ARCHELON, the Sea Turtle Protection Society of Greece
Margaritoulis D, Teneketzis K (2002) The discovery of a green turtle developmental habitat in Greece advocates a stronger regional cooperation. In: Seminoff JA (ed) Proceedings of the Twenty-Second Annual Symposium on Sea Turtle Biology and Conservation. U.S. Dep. Commer. NOAA Tech. Memo. NMFS-SEFSC-503, Miami, Florida. 308 p, p 5
Marine Parks Authority of Seychelles (2008) Hawksbill nesting in Curieuse Island Marine Park, Seychelles. In: Mast RB, Bailey LM, Hutchinson BH (eds) SWoT Report—The State of the World's Sea Turtles, Volume III, Washington, DC. 43p. Available online *at*: http://seaturtlestatus.org/report/view
Marine Research Unit: Sabah Parks (2007) Turtle Islands Park and Sipadan Island Turtle Research Report. Unpublished report.
Marine Turtle Specialist Group (2007) Red List Assessment-*Lepidochelys olivacea*. 39 p.
Marquez R Pers. comm.
Marquez R, Villanueva A, Peñaflores C (1976) Sinopsis de datos biologicos sobre la tortuga golfina, *Lepidochelys olivacea* (Eschscholtz, 1829), Instituto Nationale, Pesca, Mexico,
Marquez-M. R (1990) Sea turtles of the world. FAO fisheries Synopsis Volume 11, No. 125
Marquez-M. R, Burchfield PM, Dias-F. J, Sanchez-P. M, Carrasco-A. M, Jimenez Q, C.;, Leo-P. A, Bravo-G. R, Pena-V. J (2005) Status of the Kemp's ridley sea turtle, *Lepidochelys kempii*. Chelonian Conservation and Biology 4:761-766
Marrese M, Bentivegna F (2006) Report from Seaturtle.org Forum. Available online *at*: http://wwwseaturtleorg/gforum/gforumcgi?post=4265
Martin C, Richardson S, Hays G (2002) How deep do hatchling green turtles swim? In: Seminoff JA (ed) Proceedings of the Twenty-Second Annual Symposium on Sea Turtle Biology and Conservation. U.S. Dep. Commer. NOAA Tech. Memo. NMFS-SEFSC-503, Miami, Florida. 308 p, p 46
Martin CS, Jeffers J, Godley J (2005) The status of marine turtles in Montserrat (Eastern Caribbean). Animal Biodiversity and Conservation 28:159-168
Martinez Karam SG (2004) Variación de microsatélites nucleares en la colonia reproductora de tortugas golfinas, *Lepidochelys olivacea* (Eschscholtz, 1829), de Escobilla, Oaxaca., Universidad Del Mar
Martinez LM (2000) Ecologia de anidacion de la tortuga golfina (*Lepidochelys olivacea*) en la Playa de La Cuevita, Costa Pacifica Chocoana, Colombia, en 1998. Actualidades biologicas Medellin 22:131-143
Matsuzawa Y (2005) Nesting beach management of eggs and pre-emergent hatchlings of North Pacific loggerhead sea turtles in Japan. In: Kinan I (ed) Proceedings of the Second Western Pacific Sea Turtle Cooperative Research and Management Workshop Volume II: North Pacific Loggerhead Sea Turtles. Western Pacific Regional Fishery Management Council, Honolulu, HI, USA
Mau R, S.; B, Richards A, Ningaloo Turtle Program (2006) Personal communication. Loggerhead nesting in Western Australia. In: Mast RB, Bailey LM, Hutchinson BH (eds) SWoT Report—The State of the World's Sea Turtles, Volume II, Washington, DC. 49p. Available online *at*: http://seaturtlestatus.org/report/view
Maxwell FD (1911) Reports on inland and sea fisheries in the Thongwa, Myaungmya, and Bassein districts and the turtle banks of the Irrawaddy division. Rangoon Government Printing Office 57 pp *as cited in* Groombridge, B and R Luxmoore (1989) The green turtle and hawksbill (Reptilia: Cheloniidae): world status, exploitation and trade Secretariat of the Convention on International Trade in Endangered Species of Wild Fauna and Flora, Lausanne, Switzerland, 601 pp
Mays JL, Shaver DJ (1998) Nesting Trends of Sea Turtles in National Seashores along Atlantic and Gulf Coast Waters of the United States., U.S. Fish and Wildlife Service, 67 p.
McCombe A, Bjorndal KA, Bolten AB (2002) Compensatory growth in the green turtle (Chelonia mydas): effects of transient food restriction and subsequent refeeding in hatchlings. In: Seminoff JA (ed) Proceedings of the Twenty-Second Annual Symposium on Sea Turtle Biology and Conservation. U.S. Dep. Commer. NOAA

Tech. Memo. NMFS-SEFSC-503, Miami, Florida. 308 p, p 37
McLachlan N, McLachlan B, McLachlan J, McLachlan B, Wreck Rock Turtle Monitoring Project (2006) Queensland Turtle Conservation Project, Wreck Rock Study 2005-2006. Queensland: Environmental Protection Agency Conservation and technical data report, vol. 2006, no. 6
Mclean S, Turtlewatch Akrotiri (2006) Western British Sovereign Base Area, Turtlewatch Akrotiri Report. In: Mast RB, Bailey LM, Hutchinson BH (eds) SWoT Report—The State of the World's Sea Turtles, Volume II, Washington, DC. 49p. Available online *at*: http://seaturtlestatus.org/report/view
McMahon C, Hays G (2006) Thermal niche, large-scale movements and implications of climate change for a critically endangered vertebrate. Global Change Biology 12:1330-1338
McMahon CR, Bradshaw C, Hays G (2007) Satellite tracking reveals unusual diving characteristics for a marine reptile, the olive ridley turtle Lepidochelys olivacea. Marine Ecology Progress Series 329:239-252
McMichael E, Carthy RR, Seminoff JA (2002) Evidence of homing behavior in juvenile green turtles in the northeastern Gulf of Mexico. In: Seminoff JA (ed) Proceedings of the Twenty-Second Annual Symposium on SeaTurtle Biology and Conservation. U.S. Dep. Commer. NOAA Tech. Memo. NMFS-SEFSC-503, Miami, Florida. 308 p, p 223-224
Mellgren RL, Mann MA (2002) Comparison of the green sea turtle to other species: the case of partial reinforcement effect. In: Seminoff JA (ed) Proceedings of the Twenty-Second Annual Symposium on SeaTurtle Biology and Conservation. U.S. Dep. Commer. NOAA Tech. Memo. NMFS-SEFSC-503, Miami, Florida. 308 p, p 224
Metcalfe J, Hampson K, Andriamizava A, Andrianirina R, Caimes T, Gray A, Ramiarisoa C, Sondotra H (2007) The importance of north-west Madagascar for marine turtle conservation. Oryx 41:232-238
Meylan A, Castillo I, Decastro Gonzales N, Ordoñez C, Troeng S, Ruia A, Meylan P (2006) Bastimentos Island National Marine Park and Playa Chiriqui: Protected areas vital to the recovery of the hawksbill turtle in Caribbean Panama. In: Frick MA, Panagopoulou A, Rees AF, Williams K (eds) Twenty sixth Annual Symposium on Sea Turtle Biology and Conservation, Athens, Greece. 376 p, p 145-146
Meylan A, Maylan P (1985) Nesting of *Dermochelys coriacea* in Caribbean Panama. Journal of Herpetology 19:293-297
Meylan A, Schroeder B, Mosier A (1995) Sea Turtle Activity in the State of Florida: 1979-1992, State of Florida, Department of Environmental Protection, Florida Marine Research Institute. Number 52. 51pp + Appendices.
Meylan AB, Meylan PA, Gray JA (1998) Density and biomass of green turtles in developmental habitat in Bermuda. In: Epperly S, Braun J (eds) Proceedings of the seventeenth annual sea turtle symposium. NOAA Technical Memorandum NMFS-SEFSC-415, Orlando, Florida. 342 p.
Meylan P, Meylan A (2007) Ecología y Migración de las Tortugas Marinas en la Provincia de Bocas del Toro, Panamá. Autoridad Nacional del Ambiente Unpublished report
Miller JD (2007a) The mystery of how they will adapt: How will climate change affect sea turtles? . In: Mast RB, Bailey LM, Hutchinson BH (eds) SWoT Report—The State of the World's Sea Turtles, Volume II, Washington, DC. 49p. Available online *at*: http://seaturtlestatus.org/report/view, p 13
Miller P, Laporta M, Domingo A, Lezama C, Rios M (2006) Bycatch assessment of sea turtles by a coastal bottom trawl fishery on the Rio de la Plata estuary, Uruguay. In: Frick HCI, Panagopoulo A, Rees AF, Williams K (eds) Proceedings of the Twenty-sixth Annual Symposium on Sea Turtle Biology and Conservation, Island of Crete, Greece. 376 p, p 256
Miller P, Laporta M., Fallabrino A (2007b) Sea Turtles and Trawl Fishery In The Rio de la Plata Estuary: What Is Going On Here? In: Mast RB, Hutchinson BJ, Hutchinson AH (eds) Proceedings of the Twenty Fourth Annual Symposium on Sea Turtle Biology and conservation. NOAA Technical Memorandum NMFS-SEFSC-567, San Jose, Costa Rica. 205 p., p 196

Minarik C (1985) Olive Ridleys of Honduras. Marine Turtle Newsletter 33:3-4
Mingozzi T (2008) Personal Communication.
Mingozzi T, Cambie G, Crispino F, Mico N, Urso S (2006) Loggerhead turtle, *Caretta caretta*, in Italy: A reappraisal of nesting activity within the national scenario. In: Frick MA, Panagopoulou A, Rees AF, Williams K (eds) Twenty sixth Annual Symposium on Sea Turtle Biology and Conservation, Athens, Greece. 376 p, p 308
Mingozzi T, et al (2006) Personal communication. unpublished data.
Mingozzi T, et al. (2007) Personal communication. unpublished data.
Mitro S (Forthcoming) Country report of Suriname *In* Proceedings of the Seventh Sea Turtle Symposium for the Guianas Georgetown, Guyana
Mobaraki A (2004) Green Turtle Nesting on the Gulf of Oman Coastline of the Islamic Republic of Iran. Marine Turtle Newsletter 104:11
Mobaraki A (2006) Report on Sea Turtle Tagging Program in Iran. Bureau of Wildlife and Aquatic Affairs, Department of the Environment unpublished report
Moll EO, Bhaskar S, Vijaya J (1983) Update on the Olive Ridley on the East Coast of India. Marine Turtle Newsletter 25:2-4
Momoemausu M, Ward J, Iakopo M, Ifopo P (2006) Report on the Hawksbill Turtle Nesting Survey 2005-2006. Samoa: Division of Environment and Conservation, Ministry of Natural Resources and Environment unpublished report
Moncada F (2006) Personal communication. Leatherback nesting in Cuba. In: Mast RB, Bailey LM, Hutchinson BH (eds) SWoT Report—The State of the World's Sea Turtles, Volume I, Washington, DC. 36 p. Available online *at*: http://seaturtlestatus.org/report/view
Moncada F (2008) Personal communication. Hawksbill nesting in Cuba. In: Mast RB, Bailey LM, Hutchinson BH (eds) SWoT Report—The State of the World's Sea Turtles, Volume III, Washington, DC. 43p. Available online *at*: http://seaturtlestatus.org/report/view
Moncada F, Carrillo E, Saenz A, Nodarse G (1999) Reproduction and nesting of hawksbill turtle, *Eretmochelys imbricata*, in the Cuban Archipelago. Chelonian Conservation and Biology 3:257-263
Moncada F, Carrillo E, Saenz A, Nodarse G (1999) Reproduction and Nesting of the Hawksbill Turtle, *Eretmochelys imbricata*, in the Cuban Archipelago. Chelonian Conservation and Biology 3:257-263
Moncada F, Nodarse G, Medina Y, Escobar E, Rodríguez C, Rodríguez AM, Morales E (2006) Annual Report on Hawksbill Turtle (*Eretmochelys imbricata*) research in Cuba (February 2005-February 2006). Cuba: Marine Turtle Project, Fisheries Research Center
Moncada F, Nodarse G, Medina Y, Hernandez F, Escobar E, Rodriguez C, Rodriguez AM, Morales E (2008) Reporte anual de investigacion y conservacion de las tortugas marinas realizadas por el CIP-MIP. Cuba, 2006.
Moncada-G. F, Rodrigues AM, Marquez-M. R, Carillo E (2000) Report of the Olive Ridley Turtle (Lepidochelys olivacea) in Cuban Waters. Marine Turtle Newsletter 90:13-15
Moncado GF, Nordarse A (1998) The green turtle (Chelonia mydas) in Cuba. In: Epperly S, Braun J (eds) Proceedings of the seventeenth annual sea turtle symposium. U.S. Dep. Commer. NOAA Tech. Memo. NMFS-SEFSC-415, Orlando, Florida. 342 p.
Montero L (2004) Leatherback nesting at Humacao, 2004. Internal report to DNER:3 p
Montero L (2006) Proyecto de Conservación de Tortugas Marinas Humacao, Yabucoa y Maunabo, Puerto Rico, Temporada 2006. DNER-PR, Unpublished report
Montiel-Villalobos MG, Barrios-Garrido H (2002) Abundance of sub-adult green turtles (Chelonia mydas) captured in the Gulf of Venezuela. In: Seminoff JA (ed) Proceedings of the Twenty-Second Annual Symposium on SeaTurtle Biology and Conservation. U.S. Dep. Commer. NOAA Tech. Memo. NMFS-SEFSC-503, Miami, Florida. 308 p, p 291
Monzon-Arguello C, Lopez-Jurado L, Rico C, Marco A, Lopez P, Hays G, Lee P (2010b) Evidence from genetic and Lagrangian drifter data for transatlantic transport of small juvenile green turtles. Journal of Biogeography 37:1752-1766
Monzon-Arguello C, Munoz J, Marco A, Lopez-Jurado L, Rico C (2008) Twelve new

polymorphic microsatellite markers from the loggerhead sea turtle (Caretta caretta). Conservation Genetics 9:1045-1049
Monzon-Arguello C, Rico C, Carreras C, Calabuig P, Marco A, Lopez-Jurado LF (2009) Variation in spatial distribution of juvenile loggerhead turtles in the eastern Atlantic and western Mediterranean Sea. Journal of Experimental Marine Biology and Ecology 373:79-86
Monzon-Arguello C, Rico C, Marco A, Lopez P, Lopez-Jurado LF (2010c) Genetic characterization of eastern Atlantic hawksbill turtles at a foraging group indicates major undiscovered nesting populations in the region. Journal of Experimental Marine Biology and Ecology 387:9-14
Monzón-Argüello C, Rico C, Naro-Maciel E, Varo-Cruz N, López P, Marco A, López-Jurado L (2010a) Population structure and conservation implications for the loggerhead sea turtle of the Cape Verde Islands. Conservation Genetics
Moore MK, Bemiss JA, Rice SM, Quattro JM, Woodley CM (2003) Use of restriction fragment length polymorphisms to identify sea turtle eggs and cooked meats to species. Conservation Genetics 4:95-103
Moran K, Bjorndal KA, Bolten AB (2002) Green turtle grazing: effects on seagrass ecosystems. In: Seminoff JA (ed) Proceedings of the Twenty-Second Annual Symposium on Sea Turtle Biology and Conservation. U.S. Dep. Commer. NOAA Tech. Memo. NMFS-SEFSC-503, Miami, Florida. 308 p, p 31
Moreira L (2003) Ecologia Reprodutiva e Estimativa de Ninhos da Tartaruga Verde-Aruanã - Chelonia mydas (Linnaeus, 1758) (Testudines, Reptilia) na Ilha da Trindade - Espírito Santo - Brasil. M.Sc. Thesis. Universidade Federal do Espírito Santo
Moreira L, Baptistotti C, Scalfone L, Thomé JC, de Almeida APLS (1995) Occurrence of Chelonia mydas on the Island of Trindade, Brazil. Marine Turtle Newsletter 70:2
Moreira L, Bjorndal KA (2006) Estimates of green turtle (Chelonia mydas) nests on Tinidade Island, Brazil, South Atlantic. In: Pilcher NJ (ed) Proceedings of the Twenty-third annual symposium on sea turtle biology and conservation. NOAA technical memorandum NMFS-SEFSC-536, Kuala Lumpur, Malaysia. 261pp., p 174
Moreno A (2002) Contribución al conocimiento de las tortugas marinas en el Parque Nacional Natural Tayrona, durante los meses de junio-julio de 2002. Informe de Pasantia UAESPNN Santa Marta, Colombia
Morreale SJ, Standora EA, Spotila JR, Paladino FV (1996) Migration corridor for sea turtles. Nature 384:319-320
Morris K (1984) The National Report for the country of Saint Vincent. In: Bacon, et al (eds) Proceedings of the Western Atlantic Sea Turtle Symposium, Volume 3. University of Miami Press, Miami, Florida, San Jose, Costa Rica, p 381-385
Morris K (1987) The National Report for the country of St. Vincent and the Grenadines *In* Western Atlantic Turtle Symposium II, Mayaguez, Puerto Rico. 10 p
Morris R, Balazs GH, Spraker TR, Work TM (2002) Pharyngeal nodules seen in Hawaiian green turtles. In: Seminoff JA (ed) Proceedings of the Twenty-Second Annual Symposium on SeaTurtle Biology and Conservation. U.S. Dep. Commer. NOAA Tech. Memo. NMFS-SEFSC-503, Miami, Florida. 308 p, p 303-304
Mortimer J (2004) Personal Communication. Cited in Seminoff, J.A., (assessor). 2004. Global Status Assessment: Green turtle (*Chelonia mydas*). Marine Turtle Specialist Group. Species Survival Commission, Red List Programme: 71.
Mortimer J, Bird Island Lodge (2008) Personal communication. Hawksbill nesting on Bird Island, Seychelles. In: Mast RB, Bailey LM, Hutchinson BH (eds) SWoT Report—The State of the World's Sea Turtles, Volume III, Washington, DC. 43p. Available online *at*: http://seaturtlestatus.org/report/view
Mortimer JA (1984) Marine Turtles on the Republic of Seychelles: Status and Management. IUCN Conservation Library Gland 80 pp +4 pl
Mortimer JA (1988) Green turtle nesting at Aldabra Atoll - population estimates and trends. Biological Society of Washington Bulletin 8:116-128
Mortimer JA (2004) Seychelles Marine Ecosystem Management Project (SEYMEMP): Turtle Component. Final report, vol 1 and vol 2

Mortimer JA (2005) Sea Turtles of D'Arros Island and St. Joseph Atoll: Status and Recommendations. In: Engelhardt U (ed) Proceedings of a Scientific Symposium held at the D'Arros Research Centre. D'Arros Research Centre, Seychelles
Mortimer JA (2007b) Hawksbill sea turtle (*Eretmochelys imbricata*) five-year review. National Marine Fisheries Service & US Fish and Wildlife Service, Jacksonville, FL, 93 p
Mortimer JA, Broderick D (1999a) Population genetic structure and developmental migrations of sea turtles in the Chagos Archipelago and adjacent regions inferred from mtDNA sequence variation. In: Sheppard CRC, Seaward MRD (eds) Ecology of the Chagos Archipelago, Vol 2. Linnean Society Occasional Publications. 350 p, p 185-194
Mortimer JA, Carr A (1987) Reproduction and Migrations of the Ascension Island Green Turtle (Chelonia mydas). Copeia 1987:103-113
Mortimer JA, Day M (1999b) Sea turtle populations and habitats in the Chagos Archipelago. In: Sheppard CRC, Seaward MRD (eds) Ecology of the Chagos Archipelago, Vol 2. Linnean Society Occasional Publications. 350 p.
Mortimer JA, Donnelly M (2007a) IUCN Red List Status Assessment: hawksbill turtle (*Eretmochelys imbricata*). IUCN/SSC-Marine Turtle Specialist Group, 121 p
Mortimer JA, Jupiter T, Collie J, Chapman R, Liljevik A, Betsy B, Stevenson J, Laboudallon V, Assary M, Augeri D, Pierce S (2006) Trends in the green turtle (Chelonia mydas) nesting population at Aldabra Atoll, Seychelles (WIO) and their implications for the region. In: Pilcher NJ (ed) Proceedings of the Twenty-Third Annual Symposium on Sea Turtle Biology and Conservation, Kuala Lumpur, Malaysia. 261 p., p 75-77
Mortimer JA, Portier KM (1989) Reproductive Homing and Internesting Behavior of the Green Turtle (Chelonia mydas) at Ascension Island, South Atlantic Ocean. Copeia 1989:962-977
Moundemba J-B (1999) Cited in Fretey, J. 2001. Biogeography and conservation of marine turtles of the Atlantic coast of Africa. Secretariat, Convention on Migratory Species, CMS technical series publication, no. 6, Bonn, Germany.
Mounguéngui G-A, Verhage B (2007) Update after Five years of Marine Turtle monitoring in Gamba, Gabon (2002-2007). IBONGA-ACPE, WWF technical report
Mounguéngui G-A, Verhage B (2008) Activités de recherche et de suivi des tortues marines sur les plages de Gamba au Gabon. IBONGA-ACPE, WWF Rapport technique final
Mrosovsky N, Ryan G, James M (2009) Leatherback turtles: The menace of plastic. Marine Pollution Bulletin 58:287-289
Muccio C (1998) Informe Nacional Sobre El Estado De La Conservacion De Tortugas Marinas En Guatemala. Asociación Rescate y Conservación de Vida Silvestre (ARCAS) Unpublished report, 25 pp + annexes
Muccio C, ARCAS (2006) Leatherback nesting in the Hawaii area of Guatemala. In: Mast RB, Bailey LM, Hutchinson BH (eds) SWoT Report—The State of the World's Sea Turtles, Volume II, Washington, DC. 49p. Available online *at*: http://seaturtlestatus.org/report/view
Muccio C, ARCAS (2006) Leatherback nesting in the Hawaii area of Guatemala. In: Mast RB, Bailey LM, Hutchinson BH (eds) SWoT Report—The State of the World's Sea Turtles, Volume I, Washington, DC. 36 p. Available online *at*: http://seaturtlestatus.org/report/view
Muccio C, ARCAS (2009) Leatherback nesting in the Hawaii area of Guatemala. In: Mast RB, Hutchinson BJ, Villegas PE, Wallace B, Yarnell L (eds) SWoT Report—The State of the World's Sea Turtles, Volume IV, Washington, DC. 49 p. Available online *at*: http://seaturtlestatus.org/report/view
Muir C (2008) Personal communication. Hawksbill nesting in Tanzania. In: Mast RB, Bailey LM, Hutchinson BH (eds) SWoT Report—The State of the World's Sea Turtles, Volume III, Washington, DC. 43p. Available online *at*: http://seaturtlestatus.org/report/view
Munhofen J, Ramirez S (2007) Tagging and Nesting Research on Hawksbill Turtles (*Eretmochelys imbricata*) at Jumby Bay, Long Island, Antigua, West Indies.

Jumby Bay Hawksbill Project, WIDECAST unpublished report
Munson L, al. e (2008) Personal Communication. Works in progress - STENAPA.
Murray PA (1984) The National Report for the country of Saint Lucia. In: Bacon PR, Et. al (eds) Proceedings of the First Western Atlantic Turtle Symposium, Vol 3, Appendix 7 University of Miami Press, Miami, Florida. University of Miami Press, Miami, Florida, San Jose, Costa Rica, p 370-380
Muurmans M (2008) Personal communication.
Nagaoka SM, Bondiolo ACV, Monteiro-Filho ELdA (2008) Sea turtle bycatch by Cerco-fixo in Cananeia Lagoon Estuarine Complex, Sao Paulo, Brazil Marine Turtle Newsletter 119:4-6
Nannarelli S, De Lucia A, A.; D, Piovano S (2006) Nesting activity of the Loggerhead Sea Turtle *Caretta caretta* on the Pocket Beach of Linosa Island. In: Frick M, Panagopoulou A, Rees AF, Williams K (eds) Proceedings of the Twenty Sixth Annual Symposium on Sea Turtle Biology and Conservation, Athens, Greece. 376 p
Naro-Maciel E, Becker JH, Lima EHSM, Marcovaldi MA, R. D (2007) Testing dispersal hypotheses in foraging green sea turtles (*Chelonia mydas*) of Brazil. Journal of Heredity 98:29-39
Naseeb F, Scholte P (2006) Socotra Conservation and Development Programme. 2007. Loggerhead nesting on Socotra Island, Yemen. In: Mast RB, Bailey LM, Hutchinson BH (eds) SWoT Report—The State of the World's Sea Turtles, Volume II, Washington, DC. 49p. Available online *at*: http://seaturtlestatus.org/report/view
Nathai-Gyan N, James C, Hislop G (1987) National report for Trinidad and Tobago Western Atlantic Turtle Symposium II. Forestry Division, Ministry of Food Production, Marine Exploitation, Forestry and Evnironment, Puerto Rico. 228 p
National Marine Fisheries Service, U.S. Fish and Wildlife Service (1998) Recovery Plan for U.S. Pacific Populations of the Loggerhead Turtle (*Caretta caretta*). National Marine Fisheries Service, Silver Spring, MD
National Marine Fisheries Service and U.S. Fish and Wildlife Service (In review) US Kemp's ridley Recovery Plan.
National Office of Wildlife and Hunting (2006) Data from the 2006 nesting season. Unpublished report
National Park Service: Buck Island Sea Turtle Research Program (2006) Leatherback nesting at Buck Island Reef National Monument, St. Croix, U.S. Virgin Islands. In: Mast RB, Bailey LM, Hutchinson BH (eds) SWoT Report—The State of the World's Sea Turtles, Volume II, Washington, DC. 49p. Available online *at*: http://seaturtlestatus.org/report/view
National Park Service: Buck Island Sea Turtle Research Program (2006) Leatherback nesting at Buck Island Reef National Monument, St. Croix, U.S. Virgin Islands. In: Mast RB, Bailey LM, Hutchinson BH (eds) SWoT Report—The State of the World's Sea Turtles, Volume I, Washington, DC. 36 p. Available online *at*: http://seaturtlestatus.org/report/view
National Park Service: Buck Island Sea Turtle Research Program (2007) Leatherback nesting at Buck Island Reef National Monument, St. Croix, U.S. Virgin Islands. In: Mast RB, Bailey LM, Hutchinson BH (eds) SWoT Report—The State of the World's Sea Turtles, Volume II, Washington, DC. 49p. Available online *at*: http://seaturtlestatus.org/report/view
Nature Protection Trust of Seychelles (2007) 2006–2007 Silhouette Conservation Project: Quarterly Report. unpublished report
Nature Protection Trust of Seychelles (2008) Silhouette Conservation Project. Quarterly Report unpublished report
Nature Seychelles (2008) Hawksbill nesting on Cousin Island, Seychelles. In: Mast RB, Bailey LM, Hutchinson BH (eds) SWoT Report—The State of the World's Sea Turtles, Volume III, Washington, DC. 43p. Available online *at*: http://seaturtlestatus.org/report/view
Nature Tropicale (2006) Suivi ecologique et protection des tortues marines sur le littoral du Benin (2005 - 2006). Rapport d'activites n 0010/PTM/NT
Naughton JJ (2001) Sea turtle turvey at Oroluk Atoll and Minto Reef, Federated States of Micronesia. Marine Turtle Newsletter 55:9-12

Nava M (2006) Sea Turtle Conservation Bonaire: Progress Report 2006. Unpublished report
Nava M, Uhr A, van Dam R (2007) Sea Turtle Conservation Bonaire:, 27 p
Nel R (2006) Annual season report 2005/2006: Maputaland sea turtle monitoring and related monitoring programmes. Report for Ezemvelo KwaZulu-Natal Wildlife: 1-38
Nel R (2006) Personal communication. Leatherback nesting in South Africa. In: Mast RB, Bailey LM, Hutchinson BH (eds) SWoT Report—The State of the World's Sea Turtles, Volume I, Washington, DC. 36 p. Available online *at*: http://seaturtlestatus.org/report/view
Nel R (2008) Sea Turtles of KwaZulu-Natal: Data Report for 2007/8 Season, Nelson Mandela Metropolitan University
Nel R, Papillon J (2005) Turtle monitoring programme: Season report for 2004-2005. Report for Ezemvelo KwaZulu-Natal Wildlife:1-22
Nel R, Wright R (2006) Ezemvelo KwaZulu-Natal. Leatherback Nesting Season Report from South Africa. In: Mast RB, Bailey LM, Hutchinson BH (eds) SWoT Report—The State of the World's Sea Turtles, Volume II, Washington, DC. 49p. Available online *at*: http://seaturtlestatus.org/report/view
Nelson T (2006) Turtle Activities Report: 2001-2003
NGO KAWAN, Marine Turtle Network of Martinique (2008) Hawksbill nesting in Martinique. In: Mast RB, Bailey LM, Hutchinson BH (eds) SWoT Report—The State of the World's Sea Turtles, Volume III, Washington, DC. 43p. Available online *at*: http://seaturtlestatus.org/report/view
NGO SEPANMAR (2006) Nesting Season 2006: Parts 1, 2, and 3. Fort de France, Martinique. Ministère de l'Écologie et du Développement Durable technical report.
Nichols WJ (2007) Loggerhead sea turtle (*Caretta caretta*) 5-year review: summary and evaluation. National Marine Fisheries Service and US Fish and Wildlife Service 65 p
Nichols WJ, Resenditz A, Seminoff JA, Resenditz B (2000) Transpacific migration of a loggerhead turtle monitored by satellite telemetry Bulletin of Marine Science 67:937-947
Nisbeth BM, Nature Foundation (2008) Hawksbill nesting in Gibbs Bay, St. Maarten. In: Mast RB, Bailey LM, Hutchinson BH (eds) SWoT Report—The State of the World's Sea Turtles, Volume III, Washington, DC. 43p. Available online *at*: http://seaturtlestatus.org/report/view
Nisbeth BM, Nature Foundation (2008) Hawksbill nesting in Gibbs Bay, St. Maarten. Report
Nobetsu T, Minami H, Matsunaga H, Kiyota M, Yokota K, Kimura N, Nakano H (2004) Nesting and post-nesting studies of loggerhead turtles (*Caretta caretta*) at Omaezaki, Japan *In* Proceedings of the International Symposium on SEASTAR2000 and Bio-logging Science, Japan
Okayama T, Diaz T, Koike H, Diez CE, Marquez-M. R, Espinosa G (1996) Mitochondrial DNAanalysis of the hawksbill turtle .I. Haplotype detection among samples in the Pacific and Atlantic Oceans. International Symposium on Network and Evolution of Molecular Information, Tokyo
Okemwa GM, Nzuki S, Mueni EM (2004) The Status and Conservation of Sea Turtles in Kenya Marine Turtle Newsletter 105:1-6
Okemwa GM, Wamukota A (2006) An overview of the status of green turtles (*Chelonia mydas*) in Kenya. In: Frick M, Panagopoulou A, Rees AF, Williams K (eds) Proceedings of the Twenty Sixth Annual Symposium on Sea Turtle Biology and Conservation, Athens, Greece. 376 p, p 311
Olendo M (2008) Personal communication. Hawksbill nesting in Kenya. In: Mast RB, Bailey LM, Hutchinson BH (eds) SWoT Report—The State of the World's Sea Turtles, Volume III, Washington, DC. 43p. Available online *at*: http://seaturtlestatus.org/report/view
Olendo M (2008) Green turtle nesting - Kiunga Marine National Reserve(KMNR) - Kenya.
ONCFS Martinique (2007) Leatherback nesting in Martinique. In: Mast RB, Bailey LM, Hutchinson BH (eds) SWoT Report—The State of the World's Sea Turtles,

Volume II, Washington, DC. 49p. Available online *at*: http://seaturtlestatus.org/report/view
Ordoñez C (2006) Personal communication. Leatherback nesting in Panama. In: Mast RB, Bailey LM, Hutchinson BH (eds) SWoT Report—The State of the World's Sea Turtles, Volume I, Washington, DC. 36 p. Available online *at*: http://seaturtlestatus.org/report/view
Ordoñez C, et al. (2007) Report on Monitoring and Research Activities, Chiriqui Beach, Panama. unpublished report
Ordoñez C, Ruiz A, Troeng S, Meylan A, Meylan P (2006) 2005 Hawksbill (*Eretmochelys imbricata*) research and population recovery at Chiriqui Beach and Escudo de Veragas Island, and bastimentos Island National Marine Park. Final project report
Ordonez C, Troeng S, Meylan A, Meylan P, Ruiz A (2007) Chiriqui Beach, Panama, the Most Important Leatherback Nesting Beach in Central America. Chelonian Conservation and Biology 6:122-126
Ordoñez C, Troëng S, Meylan A, Meylan P, Ruiz A (2007) Chiriquí Beach, Panama, the most important leatherback nesting beach in Central America. Chelonian Conservation and Biology 6:122-126
Ottenwalder JA (1982) Estudio preliminar sobre el status, distribución y biología reproductiva de las tortugas marinas en la República Dominicana, Departamento de Biología, Universidad Autónoma de Santo Domingo, Santo Domingo, República Dominicana
Ottenwalder JA (1987) Ad Hoc National Report to WATS II for Haiti *In* The Second Western Atlantic Turtle Symposium (WATS2 044), Mayagüez, Puerto Rico
Ottenwalder JA (1996) The current status of sea turtles in Haiti, Society for the Study of Amphibians and Reptiles
Pacific Whale Foundation Sea Turtles: A Hawai'i Wildlife Guide. available online *at*: http://www.pacificwhale.org. .
Páez V (2006) Leatherback nesting in Colombia. In: Mast RB, Bailey LM, Hutchinson BH (eds) SWoT Report—The State of the World's Sea Turtles, Volume I, Washington, DC. 36 p. Available online *at*: http://seaturtlestatus.org/report/view
Palumbi SR (1997) Molecular biogeography of the Pacific. Coral Reefs (Supplement) 16: S47-S52
Panagopoulos D, Sofouli E, Teneketzis K, Margaritoulis D (2001) Stranding data as an indicator of fisheries induced mortality of sea turtles in Greece. In: Margaritoulis D, Demetropoulis A (eds) Proceedings of the first Mediterranean conference on marine turtles, Barcelona Convention – Bern Convention – Bonn Convention (CMS). Nicosia, Cyprus. 270 pp.
Pandav B, Choudhury BC (1998) Olive Ridley Tagged in Orissa Recovered in the Coastal Waters of Eastern Sri Lanka. Marine Turtle Newsletter 82:9-10
Papi F, Liew H-C, Luschi P, Chan E-H (1995) Long.range migratory travel of a green turtle tracked by satellite: evidence for navigational ability in the open sea. Marine Biology 122:171-175
Papillon J (2007) Maputaland sea turtle monitoring: Season report for 2006/2007. Report for Ezemvelo KwaZulu-Natal Wildlife 1-26
Parmenter CJ (1983) Reproductive migration in the hawksbill turtle (*Eretmochelys imbricata*). Copeia 1983:271-273
Parsons JJ (1962) The Green Turtle and Man, Vol. University of Florida Press, Gainesville
Patiño Martínez J, Quiñones L (2006) Leatherback nesting in La Playona, Acandí, Colombia. In: Mast RB, Bailey LM, Hutchinson BH (eds) SWoT Report—The State of the World's Sea Turtles, Volume II, Washington, DC. 49p. Available online *at*: http://seaturtlestatus.org/report/view
Patino-Martinez J, Marco A, QuiÒones L, Godley B (2008) Globally significant nesting of the leatherback turtle (Dermochelys coriacea) on the Caribbean coast of Colombia and Panama. Biological Conservation 141:1982-1988
Patiño-Martinez J, Quiñones L (2008) Personal communication. Hawksbill nesting in Colombia. In: Mast RB, Bailey LM, Hutchinson BH (eds) SWoT Report—The State of the World's Sea Turtles, Volume III, Washington, DC. 43p. Available online *at*: http://seaturtlestatus.org/report/view

Patiño-Martínez J, Quiñones L (2008) Personal communication. Hawksbill nesting in Panama. In: Mast RB, Bailey LM, Hutchinson BH (eds) SWoT Report—The State of the World's Sea Turtles, Volume III, Washington, DC. 43p. Available online *at*: http://seaturtlestatus.org/report/view

Pavia A, Monterrosa C (2008) Hawksbill nesting in Tayrona National Park, Colombia. In: Mast RB, Bailey LM, Hutchinson BH (eds) SWoT Report—The State of the World's Sea Turtles, Volume III, Washington, DC. 43p. Available online *at*: http://seaturtlestatus.org/report/view

Pavía A, Monterrosa MC (2007) Fortalecimiento del proceso de conservación de las tortugasanidantes en el Parque Nacional Natural Tayrona – Caribe Colombiano. Temporada 2007, Fundación Colombia Marina, Bogotá, Colombia. 55 p

Pearce AF (2001) Contrasting population structure of the loggerhead turtle (*Caretta caretta*) using mitochondrial and nuclear DNA markers. Master's Thesis. University of Florida

Pearce AF, Bowen BW (2001) Identification of loggerhead turtle (*Caretta caretta*) stock structure in the southeastern United States and adjacent regions using nuclear DNA markers. Report 99-Sec-04 National Marine Fisheries Service, Silver Spring, Maryland.

Peckham S, Maldonado Diaz D, Koch V, Mancini A, Gaos A, Tinker M, Nichols W (2008) High mortality of loggerhead turtles due to bycatch, human consumption and strandings at Baja California Sur, Mexico, 2003 to 2007. Endangered Species Research 5:171-183

Peckham S, Maldonado Diaz D, Walli A, Ruiz G, Crowder L, Nichols W (2007) Small-scale fisheries bycatch jeopardizes Endagered Pacific loggerhead turtles. Public Library of Science ONE 2

Pelletier D, Roos D, Ciccione S (2003) Oceanic survival and movements of wild and captive-reared immature green turtles (*Chelonia mydas*) in the Indian Ocean. Aquatic Living Resources 16:35-41

Pemberton E, Nevis Department of Fisheries (2008) Hawksbill nesting in Nevis. In: Mast RB, Bailey LM, Hutchinson BH (eds) SWoT Report—The State of the World's Sea Turtles, Volume III, Washington, DC. 43p. Available online *at*: http://seaturtlestatus.org/report/view

Pena J (2009) Personal Communication. Nesting sites of Kemps Ridley sea turtles, *Lepidochelys kempii*, at Tepehuajes, Barra Coma, and Playa Dos, Tamaulipas, Mexico. .

Peñaflores-Salazar C, Vasconcelos-Pérez J, Albavera-Padilla E, Jiménez Quiroz. MC (2001) Especies sujetas a protección especial. Tortuga golfina. In: Cisneros M, A., Beléndez LF, Zárate E, Gaspar MT, López LC, Saucedo C, Tovar J (eds) Sustentabilidad y Pesca Responsable en México Evaluación y Manejo 1999-2000 Publicado en CD, Instituto Nacional de la Pesca/SEMARNAT. México.

Pendoley K (2009) Flatback nesting in the Pilbara Region of Western Australia: Personal communication. In: Mast RB, Hutchinson BJ, Villegas PE, Wallace B, Yarnell L (eds) SWoT Report—The State of the World's Sea Turtles, Volume IV, Washington, DC. 49 p. Available online *at*: http://seaturtlestatus.org/report/view

Pendoley K, ; , Chaloupka M, Prince R (In press) An encouraging conservation outlook for the most atypical marine turtle species in the world: the endemic flatback. Endangered Species Research

Pendoley K, Howitt L, Speirs M (2008) Hawksbill nesting in Western Australia. In: Mast RB, Bailey LM, Hutchinson BH (eds) SWoT Report—The State of the World's Sea Turtles, Volume III, Washington, DC. 43p. Available online *at*: http://seaturtlestatus.org/report/view

Pereira MAM, Videira EJS, Narane DA (2009) Monitoring, tagging and conservation of marine turtles in Mozambique: 2008/09 Annual report.1-4

Pérez J, Gómez R, Estrada C, Bran A, Alfaro C (2006) Personal communication. Leatherback nesting in Guatemala. In: Mast RB, Bailey LM, Hutchinson BH (eds) SWoT Report—The State of the World's Sea Turtles, Volume I, Washington, DC. 36 p. Available online *at*: http://seaturtlestatus.org/report/view

Petro G (2006) Leatherback Nesting beach surveys in Vanuatu. Vanua-Tai Resource

Monitors Turtle Conservation Network report to NOAA
Petro G, Hickey F, Mackay K (2005) Leatherback Turtles in Vanuatu. In: Kinan I (ed) Proceedings of the Second Western Pacific Sea Turtle Cooperative Research and Management Workshop Volume I: West Pacific Leatherback and Southwest Pacific Hawksbill Sea Turtles. Western Pacific Regional Fishery Management Council: Honolulu, HI, USA, Honolulu, Hawaii, USA. 118 p p73-77
Petro G, Hickey FR, Mackay K (2007) Leatherback turtles in Vanuatu. Chelonian Conservation and Biology 6:135-137
Petro G, Hickey FR, Mackay K (2007) Leatherback Turtles in Vanuatu. Chelonian Conservation and Biology 6:135-137
Piedra R (2008) Personal communication. Hawksbill nesting on Playa Langosta, Costa Rica. In: Mast RB, Bailey LM, Hutchinson BH (eds) SWoT Report—The State of the World's Sea Turtles, Volume III, Washington, DC. 43p. Available online *at*: http://seaturtlestatus.org/report/view
Piedra R, Vélez E (2004) Reporte de actividades de investigación y protección de la tortuga baula (*Dermochelys coriacea*) temporada de anidación 2003-2004 Playa Langosta. Proyecto de Conservación en Tortugas Marinas – Tortuga Baula, Parque Nacional Marino Las Baulas, Guanacaste, Costa Rica unpublished manuscript
Piedra R, Vélez E (2005) Reporte de actividades de investigación y protección de la tortuga baula (*Dermochelys coriacea*), temporada de anidación 2004-2005, Playa Langosta. Proyecto de Conservación en Tortugas Marinas - Tortuga Baula, Parque Nacional Marino Las Baulas, Guanacaste, Costa Rica unpublished manuscript
Piedra R, Velez E, Dutton P, Possardt E, Padilla C (2007) Nesting of the Leatherback Turtle (Dermochelys coriacea) from 1999,Äì2000 Through 2003,Äì2004 at Playa Langosta, Parque Nacional Marino Las Baulas de Guanacaste, Costa Rica. Chelonian Conservation and Biology 6:111-116
Pierre-Nathoniel D (2007) Personal Communication. Sea turtle nesting in Saint Lucia. In: Mast RB, Bailey LM, Hutchinson BH (eds) SWoT Report—The State of the World's Sea Turtles, Volume II, Washington, DC. 49p. Available online *at*: http://seaturtlestatus.org/report/view
Pilcher N (2006) The 2005-2006 Leatherback Nesting Season Huon Coast, Papua New Guinea. Western Pacific Regional Fishery Management Council final report
Pilcher N (2007) Personal communication. Loggerhead nesting on Masirah Island, Oman. In: Mast RB, Bailey LM, Hutchinson BH (eds) SWoT Report—The State of the World's Sea Turtles, Volume II, Washington, DC. 49p. Available online *at*: http://seaturtlestatus.org/report/view
Pilcher NJ (1999) The hawksbill turtle, *Eretmochelys imbricata*, in the Arabian Gulf. Chelonian Conservation and Biology 3:312-317
Pilcher NJ (2000) The green turtle, *Chelonia mydas*, in the Saudi Arabian Gulf JOURNAL ARTICLE NOT FOUND ON WEBSITE, (LINK BELOW). Chelonian Conservation and Biology 3:730-734
Pilcher NJ (2006) The Huon Coast Leatherback Turtle Conservation Project. Final report, Western Pacific Regional Fisheries Management Council, Honolulu, HI, USA. 18 p
Pilcher NJ, Al-Merghani M (2000) Reproductive biology of green turtles at Ras Baridi, Saudi Arabia. Herpetological Review 31:142-147
Pilcher NJ, Marine Research Foundation (2005) Status of Sea Turtles in Qatar. Qatar: Supreme Council for the Environment and Natural Reserves final report
Pinedo MC, Polacheck T (2004) Sea turtle by-catch in pelagic longline sets off southern Brazil. Biological Conservation 119:335-339
Plotkin PT (1994a) Migratory and reproductive behavior of the olive ridley turtle, Lepidochelys olivacea, (Eschscholtz, 1829) in the eastern Pacific Ocean. . Texas A&M University
Plotkin PT (2007a) Olive ridley sea turtle (*Lepidochelys olivacea*) five-year review: summary and evaluation. NMFS & USFWS, Jacksonville, FL, 67 pp
Plotkin PT, (ed.) (2007b) Biology and conservation of ridley sea turtles, Vol. Johns Hopkins University Press, Baltimore, MD, USA

Plotkin PT, Byles R, Rostal DC, Owens DW (1995) Independent versus socially facilitated oceanic migrations of the olive ridley, *Lepidochelys olivacea*. Marine Biology 122:137-143
Plotkin PT, Byles RA, Owens DW (1994b) Post-breeding movements of male olive ridley sea turtles Lepidochelys olivacea from a nearshore breeding area. Proceedings of the Fourteenth Annual Sea Turtle Symposium NOAA TM-NMFS-SEFSC-351, p 119
Plotkin PT, Byles RA, Owens DW (1994c) Migratory and reproductive behavior of Lepidochelys olivacea in the eastern Pacific Ocean. Proceedings of the Thirteenth Annual Sea Turtle Symposium NOAA TM-NMFS-SEFSC-341 p138
Poiner LR, Harris ANM (1996) Incidental capture, direct mortality and delayed mortality of sea turtles in Australia's northern prawn fishery. Marine Biology 125:813-825
Poloczanska ES, Limpus CJ, Hays G (2009) Vulnerability of marine turtles to climate change. Advances in Marine Biology 56:151-211
Polovina JJ, Balazs GH, Howell EA, Parker DM, Seki MP, Dutton PH (2004) Forage and migration habitat of loggerhead (*Caretta caretta*) and olive ridley (*Lepidochelys olivacea*) sea turtles in the central North Pacific Ocean. Fisheries Oceanography 13:36-51
Possardt F (2007) Personal communication. Olive ridley nesting beaches in Liberia. Fish and WIldlife Serivce. Cited in Plotkin, P.T. 2007. Olive ridley sea turtle (Lepidochelys olivacea) five-year review: summary and evaluation. NMFS & USFWS, Jacksonville, FL, 67 pp.
Prince R (1993) Western Australian Marine Turtle Conservation Project: An Outline of Scope and an Invitation to Participate. Marine Turtle Newsletter 60:8-14
Prince R (1994) Status of the western Australian marine turtle populations: the Western Australian Marine Turtle Project 1986-1990. In: James R (ed) Proceedings of the Australian Marine Turtle Conservation Workshop. Queensland Department of Environment and Heritage and Australian Nature Conservation Agency, Sea World Nara Resort, Gold Coast, Australia, p 1-14
Prince R (2000) The Western Australian Marine Turtle Project. In: Pilcher NJ, Ismail G (eds) Sea turtles of the Indo-Pacific: research, management and conservation. ASEAN Academic Press, London, p 94-99
Prince R, Jensen M, Oades D, Rangers BJ (2010) Olive ridley turtle presence and nesting records for Western Australia. Marine Turtle Newsletter, in press
Prince RIT (2001) Pers. comm.
Prince RIT, Western Australia Marine Turtle Program (2009) Personal communication. Flatback nesting in Western Australia. In: Mast RB, Hutchinson BJ, Villegas PE, Wallace B, Yarnell L (eds) SWoT Report—The State of the World's Sea Turtles, Volume IV, Washington, DC. 49 p. Available online *at*: http://seaturtlestatus.org/report/view
Pritchard P (1993) Personal communication. Status and distribution of sea turtles in Suriname. Cited in Reichart, H.A. 1993. Synopsis of biological data on the olive ridley sea turtle Lepidochelys olivacea (Eschscholtz 1829) in the western Atlantic. NOAA Technical Memorandum NMFS-SEFSC-336, 78 pp.
Pritchard PCH (1966) Sea Turtles of Shell Beach, British Guiana. Copeia 1966:123-125
Pritchard PCH (1969) The Survival Status of Ridley Sea-turtles in American Waters. Biological Conservation 2:13-17
Pritchard PCH (1973) International Migrations of South American sea turtles *Chelonidae* and *Dermochelidae*. Animal Behaviour 21:18-27
Pritchard PCH (1984) Marine Turtles in Trinidad and Tobago. FAO Consultancy Report, Evaluation and Developmentof Marine Turtles in Trinidad and Tobago unpublished report:35 p
Pritchard PCH (2006) Personal communication. Leatherback nesting in Guyana. In: Mast RB, Bailey LM, Hutchinson BH (eds) SWoT Report—The State of the World's Sea Turtles, Volume I, Washington, DC. 36 p. Available online *at*: http://seaturtlestatus.org/report/view
Pritchard PCH, Trebbau P (1984) The Turtles of Venezuela, Vol. Society for the Study of Amphibians and Reptiles
Proietti MC, Lara-Ruiz P, Weiner Reisser J, da Silva L, Dellagostin OA, Marins LF

(2009) Green turtles (*Chelonia mydas*) foraging at Arvoredo Island in Southern Brazil: genetic characterization and mixed stock analysis through mtDNA control region haplotypes. Online *at* http://www.scielo.br/pdf/gmb/v32n3/a27v32n3.pdf. Genetics and Molecular Biology [online] 32:613-618
Projecto TAMAR Unpublished data
Projecto TAMAR (2008) Unpublished data. Projeto TAMAR-ICMBio database
Projeto TAMAR (2007) Personal communication. Loggerhead nesting in Brazil. In: Mast RB, Bailey LM, Hutchinson BH (eds) SWoT Report—The State of the World's Sea Turtles, Volume II, Washington, DC. 49p. Available online *at*: http://seaturtlestatus.org/report/view
Projeto TAMAR (2007) Loggerhead nesting in Brazil. In: Mast RB, Bailey LM, Hutchinson BH (eds) SWoT Report—The State of the World's Sea Turtles, Volume II, Washington, DC. 49p. Available online *at*: http://seaturtlestatus.org/report/view
Provita (2004) Programa Procosta. Proyecto Integral de Conservación y Desarrollo (PICD-Costa) Barlovento. In: Bararro R, Sanz A, Mora B (eds) Tortugas marinas en Venezuela. Acciones para su conservación. Oficina Nacional de Diversidad Biológica Fondo Editorial Fundambiente, Caracas, Venezuela, p 91-98
PROVITA (2006) Leatherback nesting in Venezuela. In: Mast RB, Bailey LM, Hutchinson BH (eds) SWoT Report—The State of the World's Sea Turtles, Volume I, Washington, DC. 36 p. Available online *at*: http://seaturtlestatus.org/report/view
Pusineri C, Quillard M (2008) Bycatch of Protected Megafauna in the Artisanal Coastal Fishery of Mayotte Island, Mozambique Channel. Western Indian Ocean Journal of Marine Science 7:195-206
Putra KS (2005) Brief Overview of Turtle Conservation in Indonesia. unpublished report
Putra KS (2006) Personal communication. Leatherback nesting in Indonesia. In: Mast RB, Bailey LM, Hutchinson BH (eds) SWoT Report—The State of the World's Sea Turtles, Volume I, Washington, DC. 36 p. Available online *at*: http://seaturtlestatus.org/report/view
Putra KS (2006) Personal communication. Leatherback nesting in Indonesia. In: Mast RB, Bailey LM, Hutchinson BH (eds) SWoT Report—The State of the World's Sea Turtles, Volume II, Washington, DC. 49p. Available online *at*: http://seaturtlestatus.org/report/view
Putrawidjaja M (2000) Marine Turtles in Irian Jaya, Indonesia. Marine Turtle Newsletter 90:8-10
Quaintance J, Rice MR, Balazs GH (2002) Basking, foraging, and resting behavior of two sub-adult green turtles in Kiholo Bay Lagoon, Hawaii. In: Seminoff JA (ed) Proceedings of the Twenty-Second Annual Symposium on SeaTurtle Biology and Conservation. U.S. Dep. Commer. NOAA Tech. Memo. NMFS-SEFSC-503, Miami, Florida. 308 p, p 225-226
Quijada A, Balladares C (2004) Conservación de las tortugas marinas en el Golfo de Paria. In: Babarro R, Sanz A, Mora B (eds) Tortugas marinas en Venezuela Acciones para su conservación. Oficina Nacional de Diversidad Biológica. Fondo Editorial Fundambiente, Caracas, Venezuela. 117 p, p 47-54
Quirós W, Chacón. D (2005) Informe del proyecto de conservación e investigación de la tortuga baula *Dermochelys coriacea* dentro del Refugio Nacional de Vida Silvestre Ostional, Temporada 2004-2005. Playa Ostional. Guanacaste, Costa Rica. 32 p
Rader H, Nsue E, S., Bradsby J, Morra W, Hearn G (2007) Leatherback nesting in Bioko Island, Equatorial Guinea. In: Mast RB, Bailey LM, Hutchinson BH (eds) SWoT Report—The State of the World's Sea Turtles, Volume II, Washington, DC. 49p. Available online *at*: http://seaturtlestatus.org/report/view
Rader HA, Angel Ela Mba M, Morra W, Hearn G (2006) Marine turtles on the southern coast of Bioko Island (Gulf of Guinea, Africa), 2001-2005. Marine Turtle Newsletter 111:8-10
Rader HA, Nsue Esono S, Bradsby J, Morra W, Hearn G (2008) Marine turtle nest counts and beach choices on Bioko Island (Gulf of Guinea, Africa) across six nesting seasons (2000/2001 through 2005/2006). In: Rees AF, Frick HC,

Panagopoulou A, Williams K (eds) Proceedings of the Twenty-Seventh Annual Symposium on Sea Turtle Biology and Conservation. U.S. Department of Commerce NOAA Technical Memorandum NMFS-SEFSC-569, Mrytle Beach, South Carolina. 261 p, p 246-247

Rajakaruna RS, Dissanayake, D.M.N., Ekanayake, E.M.L., Ranawana, K.B. (2009) Sea turtle conservation in Sri Lanka: assessment of knowledge, attitude and prevalence of consumptive use of turtle products among coastal communities. Indian Ocean Turtle Newsletter 10

Rakotonirina BP, Cooke A (1994) Sea turtles of Madagascar: Their status, exploitation and conservation. Oryx 28:51-61

Ram K (2000) Offshore studies on olive ridley sea turtles in Gahirmatha, Orissa. Kachhapa 3:13-15

Ramohia P, Siota C, Motui D, Routanis F, Pema M, Rini C, Zama M, Tetabea T, Madada L, Willy G (2007) Hawksbill and Green Turtle Nesting Activities on Kerehikapa Island in the Arnavon Community Managed Conservation Area. Report for The Nature Conservancy, Solomon Islands Field Office unpublished manuscript

Rankin-Baransky K, Williams CJ, Bass AL, Bowen BW, Spotila JR (2001) Origin of Loggerhead Turtles Stranded in the Northeastern United States as Determined by Mitochondrial DNA Analysis. Journal of Herpetology 35:638-646

Rashid SMA, Islam MZ (2006) Status and conservation of marine turtles in Bangladesh. In: Shanker K, Choudhury BC (eds) Marine Turtles of the Indian Subcontinent. Universities Press, India, Hyderguda, p 200-216

Rees A (2008) Hawksbill nesting in Oman. In: Mast RB, Bailey LM, Hutchinson BH (eds) SWoT Report—The State of the World's Sea Turtles, Volume III, Washington, DC. 43p. Available online *at*: http://seaturtlestatus.org/report/view

Rees AF, Baker SL (2006) Hawksbill and Olive Ridley Nesting on Masirah Island, Sultanate of Oman: an Update. Marine Turtle Newsletter 113:2-5

Rees AF, Saad A, Jony M (2005) Tagging green turtles (*chelonia mydas*) and loggerhead turtles (*caretta caretta*) in Syria. Testudo 6:51-55

Rees AF, Saad A, Jony M (2008) Discovery of a regionally important green turtle Chelonia mydas rookery in Syria. Oryx 42:456-459.

Reich KJ, Bjorndal KA, Bolten AB (2007) The 'lost years' of green turtles: using stable isotopes to study cryptic lifestages. Biology Letters 3:712-714

Reichart HA (1993b) Synopsis of biological data on the olive ridley sea turtle *Lepidochelys olivacea* (Eschscholtz 1829) in the western Atlantic. NOAA Technical Memorandum NMFS-SEFSC-336, 78 p

Reichart HA, Fretey J (1993a) WIDECAST Sea Turtle Recovery Action Plan for Suriname (Karen L. Eckert, Editor). CEP Technical Report No. 24. UNEP Caribbean Environment Programme, Kingston, Jamaica xiv + 65 pp

Reichart HA, Fretey JF (1993) Sea Turtle Recovery Action Plan for Suriname, UNEP Caribbean Environment Programme, 65 p.

Reis EC, L.S.; S, S.M.; V, Santos FR, J.; YR, K.A.; B, A.B.; B, Lobo-Hajdu G (2009) Genetic composition, population structure and phylogeography of the loggerhead sea turtle: colonization hypothesis for the Brazilian rookeries. Conservation Genetics Published online *at* http://www.springerlink.com/content/k03135t6r77wq0v4

Rene F, Roos D (1996) The status of sea turtle conservation in French Territories of the Indian Ocean: Isles Eparces. In: Humphrey SL, Salm RV (eds) Status of Sea Turtle Conservation in the Western Indian Ocean. UNEP Regional Seas Reports and Studies No. 165, Nairobi, Kenya. 162 pp., p 151-156

Republic of Cuba (1997) An annotated transfer of the Cuban population of hawksbill turtles (*Eretmochelys imbricata*) from Appendix I to Appendix II, submitted in accordance with Resolution Conf. 9.24 and 9.20. Proposal submitted to the Tenth Conference of the Parties to CITES, 9-20 June 1997, Harare, Zimbabwe

Revelles M, Carreras C, L.; C, Marco A, Bentivegna F, Castillo JJ, de Martino G, Mons JL, Smith MB, Rico C, Pascual M, Aguilar A (2007) Evidence for an asymmetrical size exchange of loggerhead sea turtles between the Mediterranean and the Atlantic through the Straits of Gibraltar. Journal of Experimental Marine Biology and Ecology 349:261-271

Reyes (1989) *in* Groombridge, B. and R. Luxmoore (1989) The green turtle and hawksbill (Reptilia: Cheloniidae): world status, exploitation and trade. Secretariat of the Convention on International Trade in Endangered Species of Wild Fauna and Flora.601 pp.

Reynolds M, Share the Beach (2005) Alabama Sea Turtle Nesting Report. In: Mast RB, Bailey LM, Hutchinson BH (eds) SWoT Report—The State of the World's Sea Turtles, Volume II, Washington, DC. 49p. Available online *at*: http://seaturtlestatus.org/report/view

Reynolds M, Share the Beach (2007) Loggerhead nesting in Alabama. In: Mast RB, Bailey LM, Hutchinson BH (eds) SWoT Report—The State of the World's Sea Turtles, Volume II, Washington, DC. 49p. Available online *at*: http://seaturtlestatus.org/report/view

Richards A, Mau R, Macgregor K, Bedford S (2005) Ningaloo Turtle Program, Western Australia. Annual report 2005

Richardson P (2007) New insights into Sri Lankan Green turtle migration. Online *at*: http://www.ioseaturtles.org/pom_detail.php?id=56.

Richardson S, Broderick AC, Coyne MS, Glen F, Godley BJ, Hays G (2002) Overwintering behavior of green turtles in the Mediterranean. In: Seminoff JA (ed) Proceedings of the Twenty-Second Annual Symposium on SeaTurtle Biology and Conservation. U.S. Dep. Commer. NOAA Tech. Memo. NMFS-SEFSC-503, Miami, Florida. 308 p, p 52

Rincón P, Rivera D, Rodriguez CJ, Tello P (2001) Establecimiento y caracterización de puntos focales de anidamiento de tortugas marinas en los sectores de cañaveral y arrecifes en el Parque Nacional Natural Tayrona. Seminario de Investigación (Proyecto II), Universidad Jorge Tadeo Lozano, Facultad de Biología Marina Santa Marta, Colombia

Rios D (2000-2005) Annual reports. Dirección General de Vida Silvestre SEMARNAT México

Rivalan P (2004) La dynamique des populations de tortues luths de Guyane Français: Recherche des facteures impliques et applicatin a la mise en place de stratégies de conservation. Ph.D., Université de Paris XI Orsay

Roberts MA, Karl SA (1998) Global population structure of green sea turtles (Chelonia mydas) usingmicrosatellite analysis of male-mediated gene flow. In: Epperly S, Braun J (eds) Proceedings of the seventeenth annual sea turtle symposium. U.S. Dep. Commer. NOAA Tech. Memo. NMFS-SEFSC-415, Orlando, Florida. 342 p.

Ron T (2006) Personal communication. Leatherback nesting in Angola. In: Mast RB, Bailey LM, Hutchinson BH (eds) SWoT Report—The State of the World's Sea Turtles, Volume I, Washington, DC. 36 p. Available online *at*: http://seaturtlestatus.org/report/view

Rondón MA, Hernández RS, Guada HJ (2004) Research and conservation of sea turtles in the Paria Peninsula, Venezuela: Results of the 2003 nesting season. In: Mast RB, Hutchinson BJ, Hutchinson AH (eds) Proceedings of the Twenty Fourth Annual Symposium on Sea Turtle Biology and conservation. NOAA Technical Memorandum NMFS-SEFSC-567, San Jose, Costa Rica. 205 p.

Rondón Médicci mA, Guada HJ, Revuelta Abin O, Montilla A (2006) Results of the 2005 Nesting Season of the Sea Turtle Research and Conservation Project in the Paria Peninsula, Sucre State, Venezuela. In: Frick HCI, Panagopoulo A, Rees AF, Williams K (eds) Proceedings of the Twenty-sixth Annual Symposium on Sea Turtle Biology and Conservation, Island of Crete, Greece. 376 p, p 156

Ross (1989) *in* Groombridge, B. and R. Luxmoore (1989) The green turtle and hawksbill (Reptilia: Cheloniidae): world status, exploitation and trade. Secretariat of the Convention on International Trade in Endangered Species of Wild Fauna and Flora, Lausanne, Switzerland. 601 p

.

Ross JP, Banwari MA (1982) Review of sea turtles in the Arabian area, Vol. Smithsonian Institute Press, Washington D.C

Ross JP, Ottenwalder JA (1983) The leatherback turtle, *Dermochelys coriacea*, nesting in the Dominican Republic. In: Rhodin A, Miyata K (eds) Advances in Herpetology and Evolutionary Biology. Museum of Comparative Zoology

Harvard, Cambridge, MA, p 706-713
Ruiz GA (1994) Sea turtle nesting population at Playa La Flor, Nicaragua: an olive ridley "arribada" beach. In: Bjorndal KA (ed) Proceedings of the Fourteenth Annual Symposium on Sea Turtle Biology and Conservation. NOAA Technical Memorandum NMFS-SEFSC-351, Hilton Head, South Carolina. 323 p, p 129-130
Rulie AC (2002) Reflexion sur la conservation de la tortue imbriquee (*Eretmochelys imbricata*): Application aux Seychelles. Ecole Nationale Veterinaire Toulouse Annee 2002 These: 2002-TOU3-4166
Saad A, Ali A, Darwish A (2006) Marine turtle nesting survey, Syria 2005. In: Frick M, Panagopoulou A, Rees AF, Williams K (eds) Proceedings of the Twenty Sixth Annual Symposium on Sea Turtle Biology and Conservation, Athens, Greece. 376 p, p 320
Saad MA (1999) Hadramaut coast importance in conservation of endangered greem turtle. Marine Sciences Resorces Research Center addendum Unpublished report:8 pages
Sabah Department of Wildlife (2008) Hawksbill nesting in Sabah, Malaysia. In: Mast RB, Bailey LM, Hutchinson BH (eds) SWoT Report—The State of the World's Sea Turtles, Volume III, Washington, DC. 43p. Available online *at*: http://seaturtlestatus.org/report/view
Sagun VG (2004) Postnesting Movements of Green Turtles Tagged in the Turtle Islands Tawi-Tawi, Philippines. Marine Turtle Newsletter 104:5-7
Sakamoto W, Bando T, Arai N, Baba N (1997) Migration paths of adult female and male loggerhead turtles *Caretta caretta* determined through satellite telemetry. Fisheries Science 63:547-552
Sako T, Horikoshi K (2002) Marine debris ingested by green turtles in the Ogasawara Islands, Japan. In: Seminoff JA (ed) Proceedings of the Twenty-Second Annual Symposium on SeaTurtle Biology and Conservation. U.S. Dep. Commer. NOAA Tech. Memo. NMFS-SEFSC-503, Miami, Florida. 308 p, p 305
Sales G, Giffoni B, Barata PCR (2008) Incidental catch of sea turtles by the Brazilian pelagic longline fishery. Journal of the Marine Biological Association of the United Kingdom 88:853-864
Sales G, Giffoni BB, Fiedler FN, Azevedo VG, Kotas JE, Swimmer Y, Bugoni L (In review) Circle hook effectiveness for the mitigation of sea turtle bycatch and capture of target species in a Brazilian pelagic longline fishery. Aquatic Conservation: Marine and Freshwater Ecosystems
Salm RV (1976) Critical marine habitats of the northern Indian Ocean. Contract report to the IUCN, Morges, Switzerland. *In* Kar, C.S. & S. Bhaskar. 1982. Status of sea turtles in the eastern Indian Ocean. pp. 365-372. *In*: K.A. Bjorndal (ed.) Biology and conservation of sea turtles, Smithsonian Institution Press, Washington DC.
Salm RV (1976) Critical marine habitats of the northern Indian Ocean. Contract report to the IUCN, Morges, Switzerland
Salm RV (1988) Coral reefs of the western Indian Ocean: A threatened heritage. Ambio 12:349-353
Salm RV, Jensen RAC, Papastavrou VA (1993) Marine fauna of Oman: Cetaceans, turtles, seabirds, and shallow water corals. IUCN, Gland, Switzerland
Sampson E (2005) Proyecto Conservacion de Tortugas Tora, *Dermochelys coriacea*, Reserva Natural Isla Juan Venado, Octubre 2004 - Febrero 2005. Informe tecnico de proyecto. Fauna & Flora International. In: Mast RB, Bailey LM, Hutchinson BH (eds) SWoT Report—The State of the World's Sea Turtles, Volume II, Washington, DC. 49p. Available online *at*: http://seaturtlestatus.org/report/view
Sanchez Okrucky R (1998) Pathologies, treatment, and prevention in captive green turtle, Chelonia mydas, in the Mexican Caribbean. In: Epperly S, Braun J (eds) Proceedings of the seventeenth annual sea turtle symposium. U.S. Dep. Commer. NOAA Tech. Memo. NMFS-SEFSC-415, Orlando, Florida. 342 p.
Santidrián Tomillo P, Veléz E, Reina RD, Piedra R, Paladino FV, Spotila JR (2007) Reassessment of the leatherback turtle (*Dermochelys coriacea*) nesting population at Parque Nacional Marino Las Baulas, Costa Rica: effects of conservation efforts. Chelonian Conservation and Biology 6:54-62

Santos IR, Friedrich AC, Barretto FP (2005) Overseas garbage pollution on beaches of northeast Brazil. Marine Pollution Bulletin 50
Sarti L (2004) Situación actual de la tortuga laúd Dermochelys coriacea en el Pacífico Mexicano y medidas para su recuperación y conservación. Publicación patrocinada por el WWF. Mexico: Secretaría del Medio Ambiente y Recursos Naturales
Sarti M, L., Barragán RAR, Juárez C JA, (ed.) (2004) Conservación y evaluación de la población de tortuga laúd *Dermochelys coriacea* en el Pacífico Mexicano, temporada de anidación 2003-2004. DGVS-SEMARNAT-Kutzari, Asociación para el Estudio y Conservación de las Tortugas Marinas A C
Sarti Martínez L, Barragán AR, Muñoz DG, García N, Huerta P, Vargas F (2007) Conservation and biology of the leatherback turtle in the Mexican Pacific. Chelonian Conservation and Biology 6:70-78
Save My Future Foundation (SAMFU) (2008) Personal communication. Hawksbill nesting in Liberia. In: Mast RB, Bailey LM, Hutchinson BH (eds) SWoT Report—The State of the World's Sea Turtles, Volume III, Washington, DC. 43p. Available online *at*: http://seaturtlestatus.org/report/view
Schauble C, Kennett R, Winderlich S (2006) Flatback Turtle (Natator depressus) Nesting at Field Island, Kakadu National Park, Northern Territory, Australia, 1990,Äì2001. Chelonian Conservation and Biology 5:188-194
Schroth W, Streit B, Schierwater B (1996) Evolutionary handicap for turtles. Nature 384:521-522
Schulz JP (1975) Sea turtles nesting in Surinam. . Zoologische Verhandelingen 143:1-143
Schulz JP (1975) Sea turtles nesting in Surinam. Zoologische Verhandelingen 143: 1-143. *in* Kelle, L., N. Gratiot & B. De Thoisy. 2009. Olive ridley turtle Lepidochelys olivacea in French Guiana: back from the brink of regional extirpation? Oryx 43: 243-246.
Schulz JP (1984) Turtle conservation strategy in Indonesia. IUCN/WWF Report no 6 *in* FIVE YEAR REVIEW
Schulz JP (1987) Status of and trade in *Chelonia midas* and *Eretmochelys imbricata* in Indonesia. Consultancy report prepared for IUCN Conservation Monitoring Centre *IN* FIVE YEAR REVIEW
Schumacher I, Herbst LH, Kerben MJ, Ehrhart LM, Bagley DA, Klein PA (1998) Vitellogenin levels in green turtles (Chelonia mydas). In: Epperly S, Braun J (eds) Proceedings of the seventeenth annual sea turtle symposium. U.S. Dep. Commer. NOAA Tech. Memo. NMFS-SEFSC-415, Orlando, Florida. 342 p.
Scott N, Horrocks JA (1993) WIDECAST Sea Turtle Recovery Action Plan for St. Vincent and the Grenadines (Karen L. Eckert, Editor). CEP Technical Report No 27 UNEP Caribbean Environment Programme, Kingston, Jamaica xiv + 80pp
Scott NM, Horrocks JA (1993) Sea Turtle Recovery Plan for St. Vincent and the Grenadines, UNEP Caribbean Environment Programme, Kingston, Jamaica. 80 p
Sea Turtle Association of Japan (2006) Sea turtle data in 2006: Nesting of hawksbill turtles in 2006 *In* Proceedings of the 17th Japan Sea Turtle Symposium. Sea Turtle Association of Japan, Kumano Shichirimihama, Japan
Sea Turtle Conservation Bonaire (2007) Personal communication. Loggerhead nesting in Bonaire. In: Mast RB, Bailey LM, Hutchinson BH (eds) SWoT Report—The State of the World's Sea Turtles, Volume II, Washington, DC. 49p. Available online *at*: http://seaturtlestatus.org/report/view
Sea Turtle Foundation (2009) Flatback nesting on Aims Beach, Queensland. In: Mast RB, Hutchinson BJ, Villegas PE, Wallace B, Yarnell L (eds) SWoT Report—The State of the World's Sea Turtles, Volume IV, Washington, DC. 49 p. Available online *at*: http://seaturtlestatus.org/report/view
Searle L (2002) Diet of green turtles (Chelonia mydas) captured at the Robinson Point foraging ground, Belize. In: Seminoff JA (ed) Proceedings of the Twenty-Second Annual Symposium on SeaTurtle Biology and Conservation. U.S. Dep. Commer. NOAA Tech. Memo. NMFS-SEFSC-503, Miami, Florida. 308 p, p 228-229
SEDERE (2003) Proyecto del Centro Veracruzano para la Investigación y

Conservación de la Tortuga Marina, Gobierno del Estado de Veracruz. SEDERE-CEMA. PEMEX- Gerencia Regional de Seguridad Industrial y Protección Ambiental. Xalapa, Veracruz. 70 p. Inédito
Segniagbeto GH (2006) Personal communication. Leatherback nesting in Togo. In: Mast RB, Bailey LM, Hutchinson BH (eds) SWoT Report—The State of the World's Sea Turtles, Volume I, Washington, DC. 36 p. Available online *at*: http://seaturtlestatus.org/report/view
Sella I (1982) Sea turtles in the eastern Mediterranean and northern Red Sea. In: Bjorndal KA (ed) Biology and conservation of sea turtles. Smithsonian Insitituion Press, Washginton, DC. 583 p., p 417-423
Seminoff J, Shanker K (2008b) Marine turtles and IUCN Red Listing: A review of the process, the pitfalls, and novel assessment approaches. Journal of Experimental Marine Biology and Ecology 356:52-68
Seminoff JA (2002) First Direct Evidence of Migration by an East Pacific Green Seaturtle from Michoacan, México to a Feeding Ground on the Sonoran Coast of the Gulf of California. The Southwestern Naturalist 47:314-316
Seminoff JA (2002) Global status of the green turtle (Chelonia mydas): the 2002 MTSG green turtle assessment for the IUCN Red List Programme. In: Seminoff JA (ed) Proceedings of the Twenty-Second Annual Symposium on SeaTurtle Biology and Conservation. U.S. Dep. Commer. NOAA Tech. Memo. NMFS-SEFSC-503, Miami, Florida. 308 p, p 250
Seminoff JA, (assessor) (2004) Global Status Assessment: Green turtle (*Chelonia mydas*). Marine Turtle Specialist Group Species Survival Commission, Red List Programme:71
Seminoff JA, Dutton PH (2007b) Leatherback Turtles (Dermochelys coriacea) in the Gulf of California: Distribution, Demography, and Human Interactions. Chelonian Conservation and Biology 6:137-141
Seminoff JA, Jones T, Resenditz A, Nichols WJ, Chaloupka M (2003) Monitoring green turtles (*Chelonia mydas*) at a coastal foraging area in Baja California, Mexico: using multiple indices to describe population status. Journal of Marine Biological Association UK 83:1355-1362
Seminoff JA, Resenditz A, Nichols WJ (2002) Home range of green turtles Chelonia mydas at a coastal foraging area in the Gulf of California, Mexico. Marine Ecology Progress Series 242:253-265
Seminoff JA, Schroeder BA (2007) Green sea turtle (*Chelonia mydas*) 5-year review: summary and evaluation. National Marine Fisheries Service, Silver Spring, Maryland 102 pp
Seminoff JA, Zarate P, Coyne MS, Foley A, Parker DM, Lyon BN, Dutton DM (2008a) Post-nesting migrations of Galápagos green turtles *Chelonia mydas* in relation to oceanographic conditions: integrating satellite telemetry with remotely sensed ocean data. Endangered Species Research 4:57-72
Senegas J-B, Hochscheid S, Groul J-M, Lagarrigue B, Bentivegna F (2008) Discovery of the most north-western loggerhead turtle *Caretta caretta* nest site in the Mediterranean Sea. Journal of the Marine Biological Association UK Biodiversity Records 2008:4 p
Service NMFSaUSFaW (2009) Recovery Plan for the Northwest Atlantic Population of the Loggerhead Sea Turtle (Caretta caretta). National Marine Fisheries Service, Silver Spring, MD
Settle S (1995) Status of Nesting Populations of Sea Turtles in Thailand and Their Conservation. Marine Turtle Newsletter 68:8-13
Seychelles Islands Foundation (SIF) (2008) Hawksbill nesting in Aldabra Atoll, Seychelles. In: Mast RB, Bailey LM, Hutchinson BH (eds) SWoT Report—The State of the World's Sea Turtles, Volume III, Washington, DC. 43p. Available online *at*: http://seaturtlestatus.org/report/view
Shanker K (2000) Conservation genetics of olive ridleys on the east coast of India. Kachhapa 3:9-10
Shanker K (2004) Marine turtle status and conservation in the Indian Ocean. FAO Fisheries Report No 738 Supplement
Shanker K, Andrews H (2006) Towards an integrated and collaborative sea turtle conservation programme in India: a UNEP/CMS-IOSEA Project Report. Centre

for Herpetology/Madras Crocodile Bank Trust, Post Bag 4, Mamallapuram, Tamil Nadu
Shanker K, Andrews HV (2004) Tracing the migrations of Indian marine turtles: towards an integrated and collaborative conservation program. Interim report-June 2004, Centre for Herpetology/Madras Crocodile Bank Trust Tamil Nadu Convention On The Conservation Of Migratory Species Of Wild Animals, United Nations Environment Programme / CMS Secretariat
Shanker K, Choudhury BC, (eds.) (2006) Marine Turtles of The Indian Subcontinent, Vol. Universities Press, India, Hyderguda, Hyderabad, India
Shanker K, Pandav B, Choudhury BC (2003) An assessment of the olive ridley turtle (*Lepidochelys olivacea*) nesting population in Orissa, India. Biological Conservation 115:149-160
Shanker K, Pilcher NJ (2003) Marine Turtle Conservation in South and Southeast Asia: Hopeless Cause or Cause for Hope? Marine Turtle Newsletter 100:43-51
Shanker K, Ramadevi J, Choudhury BC, Singh L, Aggarwal RK (2004) Phylogeography of olive ridley turtles (*Lepidochelys olivacea*) on the east coast of India: implications for conservation theory. Molecular Ecology 13:1899-1909
Shanker K, Tripathy B, Pandav B (2005) Biological Studies on Sea Turtles on the Coast of Orissa. Indian Ocean Turtle Newsletter 1:10-11
Sharath BK (2006) Sea turtles along the Karnataka coast. In: Shanker K, Choudhury BC (eds) Marine Turtles of the Indian Subcontinent. Universities Press, India, Hyderguda, p 141-146
Shaver D (2007) Personal communication. Loggerhead nesting in Texas. In: Mast RB, Bailey LM, Hutchinson BH (eds) SWoT Report—The State of the World's Sea Turtles, Volume II, Washington, DC. 49p. Available online *at*: http://seaturtlestatus.org/report/view
Shaver D (2009) Personal communication. In: Mast RB, Hutchinson BJ, Villegas PE, Wallace B, Yarnell L (eds) SWoT Report—The State of the World's Sea Turtles, Volume IV, Washington, DC. 49 p. Available online *at*: http://seaturtlestatus.org/report/view
Shaver D, Rubio C (2008) Post-nesting movement of wild and head-started Kemp's ridley sea turtles Lepidochelys kempii in the Gulf of Mexico. Endangered Species Research 4:43-55
Shaver D, Schroeder BA, Byles RA, Burchfield PM, Pena J, Marquez R, Martinez HJ (2005a) Movements and home ranges of adult male Kemp's ridley sea turtles (Lepidochelys kempii) in the Gulf of Mexico investigated by satellite telemetry. Chelonian Conservation and Biology 4:817-827
Shaver DJ (2005b) Analysis of the Kemp's ridley imprinting and headstart project at Padre Island National Seashore, Texas, 1978-88, with subsequent nesting and stranding records on the Texas coast. Chelonian Conservation and Biology 4:846-859
Shaver DJ (2005c) Kemp's ridley sea turtle project at Padre Island National Seashore and Texas sea turtle nesting and stranding 2003 report. US Department of the Interior, Corpus Christi, TX
Shaver DJ (2009) Texas sea turtle nesting and stranding 2008 report. U.S. Department of the Interior, Corpus Christi, TX.
Sherrill-Mix S, James M, Myers R (2007) Migration cues and timing in leatherback sea turtles. Behavioral Ecology 19:231-236
Shillinger GL, Palacios DM, Bailey H, Bograd SJ, Swithenbank AM, Gaspar P, Wallace BP, Spotila JR, Paladino FV, Piedra R, Eckert SA, Block BA (2008) Persistent Leatherback Turtle Migrations Present Opportunities for Conservation. PLoS Biology 6:e171
Shillinger GL, Swithenbank AM, Bograd SJ, Bailey H, Castelton MR, Wallace BP, Spotila JR, Paladino FV, Piedra R, Block BA (2010) Identification of high-use internesting habitats for eastern Pacific leatherback turtles: role of the environment and implications for conservation. Endangered Species Research 10:215-232
Shiode D, Kawarabayashi N, Toukai T (2006) Sea turtle bycatch in large size pound net in coastal waters of Japan as determined through questionnaire. Teichi: 54-62

Shoop CR, Kenney RD (1992) Seasonal Distributions and Abundances of Loggerhead and Leatherback Sea Turtles in Waters of the Northeastern United States. Herpetological Monographs 6:43-67

Siaffa DD, Aruna E, Fretey J (2003) Presence of sea turtles in Sierra Leone (West Africa). In: Seminoff JA (ed) Proceedings of the twenty-second annual symposium sea turtle biology and conservation. NOAA Technical Memorandum NMFS-SEFSC-503, Miami, Florida. 307 pp, p 285

Sicily WWF (2006) Riserva Naturale Orientata, Torre Salsa. Available online *at*: http://www.wwftorresalsa.it/02_08_06.htm.

Silverman R (2006) Leatherback nesting on the Osa Peninsula, Costa Rica. In: Mast RB, Bailey LM, Hutchinson BH (eds) SWoT Report—The State of the World's Sea Turtles, Volume I, Washington, DC. 36 p. Available online *at*: http://seaturtlestatus.org/report/view

Siota C, Ramohia P (2007) Peak Hawksbill Nesting Activities in the Arnavon Community Marine Conservation Area. Report for The Nature Conservancy, Solomon Islands Field Office, Honiara unpublished manuscript

Smith GW (1992) Hawksbill turtle nesting at Manatee Bar, Belize, 1991. marine Turtle Newsletter 57:1-5

Smith GW, Eckert KL, Gibson JP (1992) Sea Turtle Recovery Action Plan for Belize, Kingston, Jamaica. 86 p

Soares LS (2004) Identificacão de populacões de tartarugas cabeçudas (*Caretta caretta*, Linnaeus 1758) no litoral brasileiro atraves de sequencias do mtDNA. Msc. Thesis. Pontifcia Universidade Catolica de Minas Gerais

Solano R (2006) Personal communication. Leatherback nesting in Playa Nuevo Pacuare, Costa Rica. In: Mast RB, Bailey LM, Hutchinson BH (eds) SWoT Report—The State of the World's Sea Turtles, Volume II, Washington, DC. 49p. Available online *at*: http://seaturtlestatus.org/report/view

Solís DS, Orrego CM, Blanco-Segura RS, Harfush-Meléndez MR, Albavera-Padilla EO, Valverde RA (2007) Estimating Arribada Size: Going Global. In: Rees AF, Frick MA, Panagopoulou AF, Williams K (eds) Proceedings of the Twenty-Seventh Annual Symposium on Sea Turtle Biology and Conservation. NOAA Technical Memorandum NMFS-SEFSC-569, Mrytle Beach, South Carolina. 261 p., p 249

Solomon J, Blumenthal J (2006) Cayman Islands Government, Department of Environment Annual Marine Turtle Beach Monitoring Program 2006. unpublished report

Solomon J, Blumenthal J (2007) Annual Marine Turtle Beach Monitoring Program 2007. Cayman Islands Government, Department of Environment unpublished report

Solomon JL, Blumenthal JB, Austin TJ, Ebanks-Petrie G, Broderick AC, Godley BJ (2006) Insights into the nesting population of marine turtles in the Cayman Islands. In: Frick MA, Panagopoulou A, Rees AF, Williams K (eds) Twenty sixth Annual Symposium on Sea Turtle Biology and Conservation, Athens, Greece. 376 p, p 323-324

Solorzano E, Begona M (2006) Venezuela Second Annual Report to the Inter-American Convention for the Protection and Conservation of Sea Turtles., Ministry of Environment and Natural Resources, National Office for Biological Diversity. Unpublished report, 19 p

Song X, Wang H, Wang W, Gu H, Chan S, Jiang H (2002) Satellite Tracking of Post-Nesting Movements of Green Turtles Chelonia mydas from the Gangkou Sea Turtle National Nature Reserve, China, 2001. Marine Turtle Newsletter 97:8-9

Soto JMR, Beheregaray RCP, Rebello RARP (1997b) Range extension: nesting by *Dermochelys* and *Caretta* in southern Brazil. Marine Turtle Newsletter 77:6-7

Soto JMR, Beheregarry RCP (1997a) New records of lepidochelys olivacea (eschscholtz, 1829) and eretmochelys imbricata (linnaeus, 1766) in the southwest atlantic. Marine Turtle Newsletter 113:6-7

Sounguet GP (2006) Leatherback nesting in Gabon. In: Mast RB, Bailey LM, Hutchinson BH (eds) SWoT Report—The State of the World's Sea Turtles, Volume I, Washington, DC. 36 p. Available online *at*: http://seaturtlestatus.org/report/view

Sounguet GP (2007) Leatherback nesting in Gabon. In: Mast RB, Bailey LM, Hutchinson BH (eds) SWoT Report—The State of the World's Sea Turtles, Volume II, Washington, DC. 49p. Available online at: http://seaturtlestatus.org/report/view

Sounguet GP, Mbina C, Formia A (2004) Sea turtle research and conservation in Gabon by Aventures Sans Frontières, an organizational profile. Marine Turtle Newsletter 105:19-21

Spotila JR, Dunham AE, Leslie AJ, Steyermark AC, Plotkin PT, Paladino FV (1996) Worldwide population decline of Dermochelys coriacea: are leatherback turtles going extinct? Chelonian Conservation and Biology 2:209-222

Spotila JR, Reina RD, Steyermark AC, Plotkin PT, Paladino FV (2000) Pacific leatherback turtles face extinction. Nature 405

St. Kitts Sea Turtle Monitoring Network (2008) Hawksbill nesting in St. Kitts, Ross University School of Veterinary Medicine. In: Mast RB, Bailey LM, Hutchinson BH (eds) SWoT Report—The State of the World's Sea Turtles, Volume III, Washington, DC. 43p. Available online at: http://seaturtlestatus.org/report/view

St. Lucia Department of Fisheries (2006) Preliminary sea turtle figures for Grande Anse Beach, Saint Lucia. In: Mast RB, Bailey LM, Hutchinson BH (eds) SWoT Report—The State of the World's Sea Turtles, Volume I, Washington, DC. 36 p. Available online at: http://seaturtlestatus.org/report/view

Stapleton S, Stapleton C (2006) Tagging and Nesting Research on Hawksbill Turtle (*Eretmochelys imbricata*) at Jumby Bay, Long Island, Antigua, West Indies

Stephens SH (2003) Genetic analysis of the kemp.s ridley sea turtle (*lepidochelys kempii*) with estimates of effective population size. Master of Science, Texas A&M University

Stewart K, DeFreitas R, Kalamandeen M, Pritchard P (2006) Aspects of Marine Turtle Nesting in Guyana, 2005. Guianas Forests and Environmental Conservation Project (GFECP) World Wildlife Fund: Technical Report:16 p

Stewart K, Keller JM, Johnson C, Kucklick JR (2007) Baseline containment concentrations in leatherback sea turtles and maternal transfer to eggs confirmed. In: Rees AF, Frick MA, Panagopoulou AF, Williams K (eds) Proceedings of the Twenty-Seventh Annual Symposium on Sea Turtle Biology and Conservation. NOAA Technical Memorandum NMFS-SEFSC-569, Mrytle Beach, South Carolina. 261 pp., p 30

Stewart K, St. Kitts Sea Turtle Monitoring Network (2006) Leatherback nesting in St. Kitts. In: Mast RB, Bailey LM, Hutchinson BH (eds) SWoT Report—The State of the World's Sea Turtles, Volume I, Washington, DC. 36 p. Available online at: http://seaturtlestatus.org/report/view

Stewart K, St. Kitts Sea Turtle Monitoring Network (2007) Personal communication. Leatherback nesting in St. Kitts. In: Mast RB, Bailey LM, Hutchinson BH (eds) SWoT Report—The State of the World's Sea Turtles, Volume II, Washington, DC. 49p. Available online at: http://seaturtlestatus.org/report/view

Stivalet CJC, J.O. Díaz A (2002) Campamento Tortuguero El Callejón. Temporadas 1999-2001-2002, Desarrollo turístico y Rancho Istirincha. Carretera Federal 180. Km. 102. Nautla-Cardel. Inédito

Stringell T, Bangkaru M (2002) Egg harvesting, predation, and green turtle conservation in Pulau Banyak, Indonesia. In: Seminoff JA (ed) Proceedings of the Twenty-Second Annual Symposium on SeaTurtle Biology and Conservation. U.S. Dep. Commer. NOAA Tech. Memo. NMFS-SEFSC-503, Miami, Florida. 308 p, p 164-165

Stringell T, Bangkaru M, Steeman APJM, Bateman L (2000) Green Turtle Nesting at Pulau Banyak (Sumatra, Indonesia). Marine Turtle Newsletter 90:6-8

Suganuma H (1985) Green Turtle Research Program in Ogasawara. Marine Turtle Newsletter 33:2-3

Sunderraj SFW Pers. comm.

Sunderraj SFW, Joshua J, Kumar VV (2006) Sea turtles and their nesting habitats in Gujarat. In: Shanker K, Choudhury BC (eds) Marine Turtles of the Indian Subcontinent. Universities Press, India, Hyderguda, p 156-169

Sunderraj SFW, Joshua J, Serebiah S (2001) Sea Turtles along the Gujarat Coast. Kachhapa 5:14-16

Supreme Council for the Environment and Natural Reserves (2006) Status of Sea Turtles in Qatar *In*, Doha, Qatar. 130 p
Suwelo IS (1999) Olive Ridley Turtle Records from South Banyuwangi, East Java. Marine Turtle Newsletter 85:9
Svendson B, Stewart K, St. Kitts Sea Turtle Monitoring Network (2006) Leatherback nesting in St. Kitts. In: Mast RB, Bailey LM, Hutchinson BH (eds) SWoT Report—The State of the World's Sea Turtles, Volume I, Washington, DC. 36 p. Available online *at*: http://seaturtlestatus.org/report/view
Sybesma J (1992) Sea Turtle Recovery Action Plan for the Netherlands Antilles UNEP Caribbean Environment Programme, Kingston, Jamaica. 63 p
Talbert OR, Jr., Stancyk SE, Dean JM, Will JM (1980) Nesting Activity of the Loggerhead Turtle (Caretta caretta) in South Carolina I: A Rookery in Transition. Copeia 1980:709-719
Talma E (2006) Interim report on 2005–06 Turtle Nesting Season in the South of Mahe, Seychelles. Marine Conservation Society Seychelles technical report
Talma E (2008) Report on Turtle Nesting Activity recorded by MCSS and Banyan Tree Resort in the South of Mahe, Seychelles, during the 2007-08 Season. Marine Conservation Society Seychelles (MCSS) technical report, 77 p
Talma E (2008) Personal Communication.
Talma E, Matombe R (2008) Personal Communication.
TAMAR P (2008?) Database.
Taquet C, Taquet M, Dempster T, Soria M, Ciccione S, Roos D, Dagorn L (2006) Foraging of the green sea turtle Chelonia mydas on seagrass beds at MayotteIsland (Indian Ocean), determined by acoustic transmitters. Marine Ecology Progress Series 306:295-302
Taskavak E, Türkozan O, Kiremit F, Turkecan O, Guclu O, Akcinar C, Yilmaz C, Tuncay D (2006) A review of 2005 marine turtle nesting season on five beaches (Dalyan, Fethiye, Patara, Belek, Goksu Delta) ın Turkey. In: Frick MA, Panagopoulou A, Rees AF, Williams K (eds) Twenty sixth Annual Symposium on Sea Turtle Biology and Conservation, Athens, Greece. 376 p, p 328
Tayab MR, Quiton P (2003) Marine Turtle Conservation Initiatives at Ras Laffan Industrial City, Qatar (Arabian Gulf). Marine Turtle Newsletter 99:14-15
TCP (1999) TCP beach survey report: Ecological assessment of marine turtles on Sri Lanka's south coast between Rekawa and Godavaya. Unpublished report submitted to IUCN, Sri Lanka
Tennant M (2008) Hawksbill nesting on Gibraltar Beach, Jamaica. In: Mast RB, Bailey LM, Hutchinson BH (eds) SWoT Report—The State of the World's Sea Turtles, Volume III, Washington, DC. 43p. Available online *at*: http://seaturtlestatus.org/report/view
The Leatherback Trust: Las Baulas Conservation Project (2004-2005) Archived field report. Available online *at* http://www.leatherback.org/pages/project/report/report0405.htm.
The Turtle Conservation Project (2008) Turtle nesting beaches in Sri Lanka. Available online *at* www.tcpsrilanka.org/download/Map.pdf
Thebu J, Hitipeuw CT (2005) Leatherback conservation at Warmon beach, Papua-Indonesia: November 2003-October 2004. In: Kinan I (ed) Proceedings of the Second Western Pacific Sea Turtle Cooperative Research and Management Workshop Volume I: West Pacific Leatherback and Southwest Pacific Hawksbill Sea Turtles. Western Pacific Regional Fishery Management Council, Honolulu, Hawaii, USA. 118 p p19-23
Thome JCA, Baptistotte C, Almeida AP (2008) *Dermochelys coriacea* (Vandelli, 1761). In: Livro vermelho da fauna brasileira ameaçada de extinção Brasília: MMA, v2, Biodiversitas, 19, p 366-367 *in*
Thome JCA, Baptistotte C, de Moreira LM, Scalfoni JT, Almeida AP, Rieth DB, Barata PCR (2007) Nesting biology and conservation of the leatherback sea turtle (*Dermochelys coriacea*) in the state of Espirito Santo, Brazil, 1988-1989 to 2003-2004. Chelonian Conservation and Biology 6:15-27
Thomé JCA, Marcovaldi MA, dei Marcovaldi GG, Bellini C, Gallo BMG, Lima EHSM, da Silva ACCD, Sales G, Barata PCR (2003) An overview of Projeto TAMAR-IBAMA's activities in relation to the incidental capture of sea turtles in the

Brazilian fisheries. In: Seminoff JA (ed) Proceedings of the Twenty-second Annual Symposium on Sea Turtle Biology and Conservation. NOAA Technical Memorandum NMFSSEFSC-503, Miami, Florida. 308 p, p 119-120

Thorbjarnarson JB, Platt SG, Khaing ST (2000) Sea Turtles in Myanmar: Past and Present. Marine Turtle Newsletter 88:10-11

Tisen OB, Bali J (2002) Current status of marine turtle conservation programmes in Sarawak, Malaysia. In: Mosier A, Foley A, Brost B (eds) Proceedings of the Twentieth Annual Symposium on Sea Turtle Biology and Conservation. NOAA Technical Memorandum NMFS-SEFSC-477, Orlando, Florida, 369 p, p 12-14

Tiwari M (2007) Leatherback turtle (*Dermochelys coriacea*) five-year review. National Marine Fisheries Service & US Fish and Wildlife Service, Jacksonville, FL, 81 p

Tiwari M, Bjorndal KA, Bolten AB (2002) Thirty years of spatial and temporal patterns in distribution of emergences by nesting green turtles in Tortuguero, Costa Rica. In: Seminoff JA (ed) Proceedings of the Twenty-Second Annual Symposium on SeaTurtle Biology and Conservation. NOAA Technical Memorandum NMFS-SEFSC-503, Miami, Florida. 308 p, p 1

TM W, GH B (2010) Pathology and distribution of sea turtles landed as bycatch in the Hawaii-based North Pacific longline fishery. Journal of Wildlife Diseases 46:422-432

Tomas J, Castroviejo J, Raga JA (1999) Sea turtles in the South of Bioko Island (Equatorial Guinea). Marine Turtle Newsletter 84:4-6

Tomas J, Castroviejo J, Raga JA (2000) Sea Turtles in the South of Bioko Island (Equatorial Guinea), Africa. In: Kalb H, Wibbels T (eds) Proceedings of the nineteenth annual symposium on sea turtle conservation and biology. NOAA Technical Memorandum NMFS-SEFSC-443, South Padre Island, Texas, U.S.A. 291 p., p 247-250

Tomas J, Formia A, Castoviejo J, Raga JA (2001) Post-nesting movements of the green turtle, *Chelonia mydas*, nesting in the south of Bioko Island, Equatorial Guinea, West Africa. Marine Turtle Newsletter 94:3-6

Tomás J, León YM, Feliz P, Geraldes FX, Broderick AC, Fernández M, Godley BJ, Raga JA (2007) Sea turtle nesting populations of the Dominican Republic *In* The 14th European Congress of Herpetology, Porto, Portugal

Tomas J, Mons JL, Martin JJ, Bellido JJ, Castillo JJ (2003) First nesting activity of the loggerhead sea turtle, Caretta caretta, in the Spanish Mediterranean coast. In: Seminoff JA (ed) Proceedings of the Twenty-Second Annual Symposium on SeaTurtle Biology and Conservation. NOAA Technical Memorandum NMFS-SEFSC-503, Miami, Florida. 308 p, p 166-167

Tomas J, Raga J (2007) Occurrence of Kemp's ridley sea turtle (Lepidochelys kempii) in the Mediterranean. Journal of Marine Biological Association of the UK Biodiversity Records; On-line:1-3

TOPP (Tagging of Pacific Pelagics) (2005-2006) Leatherbacks help to map Pacific. In: Mast RB, Bailey LM, Hutchinson BH (eds) SWoT Report Volume I, Wasington, DC. 36 p. Available online *at*: http://seaturtlestatus.org/report/view, p 21

Torres D, Santa Cruz E, Mansanero LI, Santa Cruz GA (2004) Conservation of a Remnant Hawksbill (*Eretmochelys imbricata*) Population Nesting In Punta Dumalag Barangay Matina Aplaya, Davao City, Philippines. *In*. In: Agham Mindanao-2004 35-39, Vol 2. Ateneo de Davao University

Trejo A (2007) Personal Communication. Olive ridley population estimates in Playon de Mismaloya, Jalisco, Mexico. Cited in Marine Turtle Specialist Group. 2007. Red List Assessment-Lepidochelys olivacea. 39 p.

Trejos JA, Carretero E (2006) Leatherback nesting in Mexico. In: Mast RB, Bailey LM, Hutchinson BH (eds) SWoT Report—The State of the World's Sea Turtles, Volume I, Washington, DC. 36 p. Available online *at*: http://seaturtlestatus.org/report/view

Trigo HS (2005) Informe de la temporada 2005, Centro para la Protección y conservación de las tortugas marinas. Central Núcleo-eléctrica Laguna Verde, 12 p. Inédito

Tripathy B (2002) Is Gahirmatha the world's largest sea turtle rookery? Current Science 83:1299

Tripathy B, Choudhury BC (2002) Recent Sightings of the Green Turtle Chelonia

mydas on the Coast of Andhra Pradesh, India. Marine Turtle Newsletter 98:3-4
Tripathy B, Kumar RS, Choudhury BC, Sivakumar K, Nayak AK (2009) Compilation of Research Information on Biological and Behavioural Aspects of Olive Ridley Turtles along the Orissa Coast of India – A Bibliographical Review for Identifying Gap Areas of Research. Wildlife Institute of India, Dehra Dun
Tripathy B, Shanker K, Choudhury BC (2003) Important nesting habitats of olive ridley turtles *Lepidochelys olivacea* along the Andhra Pradesh coast of eastern India. Oryx 37:454-463
Tripathy B, Shanker K, Choudhury BC (2006) Sea Turtles and their Habitats in the Lakshadweep Islands. In: Shanker K, Choudhury BC (eds) Marine Turtles of The Indian Subcontinent. Universities Press, India, Hyderguda, p 119-136
Troëng S, Chacon D, Dick B (2004a) Leatherback turtle *Dermochelys coriacea* nesting along the Caribbean coast of Costa Rica. In: Coyne MS, Clarke (eds) Proceedings of the Twenty-First Annual Symposium on Sea Turtle Biology and Conservation. NOAA Technical Memorandum NMFS-SEFSC-528, Philadelphia, Pennsylvania, 368 pp., p 13
Troëng S, Chacón D, Dick B (2004) Possible decline in leatherback turtle *Dermochelys coriacea* nesting along Caribbean Central America. Oryx 38:395-403
Troëng S, Chaloupka M (2007) Variation in adult annual survival probability and remigration intervals of sea turtles. Marine Biology 151:1721-1730
Troëng S, Drews C (2004b) Money Talks: Economic Aspects of Marine Turtle Use and Conservation. WWF-International, Gland, Switzerland wwwpandaorg
Troëng S, Dutton PH, Evans D (2005a) Migration of hawksbill turtles *Eretmochelys imbricata* from Tortuguero, Costa Rica. Ecography 28:394-402
Troeng S, Harrison E, Evans D, de Haro A, Vargas E (2007) Leatherback turtle nesting trends and threats at Tortuguero, Costa Rica Chelonian Conservation and Biology 6:117-122
Troeng S, Harrison E, Evans D, Haro Ad, Vargas E (2007) Leatherback Turtle Nesting Trends and Threats at Tortuguero, Costa Rica. Chelonian Conservation and Biology 6:117-122
Troëng S, Mangel J, Reyes C (2002) Comparison of Monel 49 and Inconel 681 flipper tag loss in green turtles, *Chelonia mydas*, nesting at Tortuguero, Costa Rica. In: Seminoff JA (ed) Proceedings of the Twenty-Second Annual Symposium on SeaTurtle Biology and Conservation. NOAA Technical Memorandum NMFS-SEFSC-503, Miami, Florida. 308 p, p 121-122
Troëng S, Rankin E (2005b) Long-term conservation efforts contribute to positive green turtle *Chelonia mydas* nesting trend at Tortuguero, Costa Rica. Biological Conservation 121:111-116
Troncoso F, Urbina M (2007) Actualizacion del registro de tortugas marinas para el Sur de Chile. In: VII Simposio sobre medio ambiente: Estado Actual y Perspectivas de la Investigacion y Conservacion de las Tortugas Marinas en las Costas del Pacifico Sur Oriental (ed CREA), p 41, Antofagasta, Chile:41
Trono RB (1991) Philippine Marine Turtle Conservation Program. Marine Turtle Newsletter 53:5-7
Turkozan O (2006) Preliminary results for the population trends of two index beaches, Fethiye and Dalyan. In: Frick MA, Panagopoulou A, Rees AF, Williams K (eds) Twenty sixth Annual Symposium on Sea Turtle Biology and Conservation, Athens, Greece. 376 p, p 330
Turkozan O, C Y (2008) Loggerhead turtles, Caretta caretta, at Dalyan Beach, Turkey: Nesting activity (2004-2005) and 19-year abundance trend (1987-2005). Chelonian Conservation and Biology 7:178-187
Türkozan O, Kiremit FT (In preparation) Nesting activity of the loggerhead turtle on Patara beach, Turkey and possible nesting trend in the last 10 years.
Turkozan O, Ylmaz C (In review) Population Trend of Loggerhead Turtle, *Caretta caretta*, on Dalyan Beach, Turkey. Results of 2004-2005 Nesting Season.
Turtle and Marine Ecosystem Center (TUMEC), Fisheries Department of Malaysia (2006) Leatherback nesting in Malaysia. In: Mast RB, Bailey LM, Hutchinson BH (eds) SWoT Report—The State of the World's Sea Turtles, Volume I, Washington, DC. 36 p. Available online *at*: http://seaturtlestatus.org/report/view

Turtle Expert Working Group (1998) An assessment of the Kemp's ridley (*Lepidochelys kempii*) and loggerhead (*Caretta caretta*) sea turtle populations in the western North Atlantic. NOAA Technical Memorandum NMFS-SEFSC-409:105 pp.
Turtle Expert Working Group (2000) Assessment update for the Kemp's ridley and loggerhead sea turtle populations in the western North Atlantic. NOAA Technical Memorandum NMFS-SEFSC-444:132 pp.
Turtle Expert Working Group (2007) An assessment of the Leatherback Turtle Population in the Atlantic Ocean. NOAA Technical Memorandum NMFS-SEFSC-555:124 pp.
Turtugaruba (Aruban Foundation for Sea Turtle Protection and Conservation) (2006) Leatherback nesting data from Eagle Beach, Dos Playa and Boca Grandi, Aruba, 2004. In: Mast RB, Bailey LM, Hutchinson BH (eds) SWoT Report—The State of the World's Sea Turtles, Volume I, Washington, DC. 36 p. Available online *at*: http://seaturtlestatus.org/report/view
U.S. Fish and Wildlife Service, Gladys Porter Zoo, Secretaria de Medio Ambiente y Recursos Naturales, Secretaria de Desarrollo Urbano y Ecologia (2005) Report on the Mexico/United States of America population restoration project for the Kemp's ridley sea turtle, *Lepidochelys kempii*, on the coasts of Tamaulipas and Veracruz, Mexico: 2005. Gladys Porter Zoo, Brownsville, TX
United Nations Environment Programme: World Conservation Monitoring Centre (2006) Loggerhead - *Caretta caretta*. Available online *at* http://www.unep-wcmc.org/species/data/species_sheets/loggerhe.htm
United States Fish and Wildlife Service (2006) Fort Morgan Sea Turtle Nesting Summary., 4 p.
URS (2007) Nearshore Monitoring of Turtle Nesting and Hatchling Activities for Dolphin Energy Limited - 2007, Doha, Qatar. 41 p
Urteaga J (2005) Conservación de Tortugas Tora, *Dermochelys coriacea*, en el Refugio de Vida Silvestre Río Escalante-Chacocente, Temporada 2004-05. Informe tecnico de proyecto. Fauna & Flora International. In: Mast RB, Bailey LM, Hutchinson BH (eds) SWoT Report—The State of the World's Sea Turtles, Volume II, Washington, DC. 49p. Available online *at*: http://seaturtlestatus.org/report/view
Urteaga J (2008) Personal communication. Hawksbill nesting in Nicaragua. In: Mast RB, Bailey LM, Hutchinson BH (eds) SWoT Report—The State of the World's Sea Turtles, Volume III, Washington, DC. 43p. Available online *at*: http://seaturtlestatus.org/report/view
Urteaga JR (2004) Conservación de tortugas tora, *Dermochelys coriacea*, en el Refugio de Vida Silvestre Río Escalante – Chacocente: Temporada 2003-2004, informe anual. Nicaragua: Fauna and Flora International
Urteaga R, Motha S (2007) Resultados de Monitoreo de Tortugas Marinas en el Pacífico de Nicaragua. Temporada 2006-07. Unpublished report submitted to MARENA March, 2007
Valverde RA, Cornelius S, Mo CL (1998) Decline of the olive ridley sea turtle (*Lepidochelys olivacea*) nesting assemblage at Nancite Beach, Santa Rosa National Park, Costa Rica. Chelonian Conservation and Biology 3:58-63
Valverde RA, Taft C, Godley D (1998) Green turtle program at Tortuguero, Costa Rica. In: Epperly S, Braun J (eds) Proceedings of the seventeenth annual sea turtle symposium. U.S. Dep. Commer. NOAA Tech. Memo. NMFS-SEFSC-415, Orlando, Florida. 342 p.
Van der Elst R (2007) Personal communication. Loggerhead nesting in Inhaca, Mozambique. In: Mast RB, Bailey LM, Hutchinson BH (eds) SWoT Report—The State of the World's Sea Turtles, Volume II, Washington, DC. 49p. Available online *at*: http://seaturtlestatus.org/report/view
Van der Wal E, Van der Wal R, Turtugaruba (Aruban Foundation for Sea Turtle Protection and Conservation) (2005) Monitoring Aruba 2005, Turtugaruba - Aruban Foundation for sea turtle protection and conservation. In: Mast RB, Bailey LM, Hutchinson BH (eds) SWoT Report—The State of the World's Sea Turtles, Volume II, Washington, DC. 49p. Available online *at*: http://seaturtlestatus.org/report/view
Van der Wal E, Van der Wal R, Turtugaruba (Aruban Foundation for Sea Turtle

Protection and Conservation) (2006) Personal communication. Loggerhead nesting in Aruba. In: Mast RB, Bailey LM, Hutchinson BH (eds) SWoT Report—The State of the World's Sea Turtles, Volume II, Washington, DC. 49p. Available online *at*: http://seaturtlestatus.org/report/view

Van der Wal E, Van der Wal R, Turtugaruba (Aruban Foundation for Sea Turtle Protection and Conservation) (2008) Hawksbill nesting in Aruba. In: Mast RB, Bailey LM, Hutchinson BH (eds) SWoT Report—The State of the World's Sea Turtles, Volume III, Washington, DC. 43p. Available online *at*: http://seaturtlestatus.org/report/view

Vanherck L (2008) Personal communication. Hawksbill nesting on North Island, Seychelles. In: Mast RB, Bailey LM, Hutchinson BH (eds) SWoT Report—The State of the World's Sea Turtles, Volume III, Washington, DC. 43p. Available online *at*: http://seaturtlestatus.org/report/view

Varela RA, Lutz AP, Cray C, Bossart G (1998) Cell-mediated immunology of green turtle fibropapillomatosis. In: Epperly S, Braun J (eds) Proceedings of the seventeenth annual sea turtle symposium. U.S. Dep. Commer. NOAA Tech. Memo. NMFS-SEFSC-415, Orlando, Florida. 342 p.

Vargas SF, Vasconcelos D, Ángeles MA, Licea M (2004) Informe final de investigación de las actividades de conservación desarrollados en la Playa de Tierra Colorada durante la temporada 2003-2004. In: Sarti M, L., Barragán RAR, Juárez C JA (eds) Conservación y evaluación de la población de tortuga laúd *Dermochelys coriacea* en el Pacífico Mexicano, temporada de anidación 2003-2004. DGVS-SEMARNAT-Kutzari, Asociación para el Estudio y Conservación de las Tortugas Marinas A. C

Vargas SM, Araujo FC, Monteiro D, Estima SC, Thome JCA, Soares LS, Santos FR (2007) Occurence of mtDNA haplotypes of leatherback turtles on the Brazilian coast. In: Frick MA, Panagopoulou AF, Rees AF, Williams K (eds) Proceedings of the Twenty-Seventh Annual Symposium on Sea Turtle Biology and Conservation. NOAA Technical Memorandum NMFS-SEFSC-569, Mrytle Beach, South Carolina. 261 pp., p 167

Vargas SM, Araujo FCF, Monteiro DS, Estima SC, Almeida AP, Soares LS, Santos FR (2008) Genetic diversity and origin of leatherback turtles (*Dermochelys coriacea*) from the Brazilian coast. Journal of Heredity 99:215-220

Velez-Zuazo X, Kelez S (In Press) Multiyear analysis of sea turtle bycatch by Peruvian longline fisheries: a genetic perspective. Proceedings from the 30th Annual Symposium on Sea Turtle Biology and Conservation, Goa, India

Velez-Zuazo X, Ramos WD, Van Dam R, Diez CE, Abreu-Grobois FA, McMillan WO (2008) Dispersal, recruitment and migratory behaviour in a hawksbill sea turtle aggregation. Molecular Ecology 17:839-853

Venegas R (2006) Report of Activities and Research in Pacuare Reserve, 2006 Season. Pacuare Reserve Marine Turtle Conservation Project, Playa Mondonguillo Endangered Wildlife Trust unpublished report

Vera V (2002) Restarting the green turtle (Chelonia mydas) tagging and conservation project in Aves Island Wildlife Refuge, Venezuela. In: Seminoff JA (ed) Proceedings of the Twenty-Second Annual Symposium on SeaTurtle Biology and Conservation. U.S. Dep. Commer. NOAA Tech. Memo. NMFS-SEFSC-503, Miami, Florida. 308 p, p 171-172

Vera V (2004) Personal Communication to K. Eckert. Cited in Seminoff, J.A., (assessor). 2004. Global Status Assessment: Green turtle (Chelonia mydas). Marine Turtle Specialist Group. Species Survival Commission, Red List Programme: 71.

Vera V (2007) Nesting of green turtles in Aves Island Wildlife Refuge. 2006 season. In: Rees AF, Frick MA, Panagopoulou A, Williams JA (eds) Twenty-seventh annual symposium on sea turtle biology and conservation. NOAA Technical Memorandum NMFS-SEFSC-569, Myrtle Beach, South Carolina. 261 pp., p 275

Vera V, Montilla A (2006) Results of sea turtle nesting on Aves Island, Venezuela: 2005 season. In: Frick MA, Panagopoulou A, Rees AF, Williams K (eds) Twenty sixth Annual Symposium on Sea Turtle Biology and Conservation, Athens, Greece. 376 pp., p 331

Verhage B, Moundjim E, B. (2006) Four years of marine turtle monitoring in the Gamba

Complex of Protected Areas, Gabon, Central Africa, 2002-2006. In: Mast RB, Bailey LM, Hutchinson BH (eds) SWoT Report—The State of the World's Sea Turtles, Volume II, Washington, DC. 49p. Available online *at*: http://seaturtlestatus.org/report/view

Verhage B, Moundjim EB (2005) Three years of marine turtle monitoring in the Gamba Complex of Protected Areas, Gabon, Central Africa, 2002-2005. In: Mast RB, Bailey LM, Hutchinson BH (eds) SWoT Report—The State of the World's Sea Turtles, Volume I, Washington, DC. 36 p. Available online *at*: http://seaturtlestatus.org/report/view

Videira EJS, Louro CMM (Forthcoming) Análise da monitoria de ninhos e marcação de tartarugas marinhas no Parque Nacional do Arquipélago do Bazaruto, Moçambique.

Vissenberg D (2006) Personal communication. Leatherback nesting in St. Maarten, Netherlands Antilles. In: Mast RB, Bailey LM, Hutchinson BH (eds) SWoT Report—The State of the World's Sea Turtles, Volume II, Washington, DC. 49p. Available online *at*: http://seaturtlestatus.org/report/view

Vissenberg D, Nature Foundation (2008) Hawksbill nesting in Guana Bay, St. Maarten. In: Mast RB, Bailey LM, Hutchinson BH (eds) SWoT Report—The State of the World's Sea Turtles, Volume III, Washington, DC. 43p. Available online *at*: http://seaturtlestatus.org/report/view

Wagiman S, Malaysia Fisheries Department (2008) Hawksbill nesting in Johor, Malacca, Pahang, and Terengganu, Malaysia. In: Mast RB, Bailey LM, Hutchinson BH (eds) SWoT Report—The State of the World's Sea Turtles, Volume III, Washington, DC. 43p. Available online *at*: http://seaturtlestatus.org/report/view

Wallace B, Lewison R, McDonald S, McDonald R, Kot C, Kelez S, Bjorkland R, Finkbeiner E, Helmbrecht S, Crowder L (2010) Global patterns of marine turtle bycatch. Conservation Letters 3:131-142

wan Smolbag GP (2008) Personal communication.

Ward J, Asotasi I (2008) An assessment on the current status of the Hawksbill turtles (*Eretmochelys imbricata*) on the Aleipata islands, 2007-2008. Samoa: Division of Environment and Conservation, Ministry of Natural Resources and Environment unpublished report

Watson DM (2006) Growth rates of sea turtles in Watamu, Kenya. Earth & Environment 2:29-53

Webster WD, Cook KA (2001) Intraseasonal Nesting Activity of Loggerhead Sea Turtles (Caretta caretta) in Southeastern North Carolina. American Midland Naturalist 145:66-73

Weijerman M, van Tienen LHG, Schouten AD, Hoekert WEJ (1998) Sea turtles of Galibi, Suriname. In: Byles R, Fernandez Y (eds) Proceedings of the Sixteenth Annual Symposium on Sea Turtle Biology and Conservation. NOAA Technical Memorandum NMFS-SEFSC-412, Hilton Head, South Carolina. 158 p., p 142-144.

Weir CR, Ron T, Morais M, Duarte ADC (2007) Nesting and at-sea distribution of marine turtles in Angola, West Africa, 2000–2006: occurrence, threats and conservation implications. Oryx 41:224-231

Weisler MI (1994) The Settlement of Marginal Polynesia: New Evidence from Henderson Island. Journal of Field Archaeology 21:83-102

Wetherall JA, Balazs GH, Yong MYY (1998) Statistical methods for green turtle nesting surveys in the Hawaiian Islands. In: Epperly S, Braun J (eds) Proceedings of the seventeenth annual sea turtle symposium. U.S. Dep. Commer. NOAA Tech. Memo. NMFS-SEFSC-415, Orlando, Florida. 342 p.

White M, Haxhiu I, Sacdanaku E, Petritaj L, Rumano M, Osmani F, Vrenozi B, Robinson P, Kouris S, Boura L, Venizelos L (2009) Monitoring and Conservation of Important Sea Turtle Feeding Grounds in the Patok Area of Albania. 2008 Annual Report. Joint project of: MEDASSET; GEF/SGP; RAC/SPA (UNEP/MAP); Ministry of Environment, Albania; Natural History Museum, Albania; HAS, Albania; University of Tirana; ECAT, Albania:91 pp.

Whiting A (in press) Flatback Red List Assessment (draft). IUCN Conservation Library Gland

Whiting AU, Thomson A, Chaloupka MY, Limpus CJ (2008) Seasonality, abundance, and breeding biology of one of the largest populations of nesting flatback turtles, *Natator depressus*: Cape Domett, Western Australia. Australian Journal of Zoology 56:297-303

Whiting S (1997) Observations of a nesting olive ridley turtle in the Northern Territory. Herpetofauna 27:39-42

Whiting SD, Long JL, Coyne MS (2007a) Migration routes and foraging behaviour of olive ridley turtles *Lepidochelys olivacea* in northern Australia. Endangered Species Research 3:1-9

Whiting SD, Long JL, Hadden KM, Lauder ADK, Koch AU (2007b) Insights into size, seasonality and biology of a nesting population of the olive ridley turtle in northern Australia. Wildlife Research 34:200-210

Whiting SD, Miller JD (1998) Short Term Foraging Ranges of Adult Green Turtles (Chelonia mydas). Journal of Herpetology 32:330-337

Wibbels T (2007) Kemp's ridley sea turtle (Lepidochelys kempii) five-year review: summary and evaluation, NMFS & USFWS, Jacksonville, FL, USA

WIDECAST (2009) Basic biology of the green turtle. WIDECAST network. Online *at* http://www.widecast.org/Biology/Green.html

Williams JA, Renaud ML (1998) Tracking of Kemp's ridley (Lepidochelys kempii) and green (Chelonia mydas) sea turtles in the Matagorda Bay system, Texas. In: Epperly S, Braun J (eds) Proceedings of the seventeenth annual sea turtle symposium. U.S. Dep. Commer. NOAA Tech. Memo. NMFS-SEFSC-415, Orlando, Florida. 342 p.

Witherington B, Kubilis P, Brost B, Meylan A (2009) Decreasing annual nest counts in a globally important loggerhead sea turtle population. Ecological Applications 19:30-54

Witt MJ, Baert B, Broderick AC, Formia A, Fretey J, Gibudi A, Mounguengui Mounguengui GA, Moussounda C, Ngouessono S, Parnell RJ, Roumet D, Sounguet G-P, Verhage B, Zogo A, Godley AC (2009) Aerial surveying of the world's largest leatherback turtle rookery: A more effective methodology for large-scale monitoring. Biological Conservation 142:1719-1727

Witt MJ, Broderick AC, Coyne MS, Formia A, Ngouessono S, Parnell RJ, Sounguet GP, Godley BJ (2008) Satellite tracking highlights difficulties in the design of effective protected areas for Critically Endangered leatherback turtles *Dermochelys coriacea* during the inter-nesting period. Oryx 42:296-300

Witt MJ, Penrose R, Godley BJ (2007) Spatio-temporal patterns of juvenile marine turtle occurrence in waters of the European continental shelf. Marine Biolo 151:873-885

Witzell W (2002) Immature Atlantic loggerhead turtles (Caretta caretta): Suggested changes to the life history model. Herpetological Review 33:266-269

Work TM, Balazs GH (1998) Causes of green turtle (Chelonia mydas) morbidity and mortality in Hawaii. In: Epperly S, Braun J (eds) Proceedings of the seventeenth annual sea turtle symposium. U.S. Dep. Commer. NOAA Tech. Memo. NMFS-SEFSC-415, Orlando, Florida. 342 p.

Work TM, Balazs GH, Wolcott M, Morris R (2002) Bacteremia in free-ranging Hawaiian green turtles with Fibropapillomatosis. In: Seminoff JA (ed) Proceedings of the Twenty-Second Annual Symposium on SeaTurtle Biology and Conservation. U.S. Dep. Commer. NOAA Tech. Memo. NMFS-SEFSC-503, Miami, Florida. 308 p, p 309

World Wildlife Fund (2003) Report on a rapid ecological assessment of the Raja Ampat Islands, Papua, Eastern Indonesia,. World Wildlife Fund report 246 p

World Wildlife Fund (2005) The status of marine turtles in the United Republic of Tanzania, east Africa. World Wildlife Fund report 35 p

World Wildlife Fund (2005) Marine Turtle Conservation Activities in Mozambique, August 2004 to June 2005. Maputo, Mozambique: Marine Programme, WWF Mozambique

WWF Italia News 2007 (2007) Available online *at*: http://beta.wwf.it/client/ricerca.aspx?root=13085&parent=1979&content=1.

Wyneken J, Rhodin AGJ, Garces A, Rhodin JAG (2002) Cardiopulmonary structure and function in leatherback and green sea turtles. In: Seminoff JA (ed)

Proceedings of the Twenty-Second Annual Symposium on Sea Turtle Biology and Conservation. U.S. Dep. Commer. NOAA Tech. Memo. NMFS-SEFSC-503, Miami, Florida. 308 p, p 28

Yalçın-Özdilek Ş, Sönmez B (2007) Personal communication. Loggerhead nesting in Samandag, Hatay, Turkey. In: Mast RB, Bailey LM, Hutchinson BH (eds) SWoT Report—The State of the World's Sea Turtles, Volume II, Washington, DC. 49p. Available online *at*: http://seaturtlestatus.org/report/view

Yasuda T, Tanaka H, Kittiwattanwong K, Mitamura H, Klom-in W, Arai N (2006) Do female green turtles (Chelonia mydas) exhibit reproductive seasonality in a year-round nesting rookery?. Journal of Zoology 269:451-457

Zahir H (2008) Personal communication. Hawksbill nesting in Maldives. In: Mast RB, Bailey LM, Hutchinson BH (eds) SWoT Report—The State of the World's Sea Turtles, Volume III, Washington, DC. 43p. Available online *at*: http://seaturtlestatus.org/report/view

Zarate P (2008) Personal communication. Hawksbill nesting in Ecuador. In: Mast RB, Bailey LM, Hutchinson BH (eds) SWoT Report—The State of the World's Sea Turtles, Volume III, Washington, DC. 43p. Available online *at*: http://seaturtlestatus.org/report/view

Zarate P, Fernie A, Dutton DM (2002) First results of the East Pacific green turtle, Chelonia mydas, nesting population assessment in the Galapagos Islands. In: Seminoff JA (ed) Proceedings of the Twenty-Second Annual Symposium on SeaTurtle Biology and Conservation. U.S. Dep. Commer. NOAA Tech. Memo. NMFS-SEFSC-503, Miami, Florida. 308 p, p 70-73

Zug GR, Chaloupka M, Balazs GH (2006) Age and growth in olive ridley seaturtles (Lepidochelys olivacea) from the North-central Pacific: a skeletochronological analysis. Marine ecology 27:263-270

Zurita JC, Herrera R, Arenas A, Torres ME, Calderon C, Gomez L, Alvarado JC, Villavicencio R (2002) Nesting loggerhead and green sea turtles in Quintana Roo, Mexico. In: seminoff JA (ed) Proceedings of the Twenty-Second Annual Symposium on SeaTurtle Biology and Conservation. U.S. Dep. Commer. NOAA Tech. Memo. NMFS-SEFSC-503, Miami, Florida. 308 p, p 125-127
